# Functional Authentication of a Novel Gastropod Gonadotropin-Releasing Hormone Receptor Reveals Unusual Features and Evolutionary Insight

**DOI:** 10.1371/journal.pone.0160292

**Published:** 2016-07-28

**Authors:** Scott I. Kavanaugh, Pei-San Tsai

**Affiliations:** Department of Integrative Physiology and Center for Neuroscience, University of Colorado, Boulder, Colorado, United States of America; University of Würzburg, GERMANY

## Abstract

A gonadotropin-releasing hormone (GnRH)-like molecule was previously identified in a gastropod, *Aplysia californica*, and named ap-GnRH. In this study, we cloned the full-length cDNA of a putative ap-GnRH receptor (ap-GnRHR) and functionally authenticated this receptor as a bona fide ap-GnRHR. This receptor contains two potential translation start sites, each accompanied by a Kozak sequence, suggesting the translation of a long and a short form of the receptor is possible. The putative ap-GnRHR maintains the conserved structural motifs of GnRHR-like receptors and shares 45% sequence identity with the octopus GnRHR. The expression of the putative ap-GnRHR short form is ubiquitous in all tissues examined, whereas the long form is only expressed in parts of the central nervous system, osphradium, small hermaphroditic duct, and ovotestis. The cDNA encoding the long or the short receptor was transfected into the *Drosophila* S2 cell line and subject to a radioreceptor assay using ^125^I-labeled ap-GnRH as the radioligand. Further, the transfected cells were treated with various concentrations of ap-GnRH and measured for the accumulation of cAMP and inositol monophosphate (IP1). Radioreceptor assay revealed that only the long receptor bound specifically to the radioligand. Further, only the long receptor responded to ap-GnRH with an increased accumulation of IP1, but not cAMP. Our studies show that despite the more prevalent expression of the short receptor, only the long receptor is the functional ap-GnRHR. Importantly, this is only the second report on the authentication of a protostome GnRHR, and based on the function and the phylogenetic grouping of ap-GnRHR, we suggest that this receptor is more similar to protostome corazonin receptors than chordate GnRHRs.

## Introduction

Gonadotropin-releasing hormone (GnRH) is a member of an ancient peptide superfamily whose origin predated the protostome-deuterostome split [1, 2]. In vertebrates, the most prominent role of GnRH is the stimulation of reproductive maturation and fertility. Orthologs of vertebrate GnRH have recently been discovered in protostomes, but their physiological roles are not clearly defined [3–5]. Physiological studies using native GnRH in molluscan models suggest protostome forms of GnRH have become highly diversified and are multi-functional molecules responsible for a wide range of regulation including neural control [6], motor activity [6, 7], feeding [7], and reproduction [8–10]. Further, the presence of additional molecules that are orthologous to protostome GnRH, including adipokinetic hormone (AKH), corazonin (CRZ), and AKH/CRZ-related peptide (ACP), have been documented [11, 12]. These peptides share common ancestry with protostome GnRH [2, 11, 13] and are present in diverse invertebrate phyla to serve taxon-specific functions [11].

Recent data mining studies suggest that protostome GnRH, AKH, CRZ, and ACP bind to receptors that are structurally related to each other, and these receptor may be difficult to distinguish functionally based on sequence homology alone [2, 11]. For example, several protostome receptors identified through cloning or transcriptome analysis have been initially named GnRH receptor (GnRHR) based on their sequence homology to vertebrate GnRHRs, but one was later found to be an AKH receptor [14, 15] and another was found to be more likely to bind to AKH than protostome GnRH [16]. In an era of whole genome and transcriptome mining that facilitates the identification of molecules based on homology, the functional deorphanization of these receptors becomes the next essential step in conferring the biological importance of the ligand-receptor pair.

In 2008, a GnRH-like molecule was isolated from a gastropod mollusk, *Aplysia californica*, and named ap-GnRH [11, 17]. ap-GnRH was an unambiguous ortholog of octopus GnRH (oct-GnRH) [18] and was produced exclusively in the central nervous system (CNS) to modulate neural activity and motor behavior without stimulating reproduction [6, 7]. Despite the extensive functional characterization of ap-GnRH [5], the receptor for ap-GnRH (ap-GnRHR) has not been identified. The presence of a related *Aplysia* AKH (ap-AKH) [12], which may possess a receptor structurally similar to ap-GnRHR, also posed a challenge to the search for ap-GnRHR.

The goal of the present study was to isolate the full-length sequence of the putative ap-GnRHR and authenticate it as a functional GnRHR in *A*. *californica*. To date, only one protostome GnRHR, octopus GnRHR (oct-GnRHR), has been functionally authenticated [19], illustrating the paucity of such studies. Towards this goal, we first analyzed expressed sequence tags and whole genome shotgun sequences of *A*. *californica* to identify partial sequences for a putative ap-GnRHR. Based on these sequences, we cloned a full-length G-protein-coupled receptor (GPCR) and validated it as a bona fide ap-GnRHR by a radioreceptor assay and the activation of the inositol phosphate (IP) pathway. Interestingly, at least two isoforms of ap-GnRHR transcript were identified, and the ubiquitously expressed form failed to produce a functional ap-GnRHR, further highlighting the importance of receptor validation.

## Materials and Methods

### Animals

Wild-caught *A*. *californica* were purchased from Alacrity Marine Biological Services (Redondo Beach, CA) and maintained in an aquarium with recirculating artificial sea water filtered through biological and chemical filters and maintained at 18–20°C. Animals were fed a diet of Romaine lettuce daily. *A*. *californica* were anesthetized by an injection of 1/2 body mass of ice-cold isotonic MgCl_2_ before sacrifice. No institutional approval was required for the use of invertebrates such as *A*. *californica*, but the authors adhered to standards of humane treatment to minimize pain, stress, and suffering of experimental animals.

### Isolation of total RNA

Total RNA was isolated from *A*. *californica* central and peripheral tissues using the Trizol reagent (Invitrogen, Carlsbad, CA) and stored at -80°C until use.

### Cloning of ap-GnRHR

Initial *in silico* analyses of *A*. *californica* expressed sequence tags and whole genome shotgun sequences were performed to identify trochozoan GnRHRs or GnRHR-like receptors. Four partial *A*. *californica* sequences were identified based on their sequence similarity with the N-terminus of oct-GnRHR. Based on these partial sequences, we designed primers to amplify a larger segment of the target gene and subsequently isolated its 3' end using the 3' rapid amplification of cDNA ends (RACE). Once the complete 3’ was identified, primers were designed to capture the 5’ end of the sequence using 5’ RACE.

Both 5’ and 3’ RACE procedures were performed using the GeneRacer kit (Invitrogen, Grand Island, NY) according to manufacturer’s instructions. Abdominal ganglia total RNA was used to generate the cDNA template for 5’ and 3’ RACE. The 3’ RACE was accomplished using AcGnRHR 13F (5’-CCC TCT ACT TGT CAA CGT ACA-3’) as the gene-specific forward primer and GeneRacer 3’ Nested Primer (5’-CGC TAC GTA ACG GCA TGA CAG TG-3’) as the reverse primer. The 5’ RACE utilized GeneRacer 5’ Nested Primer (5’-GGA CAC TGA CAT GGA CTG AAG GAG TA-3’) as the forward primer and AcGnRHR 21R (5’-TTC TTC TCA TGG GAT CAA GAA TTG-3’) as the gene-specific reverse primer. Amplification was performed using 2.5 units of Platinum Pfx DNA Polymerase (Invitrogen), 1X Pfx amplification buffer, 0.3 mM dNTP, 1 mM MgSO_4_, and varying amounts of the cDNA template in 50 μl reactions. Cycling conditions included initial melting at 95°C for 1 min, 35 cycles of amplification (45 s at 95°C, 30 s at 55°C, 1 min at 72°C), followed by a 10-min final extension at 72°C. The reactions were then A-tailed by incubating the PCR products with 10 μl 1 mM dATP and 2.5 units BioReady rTaq DNA Polymerase (BullDog Bio, Portsmouth, NH) at 72°C for 20 minutes. Resulting amplicons were cloned into pGEM T-Easy (Promega, Madison, WI) and sequenced at Sequetech Corporation (Mountain View, CA). The overlapping sequences were combined and used to design primers that encompass the entire open-reading frame (ORF). Because our initial analysis suggested this receptor might have two translational start sites, we designed primers to amplify both the long and the short receptor isoforms for *in vitro* functional studies. For this, the abdominal ganglia cDNA was amplified with the forward primers AcGnRHR Full F1 (5’-CAG GAT TCA GTC CCA TGG ACG GAA CAG-3’) (for the long receptor) and AcGnRHR Full F2 (5’-CGA CGG CTC CAA ACA TGA TCT CCA AC-3’) (for the short receptor) in conjunction with the reverse primer AcGnRHR Full R2 (5’-GGA GGG GTA TTT TTA GAT GAA AGG AGG-3’). Amplification conditions were identical to the RACE PCR described above. Resulting amplicons were cloned into pGEM T-Easy and verified by sequencing.

### Reverse-transcription polymerase chain reaction (RT-PCR) analysis of ap-GnRHR expression

Total RNA from bag cell neurons, abdominal ganglia, cerebral ganglia, pedal/pleural ganglia, buccal ganglia, tail, osphradium, small hermaphroditic duct, heart and ovotestis was harvested from individual animals, pre-treated with RQ1 DNase, reverse transcribed using Superscript III first-strand cDNA synthesis kit, and amplified with one of the following two sets of gene-specific primers: (1) AcGnRHR Full F1 as the forward primer and AcGnRHR 12R (5’-GTA CTT GAC GAA CTT GCA CAT GAC-3’) as the reverse primer, and (2) AcGnRHR 12F (5’-CAA CCA CGA GTC TAA TAA AGA CGA-3’) as the forward primer and AcGnRHR 12R as the reverse primer. AcGnRHR Full F1 encompassed the first start codon of the putative ap-GnRHR, whereas AcGnRHR 12F targeted transmembrane domain (TM) 1, and AcGnRHR 12R targeted the end of extracellular loop (EL) 2 and beginning of TM 3. The first set of primers should amplify only the long receptor isoform, whereas the second set of primers would amplify both the long and the short isoforms. Amplification was performed in 25 μl-reactions using BioReady rTaq DNA Polymerase. As a control to ensure the quality of RT, *A californica* actin (ap-Actin) was amplified for all tissues examined using gene-specific primers as described previously [12, 17]. The RT-PCR was repeated 3 times to ensure consistency.

### Phylogenetic analysis

The alignment used to generate the maximum likelihood tree consisted of 334 amino acid sequences retrieved from GenBank and the deduced ap-GnRHR amino acid sequence (GenBank accession # AHE78444.1). The alignment was produced using ClustalW in MEGA6 [20], analyzed, and the tree constructed using maximum likelihood method based on the JTT matrix-based model [21] with 1,000 bootstrap replicates.

### Construct development, cell culture, and transfection

The ap-GnRHR ORFs previously subcloned into pGEM T-Easy vector were used as PCR templates to generate the coding regions of both receptor isoforms for *in vitro* receptor expression. For this, PCR was performed using Platinum Pfx DNA Polymerase with the following primers linked to selective restriction sites: AcSal1ResR (5’-GGT ATT TGT CGA CTT AGA TGA-3’) combined with AcGnRHR-L-XbalF (5’-CAG GAT CTA CAC CCA TGG ACG G-3’) to generate the long receptor, or AcSal1ResR combined with AcGnRHR-S-XbalF (5’- GAC GGC TCT AGA CAT GAT CTC C-3’) to generate the short receptor. The PCR conditions were identical to RACE. Resulting amplicons were subcloned into pGEM T-Easy, and upon verification by sequencing, digested with XbaI and SalI and subcloned into the pAWG *Drosophila* expression vector, which expressed green fluorescent protein (GFP) as a marker (Drosophila Genomics Resource Center, Bloomington, IN). pAWG plasmid containing the long or the short form of the receptor was named pAWG-L or pAWG-S, respectively.

*Drosophila melanogaster* S2 cells (Drosophila Genomics Resource Center, Stock #6) were cultured in Schneider's Insect Medium (Sigma, Milwaukee, WI) containing 10% charcoal-stripped fetal bovine serum (FBS) (BioWorld, Dublin, Ohio) and propagated according to the supplier’s instructions. For the transfection experiment, S2 cells were seeded in 24 well plates at the density of 10^5^ cells/well with 1 ml culture medium and incubated overnight. On Day 2, the culture medium was removed, and the attached cells were incubated with a transfection solution (0.12M CaCl_2_, 25mM HEPES, 0.75mM Na_2_HPO_4_, 140 mM NaCl, pH 7.1) containing 0.5 μg/ml pAWG, pAWG-L, or pAWG-S for 17 h before replacing the transfection solution with the regular culture medium. All functional studies were performed 4-days after transfection, a time point of optimal GFP signal.

### Radioreceptor assay

Competitive binding properties of ap-GnRH and ap-AKH to the long and short isoforms of ap-GnRHR were examined using S2 cells transfected with pAWG-L or pAWG-S. Synthetic ap-GnRH (pQNYHFSNGWYA-amide; GenScript, Piscataway, NJ) was radioiodinated and purified as described previously [6, 7]. Transfected S2 cells were treated with 10 nM [^125^I]-ap-GnRH without or with increasing concentrations (10^−12^ M to 10^−6^ M) of unlabeled ap-GnRH or ap-AKH (pQIHFSPDWGT-NH_2_; GenScript) in an assay buffer (137 mM NaCl, 2.7 mM KCl, 10 mM Na_2_HPO_4_, 2 mM KH_2_PO_4_, pH 7.4). The reaction (200 μl total volume) was incubated for 3.5 h on ice in a 4°C refrigerator. Following, cells were gently washed with the assay buffer, collected in a solubilizing solution of 0.5M NaOH /1% SDS, and counted on a Packard Cobra II gamma counter. All samples were run in triplicates in 3 independent experiments.

### Inositol 1-phosphate and cAMP assays

The accumulation of inositol 1-phosphate (IP1) was measured by the IP-One ELISA (Cisbio, Bedford, MA) according to the manufacturer’s instructions. S2 cells transfected as before with pAWG, pAWG-L or pAWG-S were treated without or with 10^−12^ M to 10^−6^ M of Ap-GnRH in a stimulation buffer provided by the kit for 3 h at 28°C. All treatments were performed in triplicates in 3 independent experiments. Proctolin (RYLPT; GenScript) was used at 10^−6^ M as a positive control for the assay. Proctolin receptor is endogenously expressed in S2 cells and its activation coupled to the activation of the IP pathway [22].

The accumulation of cAMP was measured by the Cyclic AMP Competitive ELISA Kit (ThermoFisher Scientific, Waltham, MA) using the acetylated protocol according to the manufacturer’s instructions. Transfected S2 cells were treated without or with 10^−12^ M to 10^−6^ M of Ap-GnRH for 1 hr at 28°C. All treatments were performed in triplicates in 3 independent experiments. Forskolin (5 μM) was used to show maximum stimulation, and 3-Isobutyl-1-methylxanthine (1 mM) was used to prevent cAMP degradation.

### Data analysis

When suitable, data were plotted and EC_50_ calculated using four-parameters logistics-based non-linear regression curve fit feature in the GraphPad Prism5 software (GraphPad, San Diego, CA). Linear regression was used in most cases when a dose effect was not observed.

## Results

**Fig 1:**
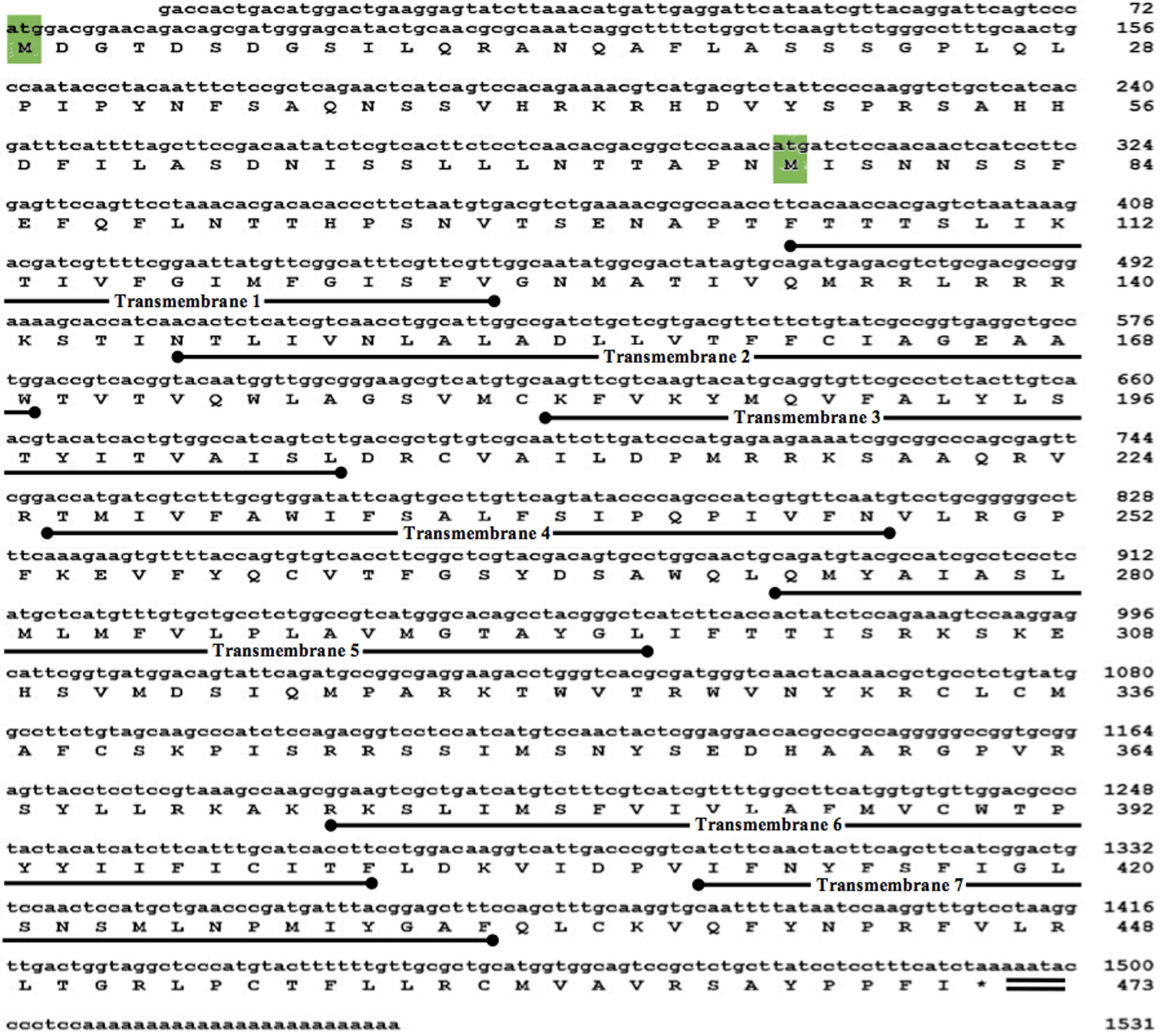
Nucleotide and deduced amino acid sequences of the full-length putative ap-GnRHR (Genbank Accession # AHE78444.1). The nucleotides (lower case letters) and amino acids (upper case letters) are numbered accordingly. The seven predicted transmembrane domains are underlined. The two putative translation start sites are highlighted in green, and the asterisk (*) denotes the stop codon. The nucleotides corresponding to the truncated polyadenylation signal (aata) are double-underlined.

The full-length ap-GnRHR spans 1531 bp and contains two potential translation start sites, each accompanied by a Kozak consensus sequence [23] (Fig 1). The first start codon generates a 1419-bp ORF encoding a longer 473-amino acid GPCR (ap-GnRHR-L). The second start codon generates a 1191-bp ORF encoding a shorter 397-amino acid GPCR (ap-GnRHR-S). A truncated polyadenylation signal AATA [24] is located 7 bases upstream of the poly (A) tail. The general structural framework predicts this receptor as a Class A GPCR.

**Fig 2:**
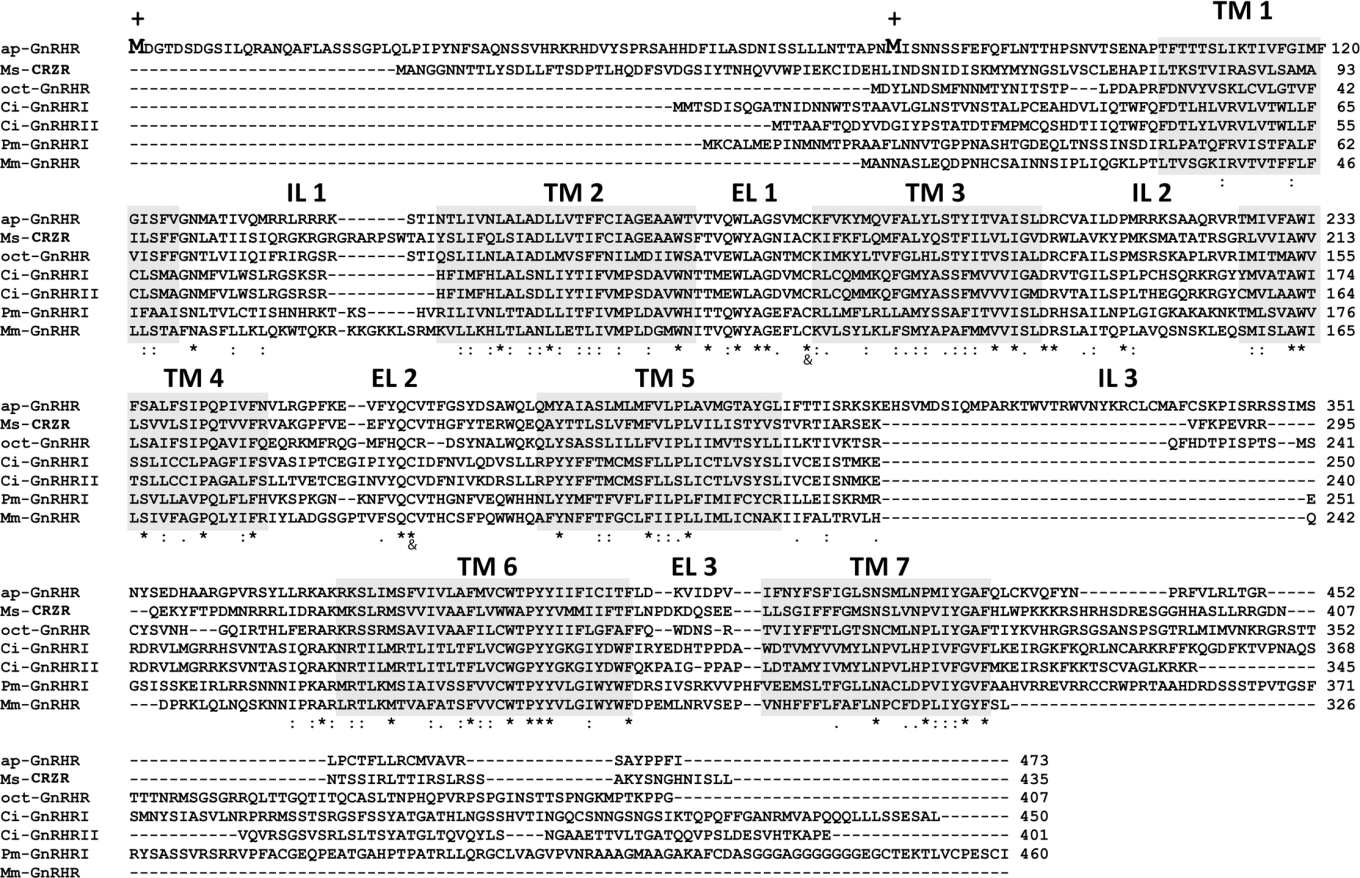
Amino acid sequence alignment of ap-GnRHR with representative GnRHR-related receptors. Shaded amino acids indicate the transmembrane (TM) domains. The predicted intracellular loops (IL) and extracellular loops (EL) are numbered accordingly. The two putative start sites (M) are each denoted by a “+”. Ms-CRZR—corazonin receptor, *Manduca sexta* (AAR14318); Oct-GnRHR—Gonadotropin-releasing hormone receptor, *Octopus vulgaris* (GNRHR_OCTVU); Ci-GnRHRI—gonadotropin-releasing hormone receptor 1, *Ciona intestinalis* (NP_001028997); Ci-GnRHRII—gonadotropin-releasing hormone receptor 2, *Ciona intestinalis* (NP_001028996); Pm-GnRHRI—gonadotropin releasing-hormone receptor, *Petromyzon marinus* (AF439802_1); Mm-GnRH—gonadotropin-releasing hormone receptor isoform *Mus musculus* (NP_001297580). Identical, highly conserved, and less conserved amino acid residues are denoted by an asterisk (*), a colon (:), and a period (.), respectively. Ampersand (&) denotes cysteine residues involved in the disulfide bridge formation.

The deduced amino acid sequence of ap-GnRHR-L was compared with the related GPCRs from representative taxa (Fig 2). Comparison of the entire receptors, including the N-terminus, revealed that this receptor exhibited 40% identity with *Manduca sexta* (tobacco hornworm) CRZ receptor (Ms-CRZR), 45% with *Octopus vulgaris* (common octopus) GnRHR (oct-GnRHR), 27% with *Ciona intestinalis* (sea squirt) GnRHRI (Ci-GnRHRI), 26% with Ci-GnRHRII, 31% with *Petromyzon marinus* (sea lamprey) GnRHR (Pm-GnRHR) and 28% with *Mus musculus* (house mouse) GnRHR (Mm-GnRHR). The cloned receptor has 8 potential N-glycosylation sites (N-X-^S^/_T_) at positions 33, 38, 64, 71, 81, 90, 96, and 352, with the first 7 sites clustered in the extracellular N-terminus. The receptor also has 6 potential protein kinase A phosphorylation sites at positions 216, 223, 307, 341, 345, and 372, and 4 potential protein kinase C phosphorylation sites at positions 50, 303, 344, and 450. As with most GnRH receptors, a conserved DRXXX^I^/_V_ motif was seen in the region connecting TM 3 and intracellular loop (IL) 2. Two cysteine residues for the formation of a highly conserved disulfide bridge between EL 1 and EL 2 [25] are located at positions 182 and 260.

Interestingly, ap-GnRHR-L has the longest extracellular N-terminus and IL 3 compared to other similar GPCRs (Fig 2). The TM regions are well conserved between ap-GnRHR and other invertebrate receptors, but the presence of the long IL 3 in ap-GnRHR greatly diminished its similarity to others. The length of extracellular N-terminus in another molluscan GnRHR (oct-GnRHR) is more similar to that of ap-GnRHR-S than ap-GnRHR-L. Like all type II vertebrate GnRHRs [25] and other invertebrate receptors in this superfamily, ap-GnRHR also possesses a C-terminal tail.

**Fig 3:**
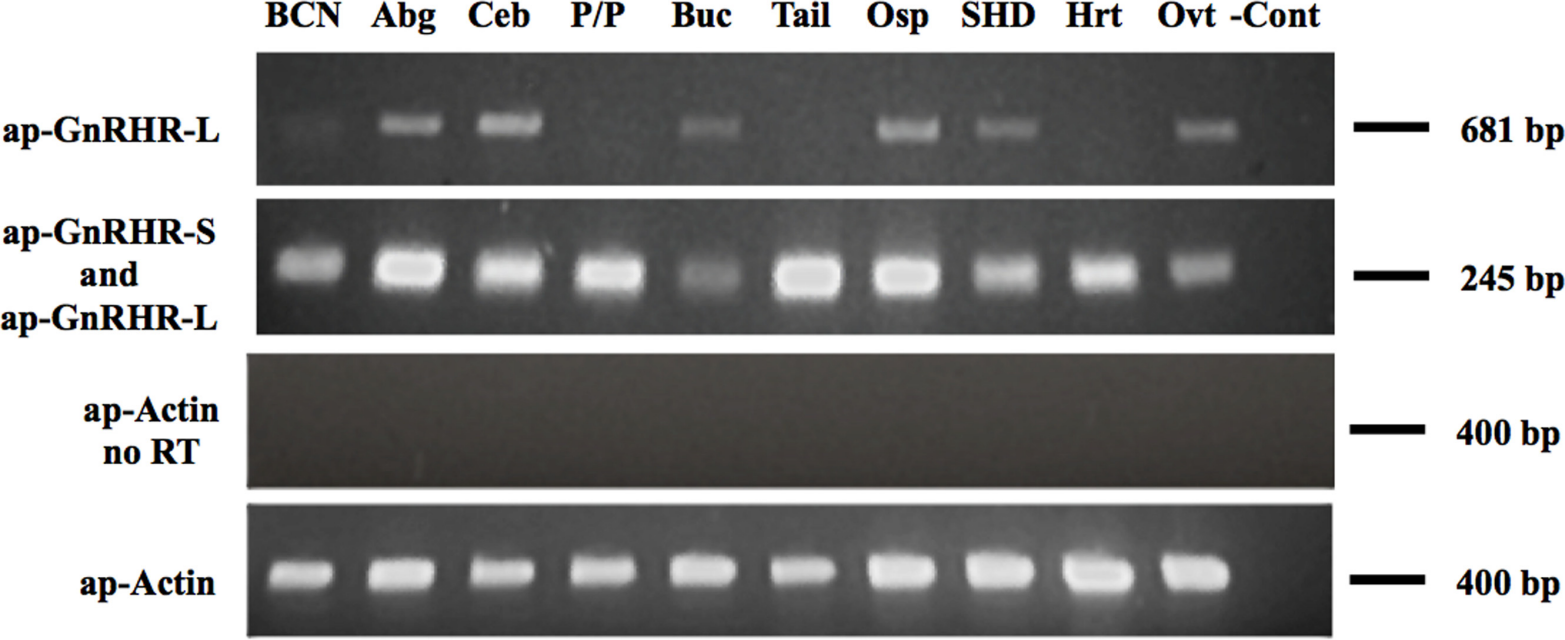
Expression of ap-GnRHR isoforms (top two panels) in different *A*. *californica* tissues examined by RT-PCR. Primers used for the top panel amplify only ap-GnRHR-L; primers used for the second panel amplify both ap-GnRHR-L and ap-GnRHR-S. Negative controls (no RT) were RNA samples that have not been reverse transcribed (third panel). ap-Actin was used as a control to ensure the quality of RNA samples (bottom panel). The sizes of the PCR products are shown on the right. BCN, bag cell neurons; Abg, abdominal ganglia; Ceb, cerebral ganglia; P/P, pedal/pleural ganglia; Buc, buccal ganglia; Tail, ventral tail tissue; Osp, osphradium; SHD, small hermaphroditic duct; Hrt, heart; Ovt, ovotestis;—Cont, negative water control.

RT-PCR results suggest ap-GnRHR-L and ap-GnRHR-S are differentially expressed (Fig 3 and S1 Fig). Since ap-GnRHR-S is part of ap-GnRHR-L, it is not possible to amplify only the short receptor without simultaneously detecting the long form. However, primers targeting specifically the long receptor revealed that ap-GnRHR-L was only expressed in the abdominal, cerebral, and buccal ganglia of the central nervous system and a few peripheral tissues including the osphradium (a chemosensory organ), small hermaphroditic duct, and ovotestis (Fig 3 and S1 Fig). Primers targeting both receptor isoforms successfully amplified a transcript in all tissues examined (Fig 3 and S1 Fig). By deduction, our results suggest the expression pattern is ubiquitous for ap-GnRHR-S but restricted for ap-GnRHR-L.

**Fig 4:**
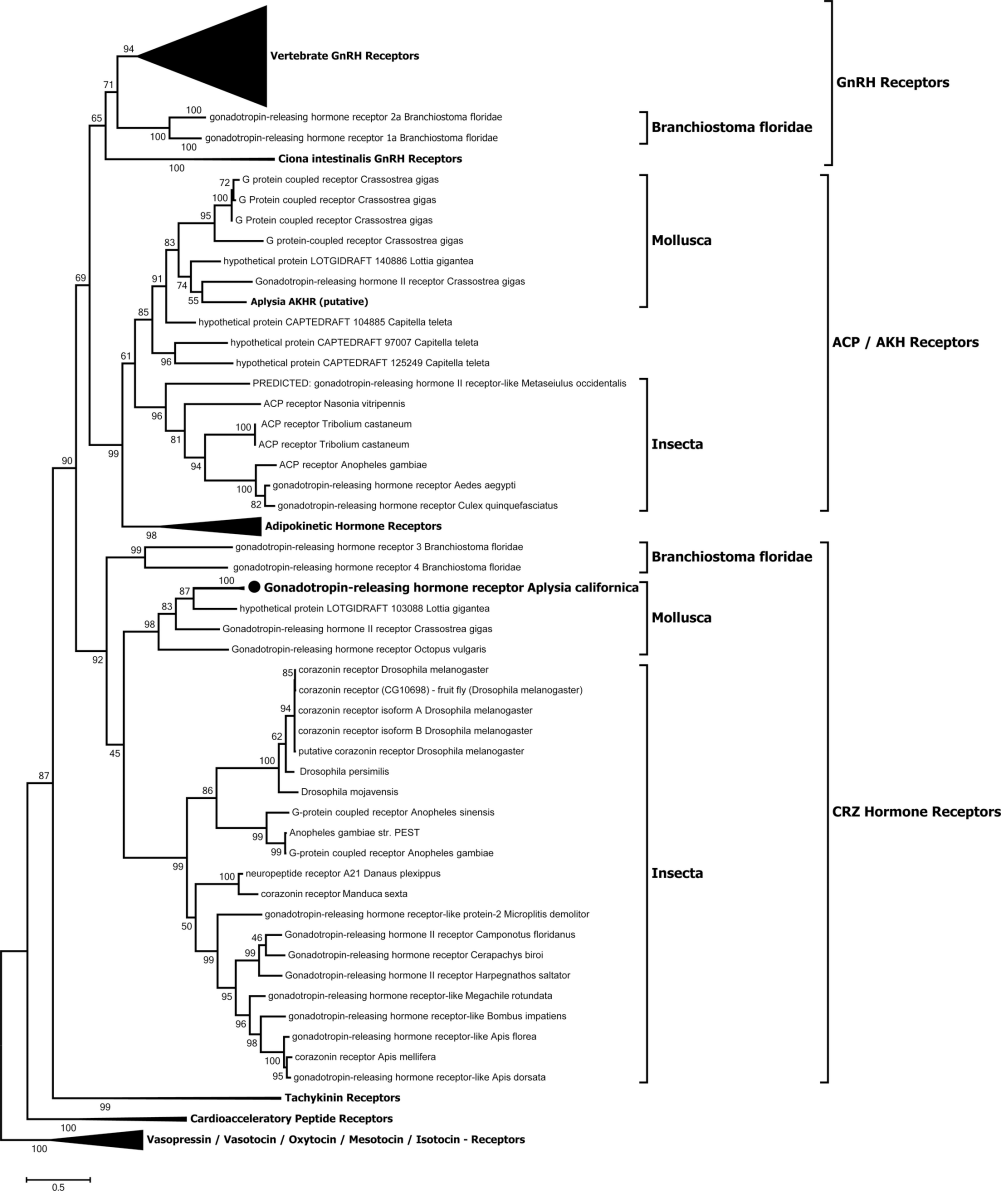
Maximum likelihood analysis of the phylogeny of GnRHR-related receptors. The bootstrap values (in %) from 1000 replicas are indicated at each branch point. The tree is drawn to scale, with branch lengths measured in the number of substitutions per site. The analysis involved 334 amino acid sequences and a total of 1171 positions in the final dataset. The invertebrate cardioacceleratory peptide receptors and vertebrate vasopressin receptor-like GPCRs were used as outgroups to root the tree. The detailed nomenclature and sequences are listed as data in S1 Dataset.

Phylogenetic analysis of 334 predicted amino acid sequences of GnRHR and related receptors (Fig 4) suggests this receptor superfamily is segregated into 3 major clades: (1) deuterostome GnRHR, (2) ACP/AKH receptors (ACPR/AKHR), and (3) CRZ receptors (CRZR). Ap-GnRHR is clustered with oct-GnRHR and several unauthenticated receptors from a limpet (*Lottia gigante*a) and an oyster (*Crossostrea gigas*). These molluscan receptors cluster with multiple insect receptors, including the authenticated fruit fly CRZR [26], to form a CRZR lineage. On the other hand, the chordate GnRHRs form a distinct group that is segregated from all other protostome receptors examined. Interestingly, a putative AKHR was identified from *A*. *californica* genome and belonged in the ACPR/AKHR lineage with other authenticated insect ACPRs and AKHRs (Fig 4).

**Fig 5:**
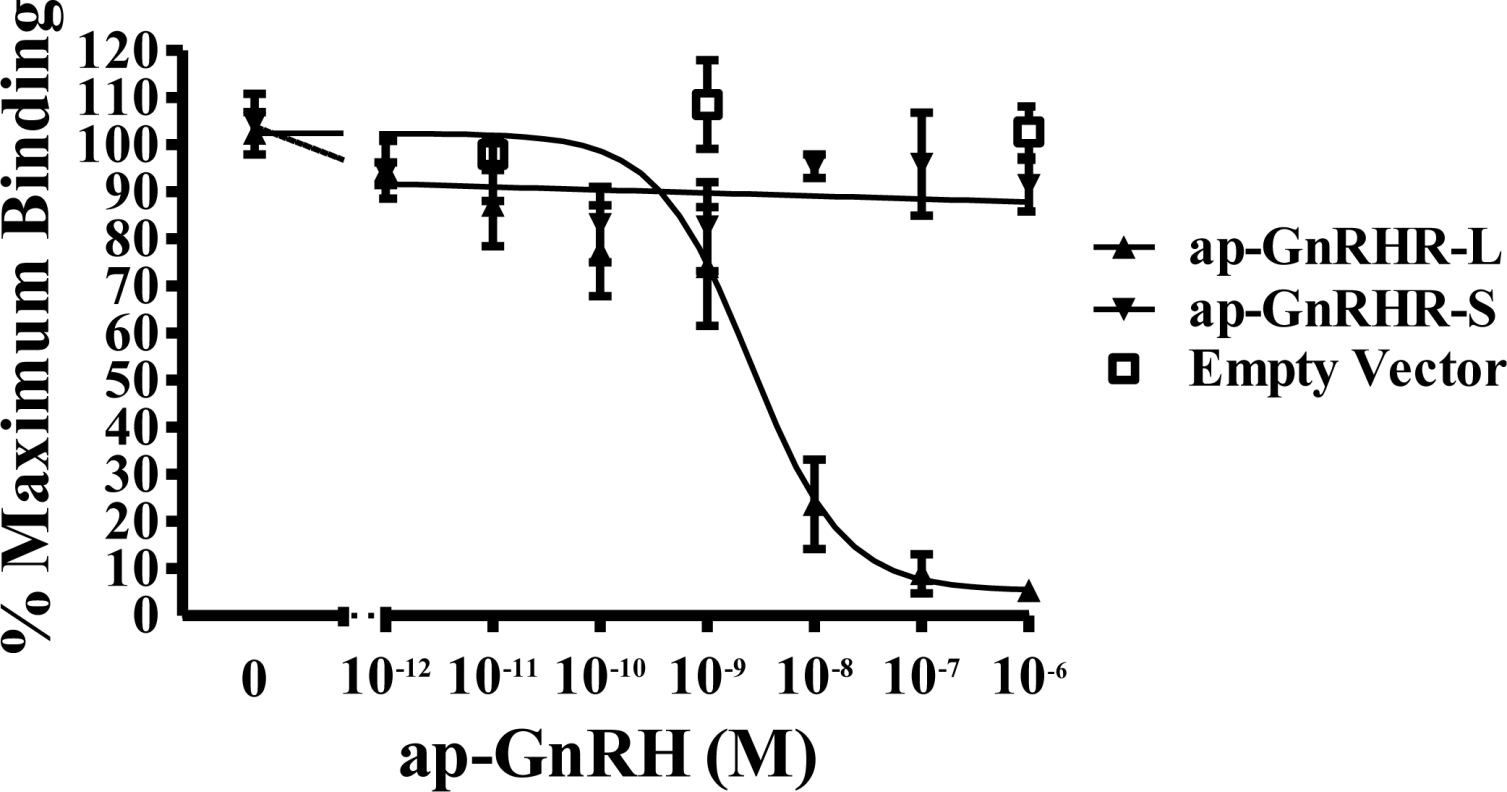
Analysis of [^125^I]-ap-GnRH binding to S2 cells transfected with ap-GnRHR-L or ap-GnRHR-S. Binding to the radioligand was displaced by increasing concentrations of unlabeled ap-GnRH in S2 cells transfected with ap-GnRHR-L (EC_50_ = 2.52 nM), but not ap-GnRHR-S. Binding data are expressed as the percentage of [^125^I]-ap-GnRH binding to the respective receptor in the absence of unlabeled hormone. Empty vector-transfected cells were used as a negative control. Each data point represents N = 3 assays run in triplicates.

**Fig 6:**
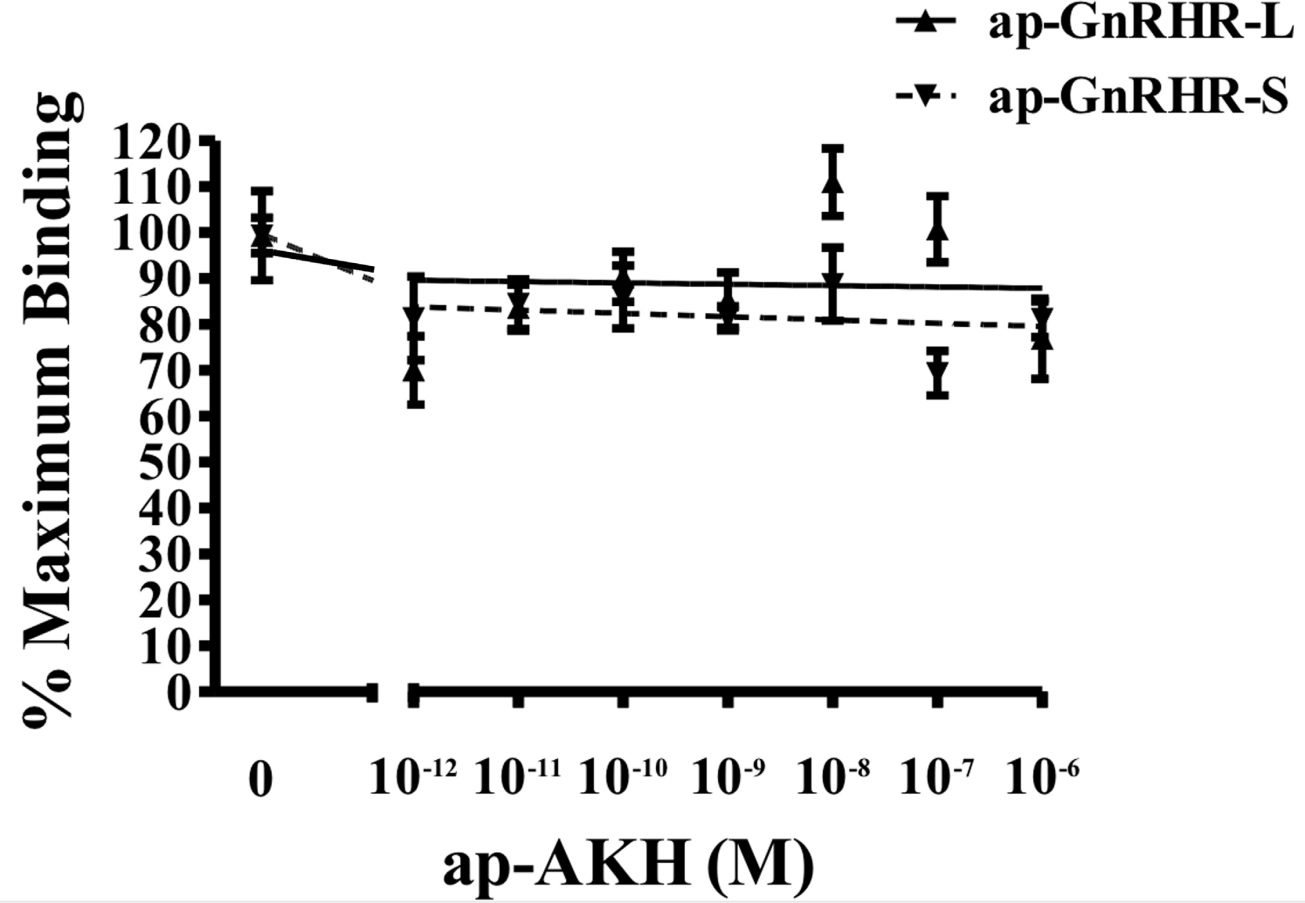
Analysis of [^125-^I]-ap-GnRH binding to S2 cells transfected with ap-GnRHR-L or ap-GnRHR-S. Binding to the radioligand was not displaced by increasing concentrations of unlabeled ap-AKH in S2 cells transfected with either ap-GnRHR-L or ap-GnRHR-S. Binding data are expressed as the percentage of [^125^I]-ap-GnRH binding to the respective receptor in the absence of unlabeled hormone. Each data point represents N = 3 assays run in triplicates.

**Fig 7:**
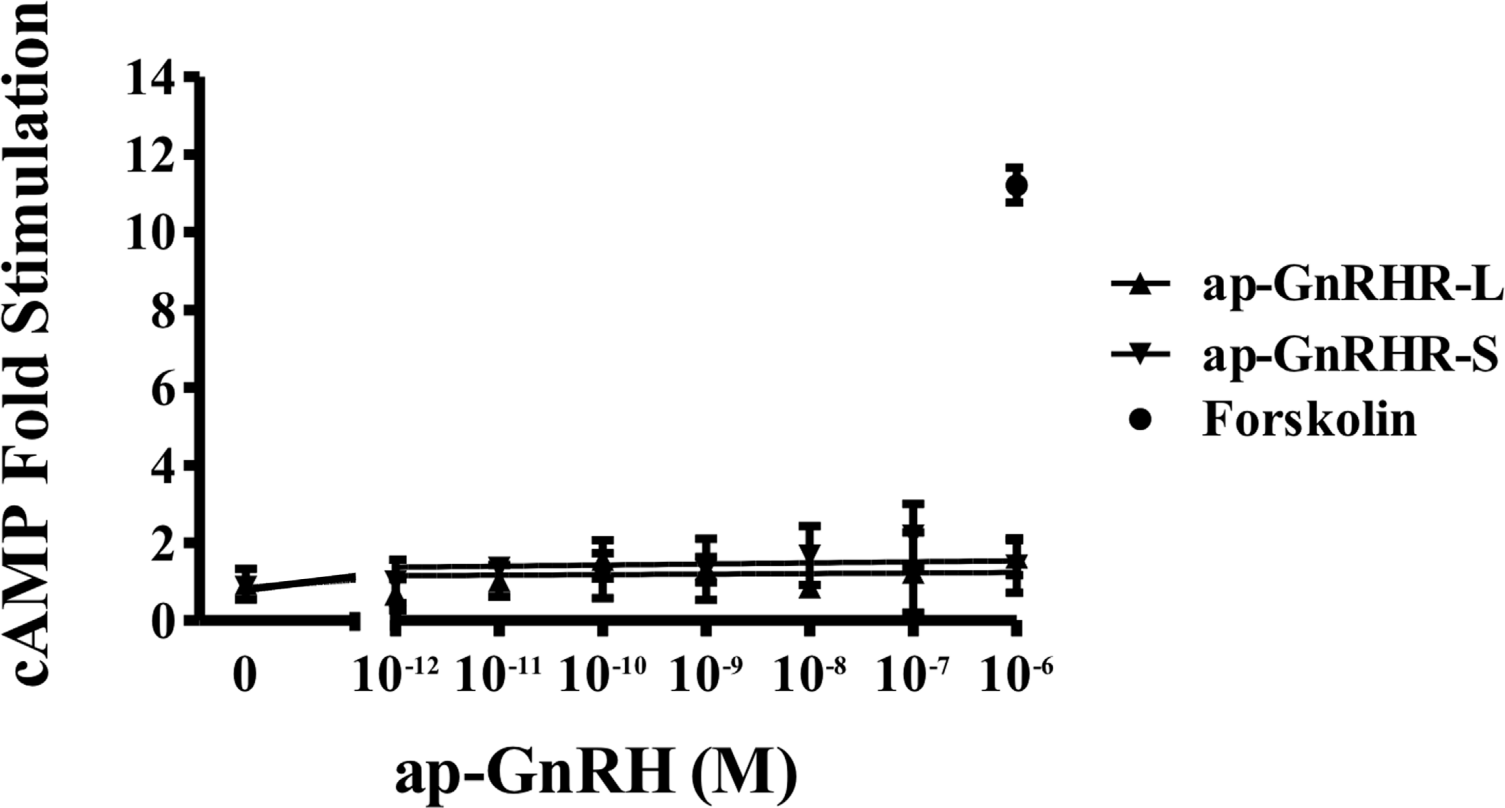
ap-GnRH did not stimulate cAMP accumulation in S2 cells transfected with either ap-GnRHR-L or ap-GnRHR-S. Data are expressed as fold stimulation over cAMP levels in the untreated cells. Forskolin was used as a positive control. N = 3 assays run in triplicates.

**Fig 8:**
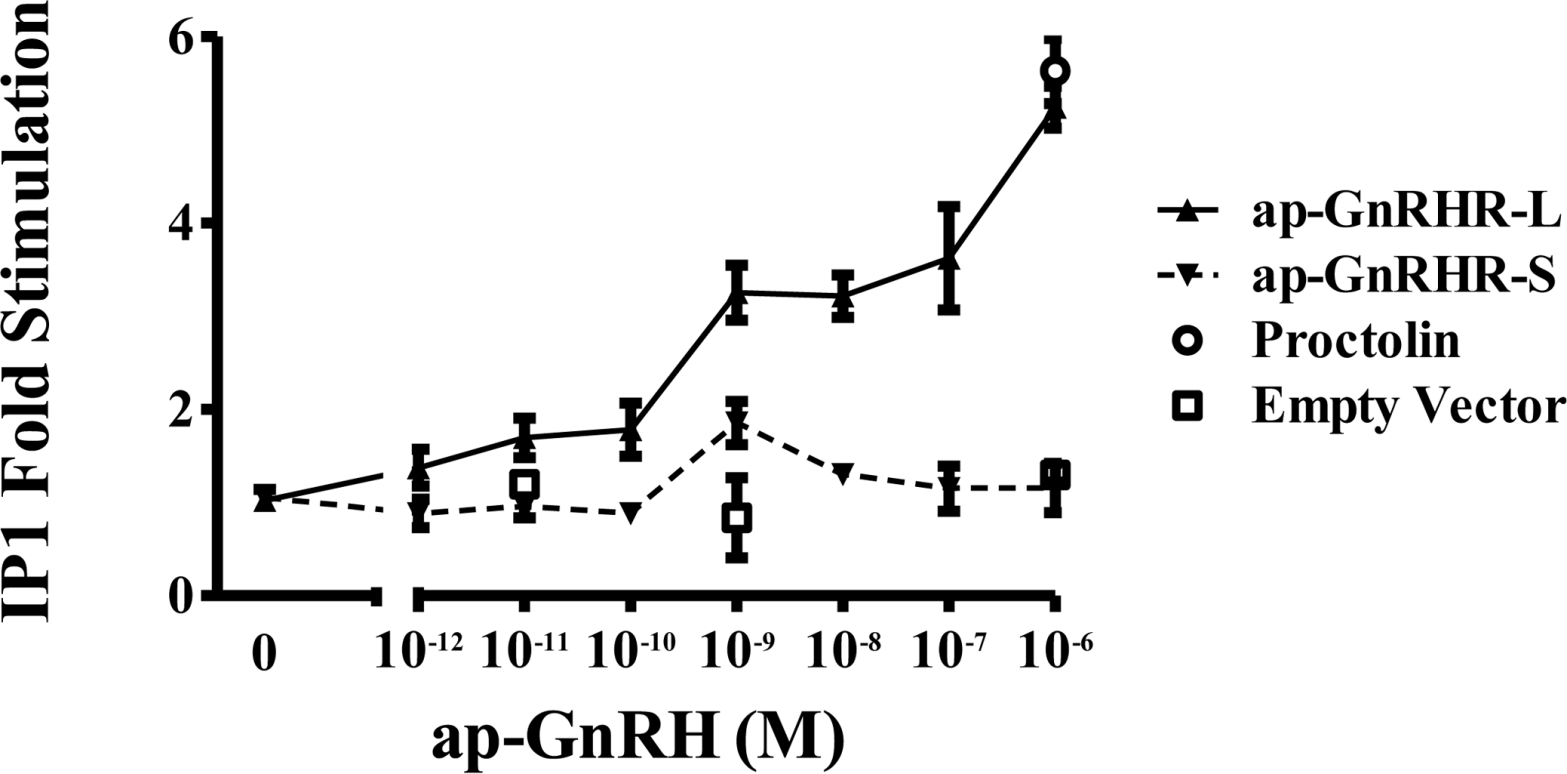
ap-GnRH stimulated IP1 accumulation in S2 cells transfected with ap-GnRHR-L, but not ap-GnRHR-S. Data are expressed as fold stimulation over IP1 levels in the untreated cells. Proctolin was used as a positive control in ap-GnRHR-L or ap-GnRHR-S-transfected cells, and empty vector-transfected cells were used as a negative control. N = 3 assays run in triplicates.

Radioreceptor assays of S2 cells transfected with ap-GnRHR-L or ap-GnRHR-S show that [^125^I]-ap-GnRH binds specifically to ap-GnRHR-L, with unlabeled ap-GnRH displacing radioligand binding at EC_50_ of 2.52 nM (Fig 5). No specific binding was observed in S2 cells transfected with ap-GnRH-S (Fig 5). Further, binding of [^125^I]-ap-GnRH to either ap-GnRHR-L or ap-GnRHR-S could not be displaced by a related peptide, ap-AKH (Fig 6). ap-GnRH did not stimulate cAMP accumulation in either ap-GnRHR isoform (Fig 7), but increased IP1 accumulation up to 6-fold in S2 cells transfected with ap-GnRHR-L (Fig 8). The quality of cAMP and IP1 assays was validated by the positive effects of forskolin and proctolin (Figs 7 and 8), respectively. S2 cells transfected with the empty vector, pAWG, failed to bind specifically to [^125^I]-ap-GnRH and did not respond to ap-GnRH in terms of cAMP or IP1 accumulation (Figs 5 and 8).

## Discussion

The rapidly emerging field of protostome GnRH has provided valuable insight into the functional and structural evolution of this hormone superfamily [2, 27]. There is now strong evidence to suggest the GnRH superfamily co-evolved with its cognate receptors exclusively in the bilaterian lineages, with both the ligands and receptors absent in non-bilaterians such as sponges, cnidarians, placozoans and ctenophores [1]. These findings placed the origin of the GnRH and GnRHR superfamilies around 570 to 700 million years ago [28], and this evolutionary timeline allowed for the tremendous diversification of ancestral molecules into modern clades consisting of chordate GnRHs, non-chordate orthologs such as AKH, CRZ, ACP, and their cognate receptors [29]. Such diversity underscores the importance of receptor deorphanization to define the exclusive ligand-receptor pairing, which has been the primary goal of the present study.

Our results validate a candidate receptor as an authentic ap-GnRHR. This receptor has several features that are novel among most known GnRHRs. The first feature is the presence of two potential start sites that might result in the translation of two endogenous receptor proteins (Figs 1 and 2). Neither start site contains the ideal Kozak sequence (GCC^A^/_G_CCATGG) found among vertebrates [23], but both start site variations have been documented as functional in higher eukaryotes. Unlike vertebrates, protostomes appear to lack a strong preference for a G at position #4 of the Kozak sequence (last G in ATGG) [30], suggesting the two start sites in ap-GnRHR may be similarly effective. The second feature is the differential expression of at least two forms of transcript that vary in the 5’ region corresponding to the extracellular N-terminus (Fig 3 and S1 Fig). A parsimonious explanation is that two splice variants, one full-length and one with a 5’ truncation, are produced in a tissue-specific manner. This may have functional implications as discussed in a later paragraph. The third feature is the unusually long IL 3 of ap-GnRHR compared to other related GPCRs (Fig 2). The IL 3 in ap-GnRHR is approximately twice as long as oct-GnRHR and is rich in phosphorylation target residues. IL 3 in vertebrate GnRHRs plays a crucial role in the coupling to G_q/11_ [25], but the functional significance of this region in ap-GnRHR is at present unclear. Despite these unusual features, ap-GnRHR-L is a bona fide receptor that binds with high affinity to ap-GnRH to activate a well-defined signaling pathway for vertebrate GnRHRs [25].

The nomenclature of invertebrate GnRH and GnRHR superfamilies is at present problematic due to several naming attempts [15, 16, 19, 27] as well as our evolving understanding of these molecular families [2, 31]. A phylogenomic analysis of the deep relationships among GnRH-like peptides revealed a strong monophyletic grouping of all chordate GnRHs, but their relationships with protostome GnRH-like molecules such as AKH, CRZ, ACP, and protostome GnRHs are paraphyletic [29]. Our present phylogenetic analysis of GnRHR-like GPCRs mirrors findings on the ligands and further re-classifies protostome GnRHR-like receptors into the ACPR/AKHR and CRZR clades, all of which are paraphyletic to chordate GnRHRs (Fig 4). At some point, the grouping and functional characteristics of protostome GnRHRs should encourage an attempt at a more consistent nomenclature. For example, ap-GnRH is stimulatory to neither gonadotropin secretion nor gonadal function in *A*. *californica*, but instead controls behavioral and motor coordination [7]. Further, the multifunctional nature of ap-GnRH is similar to other non-GnRH peptides, such as CRZ, AKH and ACP, in this superfamily [11]. By virtue of its function and the phylogenetic grouping of its receptor, ap-GnRH should be more appropriately named ap-CRZ and its receptor named ap-CRZR.

Our functional characterization of ap-GnRHR revealed that the expression of ap-GnRHR-L, but not ap-GnRHR-S, resulted in ligand binding and the initiation of intracellular signaling (Figs 5–8). A caveat is that S2 cultures transfected with ap-GnRHR-L may also produce ap-GnRHR-S, since the short receptor sequence is included in the long receptor. The short receptor alone is non-functional, but it may play a role in modulating the signaling properties of the long receptor via receptor hetero-dimerization. Hetero-dimerization among GnRHR splice variants and paralogs is common [32–35], and in the case of tunicate GnRHRs, an orphan paralog with no binding affinity for any tunicate GnRHs can dimerize with another functional tunicate GnRHR to markedly enhance the biological potency of the latter [34, 35]. Future studies using site-directed mutagenesis to alter the second start codon of ap-GnRHR-L should allow us to examine the activation of ap-GnRHR-L in isolation.

Although vertebrate GnRHRs are predominantly coupled to G_q/11_ and activate the IP pathway, their coupling to G_s_ and G_i_ has also been reported [25]. The preferential coupling of a vertebrate GnRHR to a particular G protein may also change in response to the ligand [36]. Consistent with this notion, two tunicate GnRHRs were reported to activate exclusively the cAMP pathway, but one reported to activate both cAMP and IP pathways when stimulated with a specific tunicate GnRH, reaffirming the versatility of G protein coupling [37]. Our results support the IP pathway as the primary signaling pathway activated by ap-GnRHR and are consistent with findings from oct-GnRHR [19]. That said, cAMP accumulation should be routinely examined for other trochozoan GnRHR-related receptors since ecdysozoan CRZR and AKHR are known to activate both IP and cAMP pathways [38, 39]. In addition, we noted that ap-AKH, an ortholog of ap-GnRH and ecdysozoan AKH [12], failed to bind to ap-GnRHR-L or ap-GnRHR-S (Fig 5), demonstrating a high level of receptor selectivity for specific protostome ligands. In contrast to this selectivity, an amphioxus GnRHR with structural similarity to protostome GnRHR was more promiscuous and responded similarly to amphioxus GnRH, oct-GnRH and an insect AKH [40, 41]. Based on these results, we suggest a high degree of fidelity in the ligand-receptor pairing within the protostome members of the GnRH/GnRHR superfamilies that may have resulted from long periods of ligand-receptor co-evolution.

The expression pattern of a receptor is frequently used to infer its function, but our results suggest a caveat to this approach. In our case, the non-functional ap-GnRHR-S is ubiquitously expressed in all tissues examined, an outcome consistent with our earlier work [5], but the functional ap-GnRHR-L is expressed in far fewer tissues (Fig 3). This finding carries the significant implication that some invertebrate GnRHR-like receptors may not be functional even if expressed at high levels. As such, expression studies should be regarded with caution in the absence of functional data.

The functional ap-GnRHR-L is expressed in both central and peripheral tissues, a finding consistent with previous data suggesting ap-GnRH as a neuropeptide with diverse roles in the control of neuronal, feeding, and motor activities [5–7]. Both ap-GnRHR-L and ap-GnRHR-S are weakly expressed in the ovotestis, an observation that may invite speculations on a reproductive role of this receptor. However, we have not observed any stimulatory effects of ap-GnRH on gonadal histology, egg-laying, and mating behavior in *A*. *californica* [7]. In fact, ap-GnRH reduced the ability of the neuroendocrine bag cell neurons to secrete an important reproductive hormone, thereby decreasing the probability of egg-laying in these animals [6]. The expression pattern of ap-GnRHR-L is also consistent with the functional diversity reported for ecdysozoan CRZ, AKH, and ACP in neural, behavioral, and metabolic regulation [11]. Reproductive activation by these neuropeptides in ecdysozoans is uncommon, and if observed, is likely secondary to their primary functional repertoire instead of being a dedicated and conserved effect comparable to vertebrate GnRH. Our current analyses of ap-GnRHR, when combined with the previous phylogenomic analysis [29] and physiological characterization [5–7], steer us towards the conclusion that ap-GnRHR is more similar to protostome CRZR than chordate GnRHR in structure, function, and evolutionary ancestry.

In sum, we have molecularly isolated a putative ap-GnRHR and functionally validated it as a bona fide ap-GnRHR. This receptor exhibits some unusual features, including the presence of two potential translation start sites and the differential expression of at least two transcript isoforms, but with only one form being functional. This is only the second report on the functional authentication of a trochozoan GnRHR, thus it is unclear if these features are unique to ap-GnRHR or also present in other trochozoan receptors. Additional functional pairing of protostome ligands and receptors within the GnRH and GnRHR superfamilies is needed to shed light on the histories of how these ligands and receptors co-evolved in the last 570–700 million years.

## Supporting Information

S1 Dataset A complete list of amino acid sequences and Accession numbers used to construct the evolutionary tree (Fig 4). (PDF)

S1 Fig Full images of RT-PCR (presented in Fig 3) with DNA ladder to indicate the size of the amplicons. Amplification of ap-GnRHR-L and ap-GnRHR-S (A), ap-Actin (B), and ap-Actin using RNA samples that have not been reversed transcribed (C) are shown. Tissue abbreviations are indicated in Fig 3 legend. The 12 visible DNA ladder bands are: 1517, 1200, 1000, 900, 800, 700, 600, 500, 400, 300, 200, and 100 bp (New England Biolabs, Ipswich, MA).
(TIF)
