# Supplementary material for: Functional Authentication of a Novel Gastropod Gonadotropin-Releasing Hormone Receptor Reveals Unusual Features and Evolutionary Insight
Source: PLoS One. 2016 Jul 28;11(7):e0160292. doi: 10.1371/journal.pone.0160292 (PMC4964986; doi:10.1371/journal.pone.0160292)
Supplement: S1 Dataset — (PDF) [file pone.0160292.s001.pdf]

#gi|108514727|gb|ABF93210.1|_GnRH_receptor_type_1_Epinephelus_coioides
MSGNWSPSPNTSQFPPLTDWEAPTFTRAAQFRVGATFILFLFTACSNLALFASVWCGRGR
RLASHLRPLMLSLASADLMMTFVVMPLDAVWNVTVQWYGGDVLCKLLCFLKLFAMHASAF
ILVVISLDRQHAILHPLDALNAHRRNRRMLLLAWSLSLVLASPQLFIFRTIRVEAVDFTQ
CATHGSFSHRWQETVYNMFHFITLYVVPLLVMSCCYSRILLHIHLQHLRDKAGESYLRRS
GTDIIPKARLKTLKMTVVIVLSFVVCWTPYYLLGIWYWFQPDMLRVTPEYVHHALFLFGN
LNTCCDPIIYGFYTPSFRADLAACCRRTKSDASLRSPDRLSARQDPHSAEHEADPTTNNQ
PAGD

#gi|109074666|ref|XP_001109227.1|_PREDICTED:_gonadotropin-
releasing_hormone_receptor_isoform_2_Macaca_mulatta
MANSASPEQNQNHCSVINNSIPLMQGNLPTLTLSGKIRVTVTFFLFLLSATFNSSFLLKL
QKWTQKKEKGKKLSRMKLLLKHLTLANLLETLIVMPLDGMWNITVQWYAGEFLCKVLSYL
KLFSMYAPAFMMVVISLDRSLAITRPLALKSSSKLGQSMVGLAWILSSVFAGPQLYIFRM
IHLADSSGQTKVFSQCVTHCSFPQWWHQAFYNFFTFSCLFIIPLLIMLICNAKIIFTLTR
VLHQDPHKLQLNQSKNNIPRARLKTLKMTVAFATSFTVCWTPYYVLGIWYWFDPEMLNRV
SDPVNHFFFLFAFLNPCFDPLIYGYFSL

#gi|11132714|sp|Q90334.1|ITR_CATCO_RecName:_FullIsotocin_receptor_ShortITR
MEEMFKEQDFWSFNESSRNSTVGNETFGGNQTVNPLKRNEEVAKVEVTVLALVLFLALAG
NLCVLIAIYTAKHTQSRMYYLMKHLSIADLVVAVFQVLPQLIWDITFRFYGPDFLCRLVK
YLQTVGMFASTYMLVLMSIDRCIAICQPLRSLHKRKDRCYVIVSWALSLVFSVPQVYIFS
LREIGNGVYDCWGDFVQPWGAKAYITWISLTIYIIPVAILGGCYGLISFKIWQNFKRKTK
KDQCITLTTAASKANALARVSSVKLVSKAKITTVKMTFVIVLAYIVCWTPFFFVQMWSAW
DPEAPREAMPFIISMLLASLNSCCNPWIYMFFAGHLFHDLKQSLLCCSTLYLKSSQCRCD
QEHDSRKSNCSTYVIKSTSSQRSITQSSIT

#gi|11133329|sp|Q90252.1|MTR_BUFMA_RecName:_FullMesotocin_receptor_ShortMTR
MEGLCLNLDCSELPNSSWVNSSMENQNHSSNSTRDPLKRNEEVAKVEVTVLALILFLALA
GNICVLLGIYINRHKHSRMYFFMKHLSIADLVVAIFQVLPQLIWDITFRFYAPDLVCRLV
TYLQVVGMFASTYMLLLMSLDRCLAICQPLRSLHRRSDCVYVLFTWILSFLLSTPQTVIF
SLTEVGNGVYDCRADFIQPWGPKAYITWITLAVYIIPVMILSVCYGLISYKIWQNIRLKT
VCESNLRLSTSRRATLSRVSSVRLISKAKIRTVKMTFIIVLAYIVCWTPFFFVQMWSVWD
PNPPKEASLFIIAMLLGSLNSCCNPWIYMLFTGHLFHDLLQSFLCCSARYLKTQQQGSDL
SASRKSNSSTFVLSRKSSSQKSITQPSTA

#gi|112983116|ref|NP_001037049.1|_adipokinetic_hormone_receptor_Bombyx_mori
MDIDEKVSGPGGASQKNWSHLLHVNNTYDELPLEMRFNYSHMVSMTVYSVLMVISATGNL
TVLYQLVRRRRAKRASRLDILLMHLAVADLMVTFLMMPLEIAWAGTVQWFAGDLMCRVMM
FTRTFGLYLSSFVLICIAVDRYYAILKPLNVTWEATVRRAIIVAWVCAGLASLPQSFIFH
VEEHPEVKGYNQCVSYGSLPTEKHEFAYFLVNMILMYVIPLVSTLYCSCAALFEIIRRAN
TANDKMRRSGIGLLGRARARTLKMTVTIVLVFFTCWSPYYCYCLWYWIDKESIKNLDPAL
QKAMWLFSCTNSCANPIVYGVFNRNRWNWRAGKFQNGRCRSGSGRKGSRLPHGESTEISA
ATLSRARHSNGSDHNGRRDSSYANQNGPQKHWNTINNNHVTNGMV

#gi|113461951|ref|NP_001038197.1|_oxytocin_receptor_Macaca_mulatta
MEGELAANWSTEAVNSSAAPPGAEGNCTAGPPRRNEALARVEVAVLCLILFLALSGNACV
LLALRTTRHKHSRLFFFMKHLSIADLVVAVFQVLPQLLWDITFRFYGPDLLCRLVKYLQV
VGMFASTYLLLLMSLDRCLAICQPLRSLRRRTDRLAVLATWLGCLVASAPQVHIFSLREV
ADGVFDCWAVFIQPWGPKAYITWITLAVYIVPVIVLAACYGLISFKIWQNLRLKTAAAAA
AEAPEGAAAGDGGRMALARVSSVKLISKAKIRTVKMTFIIVLAFIVCWTPFFFVQMWSVW
DANAPKEASAFIIVMLLASLNSCCNPWIYMLFTGHLFHELVQRFLCCSASYLKGNRLGET
STSKKSNSSSFVLSHRSSSQRSCSQPSTA

#gi|114705478|gb|ABI75336.1|_gonadotropin-
releasing_hormone_receptor_type_2A_Odontesthes_bonariensis
MNTSLCDSAVSMYQLVAGHQANTSLNCSSAASNWTAGGDAPQLPTFTTAAKVRVIITFIL

CGISAFCNLAVLWAAHSDGKRKSHVKVLIINLTVADLLVTFIVMPVDAVWNITVQWLAGD
FACRLLMFLKLQAMYSCAFVTVVISLDRQSAILNPLAINKARMRNRVMLTVAWGMSVVFS
VPQIFLFHNVTITHPEDFTQCTTRGSFVPHWHETAYNMFTFSCLFLLPLVIMITCYTRIF
CEISKRLRKDNLPSNEVYLRCSKNNIPKARMRTLKMSIVIVLSFIICWTPYYLLGLWYWF
FPDDLEGKVSHSLTHILFIFGLFNACLDPIIYGLFTIHFRKGLQRYYSNGTAASDVDNNT
AITGSFACAANPLSGKSELRRPSQERLMLRSDNHSKAEPSFPSSSFLIADNDTERDPNQI
SPESTF

#gi|116805391|gb|ABK27710.1|_type_1/III_gonadotropin-
releasing_hormone_receptor_hexahelical_variant_Gallus_gallus
MARLGGGTGQDAAAAAGCNVAVLRAAGGRRGGGRSHIRVLLRHLAAADLLVTVVVMPLDA
IWNITLQWRAGDLACRLLMYLRLLAMYASAFVTVVISLDRQAAILRPLAIARARCRNRAM
LRAAWMLSAALAVPQLFLFHTVTLHAPHNFTQCTTHGSFPQPWHETLYNMLSFSCLFLLP
LLIMVCCYTRILLEISRRMGSSLFSSRDVPLRCSGSNIPRARLRTLKMSLVIVSSFILCW
TPYYLLGLWYWFCPRAMQQKVPPSLSHILFIFGLFNACLDPITYGLFTIPFRRRCGCPCG
HSSEPEPPSPATGSFHCSASSLRGRQGMGGTEGPHPPIELGLPTGAGSCQSSAL

#gi|118421171|gb|ABK88281.1|_gonadotropin-
releasing_hormone_receptor_Kryptolebias_marmoratus
MNTSLWDSAAMYAVVGNHQPNASCNGSSGASNWTAGEDAPQLPTFTTAAKVRVVVTLALC
GVSAFCNLAVLWAAHADGKRKSHVRMLIINLTVADLLVTFIVMPVDAVWNITVQWLAGDL
ACRLLMCLKLLAMYSCAFVTVVISLDRQSAILNPLAINKARMRNRIMLTVAWGMSVVLSI
PQIFLFHSVTIVHPEDFTQCTTLGSFAARWHETAYNMFTFSCLFLLPLVIMITCYSRIFH
EISKRLRKDNLPSTEVHLRCSKNNIPKARMRTLKMSVVIVLSFIICWTPYYLLGLWYWFF
PDDLADKVSHSLTHILFIFGLLNACLDPVIYGLFTIRFRKGLRRYYGNAASAMDAENNTV
LTGSFTCPGSMSLKRELRRASQERLMLRSDSNRAEPPSLRNSFLTDDNDAERDPDPESAL

#gi|11993050|gb|AAG42574.1|AF144062_1_GnRH_receptor-3_Rana_catesbeiana
MNASDQPMGDGEAAPPGLCAFKGFNFSCVHANGFEKPHGPNITFLNEDHFVLPTFSTAAK
IRVAITCVLFISSACFNMATLWTITYKYRKKSHIRILIINLVAADLLITFVVMPLDAVWN
VTIQWYAGDVACRILMFLKLVAMYSSAFVTVVISLDRHAAILNPLGIGDAKKKNKAMLSV
AWTLSLLLATPQLFVFHTVSRSQPVHFVQCATVGSFKAHWLETLYNMFTFCCLFLLPLLI
MVFCYGRILVEISRKMKKAEVSSREVNLRRSYNNIPRARMRTFKMSLVIVLTFIVCWTPY
YLLGIWYWFSPEMLTSRKVPPSLSHILFLFGLFNTCLDPIIYGLFTIHFRREIRRVCRCA
TQGKDADATSLGTGSFRISTAAVPLKRSAGASGGSCKFDLEVTGVGLHSGKCEHCKRQIV
ESFM

#gi|11993052|gb|AAG42575.1|AF144063_1_GnRH_receptor-1_Rana_catesbeiana
MNISKEVSIKGCNNAQWLSSSCDLDVNMTSTNGTHTHFQLPTFSPAAKARVIITFVIFTL
SATCNLAALWSAARTSRKKRSHVRILILNLTTADLLVTFIVMPLDAIWNITVQWHAGDIA
CRILMFLKLLSMYSCAFVTVVISVDRQSAILNPLAINDAKKKNKIMLSVAWLMSAVLSLP
QLFLFHTVTITEPHNFTQCTTRGSFQQHWQETVYNMVSFVCLFLLPLLIMICCYSRILLE
ISKRMSKGTLSSKEVYLRCSKNNIPKARMRTLKMSVVIVSSFIICWTPYFLLGLWYWFYP
EIMEEKVSQSTTHILFIFGLVNACLDPITYGLFTIHFRKSLQRYCGGRRTSDADTSSSVT
GSFRCSMSSFRAKKMIVLNQELQVLQSCNGNFNNPELRLNGLGTSCL

#gi|11995301|gb|AAG42949.1|_GnRH_receptor-2_Rana_catesbeiana
MAMQLAIVNQSHLVVPDANVSSLGFPGPWSEPTFTAAAKVRVGVTCCFFLIASCSNVAVL
CSISGKRCKSHLRILILSLSIADLLVTLLVMPLDAMWNIMIQWYADEISCKILNFGKLFA
MYSAALVLVVISLDRHWAILYPLSFTSAGQRNRIMLWTAWIGSLLLASPQLFLFRLRTVP
GANFTQCATHGSFAQHWQETAYNMFTFCTLFVTPLGVMIVCYTRILWEIGKQMKHKNELA
RSKNDLISKARLKTLKMTLVIVVSFMVCWTPYYLLGIWYWFQPEMIYLTPEYVHHSLFLF
GLLHTCTDPLVYGLYTPSFKEDLRTWLRRLGGLLTRKAKNSKPLADSEMNIKDFTSMDGP
TTTATTVQSVF

#gi|122003017|sp|Q2V2K5.1|GNRHR_OCTVU_RecName:_FullGonadotropin-releasing_hormone_receptor_ShortGnRH_receptor_ShortGnRH-R_Shortoct-GnRHR_Octopus_vulgaris_gi|84095064|dbj|BAE66647.1|_GnRH_receptor_Octos
MDYLNDSMFNNMTYNITSTPLPDAPRFDNVYVSKLCVLGTVFVISFFGNTLVIIQIFRIR
GSRSTIQSLILNLAIADLMVSFFNILMDIIWSATVEWLAGNTMCKIMKYLTVFGLHLSTY
ITVSIALDRCFAILSPMSRSKAPLRVRIMITMAWVLSAIFSIPQAVIFQEQRKMFRQGMF
HQCRDSYNALWQKQLYSASSLILLFVIPLIIMVTSYLLILKTIVKTSRQFHDTPISPTSM
SCYSVNHGQIRTHLFERARKRSSRMSAVIVAAFILCWTPYYIIFLGFAFFQWDNSRTVIY
FFTLGTSNCMLNPLIYGAFTIYKVHRGRSGSANSPSGTRLMIMVNKRGRSTTTTTNRMSG
SGRRQLTTGQTITQCASLTNPHQPVRPSPGINSTTSPNGKMPTKPPG

#gi|126313702|ref|XP_001369168.1|_PREDICTED:_gonadotropin-releasing_hormone_II_receptor-like_Monodelphis_domestica
MTTSNDTVWGSSVGEEAWAGAGVQVEESALPTFSTAAKIRVGVTAALFLSSAVGNLAVLW
SVTRPRSSRLRPSPVRRLFGHLAAADLLVTFVVMPLDAAWNTTVQWLAGDVACRTLMFLK
LLAMYAAAFLPVVIGLDRQAAVLHPLGPRGGGRKLLGTAWGLSFLLALPQLFLFHTVRRA
GPVPFIQCVTKGSFKARWQEIAYNLFTFCGLFLIPLTAMAICYGRIVFSVSRHRTGKGEH
APAGQFALRRSFDNRPRVRLRALRLALLVLLTFVICWTPYYLLGLWYWFSPSMLSNVPPS
LSHILFLFGLLNAPLDPLLYGAFTLGCKKGHQGLGSDSSRGRMDSWRVPQQVVQPPSQLE
VNTEGNGKAGEMEETTL

#gi|126331566|ref|XP_001362289.1|_PREDICTED:_gonadotropin-releasing_hormone_receptor_Monodelphis_domestica
MANRVYSEQGQTHCSVINNSFPVTHRDLPTLTLSGKIRVMVTFFLFLVSTAFNASFLMKL
QRQNQKKEVKKLPRMKVLLKHLTLANLLETVIVMPLDGIWNVTVQWYAGEFLCKVLSYLK
LFSMYAPAFMMVVISLDRFLAITRPLAVKSNTKVGQSMIGIAWLLSVVFAGPQLYIFRMI
YIVDISGQRAIFSQCVTHGSFPEWWQEAFYNLLTFSCLFIFPLLIVLVCNAKIIFTLTQV
LHQDPHKLQLNRSKNNIPRARLRTLKMTVAFATLFTVCWTPYYVLGIWYWFDPEMLNRVS
DPVSHFFFLFGLLNPCFDPLIYGYFSL

#gi|134053892|ref|NP_001076809.1|_adipokinetic_hormone_receptor_Tribolium_castaneum
MNFSETLWKMKDPMSASETVQDHRNLLDWSKTSLDNATEHKLPISMRFNEGHQLSIIVYS
ILMVFSAIANTTVLVLIVKRRRKTPSRINTMLMHLAIADLLVTFLMMPLEIGWASTVSWY
AGDAMCRIMMFFRMFGLYLSSFILVCISVDRFYAVLKPLYLRALDRRDKFMLLGAWLGAT
LCSIPQMVVFHVESHPNITWYQQCVTYNVFPTYAHELTYLLFGMVMMYALPLAVIIFSYA
SILLEIRRRTRNPYGDSVTRSSLAFLGKAKVRTLKMTIIIVLVFFVCWTPYYVMCIWYWL
DRESAKNVDQRIQKALFLFACTNSCMNPVVYGVFNIRARRTGRKVSPRVNTIKHTSCIPT
PNGDSRLPPLEISLKTLE

#gi|139152230|gb|ABO77113.1|_gonadotropin-releasing_hormone_receptor_1a_Branchiostoma_floridae
MVNASETQPTLCPSSNVTNASTSACFNVSDVQGNGPGTNTTTFSLPYPLPVFDYPAKVRV
IVTFVLCFASLVGNLLVFITMFRNRARKSRVNLLIMHLAVADIFMTLIVMPLDGVWNLTV
QWYAGDVACRILQFLKLWALYASTFILVVISIDRCMAILRPLSSANGYKRGKIMVGIAWG
AGAVLSTPQAVIWHLIHVHPTPMVTFIQCSTHGFYTADWQEQLYNACVFFLVFIFPLTIM
ITCYLLILVKITRKYRELTDPTANQDNILRHSGSARLAKAKDRTWLMTFVIVSAFVINWS
PYYVISIWYLVDKSMVHYISKSASHTLFIFGLTNPCLDPLIYGLFSINFSREFRRCCGFL
KRRDLANESPYTMLTVVGAHGDTAGTRMTPSVSASAQFISTPT

#gi|139152245|gb|ABO77114.1|_gonadotropin-releasing_hormone_receptor_2a_Branchiostoma_floridae
MDCPGQGHNLYSNSTNSTVVCTDDDILAGNTNCTNVTDANSSGLAFPLPVFTFSTKVRVS
LTFILMFISLVGNLIVFVTMFRNRARKSRVNFLIMHLAVADIVMTLIVMPLDGVWNLTIQ
WYGGEAACRILMFLKMWALYASTFILVVISIDRCTAILRPLSMTDAYKRCKIMVMLVWVI
GGILSIPQLFIFHLVTPAPTFTQCATHGVYNAPWQEPLYNSFHFVMVFILPLAIMITCYL
LILAEISRKHRELTDPLGREEGHRLRHSGADRMAKAKEKTWLMTFVIVSAFVINWSPYYV

LMIWFLVDRCIVFTVPSAVSDALFIFGLTNPCLDPLIYGLFSINFVREFRRCCGWLKRKD
FVTRDSTFGGTTVVSRVDTVAIPLRSVKTTRNGTSTT

#gi|147898501|ref|NP_001079176.1|_gonadotropin_releasing_hormone_receptor_type_I_Xenopus_laevis
MAVNQTQRSLVISDNNASATGNADPWTEPTFTLAAKVRVGVTCCFFLIASCSNVAVLCSI
SGKRCKSHLRVLILSLSVADLLVTFLVMPLDALWNVMVQWYAGELSCKVLNFGKLFAMYS
AALVLVVISLDRHWAILYPLSFTSAGQRNRIMLWTAWITSLLLASPQLFLFRLRTAPGVN
FTQCATHGSFTQHWQETAYNMFTFCTLFVTPLVVMIVCYTRILWEIGKQMKHKNELARSK
NDLISKARLKTLKMTLVIVASFMVCWTPYYLLGLWYWFQPEMINQTPEYLNHSLFLFGLL
HTCTDPLVYGLYTPSFKEDLRSWIRRVSTLLSRKEKNSKQLAGSELNIKDLTSMEGPTST
AVTMQSVF

#gi|147901685|ref|NP_001091663.1|_gonadotropin_releasing_hormone_receptor_4_Danio_rerio
MNDSSPTSENIMFHQLTADTLNGSCDLPTCNNNTGEAALQLPTFSMAAKVRVIITFTLCA
VSAVCNLGVLWAASTNNKRKSHVRILIMNLTVADLLVTFIVMPVDAAWNITVQWLAGDLA
CRLLMFLKLVAMYSCAFVTVVISLDRQSAILNPLAINKAKKKNKIMLSVAWAMSVVLSVP
QMFLFHNVTITVPANFTQCTTRGSFVKHWQETIYNMFTFICLFLIPLAIMICCYTRILVE
ISRRMTKGNISSKEVHLRRSHSNIPKARMRTLKMSIVIVTSFIVCWTPYYLLGLWYWFLP
EDLEETVSHSLTHMLFIFGLFNAILDPITYGLFTIHFRKGLKRYCRSAVVLTESENNSII
TGSLKCSPSPFRMKRVTQSGTGTDPKQNTSTVGEEDKKAADGKTKE

#gi|148227062|ref|NP_001079211.1|_gonadotropin-releasing_hormone_receptor_Xenopus_laevis
MLTMSYQGIMDSQDLCALNRSCFHLKEQEKTYGPNITVLNDKAFILPTFSTAAKIRVAIT
CVLFIFSACFNIAALWTITYKYKKKSHIRILIINLVAADLFITLVVMPLDAVWNVTLQWY
AGDLACRVLMFLKLAAMYSSAFVTVVISLDRQAAILNPLGIGDAKKKNKIMLCVAWFLSY
LLAIPQLFVFHTVSRSEPIHFVQCATVGSFQAHWQETIYNMFTFFCLFLLPLLIMVSCYT
RILMEISHKMKATCVSSKEIDLRRSSNNIPRARMRTLKMSLVIVLTFIVCWTPYYLLGIW
YWFSPEMLTEEKVPPSLSHILFLFGLLNTCLDPIIYGLFTIHFRREIRRVCRCAAQGKDH
DTASVGTGSFRITTTPAPIKRTVGVLGGSGKFELEVTGHGLHSGKCDQCQGRIVESFM

#gi|14916996|sp|O42329.2|GNRR2_CLAGA_RecName:_FullGonadotropin-releasing_hormone_II_receptor_ShortGnRH_II_receptor_ShortGnRH-II-R_AltName:_FullType_II_GnRH_receptor
MSGNTTLLLSNPTNVLDNSSVLNVSVSPPVLKWETPTFTTAARFRVAATLVLFVFAAASN
LSVLLSVTRGRGRRLASHLRPLIASLASADLVMTFVVMPLDAVWNVTVQWYAGDAMCKLM
CFLKLFAMHSAAFILVVVSLDRHHAILHPLDTLDAGRRNRRMLLTAWILSLLLASPQLFI
FRAIKAKGVDFVQCATHGSFQQHWQETAYNMFHFVTLYVFPLLVMSLCYTRILVEINRQM
HRSKDKAGEPCLRRSGTDMIPKARMKTLKMTIIIVASFVICWTPYYLLGIWYWFQPQMLH
VIPDYVHHVFFVFGNLNTCCDPVIYGFFTPSFRADLSRCFCWRNQNASAKSLPHFSGHRR
EVSGEAESDLGSGDQPSGQ

#gi|156182200|gb|ABU55292.1|_type_I_gonadotropin-releasing_hormone_receptor_Callorhinchus_milii
MCLKPLPNNTMIGSVESGKNNFTLHNDSLDIAVSNSSLKFPTLSISGIIRVAITFTLFIL
SIAMNGIFLLKLSRQHKKKASRLKLLLDNLMVANLVETIIVMPMDGIWNIMVQWYGGQFL
CKVLNFLKLFSMYSPAFMVVVISIDRCLAVTKPLKSATQSTQIRKYMIYTAWLFSFVLAL
PQLWLFRMIHYSEPYAFSQCNTLRSFYNQWDQTIYNFFTFGFLFVIPLFIMLFCNFKIIF
KMMKILRHNVNEISLNRSKNIIPQARMKTLKMTIAFVTSFIICWTPYYLIGIWYWIDPDL
HNRLPEPMNHFFFVFGLLNPCFDPLIYGYFSL

#gi|156254302|gb|ABU62659.1|_gonadotropin-releasing_hormone_receptor-4_Danio_rerio
MNDSSPTSENIMFHQLTADTLNGSCDLPTCNNNTGEAALQLPTFSVAAKVRVIITFTLCA
VSAVCNLGVLWAASTNNKRKSHVRILIMNLTVADLLVTFIVMPVDAAWNITVQWLAGDLA

CRLLMFLKLVAMYSCAFVTVVISLDRQSAILNPLAINKAKKKNKIMLSVAWAMSVVLSVP
QMFLFHNVTITVPANFTQCTTRGSFVKHWQETIYNMFTFICLFLIPLAIMICCYTRILVE
ISRRMTKGNISSKEVHLRRSHSNIPKARMRTLKMSIVIVTSFIVCWTPYYLLGLWYWFLP
EDLEETVSHSLTHMLFIFGLFNAILDPITYGLFTIHFRKGLKRYCRSAVVLTESENNSII
TGSLKCSPSPFRMKRVTQSGTGTDPKQNTSTVGEEDKKAADGKTKE

#gi|156555742|ref|XP_001602277.1|_PREDICTED:_cardioacceleratory_peptide_receptor_Nasonia_vitripennis
MDFQLPSETWVSASTWPIETALGGILDASIVGYLSTEVSTLNNISRIIERNITDEIDPFY
FYQTEQFTVLWLLFSVIVVGNTSVLVGLIFGKRRKSRMNFFIKQLAFADLMVGLISVLTD
IVWRSTVAWYAGNVACKIIRFMQVVVTYSSTYVLVALSIDRYDAITRPMNFSRSWCRARA
LVTAAWSISVLFSVPIIFLYEERIVEGKNQCWIELGSPANWRIYMTVVCLTLFIIPAIII
GGCYMVIVWTIWSQSSALRHDPTRDTRRASSRGLIPRAKIKTVKMTFVIVFVFILCWSPY
IVFDLLQVYGHVPRSQTNIAVATFIQSLAPLNSAANPIIYCLFSTPFCKTVSNMQAVSWF
SGLCPSNPHLCFGTNTHGNSTRTTVTTSLTAHSSRRSGHISMLHPSSRKRVMVSLV

#gi|157119384|ref|XP_001659389.1|_cardioacceleratory_peptide_receptor_Aedes_aegypti
MKSNYFPTVSIVSYRIFFSVFLFFSFQTEQFAVLWILFIVIVLGNLAVLVTLFMNKNRKS
RMNFFIKQLAIADLSVGLLSVLTDIVQRITISWLAGNVACKVIRFIQVWVTYASTYVLVA
LSIDRYDAITHPMNFSGCWKRARRLVGAAWGFSAIFSSPMFYLYEERVIQDQLQCWIDLG
NALRWQLYMCWVATSLFVIPAFIISACYVIIIKTIWSKGSVLGPVGGKYRYRNGSAELAR
RRASSRGIIPKAKVKTVKMTIVIVIVFVLCWSPYIVFDLLQVFEQIPKTQTNIAIATFIQ
SLAPLNSAANPLIYCLFSTHFVKTLKRLPPFRWLFSSGLCGSPPDEVVSHNGSGQTSSSR
LRNHHSSDSMRTLTTSLTTSTRRPATIIRTSRVII

#gi|157123803|ref|XP_001653920.1|_gonadotropin-releasing_hormone_receptor_Aedes_aegypti
MIRGHTETTVAVIIVYCVLFIIAAGGNLSVVITLFRSRRHRRSRVSLMICHLAVADLMVA
FIMIPLEVGWRITVQWHAGNVACKVFLFMRAFCLYLSSNVLVCVSLDRCFAVIYPLRVSA
ARKRGKIMLGGAWFIAFANAIPQSIIFRVQHHPNVPDFTQCVTFGFFTTPAMETAYNLFC
VVAMYFMPLMVISAAYTVILCEISNRSREKETSDTSHTGGMRLRCNDLTHIERARQRTLR
LTITIVVVFVWCWTPYVVMTLWYMFDRESALKVDGAIQDGLFLMAVSNSCMNPLVYGSYA
MKCRRPWRRQMAPNGVQTPNAAQRRSTGK

#gi|157129044|ref|XP_001655249.1|_gonadotropin-releasing_hormone_receptor_Aedes_aegypti
MSNAILKTERDEVLNYSHSYGENYNNDVNTMPYVLSSSTSKTGVFDNETWYGTNSSNWNE
PLPIDMQFNDGHKLQIVVYSVLMVISAIGNITVLALLIKRRLKSHSRIDMMLTHLAIADL
LVTFLMMPLEIGWAATVQWRAGDIMCRVMAFFRTFGLHLSSFVLVCISVDRYYAVLQPLN
LSKSRGKIMILIAWAMATLCSAPQPFIFHVEIHPNHTWYEQCVTYNTFSNDNYHTVYNIL
VMMFMYALPLLTIICSYASIYMEIFRHSRMPNSEGFRRSSIDVLGRAKRRTLKMTITIVM
AFVICWTPYYVMSVWYWLDQKSAENVDQRVQKGLFLFACTNSCMNPIVYGIYNVKLRKKK
KPDGVKSGQSSVILRNSAKYTRHSESIRSSSD

#gi|157132199|ref|XP_001662510.1|_somatostatin_receptor_Aedes_aegypti
MEWNDTVGNDNGSELYRMDSIATESTIVTTMMVPAVAPPAVGLTFPSDLLTFIRQTASGS
ASSNLSYLLSNITRALSSPSNEFNLSRRFPLHPFFPLYNDTVFESCPSIQMPIGNLISMI
LYAVVGLIGLFGNTLVIYVVLRFSKMQTVTNMYILNLAIADQCFLIGIPFLIITMHLGEW
TFGNTMCKAYMVSTSITQFTSSIFLFIMSADRYIAVCHPISSPRFRTPLVSKIVSFFAWT
TSALIMLPVMLYANTVHRDKGKISCNIVWPSENASNSGTTFTLYSLILGFAVPLSLILMF
YYLVIRKLRTVGPKSKSKEKKRSHRKVTKLVLTVITVYVLCWLPYWISQVALINSPPDIC
KSRLEITVFVLVSCLGYSNSAMNPILYAFLSDNFKKSFLKACTCTKGKDINAQLQIENSF
FPRFARNRGSERGNSTKILHPGRQKVGDQTHPNDTANNNNTNNANANNGSSRCSGPGGPI
SNTNTNGNTTTTTMTTTTMGLGGGSSSSNVVPIASKILDTSSRPPVLHTDL

#gi|157278501|ref|NP_001098352.1|_gonadotropin-releasing_hormone_receptor_1_Oryzias_latipes
MNESSCCHPPAVTYQQSSRWDLNASCDWSAPRCNWTSGDGPLQLPTFSTAAKVRVIITFI
LCGVSTLCNSAVLWAAIGHKRKSHVRVLIINLTAADLLVTFIVMPVDAAWNITVQWLAGD
AACRFLMFLKLQAMYSCAFVTVVISLDRQSAILRPLSISAAPRRNRSMLTVAWTMSAVLS
VPQMFIFHNVTITHPANFTQCTTRGSFVTHWQETAYNMFTFTCLFLLPLSIMIICYTRIF
IQISKQMTKKNVSSDEPHLRCSKNNIPKARMRTLKMSVVIVVGFIVCWTPYYLLGLWYWF
FPDDLEGKVSHSLTHILFIFGLFNTCLDPIIYGLFTTRFHRGRRKCYGGATATLSLESKV
VTAEAVKRSSDASASRGDAGEKDNNSARTERQSSGGNI

#gi|157278583|ref|NP_001098392.1|_gonadotropin-releasing_hormone_receptor_2_Oryzias_latipes
MTKADTSRMTGNRSSLAPPTSSVVPAHLNSSLYPPIPDWEPPSFSQAAQFRVGAIFILFL
FAACSNTALLTSVWCGRGRRLASHLRPLMLSLASADLMMTFVVMPLDAAWNVTVQWYGGD
ALCKLLCFLKLFAMHASAFILVVITLDRQHAILHPLDALSAHRRNRFMLLLAWTLSLLLA
SPQLFLFRTIKVDRANFTQCVSHGSFSHRWQETVYNMFHFITLYVVPLLVMSCCYSRILL
HIHQQHLRDKAGESHLRRSGTDIIPKARMKTLKMSVVIVLSFVVCWTPYYLLGIWYWFQP
DMLRVTPEYVHHILFVFGNLNTCCDPVIYGFYTPSFRADLAACCRWSKESSSPMSLNRLS
TRRGPHSGEHEPQ

#gi|157278585|ref|NP_001098393.1|_gonadotropin-releasing_hormone_receptor_3_Oryzias_latipes
MFHHLTDQTVNGSCLGASTDCNKSADGDALQLPTFSTAAKVRVIITFTLCAVSAVCNLLV
LWAAGKGGKRKSHVRILIMNLTVADLLVTFIVMPVDAAWNITVQWQAGDLACRLLMFMKL
VAMYSCAFVTVVISLDRQSAILNPLGISEAKRKSKIMLTVAWTTSVILSLPQMFIFHNVT
ITVPENFTQCTTHGSFVQRWQETLYNMFTFVCLFLLPLVIMIFCYTRILVEISSRIARNN
MVSRDIHLRRSHNNIPKARMRTLKMSIVIVTSFIICWTPYYLLGLWYWLFPEKMEETVSH
SLTHMLFIFGLFNACLDPITYGLFTIHLRQGARRRRQISNAQTELENNSCLMQMSCLSAH
RQNVSSGLSKHTEEINDNSSTKNASSPSISVSRI

#gi|160333803|ref|NP_001103908.1|_vasopressin_V1a_receptor_Gallus_gallus
MRLGGGGGSPRAAGPPGNGSRWRGAAEDGSSPSPEAWSGAPNGSAGDWDPFGRDEELAKL
EIAVLAVTFAVAVLGNGSVLLALRRTPRKASRMHLFIRHLSLADLVVAFFQVLPQLCWEV
THRFHGPDGLCRVVKHLQVFGMFASAYMLVAMTADRYIAVCHPLKTLQQPTKRSYAMIAA
AWALSLLLSTPQYFIFSLSEVERGSRVYDCWAHFIMPWGPRAYITWITGGIFVAPVLILA
TCYGFICFRIWRSARGRARPGEAAGGGPRRGLLLAPCVSGVKTISRAKIRTVKMTFVIVS
AYVVCWAPFFTIQMWSVWDQHFPWVDSENTATTVTALLASLNSCCNPWIYMFFSGHLLQD
CVQSFPCCQKIKQTLSKEDSNSNSRRQTSFTNNRSPTHSLNTWRESPHSKSTSFIPVPT

#gi|160905682|gb|ABX52399.1|_ACP_receptor_Anopheles_gambiae
MYLAAGLLNIMDISLQHEYLQEYLQSAAAMANFSGANPYGLGGFGGLAPNGTGLLGGLDK
NGTEVTITAPGHTDSTVAVIIVYCVLFVIAAGGNLSVVITLFRSRRHRRSRVSLMICHLA
VADLMVAFIMIPLEVGWRITVQWHAGNVACKVFLFMRAFCLYLSSNVLVCVSLDRCFAVI
YPLRVSAARKRGKIMLGGAWFIAFVNAMPQSIIFRVQQHPQVPGFTQCVTFGFFATPGLE
TAYNLFCVIAMYFLPLMIISGAYTVILCEISNRSREKETSDSNSTGTMRLRCNDLTHIER
ARQRTLRLTITIVVVFVWCWTPYVVMTLWYMFDRESAAKVDVAVQDGLFLMAVSNSCMNP
LVYGSYAMKCRLPCRRRNTLGGAQTPNAAQRRSTGTYGWWDCMRVHVLCFPIEQTLEFP
RRKGRGHGSRPSFWKLYVCSDYNSLFWKRVSMPTAMGSKNEPSVLHEYLEKHLI

#gi|160905684|gb|ABX52400.1|_ACP_receptor_Tribolium_castaneum
MQAVGKMGEEHYDEDSKSNFSVLNETLDGFANETVSPDVLFQQNLTVILVYSALFVVAAV
GNLTVFISLFRSRHRKSRISLMIRHLAIADLIVTFIMIPIEVGWRLTGKWIAGNVACKVF
LFLRAFGPYLSSNVLVCVSLDRYFAVLHPLRVNDARRRGKIMLAFAWGTSFVYCIPQSFV
FRVRAHPKYPNYEQCVSFGFFENTAQEIAYNLMCVMCMYFIPLFVIIVAYTAIMCEISKN
SKETKGESYRTSNGRMRLRRSDISNIERARSRTLRMTITIVAVYVWCCTPYVIITMWYMF
DRASATSLPEWLQDTFFMMVVSNSCMNPIVYGSYVINFQRVNCNCFCFRKTASESHLNVG
SGATRSTAMVHGAGGNGYTRSPTPKSNLNLTGLLSKSRLPDKPPSVGHISFLSEPRTARN

YRSSFHSEPCSRTRMCPDELCLDTSCHSADYYSSAVL

#gi|166157856|ref|NP_001107547.1|_gonadotropin-releasing_hormone_(type_2)_receptor_2_pseudogene_Xenopus_(Silurana)_tropicalis
MPTEMNITKDTDSITCNNVLWLSSYCTMDRNTSSAAEAQNPFQLPTFSPAAKARVIITFV
IFALSAFCNLAVVWASTNTSRKKRSHVRILILNLTTADLLVTFIVMPLDAIWNITVQWQA
GDLACRILMFLKLLSMYSCAFVTVVISIDRQSAILNPLGISEAKKRNKIMLSVAWLMSIL
LSLPQLFLFHTVTITEPQNFTQCTTRGSFQEHWQETVYNMVSFVCLFLLPLLIMISCYSR
ILIEISRRMSKGALSSKEVYLRRSKNNIPKARMRTLKMSIVIVSSFIICWTPYYLLGLWY
WFYPEAMEERVSQSLTHILFIFGLVNACLDPITYGLFTIHFRKGLHRYCRGGRASDLDTS
SSVTGSFRCSMSSFRAKKIVLNQELQVMQGYNGSSNNSEFRTNGLNSSCL

#gi|166157864|ref|NP_001107548.1|_type_2/nmI_gonadotropin-releasing_hormone_receptor_Xenopus_(Silurana)_tropicalis
MAEQSAIVNQTESTLVISENNASASGNPDPWTEPTFTLAAKVRVGVTCCFFLIASFSNVA
VLCSISGKRCKSHLRVLILSLSVADLLVTFLVMPLDALWNVMVQWYAGELSCKVLNFGKL
FAMYSAALVLVVISLDRHWAILYPLSFTSAGQRNRIMLWTAWITSLLLASPQLFLFRLRT
APGVNFTQCATHGSFTQHWQETAYNMFTFCTLFVTPLVVMIVCYTRILWEIGKQMKHKNE
LARSKNDLISKARLKTLKMTLVIVVSFMVCWTPYYLLGLWYWFQPEMINQTPEYLNHSLF
LFGLLHTCTDPLVYGLYTPSFKEDLRSWIRRVSSLLSRRAKNSKQLAGSELNIKDLTSME
GPMSTAVTMQSVF

#gi|166157872|ref|NP_001107549.1|_gonadotropin-releasing_hormone_receptor_Xenopus_(Silurana)_tropicalis
MSYQEIMDPCALNLSCFHINEQEKNSGPNITLLNDKAFVLPIFSTAAKIRVAITCILFVF
STCFNVAALWTITYKYRKKSHIRILIINLVAADLFITLVVMPLDAVWNVTLQWYAGDLAC
RVLMFLKLAAMYSSAFVTVVISLDRQAAVLNPLGIRDAKKKNKIMLCVAWSLSYLLAIPQ
LFVFHTVSRSEPVHFVQCATVGSFQAHWQETIYNMFTFFCLFLLPLLIMVFCYARILMEI
THKMKAACVSSKEIDLRRSSNNIPRARMRTLKMSLVIVLTFIVCWTPYYLLGIWYWFSPE
MLTKEKVPPSLSHILFLFGLLNTCLDPIIYGLFTIHFRREIRRVCRCAAQGKDHDTASLG
TGSFRISTTTVPMKRAVLGGSGKLELEVTGYGLHSGKCEQCRGKIMESFM

#gi|169264531|emb|CAP19988.1|_G_protein_coupled_receptor_Crassostrea_gigas
MNSDADADTTIRFNQSSLYEDVYSTAPNDGVLLMKFTCENGFDEFQKNGSCQNNTTEMLP
MDLVFTDENMVTIVAYTCMFIVAACGNLTVFITLFRNRNIKSRVNQFIFHLSIADLVVTF
IMLPLEIIWNITVAWKAGDPACRILMFFRILGLYLSSFILVTISLDRYFAIVHPLSLNDA
DKRGRIMLILAWCFSIVASIPQSVIFHVETHPKYRTFRQCVTFNFFPSHNHELAYNLFNL
ITLYALPLLIITTSYSLILWEISKKTKQCKEETKCLSTRSRLRRSSVGNMERARIRTLKM
TLVIGFCQSARPQGTEGAFSFRCVQLMHGSYCLWMFTINFKREFVRCCCCLKTSWKRHKL
QRLTGKFQTSTGVQRGPLSHTASNNSCRSLPGTNVVKFFDDAAICGNGGSPVLSRDLLKP
KLDTGSRRANVNNFMKVNLPALEKSVTMDKH

#gi|169403918|dbj|BAG12379.1|_gonadotropin_releasing_hormone_receptor_type_A_Fundulus_heteroclitus
MNTCLCETAVTMRDLVTDLQANVTCNCSLASSNWTEEEVPTLPTFTTAAKIRVIITFILC
GTSAFCNLAVLWAAHRDGKRKSHVKVLIVNLTVADLLVTFIVMPVDAAWNITVQWLAGDF
ACRLLMFLKLLAMYSCAFVTVVISLDRQSAILNPLAINKARRRNRIMLAVAWGMSVVLSV
PQVFLFHNVTIIHPEDFTQCTTWGSFATRWHETAYNMFTFSCLFLLPLVIMITCYARIFH
EISKRLKKDNLDLRCSKNNIPKARMRTLKMSVVIVSSFIICWTPYYLLGLWYWFFPDDLE
GKVSHSLTHILFIFGLVNACLDPVIYGLFTIHFRKGLRRYYGNAASTAEVESTTVITGSL
YSPPNSLSLRREFRRFSQEKLVVCSDGHRRVEPPSLNSSFLEDRDLQQSSPESVL

#gi|170029979|ref|XP_001842868.1|_gonadotropin-releasing_hormone_receptor_Culex_quinquefasciatus
MGRKSYTDSTVAVIIVYCVLFIIAAGGNLSVVITLFRSRRHRRSRVSLMICHLAVADLMV
AFIMIPLEVGWRITVQWHAGNVACKVFLFMRAFCLYLSSNVLVCVSLDRCFAVIYPLRVS
AARKRGKIMLGGAWFIAFANALPQSIIFRVQQHPQVPGFTQCVTFGFFRTPGMETAYNLF

CVIAMYFMPLMIISAAYTVILCEISNRSREKETSDTSHTGGMRLRCNDLTHIERARQRTL
RLTITIVVVFVWCWTPYVVMTLWYMFDRESAIKVDGAIQDGLFLMAVSNSCMNPLVYGSY
AMKCRWPWKRQNVPNGVATPNAAQRRSTDAVSGMAGPHSDRLTGRDNKDELMYEKERSIK
LNQFGLANGAPGQTKIGMNSTGE

#gi|171850880|emb|CAP17413.1|_G_Protein_coupled_receptor_Crassostrea_gigas
MNSDADADTTIRFNQSSLYEDVYSTAPNDGVLLMKFTCENGFDEFQKNGSCQNNTTEMLP
MDLVFTDENMVTIVAYTCMFIVAACGNLTVFITLFRNRNIKSRVNQFIFHLSIADLVVTF
IMLPLEIIWNITVAWKAGDPACRILMFFRILGLYLSSFILVTISLDRYFAIVHPLSLNDA
DKRGRIMLILAWCFSIVASIPQSVIFHVETHPKYRTFRQCVTFNFFPSHNHELAYNLFNL
ITLYALPLLIITTSYSLILWEISKKTKQCKEETKCLSTRSRLRRSSVGNMERARIRTLKM
TLVIVSVFVICWTPYFVLSAWWFDSDSASQLDPKVQRGLFLFAVSNSCMDPIVYGMFTIN
FKREFVRCCCCLKTSWKRHKLQRLTGKFQTSTGVQRGPLSHTASNNSCRSLPGTNVVKFF
DDAAICGNGGSPVLSRDLLKPKLDTGSRRANVNNFMKVNLPALEKSVTMDKH

#gi|171850884|emb|CAP17415.1|_G_Protein_coupled_receptor_Crassostrea_gigas
MNSDADADTTIRFNQSSLYEDVYSTAPNDGVLLMKFTCENGFDEFQKNGSCQNNTTEMLP
MDLVFTDENMVTIVAYTCMFIVAACGNLTVFITLFRNRNIKSRVNQFIFHLSIADLVVTF
IMLPLEIIWNITVAWKAGDPACRILMFFRILGLYLSSFILVTISLDRYFAIVHPLSLNDA
DKRGRIMLILAWCFSIVASIPQSVIFHVETHPKYRTFRQCVTFNFFPSHNHELAYNLFNL
ITLYALPLLIITTSYSLILWEISKKTKQCKEETKCLSTRSRLRRSSVGNMERARIRTLKM
TLVIVILPVS

#gi|18075913|emb|CAD11992.1|_gonadotropin-
releasing_hormone_receptor_Dicentrarchus_labrax
MNTTLCDSAVALYHLTTDHQLNASCNYSSPTSNWTSGGGALQLPTFTTAAKVRVIITCIL
CGISAFCNLAVLWAAHSDGKRKSHVRVLIINLTVADLLVTFIVMPVDAVWNITVQWLAGD
LACRLLMFLKLQAMYSCAFVTVVISLDRQSAILNPLAINKARKRNRVMLTVAWGMSVVLS
VPQLFLFHNVTIIHPEDFTQCTTRGSFVTHWHETAYNMFTFSCLFLLPLVIMITCYTRIF
CEISKRMKKDNLPSNEVHLRRSKNNIPRARMRTLKMGIVIVSSFIVCWTPYYLLGLWYWF
FPDDLEGKVSHSLTHILFIFGLVNACLDPVIYGLFTIHFRKGLRRYYCNATKAADLDNNT
VITGSFICAANSLPLKREASQERFMLYSDNHSRAESTSPRSSFLRDPNQSSSESNL

#gi|184161263|gb|ACC68668.1|_gonadotropin-
releasing_hormone_receptor_3_Branchiostoma_floridae
MADASSNRSGGQHVIWTDQPESMWNSTEDELDTFLYPNSTANSSDMWDFDFPLPRFTDVT
MAKIIIVVVTFVLSFVGNVTFLITSWRLRRNRRARPLQSLLVHLAIADLIVTLVTMPSLG
IWFYTVAWLAGNGMCKLIKSLQVLGLYLSTYLTVAISIDRCISVVKPMCRNTAKRRRNMV
AVCWILSTIFSIPQAVIFHVESPVPDFQQCVTFGFYTAKWQEQLYNGLVLVVMYPIPLLV
ILICSVLTFIRLKKEGQDKDTFRTRNPTRQRLLLKARNNTLRTTAGIMTSFILCWTPYFV
TLVWILFFNWQTVSPVVFDVLFLFGIFNSCVNPIVYGLSMFKKTTARPTLSLIEFSSPFL
TRSERRSASVNSRISRTYSHVSLSTRRSWQPSSESTTSSRPNALNNQHSHSASHLMTGLP
CRYGVSRQVRGGSQQKYLHPNTTTSPGPSTRSPKEASWHLKRATLTHAHSSPSLLTSMEK
SERPNRRQSEALAVPTLPTICLTPPAESRNTAFFSAALCLLHNEYANSPTQPPVADIPYQ
PTAVNQQGDQHYLSRRLSVALPMIPPTPDTPGSWETSSWQFVGKISCSLCRYDSSSPSAS
YPDRGECVTGRPQVNARRCISLGNTDSKVNPKITERLNAEIVQKMRNSVQETKISEYDLS
FEVASFRPPLPRLSVQESCVQSSGRMASGASIVGSEGSIQSSLESPLHSPASSVTSIDCR
HRLSLCSFREGRIPSDLHSALRPKHYSDDNLKLSCRKRKAKRRVSFKTVHFLAEPTVSDS
SVPSEGSNSSTPDHHQEPTRARNTSNHRTVSADIKWHVGCQLTRKTSSTLP

#gi|185133560|ref|NP_001117823.1|_gonadotropin-
releasing_hormone_receptor_Oncorhynchus_mykiss
MIIMYVITIRIYMFYFIYSHSFKPCLFQPRMSGNLSLLRPPLVGVTGSMQPALSNMSQFP
PLVDWEAPTFTRAAQFRVGATLILFLFAACSNLALLVSVCRGRGRRLASHLRPLIMSLAA
ADLMMTFVVMPLDAIWNITVQWYGGDATCKMLCFLKLFAMHSSAFILVVVSLDRHHAILH
PLDSLNSHHRNKRMLGLAWGLSVLLALPQLFIFRAIKAEGVDFTQCVTHGSFKERWQETV
YNMFYFVTLYVFPLLVMSFCYTHILIEINQQLHRNKAGESCLRRSGTDMIPKARMKTLKM

TIIIVMSFVVCWTPYYLLGIWYWFQPEMLQVTPEYIHHALFVFGNLNTCFDPVIYGFYTP
SFRADLAMCWCYRRRDINMSPRSLDRLSAHQGPHSGEQDSDAPGVEQTKETGGDGR

#gi|189491898|gb|ACE00761.1|_adipokinetic_hormone_receptor_Manduca_sexta
MDIEDKVSGPGGASQKNWTHLNTSYDELPLDMRFNHGHMVSMVVYSVLMVVSATGNLTVL
SQLVRRKRAGRASRLDVLLMHLAVADLMVTFLMMPLEIAWAGTVQWLAGDLMCRVMMFTR
TFGLYLSSFVLVCIAIDRYYAILKPLNVTWEARVRRALVVSWVGAGLASLPQSFIFHLEE
HPDVKGYFQCVSYGSLPTVHHELAYFLVNMILMYVIPLVSMLYCSSAALLEIIKRANTSN
DKMRRSGVGILGRARARTLKMTVTIVLVFFTCWSPYYCYCLWYWIDKESIKNLDPAIQKA
MWLFSCTNSCANPIVYGLFNRNRWTWRSGHNVRHRSGSMRRGSRLPYGESMEISAATLNR
ARNSLGSDRNGRRDSAFNTYNGTQKHWNTINNNHVSNGMV

#gi|195030652|ref|XP_001988182.1|_GH11026_Drosophila_grimshawi
MAQSGEVNEVVYDHRILRDWSNVNNTNGTMHFSKDMIFNNGHRLSIIVYSILFVISTIGN
STVLFLLTKRRLRGPLRIDIMLMHLAIADLMVTFLLMPLEIVWAWTVQWRSTDLMCRLMS
FFRVFGLYLSSFVMVCISLDRYFAILKPLQRSHNRGRTMLAIAWLGSVVCSIPQAFLFHL
EEHPDVKGYFQCVTFHSFVNDFVDSLYQIATMCAMYAFPLIAFIYCYGAIYLEIYRKNQR
VLKDVIAERFRRSNDDVLSRAKKRTLKMTITIVIVFIICWTPYYFICMWYSLDKSSVDQV
NSLVRKALFIFASTNSCMNPLVYGLYNIRGRMNNNNNVSVNNRHTSLSNRLDSSNQLLQK
PISTLQHGNGNGNVVAAAVAATTKLAHVVRLKTNGDVQSPAASAPLGSQLDVDDDVSVSV
VTIRCQEQTPDQPKAPNIYLK

#gi|195126150|ref|XP_002007537.1|_GI12336_Drosophila_mojavensis
MDFEDTPRLRLPVSNSSAVAGAPDLLDSAAFNETDSNWATAANFTRLFVAAATEVAPSIA
NYTLNILDAGLALATEATATTAAAAADTNGNSETSYAIMTHSRLNSSTLGHTNSLHELSP
LAEQVPEHVMDHAPQLSRSGMLKVYVLAVMALFSMLGNLLTIWNIYKTRITRRNSRHTWS
AIYSLMFHLSIADVLVTGFCLIGEAAWSYTVQWLANELTCKLVKLFQMFSLYLSTYVLVL
IGVDRWIAVKYPMKSLNMAKRCHRLLGGTYILSLVLSLPQFFIFHVARGPFVEEFYQCVT
HGFYTADWQEQMYATFTLVFTFLLPLCILFGTYMSTFRTISSSEKMFQGSKLANYSTTKQ
LPTQTNRQRLIHKAKMKSLRISVVIIIAFLICWTPYYVMMIIFLFWNPDKKLGDELQDAI
FFFGMSNSLVNPLIYGAFHLCPGKGNKSGTGGGNNNAYSLNRGDSQRTPSMLTAVTQVDA
GGSGRHLRTFRQQSYYRSSSNGTTGGPFKEQVGLLQVGSSSGCAATPQMMRKSSTVRSPH
PNHSSAIAGRHSGCLREQEQLLLQSTPSTLVLNYDSQRGGVGVGVANGHKLLTGVSHLAD
NNERVSSV

#gi|195160605|ref|XP_002021165.1|_GL24963_Drosophila_persimilis
METDWDTEARLPIPSDAASGAENDLASNWSTLSNFTRLFNAAAPDLVNYTLNMLDLSVSM
ATEVANVSISSTTPVPAYAITNSSSMAHTDGLHESGSPMAEQVPEHMMDHAPQLSRSGLL
KVYVLAVMALFSLLGNLLTIWNIYKTRISRRNSRHTWSAIYSLMFHLSIADVLVTWFCII
GEAAWCYTVQWLANELTCKLVKLFQMFSLYLSTYVLVLIGVDRWIAVKYPMKSLNMAKRC
HRLLGGTYILSLVLSLPQFFIFHVARGPFVEEFYQCVTHGFYTADWQEQMYATFTLVFTF
LLPLCILFGTYMSTFRTISSSEKMFQGSKLANYSTNKLPTQTNRQRLIHKAKMKSLRISV
VIIIAFLICWTPYYVMMIMFMFLNPDKRLGDDLQDAIFFFGMSNSLVNPLIYGAFHLCPG
KGSKSSGGGNNNAYSLNRGDSQRTPSMLTAVTQVDGTGGSSRQMRAFRQQSYYRSSSNGC
AGPPFKEQVGLLHVGPGSSSAGPGSVSGSNSATPQLMRKGSSVRSPHPHLAILARHPSCL
REQEQQRLLLQTTPSTVVLNYDAQRGGVGVGVASGAHLTALLDNNERVSSV

#gi|195338720|ref|XP_002035972.1|_GM13935_Drosophila_sechellia
MHLAIADLMVTLLLMPMEIVWAWTVQWLSTDLMCRLMSFFRVFGLYLSSYVMVCISLDRY
FAILKPLKRSYNRGRIMLACAWLGSVVCSIPQAFLFHLEEHPAVTGYFQCVIFNSFRSDF
DEKLYQAASMCSMYAFPLIMFIYCYGAIYLEIYRKSQRVLKDVIAERFRRSNDDVLSRAK
KRTLKMTITIVIVFIICWTPYYTISMWYWLDKHSADKINPLVRKALFIFASTNSCMNPLV
YGLYNIRGRVNNNNPSVNNRHTSLSNRLDSSNQLMQKQLTNNSLLNGRGQVMAAAVSATT
KLANVVGLKGTANGNGSAAVAVTVPPTPPLTVSIAPLATDDEANDDSCLSAVTIRSQDQS
PIRQKCDDSIELTSVVK

#gi|195438288|ref|XP_002067069.1|_GK24218_Drosophila_willistoni

MAQSGEVNEEIYDHRILKDWSNVNKTNGTMHLSKDMIFNDGHRLSITVYSILFVISTIGN
TTVLYLLTKRRLRCPLRIDIMLMHLAIADLMVTFLLMPLEIAWAWTVQWRSTDLMCRLMS
FFRVFGLYLSSFVMVCISLDRYYAILKPLQRSYNRGRIMLACAWLGSAVCSIPQAFLFHL
EEHPTVKGYFQCVTFHSFVSDFDNWLYQIATMCAMYAFPLIVFIYCYGAIYVEIYRKSKR
VLKDVVAERFRRSNDDVLSRAKKRTLKMTISIVIVFIICWTPYYTICMWYSWDKSSVGQV
NSLVRKALYIFASTNSCMNPLVYGLYNIRGRMNNNNNSVNNRHTSLSNRLDSSNQLLQKP
VNNALPNGHGNVMAAAVAATTKLARVVRLKSNSTANGAAGDSPHSQHPTAVNGGATAPLA
SECDDDANDDSTVSVVTIKYQAAEKEKPNCGDSMELTKMDKS

#gi|198476585|ref|XP_001357404.2|_GA10920_Drosophila_pseudoobscura_pseudoobscura
MAHSGEINEVVYDHRILKDWSNVNNTNGTMHLSKDMIFNDGHRLSITVYSILFVISTIGN
STVLYLLTKRRLRGPLRIDIMLMHLAIADLMVTLLLMPLEIAWAWTVQWRSTDLMCRLMS
FFRVFGLYLSSFVMVCISLDRYYAILKPLQRSYNRGRIMLACAWMGSVICSIPQAFLFHL
EEHPVVRGYFQCVTFHSFVSDFDNWLYQIATMCAMYAFPLIVFIYCYGAIYLEIYRKSQR
VLKDVIAERFRRSNDDVLSRAKKRTLKMTISIVIVFIICWTPYYTICMWYSLDKTSVDKV
NSLVRKALFIFASTNSCMNPLVYGLYNIRGRMNNNNNSVNNRHTSLSNRLDSSNQLMQKP
AAANNNSLANRHGTVMAAAVAATTKLAHVVGLKSNGSNGSAVDTTALSIASAPPLPLPQP
QPLPATAPLASDDADGDNDDSSVSVVTIRCQEPPRICHNCGDSIELVSAADK

#gi|20385582|gb|AAM21341.1|AF373862_1_Drm_corazonin_receptor_Drosophila_melanogas
ter
MEDEWGSFDRLPSVPSASMDLETENEVVSNWSTLANFTRLVAGAAPEIINYTLNMIDVGV
GMATDISNLSVSTTPLPAYAISNSSSLAHTNSRHEAPPMAEQVPEHVMDHAPQLSRSGLL
KVYVLAVMALFSLLGNLLTIWNIYKTRISRRNSRHTWSAIYSLMFHLSIADVLVTWFCII
GEAAWCYTVQWLANELTCKLVKLFQMFSLYLSTYVLVLIGVDRWIAVKYPMKSLNMAKRC
HRLLGGTYILSLVLSLPQFFIFHVARGPFVEEFYQCVTHGFYTADWQEQMYATFTLVFTF
LLPLCILFGTYMSTFRTISSSEKMFQGSKLANYSTAKLPTQTNRQRLIHKAKMKSLRISV
VIIIAFLICWTPYYVMMIMFMFLNPDKRLGDDLQDAIFFFGMSNSLVNPLIYGAFHLCPG
KGGKSSGGGGNNNAYSLNRGDSQRTPSMLTAVTQVDGTGGSSRQMRAFRQQSYYRSSSNG
TAGPGAAPFKEQVGLLHVGPGNGTPGGSVSSGETPQLIRKGSALLARQPSCLREQEHQQR
LLLHEKPSTLVLSYDSQRGGVGVGVASGLLDNNERVSSV

#gi|21358377|ref|NP_648571.1|_corazonin_receptor_isoform_A_Drosophila_melanogaste
r
MEDEWGSFDRLPSVPSASMDLETENEVVSNWSTLANFTRLVAGAAPEIVNYTLNMIDVGV
GMATDISNLSVSTTPLPAYAISNSSSLAHTNSRHEAPPMAEQVPEHVMDHAPQLSRSGLL
KVYVLAVMALFSLLGNLLTIWNIYKTRISRRNSRHTWSAIYSLMFHLSIADVLVTWFCII
GEAAWCYTVQWLANELTCKLVKLFQMFSLYLSTYVLVLIGVDRWIAVKYPMKSLNMAKRC
HRLLGGTYILSLVLSLPQFFIFHVARGPFVEEFYQCVTHGFYTADWQEQMYATFTLVFTF
LLPLCILFGTYMSTFRTISSSEKMFQGSKLANYSTAKLPTQTNRQRLIHKAKMKSLRISV
VIIIAFLICWTPYYVMMIMFMFLNPDKRLGDDLQDAIFFFGMSNSLVNPLIYGAFHLCPG
KGGKSSGGGGNNNAYSLNRGDSQRTPSMLTAVTQVDGTGGSSRQMRAFRQQSYYRSSSNG
TAGPGAAPFKEQVGLLHVGPGNGTPGGSVSSGATPQLIRKGSALLARQPSCLREQEHQQR
LLLHEKPSTLVLSYDSQRGGVGVGVASGLLDNNERVSSV

#gi|221139712|ref|NP_001137393.1|_corazonin_receptor_Apis_mellifera
MTILNNYTTYMLLACDNLTNFFNHTRDLTLDSRLWNTSELSHPFHSYNLKNITCLEQAPH
SNGNTLFKSLILTIMAVVSILANLATIYSIVRCRRRHHSWSAIYTLILQLAVADLFVSVF
CIGGEAMWNYTVEWIWGNVACKLFKFFQVFSLYLSTFVLVLIGVDRFFAIRYPMKGMNTA
DRCLKFIVAAWILSFVLSLPQIIIFRVVQGPFVEKFEQCVTYGFYTEPWQEQLYVSFGLF
SMFLLPLGILIATYVFTIITISRSERMFKVKLANNDICHVNGDVNRRKLMYRAKAKSLRI
SIVIVTAFIFWWTPYYTMMIIFMFSCPDKHVSDELQNIIFFFGMSNSLVNPLIYGAFHLW
PRKRRNFMHREISTTQRRLTPTSYGSSYRRDSRETRIPILPKN

#gi|222418560|ref|NP_001138451.1|_gonadotropin_releasing_hormone_receptor_2_Danio
_rerio
MNTTQLIEDLLQNSSCKHEAKHLNSNIAGDLPHPLHLPRFTAASQVRVTLTLVLCALSAC

CNLAVLYSANNNQRKRSHVRLIITNLAAADLLVTFVVMPTDAVWNITLQWRAGDLACRTL
MFLKLVAMYSCAFVTVVISLDRQAAILNPLSINKAMRRNKVLLGVAWTMSVVLSIPQVFV
FHVVEIDSPKQFVQCTTYGSFSSRWQETFYNMFTFTFLFLLPLFIMISCYTRILFEISKK
MTEDRLLSNKVQLRRSKNNIPKARMRTLKMTVVIVLSFMVCWTPYYMLGLWYWFSPAGLE
ETVSQSLSHILFIFGLLNACLDPIIYGLFTVPLCRGMRHRQREQNIVAFELENTNNSLMT
SIRTSTSPLTLKRLAVQQTSREKLEWDAEFVLKTGDDGTKNALYSSGVATVE

#gi|222418562|ref|NP_001138452.1|_gonadotropin_releasing_hormone_receptor_1_Danio_rerio
MSGNVSLSLISLLENSSLASSSSPQSPQWETPTFTTAARYRVVATLVLFVFAAVSNLSVL
ISVTRGRGRHLASHLRLLIASLASADLVMTFVVMPLDAIWNITVQWYAGDAMCKLLCFLK
LFAMHSAAFILVVVSLDRHHAILHPLEALDAGRRNRRVLLAAWILSVLLASPQLFIYRAL
KAEGVDFVQCATHGSFRHRWQETAYNMFHFVTLYVFPLLVMSFCYTRILVEINRQMPRGK
GKGGEPCLRRSGADMIPKARMKTLKMTIIIVASFVVCWTPYYLLGIWYWFQPRMLQVTPV
YAHHALFVFGNLNTCCDPVIYGFFTPSFRADITSCFSRRNQNCSPKSLDRLSARRGGASG
EAESDPGSGDQPSGQTA

#gi|22324515|gb|AAM95605.1|AF329894_1_gonadotropin-releasing_hormone_receptor_2_Clarias_gariepinus
MPRNDSLFSPLELDTSLQDVSNSSLSPPLADWVAPTFTRAAQFRVGATLVLFLFAAVSNL
ALLISVCRGRGRRLASHLRPLIRSLAVADLMMTFIVMPLDMVWNVTVQWHGGDGLCKLLS
FLKLFAMQASAFILVVISLDRQHAILHPLDTLNAHRRNKRMLVLAWSLSLLLASPQLFIF
RAIKAEKVDFTQCVTHGSFPERWQETVYNMFHFVTLYVIPLLVMSCCYTRILFEINRQEH
KNKAGESCLRRSRTDMIPKARMKTLKMTIIIVLSFIICWTPYYLLGIWYWFQPEMLKVTP
EYVHHLLFVFGNLNTCCDPVIYGLYTPSFRADLARCWGCRSTSDSPRSLERLSARQGPHS
AEQESDPASVKGAEG

#gi|224063351|ref|XP_002190060.1|_PREDICTED:_gonadotropin-releasing_hormone_II_receptor-like_Taeniopygia_guttata
MAWPGNAGQDALDAGRGHPDPGPAVGNSSAEPPSVTPPEWGCAWSPPEEKGEEPLRLPTF
SPAAQVRVAVTFALFALSAGCNLAVLRAVGGRGSGRRPHIRLLLRHLAAADLLVTVLVMP
LDAVWNITLQWRAGDLACRLLMYLRLLAMYASAFVTVVISLDRQAAILRPLAIARARARN
RAMLHVAWALSAGLAVPQLFLFHTITLRPPHNFTQCTTRGSFPRAWHETLYNMVGFACLF
LLPLLIMVCCYARILLEISRRMGSGLFSSQDASLRCSRNNIPRARLRMLRMSLVIVSSFI
LCWTPYYLLGLWHWFCPRAMEKRISPALTHILFIFGLFNACLDPITYGLFTIPLRGGWGC
PCRHGPLAQPLSPLLRSFRCSASSLAPHQPAHGMHLSRALPLDPSHIYPSR

#gi|225030853|gb|ACN79527.1|_gonadotropin-releasing_hormone_receptor_4_Branchiostoma_floridae
MSPSQSPRTENDSAFFREDVFLKDFLNESSDPELINETSRNNKTMDRDLNLPEFTTWTLT
KIVTICILFVIAAIGNLFMARATLRLRRRSGIYLLLLHLSVGELLVTCITMPSEAIWAYT
VSWWAGDTMCRIVKYGQMLGLYLSTYITVCISLDRCVAIAFPLKKGQAPERARSMVIVSW
ALSPIFCIPQAVIFHVEVHQYVPSFHQCVTYNFYSAEWQEDLYNMLVFVVMYPAPMVIMV
ACYVCIFVSLFRHWRGTNNLETGNKTGQRERLFSKAKVRTLQMAAGILTTFFVCWTPFYC
VMMWHLFFQHEYPINQIIFDVLYPFGVSNACVNPVVYGKSVVTRKPGKSFLVNWYQAFLE
PEEYARKLDSTKAAACTRLSSLRRSEQRDRMNSLTPTVYYEVSRRNSSRIVTQDQLSGSR
V

#gi|22901732|gb|AAN10045.1|_putative_corazonin_receptor_Drosophila_melanogaster
MEDEWGSFDRLPSVPSASMDLETENEVVSNWSTLANFTRLVAGAAPEIVNYTLNMIDVGV
GMATDISNLSVSTTPLPAYAISNSSSLAHTNSRHEAPPMAEQVPEHVMDHAPQLSRSGLL
KVYVLAVMALFSLLGNLLTIWNIYKTRISRRNSRHTWSAIYSLMFHLSIADVLVTWFCII
GEAAWCYTVQWLANELTCKLVKLFQMFSLYLSTYVLVLIGVDRWIAVKYPMKSLNMAKRC
HRLLGGTYILSLVLSLPQFFIFHVARGPFVEEFYQCVTHGFYTADWQEQMYATFTLVFTF
LLPLCILFGTYMSTFRTISSSEKMFQGSKLANYSTAKLPTQTNRQRLIHKAKMKSLRISV
VIIIAFLICWTPYYVMMIMFMFLNPDKRLGDDLQDAIFFFGMSNSLVNPLIYGAFHLCPG
KGGKSSGGGGNNNAYSLNRGDSQRTPSILTAVTQVDGTGGSSRQMRAFRQQSYYRSSSNG

TAGPGAAPFKEQVGLLHVGPGNGTPGGSVSSGATPQLIRKGSALLARQPSCLREQEHQQR
LLLHEKPSTLVVSYDSQRGGVGVGVASGLLDNNERVSSV

#gi|22901736|gb|AAN10047.1|_putative_AKH_receptor_Drosophila_melanogaster
MAKVAEENDHRDLSNWSNVNDTNGTIHLTKDMVFNDGHRLSITVYSILFVISTIGNSTVL
YLLTKRRLRGPLRIDIMLMHLAIADLMVTLLLMPMEIVWAWTVQWLSTDLMCRLMSFFRV
FGLYLSSYVMVCISLDRYFAILKPLKRSYNRGRIMLACAWLGSVVCSIPQAFLFHLEEHP
AVTGYFQCVIFNSFRSDFDEKLYQAASMCSMYAFPLIMFIYCYGAIYLEIYRKSQRVLKD
VIAERFRRSNDDVLSRAKKRTLKMTITIVIVFIICWTPYYTISMWYWLDKHSAGKINPLL
RKALFIFASTNSCMNPLVYGLYNIRGRMNNNNPSVNNRHTSLSNRLDSSNQLMQKQLTNN
SLLNGRGQVMAAAVSATTKLANVVSLKGTANGNGSAAAAGTVPITPPLTVTIAPLATDDE
ANDDSCLSAVTIRCQDQSPIRQKCGESIELTSVVK

#gi|258590757|emb|CAY77164.1|_adipokinetic_hormone_receptor_Aedes_aegypti
MSNAILKTERGEVLNYSHSYGENYNNDVNTMPYVLSSSTSKTGVFDNETWYGTNSSNWNE
PLPIDMQFNDGHKLQIVVYSVLMVISAIGNITVLALLIKRRLKSHSRIDMMLTRLAIADL
LVTFLMMPLEIGWAATVQWRAGDIMCRVMAFFRTFGLHLSSFVLVCISVDRYYAVLQPLN
LSKSRGKIMILIAWAMATLCSAPQPFIFHVEIHPNHTWYEQCVTYNTFSNDNYHTVYNIL
VMMFMYALPLLTIICSYASIYMEIFRHSRMPNSEGFRRSSIDALSRAKRRTLKMTITIVM
AFVICWAPYYVMSVWYWLDQKSAENVDQRVQKGLFLFACTNSCMNPIVYGIYNVKLRKKK
KPDGVKSGQSSVILRNSAKYTRHSESIRSSSGKIRGYRDLDIPSCAIKPV

-
#gi|261599705|gb|ACX85731.1|_isotocin_receptor_Cyprinodon_nevadensis_amargosae
MDELLSAQDAWLQNFSYCNYSHLNKTHPGNNVVNPLKRNEEVAKVEVTVLVLVLLLALTG
NLCVLWAIHTTKHSKSRMYYFMKHLSIADLVVAVFQVLPQLIWDITFRFYGPDIICRLVK
YLQVVGMFASTYMLVLMSIDRCLAVCQPLRSVHKGKDRFCVIGSWILSLVFSTPQAYIFS
LREVGNGVYDCWGDFVQPWGAKAYITWMSLSIYIIPVAILSICYGLICFKIWQNINMKTK
REHFLALTPRPSKSAHPLSRVSSVRLISKAKIRTVKMTFVVVIAYIVCWTPFFFVQMWSA
WDPAAPREDMAFIISMLLASLNSCCNPWIYMLFAGHLFHDLIKSFCCCCRNYVADSSCHV
NQECRHKRGSSTFDIKNGSSMRSLTHTSSLGGPTH

#gi|268370124|ref|NP_001161243.1|_adipokinetic_hormone_receptor_Nasonia_vitripenn
is
MTTAPVNATTVASLDYDDLPIDMRFNAGHVVSIVTYSILMIISAVGNITVLALLLRRRGN
AARTRINTMLIHLAIADLLVTFLMMPLEIGWAATVSWKAGDAMCRIMSFFRMFGLYLSSF
ILICISVDRYHAVLRPLQMIDIDRRGRFMIAGSWICSALCSAPQMVVFHVEAHPTFTWYE
QCITFNTFPSFTHELTYSLFGMVMMYWFPLIVIIYTYTSILAEMYRRSKDTTSDRIRRSS
LGFLGRARVRTLKMTIIIVLVFFICWTPYYVMSLWYWIDSVTATKVDLRIQKALFLFACT
NSCMNPIVYGAFNIRKGNKVTRNWDIHTLK

#gi|28380052|sp|Q95MH6.1|GNRR2_CHLAE_RecName:_FullGonadotropin-
releasing_hormone_II_receptor_ShortGnRH_II_receptor_ShortGnRH-II-
R_AltName:_FullType_II_GnRH_receptor
MSAGNGTPWGSAVGEEAWAGSGVAVEGSELPTFSTAAKVRVGVTIVLFVSSAGGNLAVLW
SVTRPQPSQLRPSPVRTLFAHLAAADLLVTFVVMPLDATWNITVQWLAGDIACRTLMFLK
LMAMYSAAFLPVVIGLDRQAAVLNPLGSRSGVRKLLGAAWGLSFLLALPQLFLFHTVHRA
GPVPFTQCVTKGSFKARWQETTYNLFTFCCLFLLPLIAMAICYSRIVLSVSSPQTRKGSH
APAGEFALRRSFDNRPRVCLRALRLALLILLTFILCWTPYYLLGLWYWFSPTMLTEVPPS
LSHILFLFGLLNAPLDPLLYGAFTFGCRRGHQELSIDSSKEGSGRMLQQEIHALRQQEVQ
KTVTSRSAGETKGISITSI

#gi|283806645|ref|NP_001164571.1|_ACP_receptor_Nasonia_vitripennis
MDQLQGSRMQLLQDFNNDFRNDSFYSHDFRDNMSMAVPTMPPSMTFTRRTLTIIIVYCIC
FLVAAIGNLTVFLTLWRGRYRKSRISLMICHLSIADLLVAFFTIPIEIGWRLTVQWIAGN
YACKLFLFLRAFGLYLSNNILICVSLDRYFAVLYPLRVNDARRRGKFMLSVAWFFSVLYA
IPQSIVFHVENHPHHKNFTQCVTFGAFPSDLVENTYNVFCVLTMYFIPLAIICWVYLKIL

CEISSKSRDNKPVVIKAGSNGTLESSNSNQGSRMRLRRSDMSSIERARSRTLKMTIIIVV
AFIFCWTPYITMNLWYVIDKKSAKEVNEMVQESLFIMAVGNSCANPLVYGSYAIDLKKEC
FRCFLPCTTTKSNADVNLIQRSLGSKFQKPEMKSPGVSKQIVHSVCQAVHGFFKAGSGQT
KSTNVCGKVLVPISPRLSVSTTSKGVVVEKLPLHTIKPIEFKENLIKSPSCSQPPLLQLN
SSMNFSGIMEHQTA

#gi|28381363|sp|P49922.2|GNRHR_PIG_RecName:_FullGonadotropin-releasing_hormone_receptor_ShortGnRH_receptor_ShortGnRH-R_AltName:_FullLuteinizing_hormone-releasing_hormone_receptor_ShortLHRH
MANSASPEQNQNHCSAINSSILLTQGNLPTLTLSGKIRVTVTFFLFLLSTAFNASFLLKL
QKWTQRKEKGKKLSRMKVLLKHLTLANLLETLIVMPLDGMWNITVQWYAGEFLCKVLSYL
KLFSMYAPAFMMVVISLDRSLAITRPLAVKSNSRLGRFMIGLAWLLSSIFAGPQLYIFRM
IHLADSSGQTEGFSQCVTHGSFPQWWHQAFYNFFTFSCLFIIPLLIMLICNAKIMFTLTR
VLQQDPHNLQLNQSKNNIPRARLRTLKMTVAFAASFIVCWTPYYVLGIWYWFDPEMVNRV
SDPVNHFFFLFAFLNPCFDPLIYGYFSL

#gi|28822121|gb|AAO50197.1|_gonadotropin-releasing_hormone_receptor_3_Rana_dybowskii
MNASDQPMEDGEAALPGLCAFKGFNLSCVHTNGFERPHGPNITFLNEDHFVLPTFSTAAK
IRVAITCVLFISSACFNMATLWTITYKYRKKSHIRILIINLVAADLLITFVVMPLDAVWN
VTIQWYAGDVACRILMFLKLVAMYSSAFVTVVISLDRHAAILNPLGIGDAKKKNRTMLSI
AWTLSLLLATPQLFVFHTVSRSHPVHLVQCATLGSFKAHWLETLYNMFTFCCLFLLPLLI
MVFCYGRILLEISRKMKKAEASSREVNLRRSYNNIPRARMRTFKMSLVIVLTFIVCWTPY
YLLGIWYWFSPEMLTSRRVPPSLSHILFLFGLFNTCLDPIIYGLFTIHFRREIRRVCRCA
TQGKDADATSLGTGSFRVSTAAVPLKRSAGVSGGSCKFDLEVTGVGLHSGKCEHCKRQIV
ESFM

#gi|28830316|gb|AAO50198.2|_gonadotropin-releasing_hormone_receptor_1_Rana_dybowskii
MNISKEVSIKGCSNAQWLSSSCDLDVNMTSTNETLTRFQLPTFSSAAKARVIITFVIFTL
SATCNLAALWAASRTSRKKRSHVRILILNLTTADLLVTFIVMPLDAVWNITVQWHAGDIA
CRILMFLKLLSMYSCAFVTVVISVDRQSAILNPLAINDAKKKNKIMLSVAWLMSAVLSLP
QLFLFHTVTITEPHNFTQCTARGSFQQHWQKTVYNMVSFVCLFLLPLLIMICCYSRILLE
ISKRMSKGTLSSKEVYLRCSKNNIPKARTRTLKMSVVIVSSFIICWTPYLLLGLWYWFFP
EIMEEKVSQSTTHILFIFGLVNACLDPITYGLFTIHFRKSLQRYCGGRRTSDADTSSSVT
GSFRCSMSSFRAKKMIVLNQELQVLQSCNGNFNNPEFRLNGLGTSCL

#gi|291225154|ref|XP_002732566.1|_PREDICTED:_oxytocin_receptor-like_Saccoglossus_kowalevskii
MTSKLNEMKYAAVMGAFINTTVYNNTVNNSTYDIPEKHQSVLSMYQTEQLILLWFLFAFV
VIGNAIVLVSVCLVRHKKSRMNFFIMNLAIADLSVGLLNILPDIIHRYTREFYGGEIVCK
LVKYVQAIVVYGSTYQLVALSIDRYDAIVHPMNFSGNKRSMIMVISMWVVAFILAVPSPV
FFEETVLENGEVQCWIELPQTWVWKPYSVILAFLLFFIPLVIVTFCYSVIIYTIWRKSKM
MVPAKRRIFNEKNGDSRGLIPKAKIKTIKMTLCIVLSFIVCWSPFTLWFILEIYGHIPKN
DLTMTIHIIVQNLPSLNSATNPAIYGLFSTNICKELR

#gi|294489280|ref|NP_001170921.1|_gonadotropin_releasing_hormone_receptor_3_Danio_rerio
MSGNWSQYNASLLPVWTAPSFTPAAQARVAATALLFVFAAGSNLALLVSVCRSRRLASHL
RPLILSLAAADLLMTFVVMPLDMVWNVTVQWYAGDVVCKLLCFLKLFAMQTSAFILVGIS
LDRHQAILRPLDTLTAPQRNRRRMLTAWSLSALIASPQLFIFHTVKAKSVDFTQCVTHGS
FSERWHETAYNMFHFVTLYVIPLLVMSCCYTCILIEINRQLHNSNKGDSLRRSGTDMIPK
ARMKTLKMTLIIVLSFVVCWTPYYLLGIWYWFQPEMLTVTPEYVHHLLFVFGNLNSCCDP
VIYGLYTPSFRSDLIRFCCCRHHKNTPRPQQPTRHT

#gi|301068501|gb|ADK55068.1|_adipokinetic_hormone_receptor_Polyrhachis_vicina
MQLTDSKESLKCNDTMELPDELRFNSGHLVTIVAYSILMVMSAAGNITVLIMTIIKKRKS

KSRIHTLIMHLSIADPLVTFLMMPLEIGWSITVSWEAGDAMCRIMAFFRMFGLYLSSFVI
VCISIDRYYAVMRPLQILDVYRRGKIMLMLAWVGSVLCSLPQMLVFHLETYPNYTCFTQC
VTFNFFPSHLQEISYNLFSMLIMYWFPLIVIFYTYSSIFMEICRRSQEKSEDKIRRSSSG
FLSRARVRTLKMTITIIAVFIICWTPYYVMSVWYWFDRSSAQKVDERIQRALFFFACTNS
SMNPIIYGIFNIRKKNKTPIRTTTIETRLTPLSLSVKLLD

#gi|30144690|gb|AAP15162.1|_GnRH_receptor_1_Pelophylax_ridibundus
MNISKEVSIKGCNNAQWLSSSCDLDVNMTSTNGTHTHFQLPTFSPAAKARAIITFVIFTL
SATCNLAALWSAARTSRKKRSHVRILILNLTTADLLVTFIVMPLDAIWNITVQWHAGDIA
CRILMFLKLLSMYSCAFVTVVISVDRQSAILNPLAINDAKKKNKIMLSVAWLMNAVLSLP
QLFLFHTVTITEPHNFTQCTTRGSFQQHWQETVYNMVSFVCLFLLPLLIMIFCYSRILLE
ISKRMSKGTLSSKEVYLRCSKNNIPKARMRTLKVSIVIVSSFIICWTPYFFLGLWYWFYP
EIMKEKIPQSTTHILFIFGLVNACLDPITYGLFTIHFRKSLQRYCGGGRTSDADTSSSVT
GSFRCSMSSFRAKKMIVLNQELQVLQSCNGNFNNPEFRLNGLGTSCL

#gi|30144694|gb|AAP15164.1|_GnRH_receptor_3_Pelophylax_ridibundus
MNASDQPMEDGEAALPGLCAFKGFNLSCVHTNGFEKPHGPNITFLNEDHFVLPTFSTAAK
IRVAITCVLFISSACFNMATLWTITYKYRKKSHIRILIINLVAADLLITFVVMPLDAVWN
VTIQWYAGDVACRILMFLKLVAMYSSAFVTVVISLDRHAAILNPLGIGDAKKKNKTMLSI
AWTLSLLLATPQLFVFHTVSRSQPVHFVQCATLGSFKAHWLETLYNMFTFCCFFLLPLLI
MVFCYGRILLEISRKMKKAEASSREVNLRRSYNNIPRARMRTFKMSLVIVLTFIVCWTPY
YLLGIWYWFSPEMLTSRRVPPSLSHIPFLFGLFNTCLDPIIYGLFTIHFRREIRRVCRCA
TQGKDADATSLGTGSFRVSTAAVPLKRSAGVSGGSCKFDLEVTGVGLHSGKCEHCKRQIV
ESFM

#gi|302316214|gb|ADL14591.1|_gonadotropin_releasing_hormone_receptor_2_Petromyzon
_marinus
MNSSSPTWTVTWQISPSSDLPLSFTTTTSSPSPSSSSPATTVPRLQSPPVSLPSQGIVIP
SFTTASQVRVAVTFSLCALSLCCNAAVLWSASRDRRPRRSHVRILLVNLALSDLLTACVV
MPLDAAWNITVGWRGGDAACRLLMFLKLVAMYSCAFVTVVISLDRHAAVTDPLGVSRAKR
RNKTLLCAAWSLSALLALPQLFLFHVARAPSAESFTQCVTHGSFPRRWHEVCYLMFTFSC
LYLLPLVVMLASYGHILLQISRRVKNVELREMRACNHGPSTLSRARARTLCMTALIVSSF
IVCWTPYYLLGVWYCIRPAMASEDHIPEAVSHGLFLFGLLNACIDPVVYGFFSVPLGPGS
ACRRRCCCFRVVSTDRDAGSAAIHNHKHKGQRQNTVDSDPTSGHLAHLASWRSYLSSSVS
SAFVDFVRGHWHSRRPHGDARAAARVPSPTTVYVLSQLCDPHATQG

-
#gi|302316216|gb|ADL14592.1|_gonadotropin_releasing_hormone_receptor_3_Petromyzon
_marinus
MTLLAHACNFTSSSPPSFFSSAPGATPCAPGTSLVTSNPDADPRGVDPNMTTVPGSNLTG
PHPHPPPLLPTFSAAARVRVCVTLILLLASATLNGMVLRSACADRRTRSRRQRGSHVRLL
MLHLSAADLLFTLLVMPLDAAWNVTLQWRAGDAACRLLMFLKLFAMYASAFVTAVISLDR
YSAVVNPLAFGQAKRRSRVTLCLAWALSAVLALPQLVLFRVVQTSQPGPQFTQCATHGSF
PQRWQGSLYFMFTFACLFLLPLLIMVFCYARILLEIVRRGRERDGVAHDAKGVTLRCSSN
NIPRARLRTVKMTAAIVGSFLVCWTPYYLVGIWYWFWPSALDQGTLPEYINHIVFLFGLL
NACLDPLVYGLFSGQWDGTLCYRYCCCCYCCCWWSSSSSTRRRRRSGTTTGATTSSRGTM
ASSELEPQPTLATASVDLSAPLQRQGCPPVQ

#gi|302634030|gb|ADL60118.1|_hypertrehalosemic_hormone_receptor_Blattella_germani
ca
MTTTELPREQQLTEDMTFGSIHKLCIATYCVLMTVSAIGNITVLVNILKRRRNLRFGNNY
MFMHLAIADLLVTFLMMPLEIGWNATVSWRAGDAACRVMSFFRIFGLYLSSFVIVCISLD
RCFAILRPMSNVVNVAKRSRVMLTTAWSLATVCSLPQVFIFHVQQHPVFTWYEQCLDFDM
FPTQLYQFWYRILNMVLVYGFPLLVIFISYACILTEIFRRYQLSSDENFRRSSLVFLNRA
KNRTLKMAIIIFVVFFICWTPYYVMCLWYWIDQQSAEKVDLRVRKGLFLFACTNSCMNPI
VYGYFNFRSGRGSGYGATRPGQQLQHHQITALSNNSTGVNSRRGSNCSSIYRDNSNQSMS
WNRRSSHETEMHANNNRDENHLHPNSAANHNLRTTVSTVSEVPEAR

#gi|307181813|gb|EFN69256.1|_Gonadotropin-
releasing_hormone_II_receptor_Camponotus_floridanus
MKLSTIINNSSDNSSSVNVIGLSTIFPNPCDNLTDFISNNGLVGQSAEYLSFENVAIFHV
AQGPFIEEFPQCVTHGFYTEPWQEQLYASFSLFFMFLLPLVILITTYVSTVITISRSQKM
FKAEPTTGTYIRNSDLNRRRLMHRAKTKSLRISVVIVAAFLIWWTPYYTMMIIFLFLDPD
EHLSEELQSGIFFFGMSNSLVNPLIYGAFHLWPQKQRQGSYQR

#gi|307206489|gb|EFN84515.1|_Gonadotropin-
releasing_hormone_II_receptor_Harpegnathos_saltator
MNVDSSSSSDIINNTTIAIALPVVFEEPCNNLTNFVSHALGLDLKLNPRSRDNLSEIDPH
ENITCLEHAPKLTDSVYLKVIILAVISVLSLLCNLATIYSITKNRRKQRGSTIYTLLLHL
TIADLLVTVFCLAGEAIWSYNVAWLWGNAACKFFKFLQMFSLYLSTFVLVLIGVDRFVAV
RYPMKGLSMNQKCSRFVLFAWILSCILALPQIAIFHVAQGPFVEVFTQCVTHGFYTEAWQ
EQLYASFSLFFMFLLPLAILITTYVSTVITLSRSQRMFKGELTNNGMYTRSSDLNRRRLM
HRAKTKSLRISVVIVAAFLIWWTPYYTMMIIFLFLNPDEHLSDELQSGIFFFGMSNSLVN
PLIYGAFHLWPQKQQKDSYHRSKQATKQHDKQQNSIKKLRNTQTPLLLYQDSDRKVNTQQ
YQI

#gi|31242249|ref|XP_321555.1|_AGAP001558-A_Anopheles_gambiae_str._PEST
MPPSFNLSTVTYELIWGNVSAALENFTASMAGAFPFEDLHDPASSFARNASFTLGMGLAN
VIQLQQQQQQQQQQLHHSPHQYHQQVQHPQPPSTPFANVSTGQNESLANLLLHPGVHQLS
SGLVTLVGDIMAQSGATILPREECDRLNISYAFENGTALEIPGLSCYEHAPTLSKSGVIR
VIVLSAMAIVSLLGNVATMWNIQKNRKSRRVTRHNWSAIYSLIFHLSIADVLVTGFCLIG
EAAWYYTVDWVAGNLFCKLFKLCQMFSLYLSTYVLVLVGVDRWVAVKYPMKSLNTARRCH
RFLFVAYLLSFLLSTPQWMIFRVAKGPFVEDFYQCVTHGFYTDRWQEQLYTTFTLVFMFI
IPLLILIGTYLSTFMTISSSEKIFRIDTSAVDRTTYYRRSDTNRQRLIHKAKMKSLRISV
VIVVAFVVCWTPYYIMMLIFMFLNPTERFGEDLQSGIFFFGMSNSLINPLIYGAFHLVPI
RQRRNQYNQHVREGSVYFQRSSTFNHHNGHQRNLNPHIKFSNSHSNLPEEISLMSLEKDL
RSLDENVNQIHQKGGGGGGGGGGGGGGVGGGIGLSLGGAAGVDGSRRIKRRSFASKFLSF
SRLLQRNHPTKL

#gi|313661378|ref|NP_001186298.1|_oxytocin_receptor_like_Danio_rerio
MESLLKDVILWPINDSWANSSRGNDSRGLNQTVNPLKRNEEVAKVEVTVLVLILLLALAG
NLCVLVAIQTSKHGQSRMYYFMKHLSIADLVVAVFQVLPQLIWDITFRFYGPDFLCRLVK
YLQVVGMFASTYMLVLMSIDRCLAIWQPLRSLRRRKDRFYVLASWIISLLFSLPQVYIFS
LREVGDGVFDCWGDFVQPWGAKAYVTWISLTIYIIPVAILSVCYGLISYKIWQNFRLKTR
RDQCLSLTPRPTKGAALSRVSSVKLISKAKIRTVKMTFVIVMAYIICWTPFFFVQMWSAW
DPMAPREAMAFIIAMLLASLNSCCNPWIYMFFAGHLFRDLMQRCLTVSHCGCKRHWRNKN
HSGTGAMRSSSSQKSVTQSSTT

#gi|313661382|ref|NP_001186299.1|_oxytocin_receptor_Danio_rerio
MEDIFKDQDFWSFNESSRNATNETYGVNQTVNPLKRNEEVAKVEVTVLALVLFLALAGNL
CVLIAIHTAKHSQSRMYYFMKHLSIADLVVAVFQVLPQLIWDITFRFYGPDILCRLVKYL
QTVGMFASTYMLVLMSIDRCMAICQPLRSLHKRKDRCYVICSWALSLLFSIPQVYIFSLR
EVGSGVYDCWGDFVQPWGAKAYITWISLTIYIIPVTILSVCYGLISFKIWQNFKRKTKRD
QCITLTPKASKGNALARVSSVKLISKAKITTVKMTFVIVLAYIVCWTPFFSVQMWSAWDP
EAPREAMPFIISMLLASLNSCCNPWIYMFFAGHLFHDLKQNLFCCSTLYLKSSQCRYDPE
QDSRKSNSSTYVIKSTSSQRSITQTSIT

#gi|320543284|ref|NP_996297.3|_crustacean_cardioactive_peptide_receptor_Drosophil
a_melanogaster
MLHLRLFDSSLYYTLASASESSGLASSTSTERSFNGTQGAGGVAAGGESLTPTDVAAVNL
TYFTPAISHVMLAPTTIATTTASATMVQIQTTAAPSHDLETGGNSTSSDPGEFDNLNSFY
FYETEQFAVLWILFTVIVLGNSAVLFVMFINKNRKSRMNYFIKQLALADLCVGLLNVLTD
IIWRITISWRAGNLACKAIRFSQVCVTYSSTYVLVAMSIDRYDAITHPMNFSKSWKRARH
LVAGAWLISALFSLPILVLYEEKLIQGHPQCWIELGSPIAWQVYMSLVSATLFAIPALII

SACYAIIVKTIWAKGSIFVPTERAGFGAAPARRASSRGIIPRAKVKTVKMTLTIVFVFII
CWSPYIIFDLLQVFGQIPHSQTNIAIATFIQSLAPLNSAANPLIYCLFSSQVFRTLSRFP
PFKWFTCCCKSYRNNSQQNRCHTVGRRLHNSCDSMRTLTTSLTVSRRSTNKTNARVVICE
RPTKVVTVPAMSEV

#gi|321470703|gb|EFX81678.1|_hypothetical_protein_DAPPUDRAFT_317355_Daphnia_pulex
MGDDDSCLERTWFNSDQLAGELNLCPSPSNSTEDDFNTFYFYQTEQLAFLWILLILIVVG
NMAVLVALSMSSARKSRMNYFIKHLAIADLSVGVISVLTDIVWKITVAWHAGNIACKVIR
FSQVLVTYSSTYVLVALSIDRYDAICHPMNFSRGWRRARILVSVAWILSAIFSSPMLVLY
EQGLVQGQVQCWIDFTAPWQWQLYLTLVAVSLLVLPALLIFACYIVIVRTIWVQSAIFLG
INRAGGSAGTAMLDEDQESRRASSRGIIPRAKIKTVKMTFVIVFVFILCWAPYIVFDLLQ
VYGHIPKSKTMIAVATFIQSLAPLNSAANPLIYCLFSTHLCRNLR

#gi|327283769|ref|XP_003226613.1|_PREDICTED:_gonadotropin-
releasing_hormone_receptor_Anolis_carolinensis
MTDGEPPMNALHGCSLPDENSSLSACPKEAWLEPAFTLAARTRVVVTSCFFMVAACSNSV
VLYSVTRKRRKSHVQLLILSLTAADLLVTTIVMPLDAVWNITIQWYAGDSLCKLLNFLKL
FAMYSAALVLVVISLDRHAAVLRPFSFANSNRRNRVMLSVAWVLSALLASPQLFLFHLYT
VPGVNFTQCVTHGSFKERWQETTYNMFTFTTLYVTPLSVMVICYIRILFEISKQLKINQG
LARGKDDHISKARMKTLTMTILIVASFIVCWTPYYLLGLWYWFQPDMIRQMPEYVNHFLF
LFGLLHTCTDPIVYGLYTPSFREDMKMCLKGLKLTLTHQEKSLAVIVELKNKEDREQGRP
RSSVSNGGTMHTAF

#gi|328716443|ref|XP_003245941.1|_PREDICTED:_gonadotropin-
releasing_hormone_receptor-like_isoform_X1_Acyrthosiphon_pisum
MEVMDSDANTVLHSVTKGVHVGPPPVWPGMDNVTNASMFDESNLPYDMKFNEGHVVAIVT
YSILMVVSAIGNITVLTIILKRRRKAGTRIHAMLMHLAIADLLVTFLMMPLEITWAWTVQ
WVLGDPLCRIMSFFRIFGLYLSSFILICISVDRYLAVLQPMRLYQMDRRGKLMIAVAWIA
SVVCSLPQTYIFHVERHPNATWYEQCVTYNAFSSKLHELAYLYFGMFMMYWLPLIVILFC
YASIIIEIYRRSRESICGQGTDNVRRLGFLGRAKSRTLKMTIIIVIVFVVCWTPYYIMAI
WYWTDHKSAQMVDQKVQHALFMFACTNSCMNPIVYGAFNIRTRRTLVTQGVGESVASVRV
VTWHKLTVRTKANRKSTIGNKAALFKSAQNGNTNDDGLVKENVTVTTTLCNDSDINDKNT

#gi|328776392|ref|XP_001122652.2|_PREDICTED:_cardioacceleratory_peptide_receptor_
isoform_X1_Apis_mellifera
MSVEEIVNRTDDNHTFQKEGSTWNFTDYVDTNVTQLDANATKIDSFYFYKTEQFTVLWLL
FAMIVVGNIAVLIGLQWGKRRKTRMDFFIKQLAFADLLVGLISVLTDIIWKTTVSWHAGN
VACKLIRFMQAVVTYSSTYVLVALSIDRYDAITRPMNFTGRWWRARALVISAWGLSALFS
APIIFLYEEKRVEGKTQCWIDLGSPTQWKIYMSLVSFTLFIAPTLIIGGCYAVIVATIWS
QGGALRQGPTRDTRRVSSRGLIPRAKVKTVKMTLVIVFVFILCWSPYIVFDLLQVFGYLP
KTQTVVAVATFIQSLTTLNSAANPIIYCLFSTSFCKTVRNAQVISWVSGWCATNPHHCFG
TGAPNSSTRTTVTTSLTAQSSRRSGHIAMLHSTSRKRVMVSLV

#gi|332016315|gb|EGI57228.1|_Gonadotropin-
releasing_hormone_II_receptor_Acromyrmex_echinatior
MGNVMQLVEAEDEESLGKCNGTIHAELPIELRFNDGHLVTIITYSILMVISAIGNISVLT
IIKKRKSKSRIQNLVMHLSIADLFVTFLMMPFEIGWASTVSWEAGDAMCRIMAFFRTFGL
HLSSFVIICISIDRYYAVMRPLQILDIHRRGKILLMFAWIGSVLCSMPQMLVFHLETHPN
HTCYTQCITFNIFPSYVHEVFYNLFSMVVMYWCPLIVIFYTYTSIFMEICRRSREKSEDR
IRRSSCSFLSRARVRTLKMTITIIAVFIICWSPYYVMSVWYWIDRSSALKVDKRIQRALF
FFACTNSSMNPIIYGIFNIRQRNKAPMRTATIETRVTPLSLSIKLLDCQ

#gi|33316064|gb|AAQ04564.1|AF439802_1_putative_gonadotropin_releasing-
hormone_receptor_Petromyzon_marinus
MKCALMEPINMNMTPRAAFLNNVTGPPNASHTGDEQLTNSSINSDIRLPATQFRVISTFA
LFIFAAISNLTVLCTISHNHRKTKSHVRILIVNLTTADLLITFIVMPLDAVWHITTQWYA
GEFACRLLMFLRLLAMYSSAFITVVISLDRHSAILNPLGIGKAKAKNKTMLSVAWVLSVL

LAVPQLFLFHVKSPKGNKNFVQCVTHGNFVEQWHHNLYYMFTFVFLFILPLFIMIFCYCR
ILLEISKRMREGSISSKEIRLRRSNNNIPKARMRTLKMSIAIVSSFVVCWTPYYVLGIWY
WFDRSIVSRKVVPHFVEEMSLTFGLLNACLDPVIYGVFAAHVRREVRRCCRWPRTAAHDR
DSSSTPVTGSFRYSASSVRSRRVPFACGEQPEATGAHPTPATRLLQRGCLVAGVPVNRAA
AGMAAGAKAFCDASGGGAGGGGGGGEGCTEKTLVCPESCI

#gi|334904105|gb|AEH25943.1|_adipokinetic_hormone_receptor_Glossina_morsitans_mor
sitans
MSETEVNGKIYDHRVLPHWSNVKNETNGSIHYSPDMIFNAGHRLSITGYSILFVLSLIGN
STVLYLLTKRRLSSRHTSRIDIMLMHLAIADLTVTLLLMPLEVAWSYTVEWKSTDFMCRL
MSFFRVFGLYLSSFVLVCISVDRYFAIIKPLKMSTNRGRLMLLVAWCTSIVCSLPQALLF
HLGEHPNVKNYYQCVMFDALSQFQSIFYNMTTMCAMYACPLITFIYCYGAIYLKIYRESK
RMTKGVERFRRSNDDVLCRAKKRTLKMTITIVIVFIICWTPYYIICMFYWFDYNTASRFS
PLLRKALYLFACTNSCMNPIVYGMFNIRGRRDNNANSSSNRNPSVYQRGDSSSQIPKSLL
NLSHSESNKIPPTRTSTNDKLVDSPLAKLNKADTLPMICIR

#gi|340730408|gb|AEK64844.1|_gonadotropin-
releasing_hormone_receptor_1_Pelophylax_esculentus
MNISKEVGLKGCSNAQWLSGSCDLDVNITSTNGTHTHFQLPTFSPAAKARVIITFVIFTL
SATCNLAALWAAARTSRKKRSHVRILILNLTTADLLVTFIVMPLDATWNITVQWHAGDIA
CRILMFLKLLAMYSCAFVTVVISLDRQSAILNPLAINDAKKKNKIMLSVAWLMSVVLSLP
QLFLFHSVTITEPQNFTQCTTMGSFPQHWQETVYNMVSFVCLFLLPLLIMIFCYSRILLE
ISKRMSKGTLSSKEVYLRCSKNNIPKARMRTLRMSIVIVSSFIICWTPYFFLGLWYWFYP
EIMKEKIPQSTTHILFIFGLVNACLDPITYGLFTIHFRKSLQRYCGGGRTSDPDTSSSVT
GSFRCSMSSFRAKKMIVLNQELQVLQSCNGNFNNPEFRLNGLGTSCL

#gi|344306673|ref|XP_003422010.1|_PREDICTED:_gonadotropin-
releasing_hormone_II_receptor_Loxodonta_africana
MSTGNGTPWGSAAGEEAWAGSGVEVEGSELPTFSAAAKVRVGVTIVLFVSSAGGNLAVLW
SVTRPQPSQARPSPVRRLFAHLAAADLLVTFVVMPLDATWNITVQWLAGDIACRTLMFLK
LMAMYAAAFLPVVIGLDRQAAVLHPFGPRSGGRKLLGAAWGFSFLLALPQLFLFHTVRRA
GPVSFTQCVTKGSFKARWQETTYNFFTFCCLFLLPLTAMAICYSRIVLSVSSPRKRKGNH
ASADEFALRRSLDNRPRVRLRALLLALLVLLTFILCWTPYYLLGLWYWFSPTMLTEVPSS
LSHILFLFGLLNAPLDPLLYGAFTLGCRRGHQEPGAASSREEGSGRMPLRQLEAHTNVGA
SRAGETKETFL

#gi|348500118|ref|XP_003437620.1|_PREDICTED:_gonadotropin-
releasing_hormone_II_receptor_Oreochromis_niloticus
MNGSSCCDPAAVMYQQRSGFDLNASCEWPDPHCNWTSVDGALQLPTFSTAAKIRVIVTFI
LCGISTFCNLAVLWAANGHKRKSHVRVLIINLTAADLLVTFIVMPVDAVWNITVQWLAGD
LACRFLMFLKLQAMYSCAFVTVVISLDRQSAILNPLGIAMVRKRNRVMLMVAWIMSALLS
IPQMFIFHNVTITYPANFTQCTTRGSFVTHWQETAYNMFTFCCLFLLPLVIMIICYTRIF
IQISKQMTKKNMPSNEPHLRCSKNNIPKARMRTLKMSIVIVICFIVCWTPYYLLGLWYWF
FPDDLEGKVSHSLTHILFIFGLFNACLDPIIYGLFTIRFQKGLRNCYHKAAVMSSLETNA
VIMESLKCTGSVLPSKRGMTSGEKDISSEHAEAKSTDNIV

#gi|348505814|ref|XP_003440455.1|_PREDICTED:_gonadotropin-
releasing_hormone_II_receptor-like_Oreochromis_niloticus
MFHQLADQTVNGSCQGPTLACNKSADGDALELPTFSTAAKVRVIITFALCAVSAVCNLAV
LWAASSGGRRKSHVRILIMNLTVADLLVTFIVMPVDAVWNITVQWQAGDVACRLLMFMKL
VAMYSCAFVTVVISLDRQSAILNPLGISEAKRKSKVMLAVAWTMSVILSLPQMFIFRNVT
ITVPEKFTQCTTHGSFVQRWQETLYNMFTFVCLFLLPLAIMIFCYTRILIEISSRMARNN
FLSRDVHLRRSHNNIPKARMRTLKMSIVIVTSFIICWTPYYLLGLWYWLFPEKMEKTVSH
SLTHMLFIFGLFNACLDPITYGLFTIHFHKGMRRCHQSSNARTELENNTRLVQMTRLSSR
RQIASDVHSASTEVVSEGNIMKRVSNPDMSISKI

#gi|348507897|ref|XP_003441492.1|_PREDICTED:_isotocin_receptor-like_Oreochromis_niloticus
MESISNESDIWQFNESWRNSSLINGTGGLNQTNPLKRNEEVARVEVTVLALVLFLALAGN
LCVLLAIHTTKHSQSRMYYFMKHLSIADLVVAIFQVLPQLIWDITFRFYGPDILCRLVKY
LQVVGMFASTYMLVLMSIDRCLAICQPLRSLHRRKDRLYVIFSWILSLLFSIPQMFIFSL
REVGSAGSGVYDCWGDFVKPWGAKAYITWISLTIYIIPVAILSICYGLISFKIWQNFKLK
TRREQCINLTPKTTKSNTLARVSSVKLISKAKITTVKMTFVIVVAYIVCWTPFFSVQMWS
AWDPAAPREAMPFIISMLLASLNSCCNPWIYMCFAGHLFQDLRQNLLCCSTRYLKSSQCH
CERDFNSSHKSNSSTFAIKSTSSQRSITQTSTT

#gi|348521866|ref|XP_003448447.1|_PREDICTED:_isotocin_receptor-like_isoform_X1_Oreochromis_niloticus
MEDLLREQYSWSHNLTWSSSSRENESHVGNATVNPLKRNEEVAKVEVTVLVLVLLLALTG
NLCVLWAIHTTKHSQSRMYYFMKHLSIADLVVAIFQVLPQLIWDITFRFYGPDLLCRLVK
YLQVVGMFASTYMLVLMSIDRCLAVCQPLRSVHRKKDRFCVIASWMLSLIFSSPQAYIFS
LREVGNGVYDCWGDFVQPWGAKAYITWMSLSIYIFPVAILSICYGLICFKIWENFNLKTR
REHFLALTPRPSKGAQPLSRVSSVRLISKAKIRTVKMTFVVVLAYIVCWTPFFFVQMWSA
WDPAAPREDMAFIIAMLLASLNSCCNPWIYMFFAGHLFHDLMQCFFCCCRRYLTECSCSC
DQQCRHKRSSSTYVNKNTNSQRSLSRTSSTVH

#gi|350400164|ref|XP_003485755.1|_PREDICTED:_gonadotropin-releasing_hormone_II_receptor-like_Bombus_impatiens
MENGIKTISSMELSNSRANISNEVELPIDMRFNEGHIVSIVFYSVLMIISAIGNTTVLVL
IMRRKRVSKSRIHTMLMHLAIADLLVTFLMMPFEIGWAVTVSWEAGDAMCRIMAFFRMFG
LYLSSFILVCISMDRYYAIIRPLQLWDVNRRGKIMLCIAWAGSVVCSVPQMFVFHLETHP
NITWYSQCVTFNAFPTYTHEITYSLFGMIMMYWFPLVVIIYTYANILLEICRRSKKSEDK
IRRSSMAFLTRAKIRTLKMTVIIVAVFFICWTPYYVMSLWYWIDRNSAYKVDQRIQKGLF
LFACTNSCMNPIVYGAFNIRDRNKTSVRPTTIETRVTPLSLSLKLLD

#gi|350401174|ref|XP_003486073.1|_PREDICTED:_gonadotropin-releasing_hormone_receptor-like_Bombus_impatiens
MENSVRLPIDYITYILSACDNLADFTDQSRGVNHTPDARPLHNISEFVQLISTGVLNNVT
CLNHAPQLTRVVVVKVIVLSVIAILSFVANVATIYSIAINRRKQHAWSAIYTLILHLTVA
DLLVTVFCMVGDAMWSYTVAWIFGNVACKLFKFAQVFSLYLSTFILVLIGVDRFFAIRYP
MKGMNTADRCLKFVVLAWILSFIFASPQIFIFHVVQGPFIEDFKQCVTYGFYTEPWQEQL
YGSFVLFFMFLLPLAILVATYVSTVITISRSERIFKLKLTNNIIRHVNGNTNRRKLMHRA
KAKSLRISIVIITAFIIWWTPYYTMMIIFMFLDPDKHLSKELQNVIFFFGMTNSLVNPLI
YGAFHLWPRRKRSIHRELSTVQRRLTPTSCEGNGKQEPRETRTPFIPKNN

#gi|351710988|gb|EHB13907.1|_Oxytocin_receptor_Heterocephalus_glaber
MEGALIANWSADAANESAAPGDPERNCTAQPPRRNEALARVEVAVLCLILFLALSGNACV
LLALRTTRHKHSRLFFFMKHLSIADLVVAVFQVLPQLLWDITFRFYGPDLLCRLVKYLQV
VGMFASTYLLLLMSLDRCLAICRPLRS?HIFSLREVAEGVFDCWAVFIQPWGPKAYVTWI
TLSVYIVPVIVLAACYSLISFKIWQNLRLKTAAEAAAQGPGDSDVGGAGRVALARVSSVK
LISKAKIRTVKMTFIIVLAFIVCWTPFFFVQMWSVWDADAPKEASAFIIAMLLASLNSCC
NPWIYMLFTGHLFHELVQRFLCCSPSSLKGSRSRETSVSKKSNSSTFALSQRSSSQRSCS
QPSTA

#gi|35215011|dbj|BAC77240.2|_GnRH_receptor_type1_Oreochromis_niloticus
MNASLCDPAAVMYQLVADHQLNTSCNCSSGLSNWTARGEAPQLPTFSAAAKARVIITFIL
CGISAFCNLAVLWAARVDGNENPHVRVLIVNLTMADLLVTFIVMPVDAVWNITVQWLAGD
FACRLLMFLKLQAMYSCAFVTVVISLDRQSAILNPLAINKARKRNRIMLMVAWAMSVVLS
VPQMFLFHNVTIIHPEDFTRCTTRGSFVTHWQGKKNNMFTFCCLFLLPLVIMITCYTRIF
CEISRRLKKDNLPSSEMHLRCSKNNIPRARMRTLKMSIVIVLSFIICWTPYYLLGLWYWF
FPGDLEGKVSHSLTHILFIFGLVNACLDPLIYGLFTIHFRKGLRRFYTGTTTAADLENNT
VITGSFSCAANSLSL

#gi|355469763|gb|AER93393.1|_gonadotropin-releasing_hormone_1_Pseudopleuronectes_americanus
MHHLPADHQLNASCNCSSPLSNWTAGGDTLQLPTFTTAAKVRVTITFILCATSAFCNLAV
LWAAHSDGKRKSHVRVLIINLTVADLLMTFIVMPVDAVWNITVQWLAGDLACRLLMFLKL
QAMYSCAFVTVVISLDRQSAILNPLAINEARKRNRVMLSVAWAMSAVLSVPQIFLFHNVT
IIHPEEFTQCTTRGSFVSHWHETAYNMFTFSCLFLLPLVIMITCYTRILCEISKRLYTDN
LSSNEVRLRCSKNNIPRARMRTLKMSIVIVLSFIICWTPYYLLGLWYWFFPDDLEGKVSQ
SLTHILFIFGLLSACLDPVIYGLFTIHFRKGLRRYFFKAPPASDLDNHTVITGSLTCAAS
ISPLKRELSLAVSQEKFITYRSNHSKEESTSPSGSFLTADNNTARDVNQFSSDSTV

#gi|355558351|gb|EHH15131.1|_Gonadotropin-releasing_hormone_II_receptor_Macaca_mulatta
MSAGNGTPWGSAAGEEAWAASGVAVEGSELPTFSAAAKVRVGVTIVLFVSSAGGNLAVLW
SVTRPQPSQLRPSPVRTLFAHLAAADLLVTFVVMPLDATWNITVQWLAEDIACRTLMFLK
LMAMYSAAFLPVVIGLDRQAAVLNPLGSRSGVRKLLGAAWGLSFLLALPQLFLFHTVHRA
GPVPFTQCVTKGSFKARWQETTYNLFTFRCLFLLPLTAMAICYSHIVLSVSSPQTRKGSH
APAGEFALCRSFDNCPRVRLWALRLALLILLTFILCWTPYYLLGLWYWFSPTMLTEVPPS
LSHILFLFGLLNAPLDPLLYGAFTLGCQRGHQELSIDSSNEGSGRMLQQEIHALRQQEVQ
KTVTSRSAGETKDISITSI

#gi|355745613|gb|EHH50238.1|_Gonadotropin-releasing_hormone_II_receptor_Macaca_fascicularis
MSAGNGTPWGSAAGEESWAASGVAVEGSELPTFSAAAKVRVGVTIVLFVSSAGGNLAVLW
SVTRPQPSQLRPSPVRTLFAHLAAADLLVTFVVMPLDATWNITVQWLAEDIACRTLMFLK
LMAMYSAAFLPVVIGLDRQAAVLNPFGSRSGERKLLGAAWGLSFLLALPQLFLFHTVHRA
GPVPFTQCVTKGSFKARWQETTYNLFTFRCLFLLPLTAMAICYSHIVLSVSSPQTRKGSH
APAGEFALCRSFDNCPRVRLRALRLALLILLTFILCWTPYYLLGLWYWFSPTMLTEVPPS
LSHILFLFDLLNAPLDPLLYGAFTLGCQRGHQELSIDSSNEGSGRMLQQEIHALRQQEVQ
KTVTSRSAGETKDISITSI

#gi|357616496|gb|EHJ70223.1|_adipokinetic_hormone_receptor_Danaus_plexippus
MHLAVADLMVTFLMMPLEIAWAGTVQWRAGDLMCRLMMFTRTFGLYLSSFVLICIAIDRY
YAILKPLNVTWEARVRRALTVAWVCAVFASLPQSFIFHLEEHPDVRGYYQCVSYGSLPTE
RHEFAYFLVNMALMYVAPLVSTLYCSSAALLEIIRRANTANDKMRRSGVGILGRARARTL
KMTVTIVLVFFACWSPYYCYCLWYWIDKDSVKSLDPAFQKAMWLFSCTNSCANPIVYGVF
NRNRWTWRSSHTGRCRNGMRRCSRFPHGDSMEISAATLAQARQRSDRHSRRDSGFTAKNG
SQKHINNNNNVNGIV

#gi|357624999|gb|EHJ75563.1|_neuropeptide_receptor_A21_Danaus_plexippus
MISVQRGKRGRRRTRPSWTAIYSLIFQLSIADLLVTIFCIAGEAAWSFTVQWLAGNAMCK
IFKFLQMFSLYLSTFILVLIGVDRWLAVKYPMKSMATASRSVRLVIIAWILSFVLSIPQV
VVFRVAKGPFLEDFYQCVTHGFYTERWQEQIYTTLSLVFMFILPLIIILTATYVSTVRTIA
KSEKVFQPEVKQENYLTPDMNRRRLIDRAKMKSLRMSVVIVTAFIIWWTPYYVMMVIFTF
LNPDRNLSEDLLSGIFFFGMSNSLVNPVIYGAFHLWPKKKRPRNSDRESGGPQASLLRRG
DHTSSVRLTTIRSLRSSAKSSNGQNMSLL

#gi|3659702|gb|AAC61523.1|_gonadotropin-releasing_hormone_receptor_Drosophila_melanogaster
MAKVAEENDHRDLSNWSNVNDTNGTIHLTKDMVFNDGHRLSITVYSILFVISTIGNSTVL
YLLTKRRLRGPLRIDIMLMHLAIADLMVTLLLMPMEIVWAWTVQWLSTDLMCRLMSFFRV
FGLYLSSYVMVCISLDRYFAILKPLKRSYNRGRIMLACAWLGSVVCSIPQAFLFHLEEHP
AVTGYFQCVIFSSFRSDFDEKLYQAASMCSMYAFPLIMFIYCYGAIYLEIYRKSQRVLKD
VIAERFRRSNDDVLSRAKKRTLKMTITIVIVFIICWTPYYTISMWYWLDKHSAGKINPLL
RKALFIFASTNSCMNPLVYGLYNIRGRMNNNNPSVNNRHTSLSNRLDSSNQLMQKQLTNN
SLLNGRGQVMAAAVSATTKLANVVSLKGNANGNGSAAAAGTVPITPPLTVTIAPLATDDE
ANDDSCLSAVTIRCQDQSPIRQK

#gi|37359718|dbj|BAC97833.1|_gonadotropin-
releasing_hormone_receptor_3_Oryzias_latipes
MFHHLTDQTVNGSCLGASTDCNKSADGDALQLPTFSTAAKVRVIITFTLCAVSAVCNLLV
LWAAGKGGKRKSHVRILIMNLTVADLLVTFIVMPVDAAWNITVQWQAGDLACRLLMFMKL
VAMYSCAFVTVVISLDRQSAILNPLGISEAKRKSKIMLTVAWTTSVILSLPQMFIFHNVT
ISVPENFTQCTTHGSFVQRWQETLYNMFTFVCLFLLPLVIMIFCYTRILVEISSRIARTN
MVSRDIHLRRSHNNIPKARMRTLKMSIVIVTSFIICWTPYYLLGLWYWLFPEKMEETVSH
SLTHMLFIFGLFNACLDPITYGLFTIHLRQGAKRRRQISNAQTELENNSCLMQMSCLSAH
RQNVSSGLSKHTEEINDNSSTKNASSPSISVSRI

#gi|37359724|dbj|BAC97836.1|_gonadotropin-
releasing_hormone_receptor_1_Oryzias_latipes
MNESSCCHPPAITYQQSSRWDLNASCDWSAPRCNWTSGDGPLQLPTFSTAAKVRVITTFI
LCGVSTLCNSAVLWAAIGHKRKSHVRVLIINLTAADLLVTFIVMPVDAAWNITVQWLAGD
AACRFLMFLKLQAMYSCAFVTVVISLDRQSAILRPLSISAAPRRNRSMLTVAWTMSAVLS
VPQMFIFHNVTITHPANFTQCTTRGSFVTHWQETAYNMFTFTCLFLLPLSIMIICYTRIF
IQISKQMTKKNVSSDEPHLRCSKNNIPKARMRTLKMSVVIVVGFIVCWTPYYLLGLWYWF
FPDDLEGKVSHSLTHILFIFGLFNTCLDPIIYGLFTTRFHRGRRKCYGGATATLSLESKV
VTAEAVKRSSDASASRGDAGEKDNNSARTERQSSGGNI

#gi|37362422|gb|AAQ88392.1|_GnRH_receptor_type_3_Oreochromis_aureus_x_Oreochromis
_niloticus
MNASLCDPAAVMYQLVADHQLNTSCNCSSGLSNWTARGEAPQLPTFSTAAKARVIITFIL
CGISAFCNLAVLWAARVDGKRKSHVRVLIVNLTMADLLVTFIVMPVDAVWNITVQWLAGD
FACRLLMFLKLQAMYSCAFVTVVISLDRQSAILNPLAINKARKRNRIMLMVAWALSVVLS
VPQMFLFHNVTIIHPEDFTQCTTRGSFVTHWHETAYNMFTFCCLFLVPLVIMITCYTRIF
CQISRRLKKDNLPSSEMHLRCSKNNIPRARMRTLKMSIVIVLSFIICWTPYYLLGLWYWF
FPDDLEGKVSHSLTHILFIFGLVNACLDPLIYGLFTIHFRKGLRRFYTGTTTAADLENNT
VITGSFSCAANSLSLKREVSPASPERLLLCKAEWTPPRSSFLTANNDTDRKTHQSSAESI
L

#gi|375298757|ref|NP_001243561.1|_isotocin_receptor_2_Oryzias_latipes
MEIISNESEIWQFNGSWRNSSLGNGTGALNQTNPLKRNEEVAKVEVTVLALVLFLALAGN
LCVLLAIHTTKHSQSRMYYFMKHLSIADLVVAIFQVLPQLIWDITFRFYGPDILCRLVKY
LQVVGMFASTYMLVLMSIDRCLAICQPLHSLHRRKDRIYVILSWLLSLIFSIPQMFIFSL
REVGSAGSGVYDCWGDFVKPWGAKAYITWISLTIYIIPVAILSICYGLISFKIWQNFKLK
TKREQCINLTPKTSQNNTLARVSSVKLISKAKITTVKMTFVIVVAYIVCWTPFFSVQMWS
AWDQAAPREAMPFIISMLLASLNSCCNPWIYMCFAGHLFHDLRQNLLCCSTRYLKSPECR
CERDFNSSHKSNSSNFAIKSTSSSRSITQTSTT

#gi|379317097|dbj|BAL70280.1|_mesotocin_receptor_Protopterus_annectens
MNSSLENSSLENKTGGRNTSGNPLLRNEDAAKVEVAVLSVILFFALTGNICVLLAIHTST
NKQPRMYYFMKHLSIADLVVAIFQVLPQLIWDITFRFYAPDILCRLVKYLQVVGMFASTC
MLLLMTIDRCLAICQPLRSLHRRSDRVSVIVSWIISLLFSIPQIHIFSLKHMGAGVYDCW
ADFIQPWGVKAYVTWITLTVYIIPVVILTVCYGLISFKIWQNAKLKTRREPNMVMATKPA
RGAMLSRVSSVKLISKAKIRTVKMTFVIVLAYIVCWTPFFSVQMLSAWDPDVPQEALAYI
IAMLLASLNSCYNPWIYMYFARHLFHDLAQQFLCCSTHYLKSKQRSCELSSSRRGSLSTY
VFSRKSSSSQKSITQTSST

#gi|379698704|dbj|BAL70407.1|_isotocin_receptor_Amphiprion_ocellaris
MESTSSLINGTGGLNQTNPLKRNEEVAKVEVTVLALVLFLALAGNLCVLLAIHTTKHSQS
RMYYFMKHLSIADLVVAIFQVLPQLIWDITFRFYGPDILCRLVKYLQVVGMFASTYMLVL
MSVDRCLAICQPLRSLHRRKDRFYVLISWILSLLFSIPQMFIFSLREVGLAGSGVYDCWG
DFVKPWGAKAYITWISLTIYIIPVAILSICYGLISFKIWQNFKMKTRRERCISLTPKTSK
GNTLARVSSVKLISKAKITTVKMTFVIVVAYIVCWTPFFSVQMWSAWDPAAPREAMPFII
SMLLASLNSCCNPWIYMCFAGHLFQDLRQNFLCCSAHYLKSSQCRCERDFDSSHKSNSST
FAIKSTNSQRSITQTSTT

#gi|380013103|ref|XP_003690609.1|_PREDICTED:_gonadotropin-releasing_hormone_II_receptor-like_Apis_florea
MGSSIKINTTTELDNSRVNNSNYAELPIDMRFNEGHIVSIIFYSVLMIISAIGNTTVLIL
ITCRKRVSKSRIHIMLMHLAIADLLVTFLMMPLEIGWAITVSWKAGDAMCRIMAFFRMFG
LYLSSFVLVCISMDRYYAVIKPLQLWDVDKRGKIMLSFAWVGSIVCSLPQTIVFHLETHP
NITWYSQCVTFNAFPTYTHEITYSLFGMIMMYWFPLVVIIYTYTSILLEIRRRSKKSEDD
KIRRSSIGFLTRAKIRTLKMTVIIIAVFFICWTPYYVMSLWYWIDRNSAYKIDQRIQKGL
FLFACTNSCMNPIVYGAFNIRDRNKTSTRPTTIETRVTPLSLSLKLLD

#gi|380019458|ref|XP_003693622.1|_PREDICTED:_gonadotropin-releasing_hormone_receptor-like_Apis_florea
MTIPNNYSTYMLPACDNLINFFNHSRTTDLTFDSRLWNTSELSHPFHSYNLKNITCLQHA
PHATISTLFKSLILTIMAVISMLANLATIYSIVRCRRRHHSWSAIYTLILHLAIADLFVS
VFCIGGEAMWNYTVEWIWGNVACKLFKFSQVFSLYLSTFVLVLIGVDRFFAIRYPMKGMN
TADRCLKFIIVAWILSFVLSLPQIIIFHVVEGPFVEEFVQCVTYGFYTEPWQEQLYASFG
LFSMFLLPLAILIATYVFTIITISRSERMFKVKLANNDVRHVNGDVNRRKLMYRAKAKSL
RISIVIVTAFIFWWTPYYTMMIIFMFSSPDKHLSDELQNIIFFFGMSNSLVNPLIYGAFH
LRPRKRRNFIHREISTTQRRFTPTSYGSSYRRDSRETRTPILPKSN

#gi|38230726|gb|AAR14318.1|_corazonin_receptor_Manduca_sexta
MANGGNNTTLYSDLLFTSDPTLHQDFSVDGSIYTNHQVVWPIEKCIDEHLINDSNIDISK
MYMYNGSLVSCLEHAPILTKSTVIRASVLSAMAILSFFGNLATIISIQRGKRGRGRARPS
WTAIYSLIFQLSIADLLVTIFCIAGEAAWSFTVQWYAGNIACKIFKFLQMFALYQSTFIL
VLIGVDRWLAVKYPMKSMATATRSGRLVVIAWVLSVVLSIPQTVVFRVAKGPFVEEFYQC
VTHGFYTERWQEQAYTTLSLVFMFVLPLVILISTYVSTVRTIARSEKVFKPEVRRQEKYF
TPDMNRRRLIDRAKMKSLRMSVVIVAAFLVWWAPYYVMMIIFTFLNPDKDQSEELLSGIF
FFGMSNSLVNPVIYGAFHLWPKKKRSHRHSDRESGGHHASLLRRGDNNTSSIRLTTIRSL
RSSAKYSNGHNISLL

#gi|38349490|gb|AAQ67361.1|_G-protein_coupled_receptor_Anopheles_gambiae
MPPSFNLSTVTYELISGNVSAALENFTASMAGAFPFEDLHDPASSIARNASFTLGLGLAN
VIQLQQQQQQQLHHSPHQYHQQVQHQPQPPSTPFANVSTGQNESLANLLLHPGVHQLSS
GLVTLVGDIMAQSGDTILPREECDRLNISYAFENGTALEIPGLSCYEHAPTLSKSGVIRV
IVLSAMAIVSLLGNVATMWNIQKNRKSRRVTRHNWSAIYSLIFHLSIADVLVTGFCLIGE
AAWYYTVDWVAGNLFCKLFKLCQMFSLYLSTYVLVLVGVDRWVAVKYPMKSLNTARRCHR
FLFVAYLLSFLLSTPQWMIFRVAKGPFVEDFYQCVTHGFYTDRWQEQLYTTFTLVFMFII
PLLILIGTYLSTFMTISSSEKIFRIDTSAVDRTTYYRRSDTNRQRLIHKAKMKSLRISVV
IVVAFVVCWTPYYIMMLIFMFLNPTERFGEDLQSGIFFFGMSNSLINPLIYGAFHLVPIR
QRRNQYNQHVREGSVYFQRSSTFNHHNGHQRNLNPHIKFSNSHSNLPEEISLMSLEKDLR
SLDENVNQIHQKGGGGGGGGGGGGGGVGGGIGLSLGGAAGVDGSRRIKRRSFASKFLSFS
RLLQRNHPTKL

#gi|383860241|ref|XP_003705599.1|_PREDICTED:_gonadotropin-releasing_hormone_II_receptor-like_Megachile_rotundata
MGGTIKVATSTELPRFQINNSDHLELPIDMRFNEGHIVSIFIYSVLMIISAVGNTTVLVL
IIRRRRTSKSRIHNMLMHLAIADLLVTFLMMPLEIGWAITVSWKAGDAMCRIMAFFRVFG
LYLSSFILVCISIDRYYAVIHPLQLWDIDKRGKLMLCLAWGGSIACSMPQMIVFHLETHP
NITWYSQCVTFNTFPTYTHEITYSLFGMIMMYWFPLIVIIYTYTSILLEICRRSKKSEGD
KIRRSSMGFLTRAKVRTLKMTVIIVTVFFICWTPYYVMSLWYWIDRHSAYKVDQRIQKGL
FLFACTNSCMNPIVYGAFNIRDRNKTSVRPATIETRVTPLTLSLKLLD

#gi|383860410|ref|XP_003705682.1|_PREDICTED:_gonadotropin-releasing_hormone_receptor-like_Megachile_rotundata
MTAYHKSPADIPWIVLPACDNLTSGHSRLDLPLQPDHNISELDQLFHIETLNVTCLEHAP
QLTNATFLKVIVLAVMACLSFVANVATIWSIARNRRRQHSWSAIYTLILHLAVADLLVTV
FCIGGEAMWSYSVQWIWGNLACKMFKFLQVFSLYLSTFVLVLIGVDRFVAVRYPMKGLNT

AHRCIRLVAVAWILSFVLATPQIIIFHVARGPFIEEFTQCVTHGFYTETWQEQLYVSLSL
FFMFLLPLAILITTYVSTIITISRSERMFKLELTNNNMYHMNGNININRRKLMHRAKVKS
LRISVVIVAAFVLWWTPYYTMMIILMFLNPDKRLSEELQSGIFFFGMSNSLVNPLIYGAF
HLWPRRNRQSSIHRELSTTQRRMTPTSNNENGCRRGSRENRLPLFSKNNSNTSI

#gi|386873955|gb|AFJ44817.1|_gonadotropin-
releasing_hormone_receptor_1_type_B_Gobiocypris_rarus
MSVNLSLLSASPTHIWENSSLLNASLPRFASDWETSTFTVAARFRVAATLVLFVFAAISN
LSMLISVTMGRGRRLASHLRPLIASLASADLVMTFVVMPLDAIWNITVQWYAGNAMCKLL
CFLKLFAMHSSAFILVVVSLDRHHAILHPLEALDAGRRNRRMLLAAWILSILLASPQLFI
FRAIKAEGVDFVQCVTHGSFQQRWQETAYNMFHFVTLYVFPLLVMSFCYTRILVEINRQM
PRGKGKGGEPCLRRSGTDIIPKARMKTLKMTIIIVASFVVCWTPYYLLGIWYWFQPRMLH
LMPEYVHHALFVFGNLNTCCDPVIYGFFTPSFRADIVGCFCRRNQNSSPKSLDRLSARRG
GASGEAESDLGSGDQPSGQQA

#gi|386873957|gb|AFJ44818.1|_gonadotropin-
releasing_hormone_receptor_1_type_A_Gobiocypris_rarus
MSGNWSLLRVSNASLLDWTPPSFTPAAQARVAATMVLFVLAAVSNLALLISVWRGRGRRL
ASHLRPLILSLVSADLMMTFVVMPLDMVWNVTVQWYAGDGLCKLPCFLKLFAMQTSAFIL
VVISLDRHHAILRPLDSLNAHCRNRRMLLMAWTLSALIASPQLVIFRTVRAESEDFTQCV
THGSFPERWQETAYNMFHFVTLYVIPLLVMSCCYTCILIEINRQLHKSQAGESLRRSGTD
MIPKARMKTLKMTVIIVMSFVVCWTPYYLLGIWYWFQPEMLKVTPEYIHHLLFVFGNLNT
CCDPVIYGLYTPSFRADLARCCRCHPPTDSPRSLDRLSGRGESDPPKAT

#gi|388255253|gb|AFK25141.1|_gonadotropin-releasing_hormone_receptor_2_Sus_scrofa
MSAGNGTPLGSAVGEEVWAGSGVEVESSELPTFSVAAKVRVGVTVVLFVSSAGGNLAVLW
SVTRPQPSQLRPSPVRRLFAHLAAADLLVTFVVMPLDASWNITVQWLAGDIACRTLMFLK
LMAMYAAAFLPVVIGLDRQAAVLHPLGPRSAGRKLLGAAWLLSFLLALPQLFLFHTVRRA
GPVPFTQCVTKGSFKAQWQETTYNLFTFCCLFLLPLTVMTICYSRIVLSVSSPRTRKGND
APAGEFTLRRSLDNRPRVRLRALRLALLVLLTFVLCWTPYYLLGLWYWFSPTMLTEVPPS
LSHILFLFGLLNAPLDPLLYGAFTLGCRRRHQELSSDSSREGGSGRMHQQESQALRQMEV
KTNAAARKAGETKETFL

#gi|389608073|dbj|BAM17648.1|_gonadotropin-
releasing_hormone_receptor_Pagrus_major
MNTTLCDSAAAMYHLTTDHQLNASCNDSTPPSNWTVGGGALQLPTFTTAAKVRVVITCIL
CGVSAFCNLAVLWAAHSDGKRKSHVRVLIINLTVADLLVTFIVMPVDAVWNITVQWLAGD
FACRLLMFLKLQAMYSCAFVTVVISLDRQSAILNPLAINKARKRNRVMLTVAWGMSVLLS
VPQIFLFHNVTIIQPEDFTQCTTRGSFVSHWHETAYNMFTFSCLFLLPLVIMITCYTRIF
CEISKRLRKDNLPPNEVHLRRSKNNIPRARMRTLKMSIVIVSSFIICWTPYYLLGLWYWF
FPDDLEGKVSHSLTHILFIFGLVNACLDPVIYGLFTIHFRTGLRRYYRNATAASDLDNNT
VMTRSFTCAANSLQLKREVSPVSQERFMLCSDSHSREESTPSRSSFLTVDNDAERDPHQF
SSDSII

#gi|390337414|ref|XP_003724555.1|_PREDICTED:_cardioacceleratory_peptide_receptor-
like_Strongylocentrotus_purpuratus
MATQVNFDPGVTTTEGFDYTEPGSNNGTSNGIVDRWSLDKHIQLAVLWVLFTLIIVGNGI
VLIAIWLVRHKKSRLNFFITNLAVADICVGLFSVGFDILDRQTPEFIGGDIACKLYRYVQ
AYVVLASSYQLVALSFDRFFAIVYPMDFTGNGKRSTMLAAGGWILPAVLGITSPVVFQVD
PLASPDGTQMVMSCWPAALYSNRSWILKVYAMYVTSSFFYIPLILITFCYVTIIVTIWTR
AKKMGGPQKVKKSKNANRDVAYEGLSKDSNSTMPKHRASSRGLIPRAKIKTIKMTICIVC
AYICCFMPFSLFYTLEAFGCIDTSSQAVLLATPVLQNLPSLNSATNPFIYGIFSTNVCKE
LRRIPAINWIADKVPCCSAWKPLRFGRPTYQTNTHTTEFNNFSDGHTGSRGRNIVSMSGK
VVDGPSRDDSRSRDDSRNSTTSPM

#gi|391346888|ref|XP_003747698.1|_PREDICTED:_gonadotropin-
releasing_hormone_II_receptor-like_Metaseiulus_occidentalis

MTNASRVTDQNWSGVEKLAVTEAEIAEICPECMFNSESLVSIIVYSILFVCAAVGNVPVF
LSLIRNRHRKSRIKLMMLHLAIADLIVTFIFIPTEIFWVMMVQWKAGDLLCKIFQVVRAF
GPYLSSTIIICISLDRYFAVLHPLKVHDAQRRGKIMIALAWLVSLSWSLPQAVVYEVDGH
PVVKDFYQCITFNFFSNAPQQRVYVLFGLVLQYGIPLLIIIWCYSRIMLAICQRSQDGEL
ATSRNTNNLRVQYRQVRLRRSDPRNAECINRTRNRTLRLTVVIVLTFFWCWTPYVTMVVW
YQIDLDGATKINGYLRNALFMFAVSNSCVNPLLYGSYAKSNWTNVFKKCFSPRKEENEID
GHISNSRQTIIIASGGSSHRSKIIYTGSDPNQLD

#gi|391852574|ref|NP_001254688.1|_gonadotropin-
releasing_hormone_II_receptor_Callithrix_jacchus
MSAVNGTPWGSSAREEVWAGSGVEVEGSELPTFSTAAKVRVGVTIVLFVSSAGGNLAVLW
SVTRPQPSQLRPSPVRRLFAHLAAADLLVTFVVMPLDATWNITVQWLAGDIACRTLMFLK
LMAMYAAAFLPVVIGLDRQAAVLNPLGSRSGVRKLLGAAWGLSFLLALPQLFLFHTVHRA
GPVPFTQCATKGSFKARWQETTYNLFTFCCLFLLPLTAMAICYSRIVLGVSSPRTRKGSH
APAGEFALRRSFDNRPRVRLRALRLALLVLLTFILCWTPYYLLGLWYWFSPSMLSEVPPS
LSHILFLFGLLNAPLDPLLYGAFTLGCRRGHQELSMDSSREEGSRRMFQQDIQALRQTEV
QKTVTSRKAGETKDIPITSI

#gi|403947493|gb|AFR51715.1|_gonadotropin_releasing_hormone_receptor_Sebastes_sch
legelii
MNATMCDPAATMYHLSTDHQLNASCNCSSPPSNWTVGGDALQLPTFTTAAKVGVIITCIL
CGVSAFCNLAVLWAAHSDGKRKSHVRVLIINLTVADLLVTFIVMPVDAVWNITVQWLAGD
FACRLLMFLKLQAMYSCAFVTVVISLDRQSAILNPLAISKARKRNRVMLTVAWGMSAMLS
VPQLFLFHDVTIIHPEDFPQCTTRGSFVIHWHETAYNMFTFSCLFLLPLVIMITCYTRIF
CEISKRLKKDNLPSNEVHLRCSTNNIPRARMRTLKMSIVIVSSFIICWTPYYLLGLWYWF
LPNDLEGKVSHSLTHILFIFGLVNACLDPLIYGLFTIHFRKGLRRFYCNATKASDLDNNT
VLTGSFSCAANSLPLKREVSRTSQERFILRSDDHSKAESTSPISSFLTTDNDAERDPNQS
SPESIV

#gi|405967610|gb|EKC32751.1|_Gonadotropin-
releasing_hormone_II_receptor_Crassostrea_gigas
MNTTVGTGSGNMSDKGLPREMTFNDDNVMSVIAYACLFLFAASGNLTVLVTLLKSRRYKS
RVNTFIMHLSIADLIVAFIMLPLETAWHVTVAWEAGDAACRILMFFRALGFYLSSFVLVA
ISLDRYFSIVHPLSIHDADRRGKIMLTMAWLLSIIASLPQSIIFHVERHPLFTWFEQCVT
FNFFPTEQHELAYNLFNVITVYLLPLVIITTSYSLILYKVSKTARRDEEERREFGLYTHR
SHTYPLSRSGLISCTRAGIIGKARIRTLKMTLVIVTVFVLCWTPYFLMVSWFWIDRESAK
NIDTKVQRGLFIFAVSSACLDPIVYGMFTAAFRKEARRWLGWFKDRLNRMGIYTMTPNTA
SEQDNFKSSESMSFRGLRPGPTGGLMAAPRPTTILSMHFVSVFTLRFPHTSLCVNNGFPV
TDA

#gi|405977158|gb|EKC41621.1|_Gonadotropin-
releasing_hormone_II_receptor_Crassostrea_gigas
MNYSMSSFAIGNPPEVPYNSHGSSSTALTNVLLNGSEPRPNMTGDNALSTTESPMPPTFQ
DSLVKTVVLILLFTISFIGNTATLIQMYRVRKRRSTINTLIVNLAVADLIVTFFCMAAEA
VWALTVQWVAGVAMCKMLRFIQGTGLLVSTYITVVISLDRCCVIIDPISRNKAPQRVRIM
IIMSWFLSALFSIPQVSSVS

#gi|410904683|ref|XP_003965821.1|_PREDICTED:_gonadotropin-
releasing_hormone_II_receptor-like_Takifugu_rubripes
MSSLAPPTSTVAPPTSTVAPPTLPSTWTPPLSTWETPSFTSAAQFRVGATLVLFVFAACS
NLALLASVGRGRRPPSHLRPLMLSLVSADLMMTFVVMPLDAVWNVTVQWHGGDALCKVLC
FLKLFAMYASAFILVVISLDRHHVILHPLNSIKAHKRNRQMLLVAWTLSILLAMPQLFIF
RAVRMEGADFTQCATHGSFSRRWQETVYNMFHFITLYVVPLLVMSCCYSRILLHIHLQHL
REKGESYLRRSGTDIIPKARMKTLKMTVVIVLSFVVCWTPYYLLGIWYWFQPDMLRVTPE
YVHHVLFVFGNLNTCCDPVIYGFYTPSFRADLAACFHQKRRNAARAPPNRARSRDVVN

#gi|410907235|ref|XP_003967097.1|_PREDICTED:_gonadotropin-releasing_hormone_II_receptor-like_Takifugu_rubripes
MAEGAEEPSLAGQLKLGCVCLDGKNPFGASVTPICALTMFHQMTDPALNDSCTGSTSGCN
RSADGDALQLPTFSTAAKVRVIITFSLCAVSAVCNLVVLWAASNGGKRKSHVRILIMNLT
VADLLVTFIVMPVDAVWNITVQWQAGDVACRLLMFLKLVAMYSCAFVTVVISLDRQSAIL
NPLGISEAKRKSKIMLTVAWTMSVILSLPQMFIFHNVTITVPENFTQCTTHGSFVQRWQE
TLYNMFTFVCLFLLPLVIMIFCYTRILVEISSRMARNNLVSKDVYLRRSHNNIPKARMRT
LKMSIVIVTSFIVCWTPYYLLGLWYWLFPEKMEETVSHSLTHMLFIFGLFNACLDPITYG
LFTIHLHQGLKRCCRTANTRTDLENNTCFVHMTRLSSHLHNASGGYHTDKEEVNDDSRSR
PIIPVSKM

#gi|410908101|ref|XP_003967529.1|_PREDICTED:_Arg8-vasotocin_receptor-like_Takifugu_rubripes
MHTPDYALLLNGVNGSLVSRPIELNMGTSGNSTELLNSNGSDPFARNEEVAQIEIMVLSI
TFVVAVIGNVSVLLAMHNTKKKISRMHLFIKHLSLADLVVAFFQVLPQLCWEITFRFYGS
DFLCRIVKHLQVMGMFASTYMMVMMTLDRYIAICHPLKTLQQSSQRSYIMIISTWMCSLA
LSTPQYFIFSLSEIEKGSEVYDCWAHFIEPWGAKAYITWITVGIFLVPVTMLMMCYGFIC
HSIWKNIKFKKRKGTTGAATKNGLIGKNSVSSITTISRAKLRTVKMTFVIVLAYIVCWAP
FFTVQMWSVWDENFQWADSENTAVTLSALLASLNSCCNPWIYMIFSGHLLQDFVHCLPCC
VKMNTDFKKEDSDSSLRRTTLLSKMTNRSPTASSGNWRELDNSPKSSNEVE

#gi|410911976|ref|XP_003969466.1|_PREDICTED:_gonadotropin-releasing_hormone_II_receptor-like_Takifugu_rubripes
MNASSCRHRPVIMYQQSSGADLNGSWDFLAPHGNLTSAAAALQLPTFTTAAKVRVIVTFI
LCGISTFCNLAALWAVNGQKRKSHVRVLIINLTVADLLVTFIVMPVDAVWNITVQWVASD
LACRILMFLKLQAMYSCAFVTVVISLDRQFAILNPLAIIMARRRNKVMLTVAWTMSAVLS
IPQVFIFHNVTITYPANFTQCTTRGSFHSHWQETAYNMFTFSCLFLLPLVIMIICYTRIF
IQISKRMSKKSLSSNEFDLRCSKNNIPKARMRTLKMSIVIVLCFIICWTPYYLLGLWYWF
FPDDLEGKVSHSLTHILFIFGLFNTCLDPVIYGLFTIRFRRGLRGCYRKANAMSDWGAGT
MLRESPKCSAPSSRCQTALAADGKKSNAGHVEQDSCEAS

#gi|410912286|ref|XP_003969621.1|_PREDICTED:_gonadotropin-releasing_hormone_II_receptor-like_Takifugu_rubripes
MNATLCDSMSATMFHLAPECPLNISINYSWPWSNRTAMEGTPLLPTFTTAAKVRVAITWI
LCVVSAFCNMAVLWVAHSEGRRKSHVRMLIVNLTLADLLVTFIVMPVDAMWNVTVQWIAG
DFACRMLMFLKLQAMYSCAFVTVVISLDRQSAILNPLAINEARQRNKVMLTVAWIMSFVL
SLPQIFIFHSVTIILPEDFTQCTTRGSFLTRWHETAYNMFTFSCLFLLPLVIMITCYTRI
FFEISKRMQKDNLPSNEVHLRRSKNNIPKARIRTLKMSIVIVTSFIVCWTPYYLLGLWYW
FFPDDLEGKVSHSLTHILFIFGLLNACLDPIIYGLFTIHFRKGLRRFFGRGAKAAELDNN
TVITGSFTCATTLVPLRREVSSVSCEGSACSDNHTAASLRSKCLMMTLVSSN

#gi|410918599|ref|XP_003972772.1|_PREDICTED:_Arg8-vasotocin_receptor-like_Takifugu_rubripes
MLLSNSTEEPGANLSRNHTDPFGRNEEVAKIEIGVLSLTFVAAVLGNVSVLLATHRKPSR
VHLFMKHLSLADLVVAFFQVLPQLCWEVTFRFYGPDFLCRIVKHLQVLGMFASAYMMVMM
TLDRYIAICHPLQTLQRPAQRAYVMIGGTWAGSLALSAPQYFIFSLSEVSPGSAVYDCWG
HFVEPWGLRAYITWMTAGIFVVPVAALVFCYGFICRTIWKNLKCKTQRKSVEAVAEATGA
GILGPCSVSSVSILSRAKLRTVKMTFVIVLAYVLCWAPFFTVQMWSVWDHTFSWDDSEST
AVTLSALLASLNSCCNPWIYMLFGGRLLSDCAGSLPCCSRLGRRFTRRHSDCSLRGTTLL
SRLQGPRLSEPLALTGSLEVPPVS

#gi|410928722|ref|XP_003977749.1|_PREDICTED:_gonadotropin-releasing_hormone_II_receptor-like_Takifugu_rubripes
MSRNVSILLPPSPSSPSWEVPSFSVAAQCRVATTLVLFVFAAVSNSAVLISVFWGRGYRL
GAHLRPVIASLAAADLMMSFIVMPLDAVWNITVQWYAGDIMCKLMCFLKLFAMHSAAFIL
VVVSLDRYRAILHPLDSLDAGTRNRRMLAVAWSLSMLLASPQLFIFRAIKAEGVDFTQCV
THGSFLYLWQETTYNMFHFVTLYVFPLLVMIFCYTRIFTKINGQIHKNKDNENCLRRSGR

DVIPKARMKTLKMSVVIVSSFVICWTPYYLLGIWYWFHPAMIQHTPEYVHHMLFVFGNLN
TCCDPIIYGFYTPSFRADLADVMACCCGHRAADASPRSVDRLSAPVGGAAMEMESDLSSN
QQSGNAG

#gi|421975810|gb|AFX72968.1|_GnRH_receptor_1b_Anguilla_anguilla
MRMSENSSLLMLGSQGTSMNSSSNSSVSPPSADWVAPTFTRAAQFRVVATLVLFLFAAVS
NLAVLISVARGRGRRLASHLRPLIMSLAVADLMMTFIVMPLDMVWNVTVQWYAGDAMCKL
LCFLKLFAMHSSAFILVVISLDRHHAILRPLDSFDAKRRNKRMLLLAWSLSLLLASPQLF
IFRVIKAEGVDFTQCVTHGSFQQRWQETVYNMFLFVTLYVVPLLVMSLCYTRILIEINHQ
IHKSKGGESCLRRSGTDMIPKARMKTLKMTIIIVVSFVVCWTPYYLLGIWYWFHPQMIQV
TPEYVNHILFVFGNLNTCCDPVIYGLYTPSFRADLAVCWFCRRQDSSPKSLDRLSARQGN
PSAEQESDAPSVDQMRANEE

#gi|42627727|dbj|BAD11150.1|_gonadotropin-
releasing_hormone_receptor_2_Eublepharis_macularius
MTEGEPLMNPLQRCHAADENSSVSACPKAWVEPTFTLAAKVRVVITSCFFVFATCSNSVV
FASVVRKRRKSHVQLLILSLTVADLLVTVAVMPLDAVWNVTIQWYAGDALCKILNFLKLF
AMYSAALVLVVISLDRHAAILHPFSFANSSHRNRIMLCVAWMLSVLLASPQLFIFHLHTI
PGVNFTQCVTHGSFREHWQETTYNMFTFTTLYVTPLSVMIICYVHILFEISKQLKINKGL
ARGKDDHISKARMKTLKMTIVIVASFIVCWTPYYLLGLWYWFQPDMISQMPEYINHFLFL
FGLLHTCTDPIIYGLYTPSFREDMKACLRSLEKAVTRQERSKLASVSERNRDYHDSGVAP
SIASNGGTLNTAC

#gi|432110859|gb|ELK34333.1|_Oxytocin_receptor_Myotis_davidii
MLNKAVSFLPLGAAVSPFQNPELAEEDPEGLQGLGVTSPYIIKKEKNGYSLARVPFHTLG
SHPADLPAESPGEKSEPPQARQEAPPLRPTPSGPAPSRRALKSTKGQDHAVVATVMEGTP
EANWSAEVVNGSAAPPGAEGNRTQAPQRNEALARVEVAVLCLILFLALSGNACVLLALRT
TRHKHSRLFFFMKHLSIADLVVAVFQVLPQLLWDITFRFYGPDLLCRLVKYLQVVGMFAS
TYLLLLMSLDRFTWLGCLVASAPQVHIFSIREVADGVFDCWAGFIQPWGPKAYVTWITLA
VYIVPVIVLTACYSLICFKIWQNFRIKTAAEGAAKGPEGAMPGSREGTALARVSSVKLIS
RAKIRTVKMTFIIVLAFIVCWTPFFFVQMWSVWDANAPKEASAFIIAMLLASLNSCCNPW
IYMLFTGHLFHELLQRILCCSSSYRRDSRPGETSVSKKSNSSTFVLSRHSSSQRSTSQLS
SL

#gi|432117195|gb|ELK37633.1|_Gonadotropin-
releasing_hormone_receptor_Myotis_davidii
MSGTKFVIRLNWFNYNKTPEVPEARVLRFYPLGKMANGASAEQSQHHCSPTNSSSPLMQG
SLPTLTLSGKIRVTVTFFLFLLSTTFNATFLLKLKQWTQKKEKGKKLSRMNVLLKHLTLA
NLLETLIVMPLDGMWNITVQWYGGELLCKVLSYLKLFSMYAPAFMMVVISLDRSLAITRP
LPVSNSKLGQSLIGVAWLLSSLFAGPQLYIFRMIHLADGSGQSDGFSQCVTHCSFPQWWH
QAFYNFFTFSCLFIIPLLIMLICNAKIIFTLTRVLHQDPHNLQLNQSKNNIPRARLRTLK
MTVAFATSFTVCWTPYYVLGIWYWFDPEMLNRVSDPVNHFFFLFAFLNPCFDPLIYGYFS
L

#gi|432852692|ref|XP_004067337.1|_PREDICTED:_gonadotropin-
releasing_hormone_II_receptor-like_Oryzias_latipes
MNDSLYESTLTMYHLLEDEQANISCNFSSLNWTSCNVLQLPTFSTAAKVRVIITFILCGF
SAFFNLAVLWAAQSDGKRKSHVKVLIINLTVADLLVTFIVMPLDAVWNITVQWLAGDFAC
RLLMFLKLQAMYSCAFVTVVISLDRQSAILNPLAINKARMRNRVMLIAAWAMSVILSVPQ
IFLFHNVTIIYPEEFTQCATRGSFVSHRHETAYNLFTFACLFLLPLVIMITCYTRIFCEI
SKRLKRDNLPSSELHLRCSKNNIPKARMRTLKMSIVIVLSFIICWTPYYLLGLWYWFSPD
DLEGKVSHSLTHILFIFGLLNACLDPVIYGLFTIHFRKKLRRFYGNAAEATDADINTVVT
GSFTCAASSLSLKRKLWHPSQERLVLHRDNHSKADLPSPKGNLLLAESKAVRSLNQSSRE
SIL

#gi|432860411|ref|XP_004069538.1|_PREDICTED:_Arg8-vasotocin_receptor-
like_Oryzias_latipes

MMGRTGGSAMYTLSSVLLLSGENRSLDSSLSSDLMMGTSGNGTANSNGSDPFGRNEEVAQ
IEIMVLSITFVVAVIGNVSVLLAMYNTKKKMSRMHLFIKHLSLADLVVAFFQVLPQLCWE
ITFRFYGSDFLCRIVKHLQVMGMFASTYMMVMMTLDRYIAICHPLKTLQQPTKRSYIMIT
STWMCSLVLSTPQYFIFSLSEIKNGSDVYDCWAHFIQPWGAKAYITWITVGIFLVPVVIL
MLCYGFICHSIWKNIKYKKRKTTAGASNKNGLIGKNSVSSVTTISRAKLRTVKMTFVIVL
AYIVCWSPFFIVQMWSVWDENFLWDDSDNTAVTLSALLASLNSCCNPWIYMIFSGHLLQD
FVHCFSCCRRVNADKKEDSDSSIRRTTLLTKMTNRSPTGSTGNWKELDNSPKNSAQAE

#gi|432943818|ref|XP_004083285.1|_PREDICTED:_Arg8-vasotocin_receptor-like_Oryzias_latipes
MLFPSDSLCNLTGQNCSLNVTQQLGGVKKNHSDPFGRNEEVAKIEITVLSLAFVAAVVGN
VSVLLAMHRTRRKLSRMHLFMKHLSLADLVVAFFQVLPQLCWEITFRFYGPDFLCRIVKH
LQVLGMFASTYMMVMMTLDRYIAICHPLKTLQQPTQRAYIMIGSTWACSLVLSTPQYFIF
SLSEVRPGSAVYDCWGHFMEPWGLRAYITWITAGIFLLPVAILVFCYGFICRTIWMNIKY
KTRRKRTDAAEGATTNGVLSRSSVSSVSTISRAKLRTVKMTFVIVLAFVVCWAPFFTVQM
WSVWDQTFSWDDSENTTVTLSALLASLNSCCNPWIYMIFSGHLLSDFFSGLPCCRRLKIQ
LHQQDSDSSVRRTTLLSRLQGPRLSEPFRDLNLTGKSCPQVPSASYQTDLSRAES

#gi|4406500|gb|AAD20001.1|_gonadotropin_releasing_hormone_receptor_type_A_Carassius_auratus
MSDNTSLPSVSNASLLPPLTDWRAPSFTPAAQARVAATMVLFLFAAVSNLALLISVSRGR
GRRLASHLRPLIISLVSADLMMTFIVMPLDMVWNVTVQWYAGDGLCKLLCFLKLFAMQTS
AFILVVISLDRHHAILHPLDSLNAHQRNRRMLLLAWSLSALIASPQLFIFRTVKVKSVDF
TQCVTHGSFHERWYETAYNMFHFVTLYVIPLLVMSCCYTCILIEINRQLHKSTEGESLRR
SGTDMIPKARMKTLKMTIIIVLSFVVCWTPYYLLGIWYWFQPEMLKVTPEYIHHLLFVFG
NLNTCCDPVIYGLYTPSFRADLARCWRCRTPAESPRSLDRIPHENTSPTRPA

#gi|4406502|gb|AAD20002.1|_gonadotropin_releasing_hormone_receptor_type_B_Carassius_auratus
MSGKMPLLSVNPTSIWENSSVLNATPHFPSDWETPTFTVAAHFRVVATLVLFVFAAISNL
SVLISVTRGRGRHLASHLRPLIGSLASADLVMTFVVMPLDAIWNITVQWYAGNAMCKNLC
FLKLFAMHSAAFILVVVSLDRHHAILHPLEALDAGRRNRRMLLAAWILSILLASPQLFIF
RAIKAEGVDFVQCVTHGSFRQRWQETAYNMFHFVTLYVFPLLVMSFCYTHILVEINRQMP
RGKGKGGEPCLRRSGTNMIPKARMKTLKMTIIIVASFVVCWTPYYLLGIWYWFQPRMLQS
MPEYIHHALFVFGNLNTCCDPVIYGFFTPSFRADIASCFCRRNQNSSLKSLDRLSVRRGG
ASREAESDLGSGDQPSGQQA

#gi|442631927|ref|NP_001261755.1|_corazonin_receptor_isoform_B_Drosophila_melanogaster
MEDEWGSFDRLPSVPSASMDLETENEVVSNWSTLANFTRLVAGAAPEIVNYTLNMIDVGV
GMATDISNLSVSTTPLPAYAISNSSSLAHTNSRHEAPPMAEQVPEHVMDHAPQLSRSGLL
KVYVLAVMALFSLLGNLLTIWNIYKTRISRRNSRHTWSAIYSLMFHLSIADVLVTWFCII
GEAAWCYTVQWLANELTCKLVKLFQMFSLYLSTYVLVLIGVDRWIAVKYPMKSLNMAKRC
HRLLGGTYILSLVLSLPQFFIFHVARGPFVEEFYQCVTHGFYTADWQEQMYATFTLVFTF
LLPLCILFGTYMSTFRTISSSEKMFQGSKLANYSTAKLPTQTNRQRLIHKAKMKSLRISV
VIIIAFLICWTPYYVMMIMFMFLNPDKRLGDDLQDAIFFFGMSNSLVNPLIYGAFHLCPG
KGGKSSGGGGNNNAYSLNRGDSQRTPSMLTAVTQVDGTGGSSRQMRAFRQQSYYRSSSNG
TAGPGAAPFKEQVGLLHVGPGNGTPGGSVSSGATPQLIRKGSALLARQPSCLREQEHQQR
LLLHEKPSTLVLSYDSQRGGVGVGVASGLLDNNERVSSV?EQLAMAVAAAAVAVDAPDTV
EAAAATASADADANVAMEDACSCCHCLSRELVRRQLELQVLPVQ

#gi|443690008|gb|ELT92261.1|_hypothetical_protein_CAPTEDRAFT_97007_Capitella_teleta
MDCSEALIACGDPYHGLPLNETANTSDSNFEVPRELLFNTDAIVSVSILGVMFLVAAVGN
LTVFAILFFNRHSRLSRVSIFIMHLSISDLIITFIMIPMEIGWHVTVAWKAGDVGCRVLL
FFRAFGFYLGSFILVAISLDRYLSITKPLSLVDAGKRGRIMLRFSWLFAFVAAIPQSIIF
HVEQHPIHTWFSQCVTFNFFPSDQHEMAYNVSTVCMMYVVPLLLITACYSLILREVSKKT

SSTRTVSGGQCRVRMSGNGRLKMAKARTNTLRMTIVIVLVFVICWSPYFCMLLVWWIDRG
QAVKVPPKLQRAIFIFAVSNSCVNPFVYGTCSHIYINGASMSTSMKPRYSNVCLN

#gi|443703272|gb|ELU00909.1|_hypothetical_protein_CAPTEDRAFT_104885_Capitella_teleta
MVFGEEGLISVIAYCILFLIAACGNLTVFITLYRNRHRKSRVNLFIMHLSVADLIVTFIV
LPLEVTWHITVAWYAGDAACRIIMFFRAMGFYLSSFILIAISLDRYFAILHPLSLNDADK
RSKLMIIFSWAFSVIASIPQSIIFHVEQNPTYSWFSQCVTFNFFPSEGHELAYNMFNMIT
VYALPLVVIIVSYSLILCEMTRKTRESQEDVKAPSKMNASERLRRSNVGHIQRARIRTLR
LTIMMVLVFILCWTPYFVMSSWWWFDRSSAAKVDPRIQKGLFLFAVSNSCMDPIVYGKPC
IRDTPPRI

#gi|443703855|gb|ELU01220.1|_hypothetical_protein_CAPTEDRAFT_125249_Capitella_teleta
MNFTTSLDVWQQENASTVGPGAAGPPREIQFNGDSVVSIVTYSVLFCFSAVGNLSVFLTL
VAARYQRRASRVSLFILHLTVADLFVTFVMIPMEIGWHVTVSWKAGDVACRLLMVLRTFG
FYLSSLILIAISLDRYLSIAHPISIQSAGRRSRIMILVAWVIGFIASLPQSLIFHAAQHP
EFELYVQCVTFGSFPTQAHELAYNLFNMFLLYSIPLIAIVLCYSLILREMDASQNTFEIM
ADPDPIVGQTGRLDKARLHTLKMTIVIVAVFIVCWTPYFIMHILWWIDKNTAEKVNPKIQ
RGLFIFAVSNSCVNPVIYGQYFM

#gi|443724328|gb|ELU12393.1|_hypothetical_protein_CAPTEDRAFT_136071_partial_Capitella_teleta
FQTPQLAFLCVIFVFIVTGNISVIIAIFMSKARKSRMHFFILHLAAADLLTGMITVVTDL
SWKITVEWHAGLFGCKVIRFLQSVVVFAANYILVALSIDRLDAIARPMNFSSSGRRAKML
VSTAWILAFLFSAPMGIIPELTVRRGLVQCFISLPEPWHWKVYITILAFALFILPAIIIS
TCYIIIVIVIWSKGNVSTTSNKRQLSKRGRESTTSSRGMIPKARIKTIKMTFAIVTVFII
CWVPFFVFDLADVYGLIPRTKHKRAIAIFIQSLATLNSATNPVIYFIFSKTQCSPLR

#gi|4502331|ref|NP_000697.1|_vasopressin_V1a_receptor_Homo_sapiens
MRLSAGPDAGPSGNSSPWWPLATGAGNTSREAEALGEGNGPPRDVRNEELAKLEIAVLAV
TFAVAVLGNSSVLLALHRTPRKTSRMHLFIRHLSLADLAVAFFQVLPQMCWDITYRFRGP
DWLCRVVKHLQVFGMFASAYMLVVMTADRYIAVCHPLKTLQQPARRSRLMIAAAWVLSFV
LSTPQYFVFSMIEVNNVTKARDCWATFIQPWGSRAYVTWMTGGIFVAPVVILGTCYGFIC
YNIWCNVRGKTASRQSKGAEQAGVAFQKGFLLAPCVSSVKSISRAKIRTVKMTFVIVTAY
IVCWAPFFIIQMWSVWDPMSVWTESENPTITITALLGSLNSCCNPWIYMFFSGHLLQDCV
QSFPCCQNMKEKFNKEDTDSMSRRQTFYSNNRSPTNSTGMWKDSPKSSKSIKFIPVST

#gi|4504059|ref|NP_000397.1|_gonadotropin-releasing_hormone_receptor_isoform_1_Homo_sapiens
MANSASPEQNQNHCSAINNSIPLMQGNLPTLTLSGKIRVTVTFFLFLLSATFNASFLLKL
QKWTQKKEKGKKLSRMKLLLKHLTLANLLETLIVMPLDGMWNITVQWYAGELLCKVLSYL
KLFSMYAPAFMMVVISLDRSLAITRPLALKSNSKVGQSMVGLAWILSSVFAGPQLYIFRM
IHLADSSGQTKVFSQCVTHCSFSQWWHQAFYNFFTFSCLFIIPLFIMLICNAKIIFTLTR
VLHQDPHELQLNQSKNNIPRARLKTLKMTVAFATSFTVCWTPYYVLGIWYWFDPEMLNRL
SDPVNHFFFLFAFLNPCFDPLIYGYFSL

#gi|4507343|ref|NP_001049.1|_substance-P_receptor_isoform_long_Homo_sapiens
MDNVLPVDSDLSPNISTNTSEPNQFVQPAWQIVLWAAAYTVIVVTSVVGNVVVMWIILAH
KRMRTVTNYFLVNLAFAEASMAAFNTVVNFTYAVHNEWYYGLFYCKFHNFFPIAAVFASI
YSMTAVAFDRYMAIIHPLQPRLSATATKVVICVIWVLALLLAFPQGYYSTTETMPSRVVC
MIEWPEHPNKIYEKVYHICVTVLIYFLPLLVIGYAYTVVGITLWASEIPGDSSDRYHEQV
SAKRKVVKMMIVVVCTFAICWLPFHIFFLLPYINPDLYLKKFIQQVYLAIMWLAMSSTMY
NPIIYCCLNDRFRLGFKHAFRCCPFISAGDYEGLEMKSTRYLQTQGSVYKVSRLETTIST
VVGAHEEEPEDGPKATPSSLDLTSNCSSRSDSKTMTESFSFSSNVLS

#gi|45382825|ref|NP_989984.1|_gonadotropin-releasing_hormone_receptor_Gallus_gallus
MCVPAALIEAEPPHHPTTEGDTNTSATHCLEHWVEPRFTKAAKVRVAITAVFFLLAACSN
TAVLGSLLRKRRKCHVRPLILSLALADLLVTVAVMPLDAAWNVTVQWYGGDLSCKLLNFL
KLFAMYAAALVLVVISLDRHAAVLQPFARARRRNGLLLRAAWLGSVLLASPQLFLFHVHT
VPGGNFTQCVTHGSFRAHWEETVYNMFTFTTLYITPLSIMIVCYVRIIWEISKQLKINKS
LVRSQNDHISKARMKTLKMTIVIVASFIICWTPYYLLGLWYWFHPAMIQRMPEYINHSFF
LFGLLHTCTDPIIYGLYTPSFREDVQLCLRGIEAAISQHVRHKPISVSEKTTKDGDVNGQ
VTSGGSNGTTVNTVC

#gi|45552233|ref|NP_995639.1|_adipokinetic_hormone_receptor_isoform_C_Drosophila_melanogaster
MAKVAEENDHRDLSNWSNVNDTNGTIHLTKDMVFNDGHRLSITVYSILFVISTIGNSTVL
YLLTKRRLRGPLRIDIMLMHLAIADLMVTLLLMPMEIVWAWTVQWLSTDLMCRLMSFFRV
FGLYLSSYVMVCISLDRYFAILKPLKRSYNRGRIMLACAWLGSVVCSIPQAFLFHLEEHP
AVTGYFQCVIFNSFRSDFDEKLYQAASMCSMYAFPLIMFIYCYGAIYLEIYRKSQRVLKD
VIAERFRRSNDDVLSRAKKRTLKMTITIVIVFIICWTPYYTISMWYWLDKHSAGKINPLL
RKALFIFASTNSCMNPLVYGLYNIRGRMNNNNPSVNNRHTSLSNRLDSSNQLMQKQLTNN
SLLNGRGQVMAAAVSATTKLANVVSLKGTANGNGSAAAAGTVPITPPLTVTIAPLATDDE
ANDDSCLSAVTIRCQDQSPIRQKCGDSIELTSVVK

#gi|465967294|gb|EMP31298.1|_Gonadotropin-releasing_hormone_II_receptor_Chelonia_mydas
MNATFRMKVSELLEPGLPAVGNQSRGQPWEEVEVSPGGGANASLPSEEVFLLPTFSTAAK
VRVAITFVLFLSSACFNIAVLWTVTQKYRKRPHVRILIVNLAAADLLVTFVVMPLDAAWN
ITVQWYAGDLACRALMFLKLVAMYASAFITVVISLDRQAAILHPLSMGDAKKKNKAMLCV
AWALSVLLALPQMFVFHAVSRSQPIYFIQCATVGSFQAHWQETLYNMFTFSCLFLLPLLI
MVLCYSRILIEISGKMKRACVSSKEVHLRRSYNNIPRARMRTLKMSIVIVLTFVLCWTPY
YLLGLWYWFSPEMLSRKKVPPSLSHILFLFGLFNACLDPLIYGLFTMHFHREIRCVCRCT
HRRKEQASALIGSFRASTTAAPIRSAREDQGGTGKYELEVTANMALPAGRCELCRKKIVE
SFI

#gi|466002037|gb|EMP41667.1|_Gonadotropin-releasing_hormone_II_receptor_Chelonia_mydas
MTVGQIHSWRNMDRLQGSHTKDGLLCLILLSVVCVVLTLLSALVPGNKQITVAALKQVHA
GMPEERDLMTPPQSADVVDENLSASDCPEPWIEPTFTLAARVRVIVTICFFLIAACSNSV
VLYSIMRKRRKSHVRLLILSLTVADLLVTFTVMPLDAVWNVTVQWYAGDLPCKLLNFLKL
FAMYSAALVLVVISIDRHSAILHPFAFANSSRRNRLMLYVAWVMSLLLASPQLFLFHLHT
VPGVNFTQCVTHGSFQEHWEEILYNMFTFTTLYVAPLSVMIVCYIRIIWEISKQLKINKG
LARSKNNHISKARMKTLKMTIVIVASFIICWTPYYLLGLWYWFQPDMIQSMPEYINHSLF
LFGLLHTCTDPIIYGLYTPSFREDMKACLRGIETVITGQERNKLMSSSEMNIKDYIINGG
AASGASNGTIIHTVC

#gi|47523620|ref|NP_999438.1|_gonadotropin-releasing_hormone_receptor_Sus_scrofa
MANSASPEQNQNHCSAINSSILLTQGNLPTLTLSPNIRVTVTFFLFLLSTAFNASFLLKL
QKWTQRKEKGKKLSRMKVLLKHLTLANLLETLIVMPLDGMWNITVQWYAGEFLCKVLSYL
KLFSMYAPAFMMVVISLDRSLAITRPLAVKSNSRLGRFMIGLAWLLSSIFAGPQLYIFRM
IHLADSSGQTEGFSQCVTHGSFPQWWHQAFYDFFTFSCLFIIPLLIMLICNAKIMFTLTR
VLQQDPHNLQLNQSKNNIPRARLRTLKMTVAFAASFIVCWTPYLVLGIWYWFDPEMVNRV
SDPVNHFFFLFAFLNPCFDPLIYGYFSL

#gi|48675949|ref|NP_001001639.1|_gonadotropin-releasing_hormone_(type_2)_receptor_2_Sus_scrofa
MSAGNGTPLGSAVGEEVWAGSGVEVESSELPTFSVAAKVRVGVTVVLFVSSAGGNLAVLW
SVTRPQPSQLRPSPVRRLFAHLAAADLLVTFVVMPLDASWNITVQWLAGDIACRTLMFLK
LMAMYAAAFLPVVIGLDRQAAVLHPLGPRSGGRKLLGAAWLLSFLLALPQLFLFHTVRRA
GPVPFTQCVTKGSFKAQWQETTYNLFTFCCLFLLPLTVMTICYSRIVLSVSSPRTRKGND

APAGEFTLRRSLDNRPRVRLRALRLALLVLLTFVLCWTPYYLLGLWYWFSPTMLSEVPPS
LSHILFLFGLLNAPLDPLLYGAFTLGCRRRHQELSSDSSREGGSGRMHQQESQALRQMEV
KTNAAARKAGKTKETFL

#gi|498930198|ref|XP_004539654.1|_PREDICTED:_gonadotropin-releasing_hormone_II_receptor-like_Maylandia_zebra
MVDEHNVPSPELQKMNASLCDPAAVMYRLVADHQLDTSCNCSSALSNWTARGVAPQLPTF
STAAKARVIITFILCGISAFCNLAVLWAARIDGKRKSHVRVLIVNLTMADLLVTFIVMPV
DAVWNITVQWLAGDFACRLLMFLKLQAMYSCAFVTVVISLDRQSAILNPLAINKARKRNR
IMLTVAWVMSVVLSVPQMFLFHNVTIIHPEDFTQCTTRGSFVTHWHETAYNMFTFCCLFL
LPLVIMITCYTRIFCEISRRLKKDNLPSSEMHLRCSKNNIPRARMRTLKMSIVIVLSFII
CWTPYYLLGLWYWFFPDDLEGKVSHSLTHILFIFGLVNACLDPLIYGLFTIHFRKGLRRF
YTGTTTSADLENNTVITGSFSCAANSLSLKREVSPASPERLLLCKAEWTPPRSSFLTADN
DTDRKTHQSSAESML

#gi|498930987|ref|XP_004539832.1|_PREDICTED:_gonadotropin-releasing_hormone_II_receptor-like_Maylandia_zebra
MNGSSCCDPAAVMYQQRSGLDLNASCEWPDPHCNWTSVDGALQLPTFSTAAKIRVIVTFI
LCGISTFCNLAVLWAANGHKRKSHVRVLIINLTAADLLVTFIVMPVDAVWNITVQWLAGD
LACRFLMFLKLQAMYSCAFVTVVISLDRQSAILNPLGIAMVRKRNRVMLMVAWIMSALLS
IPQMFIFHNVTITYPANFTQCTTRGSFVTHWQETAYNMFTFCCLFLLPLVIMIICYTRIF
IQISKQMTKKNMPSSEPHLRCSKNNIPKARMRTLKMSIVIVICFIVCWTPYYLLGLWYWF
FPDDLEGKVSHSLTHILFIFGLFNACLDPIIYGLFTIRFQKGLRNCYRKAAVMSSLETNA
VIMESLKCTGSVLPSKRGMTSGEKDFSSEQAEAKSTDNSV

#gi|498994488|ref|XP_004553261.1|_PREDICTED:_gonadotropin-releasing_hormone_II_receptor-like_Maylandia_zebra
MYHQLTDQTVNGSCQGPTSACNKSADGDALELPTFSTAAKVRVIITFALCAVSAVCNLAV
LWAASSGGRRKSHVRILIMNLTVADLLVTFIVMPVDAVWNITVQWQAGDVACRLLMFMKL
VAMYSCAFVTVVISLDRQSAILNPLGISEAKRKSKVMLAVAWTMSVILSLPQMFIFRNVT
ITVPEKFTQCTTHGSFVQRWQETLYNMFTFVCLFLLPLAIMIFCYTRILIEISSRMARNN
FLSRDVHLRRSHNNIPKARMRTLKMSIVIVTSFIICWTPYYLLGLWYWLFPEKMEKTVSH
SLTHMLFIFGLFNACLDPITYGLFTIHFHKGMRRCRQSSNARTELENNTRLVQMSRLSSR
RQIASDVHSASTEVVRESNIMKHVSNPDMSISKI

#gi|499005092|ref|XP_004535493.1|_PREDICTED:_gonadotropin-releasing_hormone_II_receptor-like_isoform_X1_Ceratitis_capitata
MKMPQNEDEVNEKIYDHRIMPDWSNVVNETNGTMHYSKDMIFNDGHRLSITVYSILLVLS
SIGNSTVLYLIVKRRWHNRTRSPSRIDIMLMHLAIADLMVTFLLMPLEIAWAYTVQWRST
DFVCRTMSFFRVFGLYLSSFVLVSISIDRYYAILKPLKFSTNRGRIMLAIAWVASVVCST
PQAFVFHLEEHPKVKGYYQCVTFHSFPTELHKLIYQISNMCAMYAFPLVTFIYCYGCIYR
EIYRKSKRMVKGDDKNGFARFRRSNDDVLGRAKKRTLKMTITIVIVFVICWTPYYIMSLW
FWLDKASAYNVNPLLRKIFFIFACTNSCMNPLVYGVFNIRGKGNNNNTSVNNRHTSLSSA
GRLAMRERNGSAHTVVTLPAVINGSSGDTSNVTQSTTLSNDIFTISKIAEDMEIEKAPKI
SISDSIELAHAPKSQ

#gi|499005100|ref|XP_004535495.1|_PREDICTED:_gonadotropin-releasing_hormone_II_receptor-like_isoform_X3_Ceratitis_capitata
MKMPQNEDEVNEKIYDHRIMPDWSNVVNETNGTMHYSKDMIFNDGHRLSITVYSILLVLS
SIGNSTVLYLIVKRRWHNRTRSPSRIDIMLMHLAIADLMVTFLLMPLEIAWAYTVQWRST
DFVCRTMSFFRVFGLYLSSFVLVSISIDRYYAILKPLKFSTNRGRIMLAIAWVASVVCST
PQAFVFHLEEHPKVKGYYQCVTFHSFPTELHKLIYQISNMCAMYAFPLVTFIYCYGCIYR
EIYRKSKRMVKGFARFRRSNDDVLGRAKKRTLKMTITIVIVFVICWTPYYIMSLWFWLDK
ASAYNVNPLLRKIFFIFACTNSCMNPLVYGVFNIRGKGNNNNTSVNNRHTSLSSAGRLAM
RERNGSAHTVVTLPAVINGSSGDTSNVTQSTTLSNDIFTISKIAEDMEIEKAPKISISDS
IELAHAPKSQ

#gi|504159178|ref|XP_004590911.1|_PREDICTED:_gonadotropin-releasing_hormone_receptor_isoform_X1_Ochotona_princeps
MANSAASEQNQNHCLAINNSILVMQANLPTLTLSGKIRVTITFFLFLLSTTFNASFLLKL
QKWTQKGKTLSRMKVLLKHLTLANLLETLIVMPLDGMWNITVQWYAGEFLCKVLSYLKLF
SMYAPAFMMVVISLDRSLAITRPLAVKSSHKLKQSMIGLAWLLSSLFAGPQLYIFRMLHL
ADGTEQMEVFSQCVTHCSFPQWWHQAFYNLFTFSCLFLIPLLIMLICNAKIIFTLTRVLH
QDPHKLQLNQSKNNVPRARLRTLKMTVAFATSFIVCWTPYYVLGIWYWFDPEMLNQVSEP
VNHFFFLFAFLNPCFDPLIYGYFSL

#gi|505851549|ref|XP_004619220.1|_PREDICTED:_gonadotropin-releasing_hormone_II_receptor-like_Sorex_araneus
MSAGNRTAPEPKAGEVWAGSGAEVEGLELPTFSTAAKVRVGVTIVLFVSSATGNLAVLWS
VTRPQSSPLRPSPIRRLFAHLAAADLLVTFVVMPLDATWNVTVQWLAGDIACRALMFLKL
MAMYAAAFLPVVIGLDRQAAVVHPLGPRSGGRKLLGAAWGLSFLLALPQLFLFRTVRRAG
PIPFTQCVTKGSFKARWQETTYNFFTFSCLFLLPLTAMTVCYSRIVLSVSSPQSRKGSPA
RAAELALRRSSDNRPRVRLRALRLALLVLLTFVLCWAPYYLLGLWYWFSPAMLTQVPPSL
SHILFLFGLLNAPLDPLLYGAFTLGCRRGTHELSVEISREGESGKMTQPKIQVLRQLETQ
RDVPPQAAEETKETLM

#gi|507713721|ref|XP_004647900.1|_PREDICTED:_gonadotropin-releasing_hormone_II_receptor-like_Octodon_degus
MSAGNGTPSGPAVGEEAWAGSGVQVEGSELPTFSAAAKVRVGVTIVLFLSSAGGNLAVLW
SVTRPQPSHLRPSPVRRLFAHLAAADLLVTFVVMPLDATWNITVQWLAGDIACRTLMFLK
LMAMYAAAFLPVVIGLDRQAAVLNPLGPRSGGRKLLGTAWGLSFLLALPQLFLFHTVRRA
GPVPFTQCVTKGSFKAQWQETTYNLFTFCCLFLLPLTAMAICYSRIVLSVSGPQPSKRSR
APAGEFNLRRSLDTRPRVRLRAMRLALLILLTFVLCWTPYYLLGLWYWFSPTMLSEVPPS
LSHILFLFGLLNAPLDPLLYGAYTLGCRRGHQEPSLDGSSQGGPWRMSGQDTQAPRQLGI
TTKVSSREGEQRETFL

#gi|507943027|ref|XP_004681318.1|_PREDICTED:_gonadotropin-releasing_hormone_receptor_isoform_X1_Condylura_cristata
MANSASEQNQNHCSAANSSSSLLEGNFPTLTLSGKIRVTVTFFLFLLSTTFNASFLLKLQ
KWTQKKEKSKKLSRMKVLLKHLTLANLLETLIVMPLDGMWNITVQWYAGELLCKVLSYLK
LFSMYAPAFMMVVISLDRSLAITRPLALKRSSNLGQPMIVLAWLLSSIFAGPQLYIFQVI
HLADSSGQTKGFSQCVTHCSFPQWWHQAFYNFFTFSCLFIIPLLIMLICNAKIIFTLTQV
LQQDPHQLQLNQSMNNIPRARLRTLKMTVAFATSFTVCWTPYYVLGIWYWFDPEMLNRVS
DPVNHFFFLFAFLNPCFDPLIYGYFSL

#gi|512391013|gb|AGO01050.1|_GnRHR_Cairina_moschata
MAGQGPGAAAAGGHRPDAGPAAGNSSTEPPGSPTLPEQGCAWSPRPEGGEEPLRLPTFSP
AAQARVAVTFALFALSAGCNLAVLRAAGGRGGARRSHIRLLLRHLAAADLLVTVAVMPLD
AVWNITLQWRAGDLACRLLMYLRLLAMYASAFVTVVISLDRQAAILHPLAIARARRRNRA
MLRAAWLLSAALSLPQLFLFRTVTLQPPHNFTQCTTRGSFPQRWHETLYNMLSFTCLFLL
PLLIMVCCYARILLEISRRMGSGLFPSRDVPLRCSGNNIPRARLRMLKMSLVIVSSFILC
WTPYYLLGLWYWFCPRAMQKRVSPSLTHILFIFGLFNACLDPITYGLFTIPFRRGCGCPC
GHSPEPEPPSPATGSFRCSASSLRARRGAGGVQGQQVPARPGLPAGAGSCQSSAL

#gi|512772525|gb|AGO05922.1|_isotocin_receptor_Sparus_aurata
MEDFLREQNSWAQNVSWSNLSRGNESHLGNTTVNPLKRNEEVAKVEVAVLVLVLLLALTG
NLCVLWAIHATKHSQSRMYFFMKHLSIADLVVAVFQVLPQLIWDITFRFYGPDLLCRLVK
YLQVVGMFASTYMLVLMSIDRCLAICQPLRSVHKRNDRLCVVASWMLSLIFSSPQAYIFS
LKEVGDGVYDCWGDFVQPWGAKAYITWMSLSIYIFPVAILSVCYGLIWFKKWQNFNLKTR
REHILALTARPSKGALPLTRVSSVRLISKAKIRTVKMTFVVVVAYIMCWTPFFFVQMWSA
WDPAAPREDMAFIIAMLLASLNSCCNPWIYMFFAGHLFQDLTRCFFCCCRQYLTASTCSC
DRPCRHKSSSATYVIKNESSQRSLTHTSSTGGLGH

#gi|523704505|ref|NP_001265799.1|_isotocin_receptor-like_Oryzias_latipes

MEDLLLELESWSHNVSARNSSCRNESGAENSTVNPLKRNEEAAKVEVTVLVLVLFLALMG
NLCVLLAIHTTKHSHSRMYYFMKHLSIADLVVAVFQVLPQLIWDITFRFYGPDILCRLVK
YLQVVGMFASTYMLVLMSVDRCSAICLPFRFVNKRRDRICVIASWMLSLVFSAPQAYIFS
LKEVGNGVYDCWGDFVHPWGAKAYITWMSLSIYILPVAILGICYGLICFKIWENINMKTR
RDRFVAVTSKGTHPLSRVSSVRLISKAKIRTVKMTFVVVIAYIVCWTPFFFVQMWSAWDP
AAPREDMAFIIAMLLASLNSCCNPWIYMFFAGHLFHDLIQCFFSCCRQYLTDSSCGCDEQ
RRDKSSSSSDVIKNAREASHTHPA

#gi|525008495|ref|XP_005051891.1|_PREDICTED:_gonadotropin-
releasing_hormone_II_receptor-like_Ficedula_albicollis
MSEEQLPASSHCPTAREDTNVSASVYPQYWVEPRFTQAAKVRVIITAVFFLLAAGSNTAV
LGSLLRKRRKSHVQPLILSLALADLLVTVMVMPLDAAWNVTVQWYGGDISCKILNFLKLF
AMYAAALVLVVISLDRHAAILRPFSRAHRRNGMLLRAAWGGSVLLALPQLFLFHLNTIPG
GNFTQCVTHGSFRAHWEETVYNMFTFTTLYITPLSVMIVCYIRILWEISKQLKVNKGLTR
NQNDHISKARMKTLKMTVVIVATFIICWTPYYLLGLWYWFQPAMIQKMPEYVNHSFFLFG
LLHTCTDPIIYGLYTPSFREDVQLCLRGIETAITRHKRHKPISASEKNIKDGAANGGMAS
GGSNGTTVSTV

#gi|528524336|ref|XP_005174743.1|_PREDICTED:_gonadotropin_releasing_hormone_recep
tor_1_isoform_X1_Danio_rerio
MSGNVSLSLISLLENSSLASSSSPQSPQWETPTFTTAARYRVVATLVLFVFAAVSNLSVL
ISVTRGRGRHLASHLRPLIASLASADLVMTFVVMPLDAIWNITVQWYAGDAMCKLLCFLK
LFAMHSAAFILVVVSLDRHHAILHPLEALDAGRRNRRMLLAAWILSVLLASPQLFIFRAI
KAEGVDFVQCATHGSFQHRWQETAYNMFHFVTLYVFPLLVMSFCYTRILVEINRQMPRGK
GKGGEPCLRRSGADMIPKARMKTLKMTIIIVASFVVCWTPYYLLGIWYWFQPRMLQVTPE
YVHHALFVFGNLNTCCDPVIYGFFTPSFRADITSCFSRRNQNCSPKSLDRLSARRGGASG
EAESDLGSGDQPSGQTA

#gi|529439539|ref|XP_005239517.1|_PREDICTED:_gonadotropin-
releasing_hormone_II_receptor-like_Falco_peregrinus
MEAWGLPPYIPATGTPPQLHGVVDSNVTSWLSPHGAGAGAPSDLPGDPLLDAELMLPEFV
DAGTVTQAAAACLPIVAGRDHLDPGPAVGNTSAEPPGNLSPREQGCAWSPQAEGGEEPLQ
LPTFSPAAQARVAVTLVLFALSASCNLAVLRAAGGRAILHPLAIARARRRNRIMLYVAWL
LSAGLSVPQLFLFHTVTLRPPHNFTQCTTRGSFPQPWHETLYNMLGFTCLFLLPLLIMVC
CYTRILLEISRRMGSSLCECLGTRLPRPRLRMLKMSLVIVSSFILCWTPYYLLGLWYWFC
PRAMEKRVSPALTHILFIFGLFNACLDPITYGLFTIPFQRGWGCPPGHGPEPQPSSPATG
SFHCSASSLPPKRGIPGTRGWQVPPGTTGSCQSSSL

#gi|529445640|ref|XP_005242530.1|_PREDICTED:_gonadotropin-
releasing_hormone_II_receptor-like_Falco_peregrinus
MSDEQLPTAPRCPAVERDTNISVSGCPEHWVEPRFTQAARVRVIFTAIFFLLAVGSNVAV
LGSLLRKRRKSHVRPLILSLALADLLVTLTVMPLDAVWNVTVQWYGGDTSCKLLNFLKLF
AMYAAALVLVVISLDRHAAILHPFSHARRRNGLLLRTAWAGSVLLALPQLFLFHLHTIPG
GNFTQCVTHGSFRAHWEETAYNMFTFTTLYITPLSVMIVCYIRIIWEISKQLKNNKGLVR
SQNDHISKARMKTLKMTIVIVATFIICWTPYYLLGLWYWFQPAMIQKMPEYVNHSFFLFG
LLHTCTDPVIYGLYTPSFREDVQLCLRGVETAITRHKRHKPVSVSEKNIKDGAVNGGVAS
GGSNGTTVNTIC

#gi|530604232|ref|XP_005293855.1|_PREDICTED:_gonadotropin-
releasing_hormone_II_receptor-like_Chrysemys_picta_bellii
MNATFRMKVSELLEPGSPAVGNQSRGQPWEEVEVSPGGGANTSLPGEEVFLLPTFSTAAK
VRVGITFVLFLSSACFNIAMLWTVTQKYRKRPHIRILIVNLAAADLLVTFVVMPLDAAWN
ITVQWYAGDLACRALMFLKLVAMYASAFITVVISLDRQAAILHPLSMGDAKKKNKTMLCV
AWALSVLLALPQMFVFHAVSRSQPIYFIQCATVGSFQAHWQETLYNMFTFSCLFLLPLLI
MVLCYSRILIEISGKMKRACVSSKEVHLRRSYNNIPRARMRTLKMSIVIVLTFVLCWTPY
YLLGLWYWFSPEMLSRKKVPPSLSHILFLFGLFNACLDPLIYGLFTMHFRREIRLVCRCT
HRRKEQASTPIGSFRASTTAAPIRSAGEDQGGSGKYELEVTANVALPAGRCELCRKKIVE

SFI

#gi|530622383|ref|XP_005300801.1|_PREDICTED:_gonadotropin-releasing_hormone_II_receptor-like_Chrysemys_picta_bellii
MNVSQGAGMVPCSSLLPSEGGCGWGPWPNGSEGALQLPTFSPAAKARVILTFVLLTLSAA
GNLAVLWAGAGGRRGKLSHVRVLLLHLAAADLLVTFVVMPLDAVWNITVQWRAGDLACRL
LMYLKLLAMYASAFITVVISLDRQAAILRPLAIAQAHRRNRLMLYAAWLLSAGLSVPQLF
LFHTVTIRSPQTFTQCTTWGSFPRRWHETAYNMLGFACLFLLPLLIMVSCYSRILLEISR
RMGTGLFSKEVALRRSSSNIPRARLRVLKLSLVIVSSFVVCWTPYYLLGLWYWFCPRAME
ETVSQSLTHVLFIFGLLNACLDPLTYGLFTIPFRRGLGHCCRGGHGAGPEPCSPATGSSR
CSATSLHAKRSTTTTPAHETPGAQSQLGNGTGSVDSNYL

#gi|532113397|ref|XP_005341377.1|_PREDICTED:_gonadotropin-releasing_hormone_II_receptor-like_Ictidomys_tridecemlineatus
MSAGNGTPRRSAAGEEMWAGSGVELEDAELPTFSAAAKVRVGVTVVLFVSSAGGNLAVLW
SVTRRQSSQCRPSPVRRLFAHLAAADLLVTFVVMPLDATWNITVQWLAGDIACRTLMFLK
LMAMYAAAFLPVVIGLDRQAAVLNPLGPRSGAKKLLGIAWGLSFLLALPQLFLFHTVRKA
GPVPFTQCVTKGSFKAQWQETTYNLFTFCCLFLLPLTAMAICYSRIVLSVSSPRTRKGSQ
APADEFILRRSLDTRPRVRLRALRLALLVLLTFVLCWTPYYLLGLWYWFSPTMLTEVPPS
LSHILFLFGLLNAPLDPLLYGAFTLGCRRGHQTLSKDSSQEGGSGRMPQQESKTLRQLKM
QTIVTSRGGETKETFL

#gi|541991258|ref|XP_005446235.1|_PREDICTED:_gonadotropin-releasing_hormone_II_receptor-like_Falco_cherrug
MALGRDHLDPGPAVGNTSAEPPGNLSPREQGCAWSPQAEGGEEPLQLPTFSPAAQARVAV
TLVLFALSASCNLAVLRAAGGRRGSRRSHIHLLLLHLAVADLLVTVAVMPLDAIWNITLQ
WNRIMLYVAWLLSAGLSVPQLFLFHTVTLRPPHNFTQCTTRGSFPQPWHETLYNMLGFTC
LFLLPLLIMVCCYTRILLEISRRMGSSLCDQAKCPPLMRHAPMSLHCSRNNIPRARLRML
KMSLVIVSSFILCWTPYYLLGLWYWFCPRAMEKRVSPALTHILFIFGLFNACLDPITYGL
FTIPFQRGWGCPCGHGPEPQPSSPATGSFRCSASSLPPKRGIPGTRGWQVPPGTTGSCQS
SSL

#gi|542149164|ref|XP_005483541.1|_PREDICTED:_gonadotropin-releasing_hormone_II_receptor-like_Zonotrichia_albicollis
MSEEQLPASPHCPTTQEDTNVSASAFPQYWVEPRFTPAAKVRVIITAMFFLLAAGSNAAV
LGSLLRKRRKSHVQPLILSLALADLLVTVMVMPLDAAWNVTVQWYGGDISCKVLNFLKLF
AMYAAALVLVVISLDRHAAILHPFSRAHRRNGMLLRAAWAGSVLLALPQLFLFHLNTIPG
RNFTQCVTHGSFRAHWEETVYNMFTFTTLYITPLSVMIVCYIRILWEISKQLKVNKGLTR
NQNEHISKARMKTLKMTIVIVATFIICWTPYYLLGLWYWFQPAMIQKMPEYVNHSFFLFG
LLHTCTDPIIYGLYTPSFREDVQLCLRGIETAITRQKRHKPISASEKNTKDGAANAGVAS
GGSNGTTVSTV

#gi|543269516|ref|XP_005424330.1|_PREDICTED:_gonadotropin-releasing_hormone_II_receptor-like_Geospiza_fortis
MSEEQLPASSHCPTTQEDTNVSASAYPQYWVEPRFTQAAKVRVIITAIFFLLAAGSNAAV
LGSLLRKRRKSHVQPLILSLALADLLVTVMVMPLDAAWNVTVQWYGGDISCKILNFLKLF
AMYAAALVLVVISLDRHAAILHPFSRAHRRNGMLLRAAWAGSVLLALPQLFLFHLNTIPG
RNFTQCVTHGSFRAHWEETVYNMFTFTTLYITPLSVMIVCYVRILWEISKQLKVNKGLTR
NQNEHISKARMKTLKMTIVIVATFIICWTPYYLLGLWYWFQPAMIQKMPEYVNHSFFLFG
LLHTCTDPIIYGLYTPSFREDVQLCLRGIEIAITRQKRQKPISASEKNTKDGAANGGVAS
GGSNGTTVSTV

#gi|543269628|ref|XP_005424367.1|_PREDICTED:_gonadotropin-releasing_hormone_II_receptor-like_Geospiza_fortis
MAWLGNARQDTLDTACLQLFLFHTITLRPPHNFTQCTTRGSFPRAWHETLYNMVGFACLF
LLPLLIMVCCYARILLEISRRMGSGLFSSRDASLRCSRNNIPRARLRMLRMSLVIVSSFI
LCWTPYYLLGLWHWFCPRAVERRISPALTHILFIFGLFNACLDPITYGLFTIPLRGGWGC

PCRHGPPARPPSPATGSFRCSASSVPPQRGAQGLPRPRGAARDPSCHSSSL

#gi|543276537|ref|XP_005426691.1|_PREDICTED:_oxytocin_receptor_Geospiza_fortis
MEKLYLAGSSTWIINSSLGNGSLRLENLSAGRNSTADPLKRNEDMAKVEVTVLCLILFLA
LTGNLCVLLAIHTTRHKHSRMYFFMKHLSIADLVVAIFQVLPQLIWDITFRFYGPDFLCR
LIKYLQVVGMFASTYMLLLMSLDRCLAICQPLRSLHRRADRVSVLLTWLLCLLVSIPQIH
IFSLRDVGNGVYDCWADFIQPWGPKAYVTWITLMVYIIPVLMLSICYGLISFKIWQNVKL
KTAHGPSVGLGSGSRGGMVFARVSSTRLISKAKIRTVKMTFIIVLAFIVCWTPFFFVQMW
SVWDTNAPQEASPFIIAMLLASLNSCCNPWIYMLYTGHLFHDLMRRFLCCSTRYLKSRPA
CELSKRSNSSSFVLSCRATSQRSFVQPPTT

#gi|543354759|ref|XP_005521846.1|_PREDICTED:_gonadotropin-releasing_hormone_II_receptor-like_Pseudopodoces_humilis
MSEEQLPASPHCPTVQEDTNVSASAYPQYWVEPQFTQAAKVRVIITAIFFLLAAGSNTAV
LGSLLRKRRKSHVQPLILSLALADLLVTVMVMPLDAAWNVTVQWYGGDISCKILNFLKLF
AMYAAALVLVVISLDRHAAILHPFSRAHHRNGMLLRAAWAGSVLLALPQLFLFHLNTVPG
RNFTQCVTHGSFRAHWEETVYNMFTFTTLYITPLSVMIICYIRILWEISKQLKVNKGLTR
NQNDHISKARMKTLKMTIVIVATFIICWTPYYLLGLWYWFQPTMIQKMPEYVNHSFFLFG
LLHTCTDPIIYGLYTPSFREDVQLCLRGIETAITRQKRHKPILASERNIKDGAANGGVAS
GGSNGTTVSMV

#gi|543354800|ref|XP_005521861.1|_PREDICTED:_gonadotropin-releasing_hormone_II_receptor-like_Pseudopodoces_humilis
MAWLGNARQDRLDAGRGHPDLGPAVGNASAEPPGPTPPEWGCAWSPPEEHGEEPLRLPTF
SPAAQVRVAVTFALFALSAGCNLAVLRAVGGRGSGRRPHIRLLLRHLAAADLLVTVVVMP
LDAVWNITLQWRAGDLACRVLMYLRLLAMYASAFVTVVISLDRQAAILRPLAIARARARN
RAMLHAAWLLSAGLAVPQLFVFHTITLRPPHNFTQCTTRGSFPRPWQETLYNMVGFACLF
LLPLLIMVCCYARILLEISRRMGSGLFSSQDASLRCSRNNIPRARLRMLRMSLVIVSSFI
LCWTPYYLLGLWHWFCPRAMEKRISPALTHILFIFGLFNACLDPITYGLFTIPLRGGWGC
PCKHGPLAQPPSPATGSFRCSASSLPPGRGLQELRRSRGAARDASSHSSSL

#gi|543355249|ref|XP_005522015.1|_PREDICTED:_oxytocin_receptor_Pseudopodoces_humilis
MEKLYLAGSSTWTINSSLENGSLRLENPSAGRNSSADPLKRNEDMAKVEVTVLCLILFLA
LTGNLCVLLAIHTTRHKHSRMYFFMKHLSIADLVVAIFQVLPQLIWDITFRFYGPDFLCR
LIKYLQVVGMFASTYMLLLMSLDRCLAICQPLRSLHRRADRVSVLLTWLLCLLVSIPQIH
IFSLRDVGNGVYDCWADFIQPWGPKAYVTWITLMVYIIPVLMLSICYGLISFKIWQNVKL
KTAHGPSVGLGSGSRGRMVFARVSSTRLISKAKIRTVKMTFIIVLAFIVCWTPFFFVQMW
SVWDTNAPQEASPFIIAMLLASLNSCCNPWIYMLYTGHLFHDLRRRFLCCSTRYLKSRPG
CELSKKSNSSSFVLSCRSTSQRSFVQPPTT

#gi|543719536|ref|XP_005500990.1|_PREDICTED:_gonadotropin-releasing_hormone_II_receptor-like_Columba_livia
MEGDTNISAPGCLEHWVEPQFTQAARVRVIITAIFFLLAAGSNAAVLGSLLRKRRESHVR
PLILSLALADLLVTVAVMPLDAAWNVTVQWYGGDISCKLLNFLKLFAMYASALVLVVISL
DRHAAVLHPFSRARRRNGLLLRAAWAGSVLLALPQLFLFHLHTAPGGNFTQCVTHGSFRA
HWEETIYNMFTFTTLYITPLSVMIFCYVRIIWEISKQLKINKGLIRNQNDHISKARMKTL
KMTIVIVATFIICWTPYYFLGLWYWFQPAMIQKMPEYVNHSFFLFGLLHTCTDPVIYGLY
TPSFREDVQLCLRGIETAITRHERHKPISVTEKNIKDGAVNGGVASGGSNGTTVNTAC

#gi|543736231|ref|XP_005509070.1|_PREDICTED:_oxytocin_receptor_Columba_livia
MQKLYFAGSSLWTINSSLGNGSLHLENQSSGRNSTTDPLKRNEDMAKVEVTVLCLILFLA
LTGNLCVLLAIHTTRQKHSRMYFFMKHLSIADLVVAIFQVLPQLIWDITFRFYGPDFLCR
LIKYLQVVGMFASTYMLLLMSLDRCLAICQPLRSLHRRADRVSVLLTWLLCLLVSIPQIH
IFSLRDVGNGVYDCWADFIQPWGPKAYVTWITLMVYIIPVLMLSICYGLISFKIWQNVKL
KMAHGPNMGLGSSSCSGTAFARVSSTRLISKAKIRTVKMTFIIVLAFIVCWTPFFFVQMW
SVWDTNAPQEASPFIIAMLLASLNSCCNPWIYMLYTGHLFHDLMRRFLCCSTHYLKSRPA

CELSVGKKSNSSSFVLSCRETTERSSGEPRVLQGLGSCSEPLGQNIYSRIGCNYFYSHKK
L

#gi|544403038|ref|XP_005542152.1|_PREDICTED:_gonadotropin-releasing_hormone_II_receptor-like_isoform_X1_Macaca_fascicularis
MSAGNGTPWGSAAGEEAWAASGVAVEGSELPTFSAAAKVRVGVTIVLFVSSAGGNLAVLW
SVTRPQPSQLRPSPVRTLFAHLAAADLLVTFVVMPLDATWNITVQWLAGDIACRTLMFLK
LMAMYSAAFLPVVIGLDRQAAVLNPLGSRSGVRKLLGAAWGLSFLLALPQLFLFHTVHRA
GPVPFTQCVTKGSFKARWQETTYNLFTFRCLFLLPLTAMAICYSHIVLSVSSPQTRKGSH
APAGEFALCRSFDNCPRVRLRALRLALLILLTFILCWTPYYLLGLWYWFSPTMLTEVPPS
LSHILFLFGLLNAPLDPLLYGAFTLGCQRGHQELSIDSSNEGSGRMLQQEIHALRQQEVQ
KTVTSRSAGETKDISITSI

#gi|548344274|ref|XP_005724558.1|_PREDICTED:_isotocin_receptor-like_Pundamilia_nyererei
MEDLLREQYSWSHNLTWSSSSRENESYVGNTTVNPLKRNEEVAKVEVTVLVLVLLLALTG
NMCVLWAIHTTKHSQSRMYYFMKHLSIADLVVAIFQVLPQLIWDITFRFYGPDLLCRLVK
YLQVVGMFASTYMLVLMSIDRCLAVCQPLRSVHKKKDRFCVIASWMLSLIFSSPQAYIFS
LREVGNGVYDCWGDFVQPWGAKAYITWMSLSIYIFPVAILSICYGLICFKIWENFNLKTR
REHFLALTPRPSKGAQPFSRVSSVRLISKAKIRTVKMTFVVVLAYIVCWTPFFFVQMWSA
WDPAAPREDMAFIIAMLLASLNSCCNPWIYMFFAGHLFHDLMQCFFCCCRRYLTECSCSC
DQQCRHKRGSSTYVNKNTNSQRSLSRTSSTVH

#gi|548376257|ref|XP_005733449.1|_PREDICTED:_gonadotropin-releasing_hormone_II_receptor-like_Pundamilia_nyererei
MNASLCDPAAVMYQLVADHQLDTSCNCSSALSNWTARGMAPQLPTFSTAAKARVIITFIL
CGISAFCNLAVLWAARIDGKRKSHVRVLIVNLTMADLLVTFIVMPVDAMWNITVQWLAGD
FACRLLMFLKLQAMYSCAFVTVVISLDRQSAILNPLAINKARKRNRIMLTVAWVMSVVLS
VPQMFLFHNVTIIHPEDFTQCTTRGSFVTHWHETAYNMFTFCCLFLLPLVIMITCYTRIF
CEISRRLKKDNLPSSEMHLRCSKNNIPRARMRTLKMSIVIVLSFIICWTPYYLLGLWYWF
FPDDLEGKVSHSLTHILFIFGLVNACLDPLIYGLFTIHFRKGLRRFYTGTTTSADLENNT
VITGSFSCAANSLSLKREVSPASPERLLLCKAEWTPPRSSFLTADNGTDRKTHQSSAESM
L

#gi|548515061|ref|XP_005695760.1|_PREDICTED:_oxytocin_receptor_Capra_hircus
MDRMFGAGGATGTREKVLECFEILVPEAPPPSPRPPPVRLSVLATWLGCLVASAPQVHIF
SLREVADGVFDCWAVFIEPWGPKAYITWITLAVYIVPVIVLAACYGLISFKIWQNLRLKT
EAAAAEAAAGAEGAAADCPGRAALARVSNVKLISKAKIRTVKMTFIVVLAFIVCWTPFFF
VQMWSVWDADAPKEASAFIIAMLLASLNSCCNPWIYMLFTGHLFQELVQRFLCCSFRRLK
GSQPGETSVGKKIHSYTFVLSRYSSSQRSCSQPSTV

#gi|551493404|ref|XP_005798270.1|_PREDICTED:_gonadotropin-releasing_hormone_II_receptor-like_Xiphophorus_maculatus
MFHQLKDQTVNGSCQGTTLDCNKSADGNALQLPTFSTAAKVRVIITFTLCAVSAVCNLIV
LWAAGKGGKRKSHVRILIMNLTVADLLVTFIVMPVDAVWNITVQWQAGDAACRLLMFMKL
VAMYSCAFVTVVISLDRQSAILNPLGISEAKRKSKIMLTVAWTMSFILSLPQIFIFHNVT
ITVPENFTQCTTHGSFIQRWQETLYNMFTFTCLFLLPLVIMIFCYTRILVEISSRMAQNN
TLSRDIHLRRSHSNIPKARMRTLKMSIVIVTSFIICWTPYYLLGLWYWLFPEKMEETVSH
SLTHMLFIFGLFNACLDPITYGLFTIHFRQGLRRRCQNSTTHTELENNTCLVQMSGLSSR
RHFTSGGSGKQTGEESDSSNLKNASCPVISVSEV

#gi|551502058|ref|XP_005802574.1|_PREDICTED:_gonadotropin-releasing_hormone_II_receptor-like_Xiphophorus_maculatus
MNTSSCCAAPVAMYQQSSGSDLNASCDLSAPRCNWTAVDGTPQLPTFSTAAKVRVIVTFI
LCGVSTLCNVAVLWAASGHRRRSHVRVLIVNLTAADLLVTLIVMPVDAAWNITVQWLAGD
LACRFLMFLKLQAMYSCAFVTVVISLDRQSAILDPLQISMAPKRNRVMLTVAWIMGALLS
IPQVFIFHNVTITYPANFTQCTTRGSFVTHWQETAYNMFTFSWLFLLPLAIMITCYSRIF

IHISKQMTKKNVSSDEPRLRCSKNNIPKARMRTLKMSVVIVVSFIVCWTPYYLLGLWYWF
FPDDLEGKVSHSLTHILFIFGLFNTCLDPIIYGLFTVRFSRRPRSRCSRATVISRLDAKP
ATAESVKCTLPGGSAKGGRDGSCEPNEQKSTDSRL

#gi|551514148|ref|XP_005808585.1|_PREDICTED:_gonadotropin-
releasing_hormone_II_receptor-like_Xiphophorus_maculatus
MNAIPCESAVTMRDPVVDPHVNVTSNCSLDAEDVPQLPTFTTAAKVRVIITFVLCGASAF
CNLAVLWAAHRDGKRKSHVRVLIVNLTVADLLVTFVVMPVDAAWNITVQWLAGDFACRLL
MFLKLLAMYSCAFVTVVISLDRQAAILNPLAINKARMRNRIMLAVAWGVSVALSIPQIFL
FHNVTIVHPEKFTQCTTRGSFATRWHETAYNMFTFSCLFLLPLVIMITCYARIFHEISKR
LEKDNLRLRCSKNNIPKARMRTLKMSVVIVSSFIVCWTPYYLLGLWYWFFPDHLEDKVSH
SLTHILFIFGLVNACLDPLIYGLFTIHFRKGLRRYYPNAASAGDAENTTVLTGSFTSPAL
SLSLRRELRRLSQEKLVLCGDGHRKAGPPSLSSSFLAEDRDVQQSSPESIL

#gi|554804360|ref|XP_005913135.1|_PREDICTED:_gonadotropin-
releasing_hormone_II_receptor-like_Haplochromis_burtoni
MNGSSCCDPAAVMYQQRSGLDLNASCEWPDPHCNWTSVDGALQLPTFSTAAKIRVIVTFI
LCGISTFCNLAVLWAANGHKRKSHVRVLIINLTAADLLVTFIVMPVDAVWNITVQWLAGD
LACRFLMFLKLQAMYSCAFVTVVISLDRQSAILNPLGIAMVRKRNRVMLMVAWIMSALLS
IPQMFIFHNVTITYPANFTQCTTRGSFVTHWQETAYNMFTFCCLFLLPLVIMIICYTRIF
IQISKQMTKKNMPSNEPHLRCSKNNIPKARMRTLKMSIVIVICFIVCWTPYYLLGLWYWF
FPDDLEGKVSHSLTHILFIFGLFNACLDPIIYGLFTIRFQKGLRNCYRKAAVMSSLETNA
VIMESLKCTGSVLPSKRGMTSGEKDISSEQAEAKSTDNSV

#gi|554836489|ref|XP_005928728.1|_PREDICTED:_gonadotropin-
releasing_hormone_II_receptor-like_Haplochromis_burtoni
MYHQLTDQTVNGSCQGPTSACNKSADGDALELPTFSTAAKVRVIITFALCAVSAVCNLAV
LWAASSGGRRKSHVRILIMNLTVADLLVTFIVMPVDAVWNITVQWQAGDVACRLLMFLKL
VAMYSCAFVTVVISLDRHSAILNPLGISEAKRKSKVMLAVAWTMSVILSLPQMFIFRNVT
ITVPEKFTQCTTHGSFVQRWQETLYNMFTFVCLFLLPLAIMIFCYTRILIEISSRMARNN
FLSRDVHLRRSHNNIPKARMRTLKMSIVIVTSFIICWTPYYLLGLWYWLFPEKMEKTVSH
SLTHMLFIFGLFNACLDPITYGLFTIHFHKGMRRCRQSSNARTELENNTRLVQMSRLSSR
RQIASDVHSASTEVVRESNIMKHVSNPDMSISKI

#gi|555943808|ref|NP_001273217.1|_gonadotropin-releasing_hormone_II_receptor-
like_Haplochromis_burtoni
MNASLCDPAAVMYQLVADHQLDTSCNCSSALSNWTARGTAPQLPTFSTAAKARVIITFIL
CGISAFCNLAVLWAGAQDGKRKSHVRVLIVNLTMADLLVTFIVMPVDAVWNITVQWLAGD
FACRLLMFLKLQAMYSCAFVTVVISLDRQSAILNPLAINKARKRNRIMLMVAWVMSVVLS
VPQMFLFHNVTIIHPEDFTQCTTRGSFVTHWHETAYNMFTFCCLFLLPLVIMITCYTRIF
CEISRRLKKDNLPSSEMHLRCSKNNIPRARMRTLKMSIVIVLSFIICWTPYYLLGLWYWF
FPDDLEGKVSHSLTHILFIFGLVNACLDPLIYGLFTIHFRKGLRRFYTGTTTSADLENNT
VITGSFSCAANSLSLKREVSPASPERLLLCKAEWTPPRSSFLTADNDTDRKTHQSSAESI
L

#gi|556963563|ref|XP_005991707.1|_PREDICTED:_gonadotropin-
releasing_hormone_II_receptor-like_isoform_X1_Latimeria_chalumnae
MNFSFHGVIEESTNPALGGTHLANVSCFKVMKEAIPSINTSSPEEIFILPTFSTAAKVRV
AITFLLFLSSACFNLAVLWTVSQKCRRKSHVKVLIMNLAVADLLVTFIVMPLDASWNITV
QWYGGDLSCRILMFLKLAAMYSSAFVTAVISIDRHSAILNPLGIADANKKNKIMLCMAWV
LSVLLAIPQFFVFHMVSRSQPVYFIQCATVGSFQEHWQETLYNMFTFTCLFLLPLLIMVF
CYSRILLEISRKMKKACISSKEIDLRRSYNNIPKARMRTLKMSIIIVLTFIVCWTPYYLL
GIWYWFSPEMLTREKVPESLSHILFIFGLFNACLDPLIYGLFTVQFRRACRCAKAVRDKD
AASLMTSSFRFSTSTTHAKRAGPRVPVGRIRYEMKALRSSTKEINCDLCKRNIAENFM

#gi|55725596|emb|CAE54804.2|_gonadotrophin_releasing_hormone_receptor_1A_Dicentra
rchus_labrax

MTGNLSLWVSTPADPLQNDSVFSLLSSPAPTSPPPWEAPSFTVAARCRVAATLVLFVFAA
ASNLSVLISVCWGRGYRLAAHLRPLIASMASADLVMTFVVMPLDAIWNITVQWYAGDIMC
KLLCFLKLFAMHSAAFILVVVSLDRYRAILHPLDSLDAGLRNRRMLLVAWTLSLLLASPQ
LFIFRAIKADGVDFTQCVTHGSFQYLWQETAYNMFHFVTLYVFPLLVMTFCYTRILAKIN
GQMHKTKDGEHCLRRSGTDMIPKARMKTLKMTIVIVSSFVICWTPYYLLGIWYWFQPAMI
KHTPEYVHHILFVFGNLNTCCDPVIYGFYTPSFRADLADVLACCHGRQTNASPRSVDRLS
ARSAGAAVEMESDLSSNQHSGNPS

#gi|557269739|ref|XP_006020172.1|_PREDICTED:_gonadotropin-releasing_hormone_II_receptor-like_Alligator_sinensis
MIEGMTEEQHLRATLQATGAMEENVSISSCSDTWIEPTFTLAARVRVIITICFFLVAVCS
NSAVLYSILRKRRKSHVKLLILSLTAADLLVTFTVMPLDAVWNVTVQWYAGDLLCKLLNF
LKLFAMYSAALVLVVISIDRHSAILHPFTFASSSRRNRLMLCVAWVMSLFLASPQLFLFH
LHTVPGVNFTQCVTHGSFREHWEETAYNMFTFTTLYVTPLSVIIVCYIRIIWEISKQLKI
NKGLARSKDDHISKARMKTLKMTIVIVATFIICWTPYYLLGLWYWFQPDMIQRMPEYINH
SLFLFGLLHTCTDPVIYGLYTPSFREDMKMCLRGIETVFTGQERHKPISSSEVNVKDYVA
NGGAASGGSNGTTVHTVC

#gi|557282603|ref|XP_006024205.1|_PREDICTED:_mesotocin_receptor-like_Alligator_sinensis
MEKLYFAGNDLWTINGSLENTSLSLENLTYGKNSTTDPLKRNEDMAKVEVAVLCLIFFLA
LTGNLCVLLAIHTTRHKHSRMYYFMKHLSIADLVVAIFQVLPQLIWDITFRFYGPDFLCR
LVKYLQVVGMFASTYMLLLMSLDRCLAICQPLRSLHRRSDRLSVLLTWLLCLLVSIPQIH
IFSLRDVGNGVYDCWADFIQPWGLKAYITWITLTVYIIPVLMLSVCYGLISFKIWQNVKL
KTAHETNMSLTTNSSGAALSRVSSIKLISKAKIRTVKMTFIIVLAFIVCWTPFFFVQMWS
VWDKNAPQEASPFIIAMLLASLNSCCNPWIYMLYTGHLFHDLMQRFLCCSTRYLKSRQRC
DLSGSKKSNSSSFVLSRKSSSQRSFTQPSMA

#gi|557320901|ref|XP_006033575.1|_PREDICTED:_gonadotropin-releasing_hormone_II_receptor-like_Alligator_sinensis
MLELGSLVAGNQSCGEPQVTAGSLASGGFNASIPGEEVFLLPTFSTAAKVRVAITCVLFL
SSACFNIAVLWTVTRKYRKRPHVRILIVNLAAADLLVTFVVMPLDAVWNITVQWYAGDVA
CRALMFLKLVAMYASAFVTVVISLDRQAAILNPLGVGDAKKKNKAMLCVAWGLSVLLALP
QIFVFHTVSRSQPSYFIQCATVGSFSAHWQETLYNMFTFACLFLLPLLIMVLCYSRILLE
ISGKMKKACVSSQEIHLRRSYNNIPRARLRTLKMSIVIVFTFIVCWTPYYLLGIWYWFSP
DMLTREKVPPSLSHILFLFGLFNACLDPLIYGLFTVHFRREIWRACRCTRRGKEPEAASM
LTGSFRISTTAVPTRRAGEGHEGSGKYEMEVTAGAAPPSKRCELCRRRMMESFM

#gi|557755372|ref|XP_005177426.1|_PREDICTED:_gonadotropin-releasing_hormone_receptor-like_isoform_X1_Musca_domestica
MSEQEVNEKIYDHRVLTDWSNVNNNTNGTMHYSKDMIFNDGHRLSITVYSILFVISTIGN
STVLYLLTKRRLRGPSRIDIMLMHLAIADLMVTFLLMPLEIAWAYTVQWKSTDFMCRLMS
FFRVFGLYLSGFVLVCISIDRYYAILKPLKLSTNRGRIMLTIAWCSSVVCSLPQAYFFHL
EEHPKVSGYFQCVTFHSFPSEFHHIMYQIATMCAMYACPLITFIYCYGSIYLEIYRKSQR
IVKGIERFRRSNDDVLSRAKKRTLKMTITIVIVFLICWTPYYIIAMWYWFDKTSVDKVSA
LVSKSLFIFACTNSCMNPIVYGAFNIRGRIGNNASTISNRHTSLYNRGDSSNQLPKHLLN
LSEGSAKTGSTHINQTDTTDISINHKINETTTTANGCTEISNKVDEVSGSPVICINCVDS
IELSNRQKS

#gi|557755376|ref|XP_005177428.1|_PREDICTED:_gonadotropin-releasing_hormone_receptor-like_isoform_X3_Musca_domestica
MSEQEVNEKIYDHRVLTDWSNVNNNTNGTMHYSKDMIFNDGHRLSITVYSILFVISTIGN
STVLYLLTKRRLRGPSRIDIMLMHLAIADLMVTFLLMPLEIAWAYTVQWKSTDFMCRLMS
FFRVFGLYLSGFVLVCISIDRYYAILKPLKLSTNRGRIMLTIAWCSSVVCSLPQAYFFHL
EEHPKVSGYFQCVTFHSFPSEFHHIMYQIATMCAMYACPLITFIYCYGSIYLEIYRKSQR
IVKGIVAERFRRSNDDVLSRAKKRTLKMTITIVIVFLICWTPYYIIAMWYWFDKTSVDKV
SALVSKSLFIFACTNSCMNPIVYGAFNIRGRIGNNASTISNRHTSLYNRGDSSNQLPKHL

LNLSEGSAKTGSTHINQTDTTDISINHKINETTTTANGCTEISNKVDEVSGSPVICINCV
DSIELSNRQKS

#gi|557878657|ref|NP_001273664.1|_gonadotropin-releasing_hormone_receptor-like_Latimeria_chalumnae
MENNFKNGTQGKNCCAITNNSLPALKNYTDLPTLTISGKIRVIATMFLFLTSCILNVSFL
IKVYRWEKKNKSSRMKVLLKHLMLANLLETVIVMPLDGVWNITVQWYAGEFLCKVLNFLK
LFSMYAPAFMVVVISLDRCLAITRPFSVKNNNKFGKYNIHLAWTISLILAGPQIFVFGVI
DERYRADSDSMEIFFQCVTHKSFTEWWQQTFYNLFTFGCIFVTPLLIMLVCNFKIIFTMT
QVLRQDPSKMDLKRSKNNIPQARLKTLKMTIAFATSFIICWAPYYVLGIWYWFEPEILRW
VSDPVYHFFCLFGLLNPCFDPLIYGYFSL

#gi|557878688|ref|NP_001273667.1|_gonadotropin-releasing_hormone_II_receptor-like_Latimeria_chalumnae
MPATENQSQFMLTLNITSYTNNSSKPWTEPTFTVAARVRVIVTFCFFFMAACSNLAVLYS
VTKKRRKSHIRILILSLTIADLMITFIVMPLDAIWNTTVQWYAGDMACKVLNFLKLFAMY
SSALVLVVISLDRHSAILNPFSFAGASHRNKIMLSIAWVTSVLLASPQLFIFRLHTVKNE
NFTQCVTYGSFKKHWQETVYNMFTFTTLYITPLTVMMICYARILWEISKQMKDDKALARS
KSDHFSKARMKTLKMTIVIVASFIVCWTPYYLLGLWYWFQPGMIQKTPEYVNHTLFLFGL
LHTCSDPIIYGLYTPSFREDMMMCLKRIDRGIRRHKNSYNQRSDFMLNLKDNLNGEAFDN
IKVGSLTSNGTMVQSIF

#gi|557950089|gb|AHA44513.1|_GnRH_receptor_type_IIa-1_Ambystoma_mexicanum
MNSTQALGVIYCSNFILLNTTCALDTRDYWHNGSHGSFQLPTFSSAAKARVIITFAIFVI
SAFCNLAVLWAAARTSRKKRSHVRILILNLTAADLLVTFVVMPLDAVWNITVQWQAGDLA
CRFLMFLKLLAMYSCAFVTVVISIDRQSAILNPLAISDAKKKNRIMLYVAWLMSTVLSLP
QLFLFHTVTITAPQNFTQCTTRGSFQEHWQETGYNMVSFVCLFLLPLLIMISCYSRILLE
ISRRMAKGTISSKEVYLRRSKNNIPKARMRTLKMSIVIVSSFIICWTPYYLLGLWYWFYP
DTMEGKVSQSLTHILFIFGLFNACLDPITYGLFTIHFRKGLQRYCGRAKMISDHETPSSI
TGSFRCSMSSFRIKRSTVVTQEMPSVNGSQNYLQVRTNGLSSSCL

#gi|557950091|gb|AHA44514.1|_GnRH_receptor_type_IIa-2_Ambystoma_mexicanum
MNISVFSPMMCGVKGLNQSCKHVKDQAALWPRPNITFPSEESFVLPTFSTAAKVRVAITC
ILFVSSTCFNLATLWTITHKHRKKSHIRILIINLAMADLLVTFIVMPLDAIWNVTVQWYA
GDLACRLLMFLKLVAMYASAFVTVVISLDRQSAVLNPLAIGEAKKKNKTMLCVAWILSIL
LAIPQLVVFHTVSRSQPVYFVQCATVGSFQAHWQETLYNMFTFACLFLLPLLIMVLCYSR
ILREISRKMKKACAVSSKEVHLRRSYNNIPKARMRTLKMSIVIVLTFIVCWTPYYLLGIW
YWFSPEMLTREKVPPSLSHILFLFGLFNACLDPVIYGLFTIHFRREIRRVCRCAKHATDA
DTASMATGSFRVSTTAVPIKKALGCQDGSGRFELEVTGVGIHNGKCEHCKGKVAESFM

#gi|557950093|gb|AHA44515.1|_GnRH_receptor_type_IIb_Ambystoma_mexicanum
MSVTLYPTNHTQRTLSPSSDRSATGLAEPWLEPAFTAAARIRVVVTCCFFLASASSNTAV
LISILRKRRKSHIRVLILSLTVADLLVTALVMPLDAVWNVTLQWYAGALLCKLLNFSKLF
SMYAAALVLVVISLDRHSAILYPFSFTGASRRNRVMLCVAWVLSILLASPQLFLFRLHTI
QSANFTQCVTHGSFKERWQETTYNLFTFFSLFVTPLSVMVVCYARILWEISRQMKDKKEL
ARSKSDHISKARLKTLKMTVVIVASFMLCWAPYYLLGLWYWFHPEMIHETPEYIHHSLFL
CGLLHTCSDPVIYGLYTPSFREDLRTGLRRVSSALTGCKKAQKELSGSELNIKDGATDMP
SSGSTVQTVF

#gi|558190133|ref|XP_006129246.1|_PREDICTED:_gonadotropin-releasing_hormone_II_receptor-like_Pelodiscus_sinensis
MTPPQSADVAEKNLSVSGCPEPWIEPTFTLAAKARVIVTICFFLIAACSNSVVLYSIMRK
RRKSHVRLLILSLTVADLLVTFTVMPLDAVWNVTVQWYAGDLSCKLLNFLKLFAMYSAAL
VLVVISIDRHSAILHPFAFASSNRRNRLMLCVAWGMSFLLASPQLFLFHLHTVPGVNFTQ
CVTHGSFQAHWEETLYNMFTFTTLYVAPLSVMIVCYIRVIWEVSKQLKINQGLATGKNNH
ISKARLKTIKMTIVIVASFVICWTPYYLLGLWYWFQPGMIQSMPEYVNHSLFLFGLLHTC
TDPVIYGLYTPSFREDMKMCLRGIETVITGQERNKLMSSSEMNIKDCTANSGAVSGASHG

TAIHTVC

#gi|558215982|ref|XP_006134742.1|_PREDICTED:_gonadotropin-releasing_hormone_II_receptor-like_isoform_X1_Pelodiscus_sinensis
MNATFRRKVGELFEPESPTVGNQTRGQPWEELEVSSGDGANTSLPSEEVFLLPTFSTAAK
VRVAITLVLFLSSACFNIAVLWTVTQKYRKRPHIRILIVNLATADLLVTFVVMPLDAAWN
ITVQWYAGDLACRALMFLKLVAMYASAFITVVISLDRQAAILHPLSMGDAKKKNKAMLCG
AWALSVLLALPQMFVFHTVSRSQPIYFIQCATVGSFHAHWQETLYNMFPFSCLFLLPLLI
MVLCYSRILIEISGKMKRACGEPGQPPAALSTASSQEVHLRRSYNNIPRARMRTLKMSIV
IVLTFVLCWTPYYLLGLWYWFSPEMLSRKKVPPSLSHILFLFGLFNTCLDPLIYGLFTMH
FRREIRRACPCSHRRKEQDAGSTLIGSFRASTTAVPIRRAGEDEGGSGKYELEVTAHMGL
PAGRCELCRRKTVESFI

#gi|558215985|ref|XP_006134743.1|_PREDICTED:_gonadotropin-releasing_hormone_II_receptor-like_isoform_X2_Pelodiscus_sinensis
MNATFRRKVGELFEPESPTVGNQTRGQPWEELEVSSGDGANTSLPSEEVFLLPTFSTAAK
VRVAITLVLFLSSACFNIAVLWTVTQKYRKRPHIRILIVNLATADLLVTFVVMPLDAAWN
ITVQWYAGDLACRALMFLKLVAMYASAFITVVISLDRQAAILHPLSMGDAKKKNKAMLCG
AWALSVLLALPQMFVFHTVSRSQPIYFIQCATVGSFHAHWQETLYNMFPFSCLFLLPLLI
MVLCYSRILIEISGKMKRACASSQEVHLRRSYNNIPRARMRTLKMSIVIVLTFVLCWTPY
YLLGLWYWFSPEMLSRKKVPPSLSHILFLFGLFNTCLDPLIYGLFTMHFRREIRRACPCS
HRRKEQDAGSTLIGSFRASTTAVPIRRAGEDEGGSGKYELEVTAHMGLPAGRCELCRRKT
VESFI

#gi|560892585|ref|XP_006172806.1|_PREDICTED:_gonadotropin-releasing_hormone_II_receptor-like_Camelus_ferus
MSAGNGTPSGSAAGEVWAGSGVEVEGSELPTFSVAAKVRVGVTVVLFISSAGGNLAVLWS
VTRPQPSQLRPSPVRRLFAHLAAADLLVTFVVMPLDATWNITVQWLAGDIACRTLMFLKL
MAMYAAAFLPVVIGLDRQAAVLHPLGPRSTGGKLLGAAWGLSFLLALPQLFLFHTVRRAG
PVPFTQCVTKGSFKAQWQETTYNLFTFCCLFLLPLTAMTICYSRIVLSASSSRTRKGNHA
SAGEFALRRSLDNHPRVRLRALRLALLVLLTFILCWTPYYLLGLWYWFSPTMLNEVPPSL
SHILFLFGLLNAPLDPLLYGAFTLGCRRGHQELSIDSSREGGSGRKPPQEIQALRQMEIQ
ANVAAKEAGETKETFL

#gi|560980508|ref|XP_006212503.1|_PREDICTED:_gonadotropin-releasing_hormone_II_receptor-like_Vicugna_pacos
MSAGNGTPSGSAAGEEVWAGSGVEVEGSELPTFSVAAKVRVGVTVVLFISSAGGNLAVLW
SVTRPQPSQLRPSPVRRLFAHLAAADLLVTFVVMPLDATWNITVQWLAGDIACRTLMFLK
LMAMYAAAFLPVVIGLDRQAAVLHPLGPRSTGGKLLGAAWGLSFLLALPQLFLFHTVHRA
GPVPFTQCVTKGSFKAQWQETTYNLFTFCCLFLLPLTAMTICYSRIVLSVSSSRTRKGNH
ASAGEFTLRRSLDNRPRVRLRALRLALLVLLTFILCWTPYYLLGLWYWFSPTMLNEVPPS
LSHILFLFGLLNAPLDPLLYGAFTLGCRRAHQELSIDSSREGGSGRKPQQEIQALRQMEI
QANVAAKEAGETKETSL

#gi|56112299|gb|AAV71128.1|_gonadotropin-releasing_hormone_receptor_I_Acanthopagrus_schlegelii
MDTTLCDSAAAMYHLTTDHQLNASCNYSTPPSNWTAGGGGQQLPTFTTAAKVRVVITCIL
CGVSAFCNLAVLWAAHSDGKRKSHVRVLIINLTVADLLVTFIVMPVDAVWNITVQWLAGD
FACRLLMFLKLQAMYSCAFVTVVISLDRQSAILNPLAINKARKRNRVMLTVAWGMSVLLS
VPQMFLFHNVTIVHPEDFTQCTTRGSFVRHWHETAYNMFTFFCLFLLPLVIMITCYTRIF
CEISKRLRKDNLPSNEVHLRRSKNNIPRARMRTLKMSIVIVSSFIICWTPYYLLGLWYWF
FPDDLEGKVSHSLTHILFIFGLVNACLDPVIYGLFTIHFRTGLRRYYRNATTASDLDNNT
VITGSFVCAANSLQLKREVSPVSQERFMLCSDSHSREESTPSRSSFLTADNDAEKDSHQF
CSDSII

#gi|564236205|ref|XP_006275012.1|_PREDICTED:_gonadotropin-releasing_hormone_II_receptor-like_Alligator_mississippiensis

MEENVSISSCSETWIEPTFTLAARVRVIITICFFLVAVCSNSAVLYSILRKRRKSHVKLL
ILSLTAADLLVTFTVMPLDAVWNVTVQWYAGDLLCKLLNFLKLFAMYSAALVLVVISIDR
HSAILHPFTFASSSRRNRLMLCVAWVMSLFLASPQLFLFHLHTVPGVNFTQCVTHGSFRE
HWEETAYNMFTFTTLYVTPLSVIIVCYIRIIWEISKQLKINKGLARSKDDHISKARMKTL
KMTIVIVATFIICWTPYYLLGLWYWFQPDMIQRMPEYINHSLFLFGLLHTCTDPVIYGLY
TPSFREDMKMCLRGIETVFTGQERHKPISSSEVNFKDYVANGGAASGGS?GTTVHTVC

#gi|568259533|gb|ETN67429.1|_gonadotropin-
releasing_hormone_receptor_Anopheles_darlingi
MQASNTVAVLLVDLRNDSSLQQRSNRMLEIMPHAKLNHRIEDHRNLDDWSFYANQSVEPD
GMPIDMRFNSGHILSIMVYSTLMVFSATGNIKVLSILAQRKVRATSRINIMLAHLAIADL
LVTFLMMPLEIGWAYTVMWNAGDLLCRVMAFFRTFGLYLSSFILICISIDRYFAVLQPLK
VHPHRAMLMISGAWFMSGLCSLPQSFIFHVETHPNYTEFYQCVTYHFFEEEIYEIIYNVL
GMCFMYALPLVVILYCYGSIYYEIFSRTNPRNLESFRRSSIDVLGRAKRKTLRMTITIVI
VFVVCWTPYYVMSLWYWLDKESAKNVDQRIQKALFLFACTNSCMNPVVYGIFNVRKKRTK
STVKLQEQRSCGSNLTMKV

#gi|570362329|gb|AHE78444.1|_putative_gonadotropin_releasing-
hormone_receptor_Aplysia_californica
MDGTDSDGSILQRANQAFLASSSGPLQLPIPYNFSAQNSSVHRKRHDVYSPRSAHHDFIL
ASDNISSLLLNTTAPNMISNNSSFEFQFLNTTHPSNVTSENAPTFTTTSLIKTIVFGIMF
GISFVGNMATIVQMRRLRRRKSTINTLIVNLALADLLVTFFCIAGEAAWTVTVQWLAGSV
MCKFVKYMQVFALYLSTYITVAISLDRCVAILDPMRRKSAAQRVRTMIVFAWIFSALFSI
PQPIVFNVLRGPFKEVFYQCVTFGSYDSAWQLQMYAIASLMLMFVLPLAVMGTAYGLIFT
TISRKSKEHSVMDSIQMPARKTWVTRWVNYKRCLCMAFCSKPISRRSSIMSNYSEDHAAR
GPVRSYLLRKAKRKSLIMSFVIVLAFMVCWTPYYIIFICITFLDKVIDPVIFNYFSFIGL
SNSMLNPMIYGAFQLCKVQFYNPRFVLRLTGRLPCTFLLRCMVAVRSAYPPFI

#gi|572262443|ref|XP_006609474.1|_PREDICTED:_gonadotropin-
releasing_hormone_II_receptor-like_Apis_dorsata
MGSSIKIITTTELNNSRVNNSNYTELLPIDMRFNEGHIISIVFYSVLMIISAIGNTTVLI
LITCRKRVSKSRIHIMLMHLAIADLLVTFLMMPLEIGWAITVSWKAGDVMCRIMAFFRIF
GLYLSSFVLVCISMDRYYAVIKPLQLWDVDKRGKIMLSFAWIGSIVCSLPQTIVFHLETH
PNITWYSQCVTFNAFPTYTHEITYSLFGMIMMYWFPLVVIIYTYTSILLEIRRRSKKSED
GKIRRSSIGFLTRAKIRTLKMTVIIIAVFFVCWTPYYVMSLWYWIDRNSAYKIDQRIQKG
LFLFACTNSCMNPIVYGAFNIRDRNKTSARPTTIETRVTPLSLSLKLLD

#gi|572312496|ref|XP_006621964.1|_PREDICTED:_gonadotropin-
releasing_hormone_receptor-like_Apis_dorsata
MTIPNNYTTYMLPACDNLTNFFNHSRDLTLDSRLWNTSELSHPFHSYNLKNITCLEHAPY
SNSNTFFKSLMLTIIAVTSILANLATIYSIIRCRRRHHSWSAIYTLILHLAVADLFVSVF
CIGGEAMWNYTVEWIWGNVACKLFKFLQVFSLYLSTFVLVLIGVDRFFAIRYPMKGMNTA
DRCLKFIIVVWILSFVLSLPQIIIFHVAQGPFVEKFEQCVTYGFYTEPWQEQLYVSFGLF
SMFLLPLGILIATYVFTIITISRSERMFKVKLANNDICHVNGDVNRRKLMYRAKTKSLRI
SIVIVTAFIFWWTPYYTMMIIFMFSSPDKHFSDELQNIIFFFGMSNSLVNPLIYGAFHLW
PRKRRNFMHREISTTQRRFTPTSSYRRDSRETRTPILPKNY

#gi|5733822|gb|AAD49750.1|AF174481_1_gonadotropin-
releasing_hormone_receptor_Typhlonectes_natans
MNSTFSSEDRDPTHLAAINHSWGPVEVAAETTRLNTTHHSEEVFVLPTFSTAAKVRVTIT
FVLFISSACFNIIALWTITQKYKKRSHVRILISNLAVADLLVTFIVMPLDAIWNITVQWY
AGDLVCRVLMFLKLVAMYASAFVTVVISLDRQSAILNPLGIGDAKKKNKIMLCVAWVLSV
LLAVPQLFVFHAVSPSQSEYFIQCATVGSFQGHWQETLYNMFTFSCLFLLPLLIMVLCYS
RILIEISRKMKKACVSSKEVHLRRSSNNIPKARLRTLKMSIVIVLTFIVCWTPYYLLGIW
YWFSPEMLTRERVPPSLSHILFLFGLFNACLDPLIYGLFTIHFRREIRRVCRCRKGVKEL
NIATGSFRVSTSAVPTGKASGAQNGLEVTGLNLQLGKCEQCRRKMAESFL

#gi|573881705|ref|XP_006629045.1|_PREDICTED:_gonadotropin-releasing_hormone_II_receptor-like_Lepisosteus_oculatus
MYAPEMNETLRGHELMMFLYSPGYQANSSCDTSSMPLCTRNGTGEVLQLPTFSTAAKARV
IITFILCIVSAICNLAVLWTASTNTKRKSHVRILIINLTVADLLVTFIVMPVDAVWNITV
QWLAGDVSCRLLMFLKLLAMYSCAFVTVVISLDRQSAILNPLAINKAKKKNKIMLSVAWA
MSAFLSVPQMFIFHNVTITVPQNFTQCTTRGSFSKHWQETVYNMFTFVCLFLLPLAIMIF
CYTRILMEISKRMTKGNISSKEVHLRRSKNNIPKARMKTLKMSIVIVSSFIVCWTPYYLL
GLWYWFSPDVLEETISHSLTHILFIFGLFNACLDPITYGLFTIHFRKGLKRYCRSATVLV
ESENNTTLTASFRCSLSSFRMKRRTPLGQDAPSANKEPESKTSSPFNNYLTVYHDSKSDR
NQIDPVNMI

#gi|573885740|ref|XP_006630737.1|_PREDICTED:_isotocin_receptor-like_Lepisosteus_oculatus
MEDILFKDQDLWSVNASLENSSLANATREANGTVNPVLRNEEVAKVEVTVLALILLLALT
GNLCVLLAIYTTKNNQSRMYFFMKHLSIADLVVAIFQVLPQLIWDITFRFYGPDFLCRLV
KYLQVVGMFASTYMLVLMSIDRCLAICQPLRSLHRRADRFYIITSWVLSLLFSIPQVYIF
SLREIPAGSGVYDCWGDFIQPWGAKAYVTWISLTIYIIPVAILSVCYGLISFKIWQNFKL
KTRKDQSIPLTPKAFKGSALSRVSSVKLISKAKIRTVKMTFVIVLAYIVCWTPFFFVQMW
SAWDPAAPREELAFIIAMLLASLNSCCNPWIYMFFAGHLFHDLMQHFLCCSASYLKATQC
SCELDSSRKSNSSTYIIKNSSSQKSITQTSST

#gi|573908352|ref|XP_006641895.1|_PREDICTED:_gonadotropin-releasing_hormone_II_receptor-like_Lepisosteus_oculatus
MSGNLSLFLMPAPDITGNSSNATAPPAGDWQEPTFTRAAQFRVGATLVLFLFAAVSNLAV
LASVAGGRGRRLASHLRPLILSLAAADLMMTFIVMPLDAIWNVTVQWYGGDALCRLLCFL
KLFAMQASAFILVVISLDRHHAILRPLDSFDANRRNKRMLVLAWSASFLVASPQLFIFRA
IKAETADFTQCVTHGSFRQRWQETLYNMFHFVTLYVVPLLVMSCCYARILAEINRQMKKS
KGGEASLRRSGTDQIPKARMKTLKMTIIIVASFIICWTPYYLLGIWYWFQPQMLRDTPEY
VNHILFLFGNLNTCCDPVIYGLYTPSFRADLAACWCRRPQDSPRSLERLSSRRTPHSAAL
ESKSESPATEHQSATRG

#gi|578359002|gb|AHI15744.1|_vasotocin_V1_hormone_receptor_1_Petromyzon_marinus
MPGNVTGEMEGAILNGTGVAYAPATSNGSHVVPAEAFTAALASINATLNGSSQHALDRNE
EVAKVEIALLSIILFVAIVGNVCVLLALINTRKKTSRMHLFIMHLSIADLVVAFFQVLPQ
LIWKITYRFNGSDFLCRAIKYLQILGMFASTYVLIMMGLDRYIAICHPLRTLRQSSKQSY
QMIFVSWFLSMLFSLPQAFIFSMSEVENSGIIDCWAEFIKPWGTKAYITWMTGSVFIIPV
IILIWCYGMITFAIWKNIKAKTQEGDSRHNPAKSSAPSRVSSVRSISKAKIRTAKMTFVI
IMVYIICWTPFFFVQMWSVWDSSAPFEGIPFAIVMLLASLNSCTNPWIYMFFSGHLLYDF
VRYFPCGARSRARSREADESRASDSSRRNHTFVSRLTRRSLTLSSGSQHEEASSRTTSLS
PVARVPKTYFA

#gi|583968646|ref|XP_006779777.1|_PREDICTED:_gonadotropin-releasing_hormone_II_receptor-like_isoform_X1_Neolamprologus_brichardi
MNGSSCCDPAAVMYQQRSGLDLNASCEWPDPHCNWTSVDGALQLPTFSTAAKIRVIVTFI
LCGISTFCNLAVLWAANGHKRKSHVRVLIINLTAADLLVTFIVMPVDAVWNITVQWLAGD
LACRFLMFLKLQAMYSCAFVTVVISLDRQSAILNPLGIAMVRKRNRVMLMVAWIMSALLS
IPQMFIFHNVTITYPANFTQCTTRGSFVTHWQETAYNMFTFCCLFLLPLVIMIICYTRIF
VQISKQMTKKNMPSNEPHLRCSKNNIPKARMRTLKMSIVIVICFIVCWTPYYLLGLWYWF
FPDDLEGKVSHSLTHILFIFGLFNACLDPIIYGLFTIRFQKGLRNCYRKAAVMSSLETNA
VIMESLKCTGSVLPSKRGMTSGEKDISSEQAEAKSTDNSV

#gi|583968648|ref|XP_006779778.1|_PREDICTED:_gonadotropin-releasing_hormone_II_receptor-like_isoform_X2_Neolamprologus_brichardi
MNGSSCCDPAAVMYQQRSGLDLNASCEWPDPHCNWTSVDGALQLPTFSTAAKIRVIVTFI
LCGISTFCNLAVLWAANGHKHAVWNITVQWLAGDLACRFLMFLKLQAMYSCAFVTVVISL
DRQSAILNPLGIAMVRKRNRVMLMVAWIMSALLSIPQMFIFHNVTITYPANFTQCTTRGS
FVTHWQETAYNMFTFCCLFLLPLVIMIICYTRIFVQISKQMTKKNMPSNEPHLRCSKNNI

PKARMRTLKMSIVIVICFIVCWTPYYLLGLWYWFFPDDLEGKVSHSLTHILFIFGLFNAC
LDPIIYGLFTIRFQKGLRNCYRKAAVMSSLETNAVIMESLKCTGSVLPSKRGMTSGEKDI
SSEQAEAKSTDNSV

#gi|583975629|ref|XP_006783187.1|_PREDICTED:_isotocin_receptor-like_Neolamprologus_brichardi
MEDLLREQYSWSHNLTWSSSSRENESYVGNTTVNPLKRNEEVAKVEVTVLVLVLLLALTG
NLCVLWAIHTTKHSQSRMYYFMKHLSIADLVVAIFQVLPQLIWDITFRFYGPDLLCRLVK
YLQVVGMFASTYMLVLMSIDRCLAVCQPLRSVHKKKDRFCVIASWMLSLIFSSPQAYIFS
LREVGNGVYDCWGDFVQPWGAKAYITWMSFSIYIFPVAILSICYGLICFKIWENFNLKTR
REHFLALTPRPSKGAQPFSRVSSVRLISKAKIRTVKMTFVVVLAYIVCWTPFFFVQMWSA
WDPAAPREDMAFIIVMLLASLNSCCNPWIYMFFAGHLFHDLMQCFFCCCRRYLTECSCSC
GQQCRHKRGSSTYVNKNTKSQRSLSRTSSTVH

#gi|583988407|ref|XP_006789425.1|_PREDICTED:_gonadotropin-releasing_hormone_II_receptor-like_Neolamprologus_brichardi
MYHQLTDQTVNGSCQGPTSACNKSADGDALELPTFSTAAKVRVIITFALCAVSAVCNLAV
LWAASSGGRRKSHVRILIMNLTVADLLVTFIVMPVDAVWNITVQWQAGDVACRLLMFMKL
VAMYSCAFVTVVISLDRQSAILNPLGISEAKRKSKVMLAVAWTMSVILSLPQMFIFRNVT
ITVPEKFTQCTTHGSFVQRWQETLYNMFTFVCLFLLPLAIMIFCYTRILIEISSRMARNN
FLSRDVHLRRSHNNIPKARMRTLKMSIVIVTSFIICWTPYYLLGLWYWLFPEKMEKTVSH
SLTHMLFIFGLFNACLDPITYGLFTIHFHKGMRRCRQSSNARTELENNTRLVQMIRLSSR
RQIASDVHSASTEVVSESNIMKHVSNPDMSISKI

#gi|584076164|ref|XP_006758278.1|_PREDICTED:_oxytocin_receptor_Myotis_davidii
MEGTPEANWSAEVVNGSAAPPGAEGNRTQAPQRNEALARVEVAVLCLILFLALSGNACVL
LALRTTRHKHSRLFFFMKHLSIADLVVAVFQVLPQLLWDITFRFYGPDLLCRLVKYLQVV
GMFASTYLLLLMSLDRFTWLGCLVASAPQVHIFSIREVADGVFDCWAGFIQPWGPKAYVT
WITLAVYIVPVIVLTACYSLICFKIWQNFRIKTAAEGAAKGPEGAMPGSREGTALARVSS
VKLISRAKIRTVKMTFIIVLAFIVCWTPFFFVQMWSVWDANAPKEASAFIIAMLLASLNS
CCNPWIYMLFTGHLFHELLQRILCCSSSYRRDSRPGETSVSKKSNSSTFVLSRHSSSQRS
TSQLSSL

#gi|584600523|gb|AHJ11243.1|_G_protein-coupled_receptor_Nilaparvata_lugens
MNVSAGGDELQGSQNGSLSECGPQQWPHLQLFPHWHQWCVNSTFTFDFPIDMQFNDGHRL
SIIAYSTLMLLSAIGNFTVLSILIRRRRNNINNMLIHLAIADLLVTFLLMPLEIAWSATV
MWWAGDAWCRVTAFFRTFGLFQSSFVLVCISIDRYFAVLKPMNHLSDVDRRGKIMLSCAW
IGSVLCSLPQMLVFRVQAHPYVPWFEQCITIWFAQDAKNPSVQEFVYFVFGMLMMYCIPF
LVILFCYASILGEIYRHLREDKSDRFRRSSLGFLGKAKGRTLKTTVIIVVVFLICWTPYY
FMSLWHFFDHKGSQKVDQKLQKGLYIFACTNSCMNPIVYGAFNIRPRRTDRRGGAAGGVG
GQRRRPIHQLSDDSIQISRLSSTVSNGSQTQRNDTKQSVVFSL

#gi|585709379|ref|XP_006899575.1|_PREDICTED:_gonadotropin-releasing_hormone_II_receptor-like_Elephantulus_edwardii
MSAGNGTPWVSAVGEEVWAGSGVEVEESELPTFSAAAKVRVGVTIMLFVSSAGGNLAVLW
SVTRPQPSQVRPSPVRRLFAHLAAADLLVTFVVMPLDATWNITVQWLAGDIACRTLMFLK
LMAMYAAAFLPVVIGLDRQAAVLHPFGPRSGGKKLLGAAWGLSFLLALPQLFLFHTVRRA
GPVPFTQCVTKGSFKAQWQETTYNLFTFCCLFLLPLTVMAICYSRIVFSVSSPRTRKGSH
APASGFALRRSLDNRPRVRLRALRLALLVLLTFVLCWTPYYLLGLWYWFSPTMLTEVPPS
LSHILFLFGLLNAPLDPLLYGAFTLGCRRGNPELSTDSTSQRGSGRMSLRQLEAHTSVAA
KRAGGTKETFL

#gi|586461491|ref|XP_006861394.1|_PREDICTED:_gonadotropin-releasing_hormone_II_receptor-like_Chrysochloris_asiatica
MSGRNGTPWGSAAGEEVWAGSGVEVEESELPTFSAAAKIRVGVTIVLFVSSAGGNLAVLW
SVTRPQPSQVRPSPVRRLFTHLAAADLLVTFVVMPLDATWNITVQWLAGDIACRILMFLK
LMAMYAAAFLPVVIGLDRQAAVLHPFGPRSGGRKLLGAAWGLSFLLALPQLFLFHTVRRA

GPVPFTQCVTKGSFKAQWQETTYNLFTFCCLFLLPLTAMVICYSRIVLSVSSPRTRKGNH
APADGFALRRSLDNRPRVRLRALRLALLVLVTFVLCWTPYYLLGLWYWFSPTMLTEVPPS
LSHILFLFGLLNAPLDPLLYGAFTLGCRRGHQELGTDSSREGGSGRMPLRQLEAHTNVAS
SRAGETKETFL

#gi|586521099|ref|XP_006916576.1|_PREDICTED:_gonadotropin-releasing_hormone_receptor_isoform_X1_Pteropus_alecto
MANSASSEQNQNHCSAVNSSNLLVQDNLPTLTISGKIRVTVTFFLFLLSTTFNASFLLKL
QKWTQKKEKGKKLSRMKVLLKHLTLANLLETLIVMPLDGMWNITVQWYAGELLCKVLSYL
KLFSMYAPAFMMVVISLDRSLAITRPLAVKSNKLSQSNIGLAWLLSSLFAGPQLYIFRMI
HLADDSGQTGGFSQCVTHCSFPQWWHQAFYNFFTFSCLFTIPLLIMLICNAKIIFALTQV
LHQDPHQLQLNQSKNNIPRARLRTLRMTVAFATSFTVCWTPYYVLGIWYWFDPEMLNRVS
DPVNHFFFLFAFLNPCFDPLIYGYFSL

#gi|586525364|ref|XP_006917636.1|_PREDICTED:_oxytocin_receptor_Pteropus_alecto
MEGALLANWSVEAVNGSAPPPVVEGNLTAGPPQRNEALARVEVAVLSLILFLALSGNACV
LLALRITRHKHSRLFFFMKHLSIADLVVAVFQVLPQLLWDITFRFYGPDLLCRLVKYLQV
VGMFASTYLLLLMSLDRCLAICQPLRALNRRADRLAVLATWLGCLVASVPQVHIFSLREV
AEGVFDCWAVFIQPWGPKAYVTWITLVVYIVPVIVLAACYGLICFKIWQNFRLKTAAAAV
EGPEGAAASSGERAALARVSNVKLISKAKIRTVKMTFIIVLAFIVCWTPFFFVQMWSVWD
ADAPTEASAFIIAMLLASLNSCCNPWIYMLFSGHLFHELMQRFLCCSCGYLKGPRPGETS
VSKKSNSSTFVLSRHSSSQRSCSQPSSV

#gi|590001401|gb|AHL26526.1|_gonadotropin-releasing_hormone_receptor_4_Salmo_salar
MSHEIMMFYQMMEQAQNTSCEAPTPICNKSASGDSLQLPTFSTAAKVRVIITFTLCAMSA
VCNLAVLWAANTNGKRKSHVRILIINLTVADLLVTFIVMPVDAVWNITVQWQAGDVACRL
LMFMKLVAMYSCAFVAVVISLDRQSAILNPLAINEAKKRSKIMLSVAWVMSVILSVPQML
IFHSVTITVPEKFTQCTTHGSFVQHWQETLYNMFTFACLFLLPLIIMIFCYTRILVEISS
RMTHGNMSSKEIHLRRSHNNIPKARMRTLKMSIVIVTSFIVCWTPYYLLGLWYWFFPDDM
DETVSHSLTHMLFIFGLFNACLDPITYGLFTIHFRHGLKRYCRSTATFSRESENNTTLTG
SFRRSPFRLTRLTQQGQTSITGQMAEEEQVKKTCRYSNYLTVHSSGEGDPGPTSPESTI

#gi|591357026|ref|XP_007053591.1|_PREDICTED:_gonadotropin-releasing_hormone_II_receptor-like_Chelonia_mydas
MDRLQGSHTKDGLLCLILLSVVCVVLTLLSALVPGNKQITVAALKQVHAGMPEERDLMTP
PQSADVVDENLSASDCPEPWIEPTFTLAARVRVIVTICFFLIAACSNSVVLYSIMRKRRK
SHVRLLILSLTVADLLVTFTVMPLDAVWNVTVQWYAGDLPCKLLNFLKLFAMYSAALVLV
VISIDRHSAILHPFAFANSSRRNRLMLYVAWVMSLLLASPQLFLFHLHTVPGVNFTQCVT
HGSFQEHWEEILYNMFTFTTLYVAPLSVMIVCYIRIIWEISKQLKINKGLARSKNNHISK
ARMKTLKMTIVIVASFIICWTPYYLLGLWYWFQPDMIQSMPEYINHSLFLFGLLHTCTDP
IIYGLYTPSFREDMKACLRGIETVITGQERNKLMSSSEMNIKDYIINGGAASGASNGTII
HTVC

#gi|591373269|ref|XP_007061334.1|_PREDICTED:_mesotocin_receptor-like_Chelonia_mydas
MEKLSLEGTDLWTINGSLINCSLSLENQTYGVNSTTDPLKRNEDMAKVEVTVLCLILFLA
LTGNLCVLLAIHTTRHKHSRMYYFMKHLSIADLVVAIFQVLPQLIWDITFRFYGPDFLCR
LVKYLQVVGMFASTYMLLLMSLDRCLAICQPLRSLHRRSDRVSVLLTWLLCLLVSIPQIH
IFSLRDVGNGVYDCWADFIQPWGAKAYITWITLTVYIIPVLMLSVCYGLISFKIWQNVKL
KTAHETNINLSSSGAALSRVSSIKLISKAKIRTVKMTFIIVLAFIVCWTPFFFVQMWSVW
DKNAPKEASPFIIAMLLASLNSCCNPWIYMLFTGHLFHDLIHRFLCCSTHYLKSRQGCDL
SISKKSNSSTFALSHKSSSQKSFTQPSTA

#gi|591380185|ref|XP_007064636.1|_PREDICTED:_gonadotropin-releasing_hormone_II_receptor-like_Chelonia_mydas
MNATFRMKVSELLEPGLPAVGNQSRGQPWEEVEVSPGGGANASLPSEEVFLLPTFSTAAK

VRVAITFVLFLSSACFNIAVLWTVTQKYRKRPHVRILIVNLAAADLLVTFVVMPLDAAWN
ITVQWYAGDLACRALMFLKLVAMYASAFITVVISLDRQAAILHPLSMGDAKKKNKAMLCV
AWALSVLLALPQMFVFHAVSRSQPIYFIQCATVGSFQAHWQETLYNMFTFSCLFLLPLLI
MVLCYSRILIEISGKMKRACGEPGPPLASPGPLSSKEVHLRRSYNNIPRARMRTLKMSIV
IVLTFVLCWTPYYLLGLWYWFSPEMLSRKKVPPSLSHILFLFGLFNACLDPLIYGLFTMH
FHREIRCVCRCTHRRKEQASALIGSFRASTTAAPIRSAREDQGGTGKYELEVTANMALPA
GRCELCRKKIVESFI

#gi|597781955|ref|XP_007254537.1|_PREDICTED:_gonadotropin-
releasing_hormone_II_receptor-like_Astyanax_mexicanus
MNDISPSTATMFHQLTESAPNDSCYYSECNSTTGEAALQLPTFSVAAKARVIITFTLCAV
SAACNLAVLWAASTSTRRKSHVRILIINLTVADLLVTFIVMPVDAAWNITVQWLAGDFAC
RLLMFLKLVAMYSCAFVTVVISLDRHSAILNPLAISEAKKKSKIMLSAAWAMSIVLSVPQ
MFIFHTVTITVPANFTQCTTRGSFVKHWQETLYNMFTFVCLFLLPLVIMIFCYTRILLEI
SQRLSKGNISSKEVHLRRSNNYIPKARMRTLKMSIVIVTSFIVCWTPYYLLGLWYWFLPD
DLEETVSHSLTHMLFIFGLFNAILDPITYGLFTIHFRKGLKRYCRGATALNELENNTVLT
GSLKCSPSPLCKKGLQRPSQKTKALEGREGKKSGSQRSNLVMHEMEGGQPNLSTQESVI

#gi|602663397|ref|XP_007437330.1|_PREDICTED:_gonadotropin-
releasing_hormone_II_receptor-like_Python_bivittatus
MDTTLWGQGRSNSTEEIFSPGEVPHGSFQLPTFSTAAKIRVAVTVALFLCSTGTNVAMLR
SVTQKFQHKPHLRILLVNLAAADLLVTFVVMPLDAVWNVTVQWYAGDLACRLLMFLKLVA
MYACSFVTVVISLDRWAAVLHPLRVSRAKRKSKAMLGVAWALSFILAIPQMFVFHTVSRS
QPIHFVQCATVGSFEAHWQEACYNLLTFSCLFLLPLLVMVLCYACIFAEISRKMARANAS
PKSKEVPLRCSRNNIPQIRVRTLKMSAITVLTFVACWTPYYMLGLWYWFSPEMLTREQVP
PSLSHIFFLFGLFSTCLDPLVYCFCSARFRSGSRQPKKGPPSLPMASFQASIVSSCPVAE
LGGD

#gi|602729630|ref|XP_007450240.1|_PREDICTED:_gonadotropin-
releasing_hormone_receptor_Lipotes_vexillifer
MANSASPEQNQSHCSAINSSIPLTQGNLPTLTLSGKIRVTVTFFLFLLSTTFNASFLLKL
QKWTQRKEKGKKLSRMKVLLKHLTLANLLETLIVMPLDGMWNITVQWYAGELLCKVLSYL
KLFSMYAPAFMMVVISLDRSLAITRPLAVKSNKKLGQLMIGLAWLLSSIFAGPQLYIFRM
IHLEDDSGQTEGFSQCVTHCSFPQWWHQAFYNFFPFPCLFIIPLLIMLICNAKIIFTLTR
VLHQAPHKLQLNQSKNNIPQARLRTLKMTVAFATSFTVCWTPYYVLGIWYWFDPEMVNRV
SDPVNHFFFLFAFLNPCFDPLIYGYFSL

#gi|60302672|ref|NP_001012627.1|_gonadotropin-
releasing_hormone_II_receptor_Gallus_gallus
MARLGGGTGQDAAAAGGGWLDPGPTVGNVSTEPSSSTSHPKRGCAWSPRLESAEEPLLLP
TFSPAAQARVAATFVLFVLSAGCNVAVLRAAGGRRGGGRSHIRVLLRHLAAADLLVTVVV
MPLDAIWNITLQWRAGDLACRLLMYLRLLAMYASAFVTVVISLDRQAAILRPLAIARARC
RNRAMLRAAWMLSAALAVPQLFLFHTVTLHAPHNFTQCTTHGSFPQPWHETLYNMLSFSC
LFLLPLLIMVCCYTRILLEISRRMGSSLFSSRDVPLRCSGSNIPRARLRTLKMSLVIVSS
FILCWTPYYLLGLWYWFCPRAMQQKVPPSLSHILFIFGLFNACLDPITYGLFTIPFRRRC
GCPCGHSSEPEPPSPATGSFHCSASSLRGRQGMGGTEGPHPPIELGLPTGAGSCQSSAL

#gi|60729565|pir||JC7896_corazonin_receptor_(CG10698)_-
_fruit_fly_(Drosophila_melanogaster)
MEDEWGSFDRLPSVPSASMDLETENEVVSNWSTLANFTRLVAGAAPEIINYTLNMIDVGV
GMATDISNLSVSTTPLPAYAISNSSSLAHTNSRHEAPPMAEQVPEHVMDHAPQLSRSGLL
KVYVLAVMALFSLLGNLLTIWNIYKTRISRRNSRHTWSAIYSLMFHLSIADVLVTWFCII
GEAAWCYTVQWLANELTCKLVKLFQMFSLYLSTYVLVLIGVDRWIAVKYPMKSLNMAKRC
HRLLGGTYILSLVLSLPQFFIFHVARGPFVEEFYQCVTHGFYTADWQEQMYATFTLVFTF
LLPLCILFGTYMSTFRTISSSEKMFQGSKLANYSTAKLPTQTNRQRLIHKAKMKSLRISV
VIIIAFLICWTPYYVMMIMFMFLNPDKRIGDDLQDAIFFFGMSNSLVNPLIYGAFHLCPG
KGGKSSGGGGNNNAYSLNRGDSQRTPSMLTAVTQVDGTGGSSRQMRAFRQQSYYRSSSNG

TAGPGAAPFKEQVGLLHVGPGNGTPGGSVSSGETPQLIRKGSALLARQPSCLREQEHQQR
LLLHEKPSTLVLSYDSQRGGVGVGVASGLLDNNERVSSV

#gi|607301985|gb|EZA44675.1|_gonadotropin-releasing_hormone_receptor-like_protein-1_Microplitis_demolitor
MSDLRVPGRLIEPLKSCVGVTLVTSNSNHTLIEDTLTSNPCNIENYQLIYGLSRRKMSNS
NETKSVSELMSELPIDMQFNEGHLLSIVTYSILMVISIAGNTTVLVLIIERRKTNQSRIN
TLLLHLAIADLLVTLLMMPLEIGWAATVSWRAGDLMCRIMAFFRVFGIYLSSFVLVCISI
DRYYAVLKPMQLINIDRREKLMLIGAWVGAVLCSAPQTLVFHVQRHPDFPWYEQCITYHF
LFSNVTQEVGYSVFSMIMMYCFPLVVIIYTYTSILIEIHSKTRENSKDKIRRSSIGFLGR
ARIRTLKMTIIIVAVFFICWTPYYIMSIWYWIDRQSASKLDQKIQKGLFLFACTNSCMNP
IVYGAFNIRRNTHRTVRASTLETRITPFTLSVKLIDTK

#gi|607301988|gb|EZA44678.1|_gonadotropin-releasing_hormone_receptor-like_protein-2_Microplitis_demolitor
MGLVIASPTAVINSQNYLIIILPLCNNNNNNNNNNNNNNNNNNNNNNNNDRQSININETNK
IWMLNMSRDDDDSMVNLTSLDHAPSLTSSAFIKAIVLGVMAAFSLIANVITIWSISKNRR
KRHQNYSAIYTLILHLSVADLLVTIFCIAGEALWSYTVAWIWGNVTCKLFKFLQMFSLYL
STFVLVLIGLDRFLAVKYPMKSLNKTNRCNRLVLITWIISFILSVPQLIIFHVAQGPFIE
DFSQCVTHGFYTQVWQEQLYTTLSLIFMFILPLIILVATYVSTVITISRSEKSFKFDVTN
NKIKNQITGDINRRRLMHRAKSKSLRISIVIVTAFVIWWTPYYIMMIIFMFLDPDEHLSE
DLQSGIFFFGMSNSLVNPLIYGAFHLWPQKKSHLKFRQKEGSMLQLRSTTTHTSFTLATR
RCSTRLIRNPQGYLTSTKTKRNDKFNEISHPIDETMLDTLINEPANNNEISTQYPSSKLI
IQYNNNHSDAKDTRNNFIQDN

#gi|607356128|gb|EZA50674.1|_Gonadotropin-releasing_hormone_II_receptor_Cerapachys_biroi
MENALLLPEVEDESVMHFLENTKCNNTTYAELPDEMRFNDGHIVTIVTYGILMMISAAGN
ITVLITTIIRRRKSKSRIHTLVMHLSIADLFVTFLMMPLEIGWAITVSWEAGDAMCRIMA
FFRIFGLYLSSFVIICISVDRYFAVMQPLQILDVHRRGKIMLILAWIGSVLCSLPQMLVF
HLETHPNHTCYTQCITFNTFPTYMHELSYSFFGMVTMYWFPLTVICYTYTSIFVEICRRS
KEKNEDKIRRSSIDFLTRARVRTLKMTVTIITVFFICWSPYYVMSLWYWFDRSSANKVDL
RIQRALFFFACTNSSMNPIIYGIFNIRKQRNKAPIRAPTIETRVTPLSLSIKLLD

#gi|607357263|gb|EZA51728.1|_Gonadotropin-releasing_hormone_receptor_Cerapachys_biroi
MPRTTKPTMIASSISSLMPNATVVLPVPCDNLTDFLIQSHDVLVDRSVAYLPSKNITCLE
HAPKLTKAVYLKVIILGVMSVLSLVCNSATIYSITKNRRKQRGCSAIYTLILHLSIADLL
VTVFCMAGDAIWSYNVAWLWGNAACKIFKFLQMFSLYLSTFVLVLIGVDRFVAVRYPMKG
LNASQKCSRFLLLAWVLSFILATPQVAIFHVAQGPFIEVFTQCVTHGFYTEAWQEQLYAS
FSLFFMFILPLVILITTYVSTVITISRSQTMFRTEPARNTTYARNGDINRRRLMHRAKTK
SLRISVVIVMAFLIWWTPYYTMMIILLFLNPDEHLSEELQSGIFFFGMSNSLVNPLIYGA
FHLWPQKQRQGSYQRSDFVMHQRASKQRETQTPLFSH

#gi|617389807|ref|XP_007549120.1|_PREDICTED:_gonadotropin-releasing_hormone_II_receptor-like_Poecilia_formosa
MLPAPPLVPVLNPSFYPSRCRQRSKCFLLPKVQEMNSIPCESAVTMRDPVADLHVNVTSN
CSLAASNLTVAEDVPQLPTFTTAAKIRVIITFILCGMSAFCNLAVLWAAHRDGKRKSHVR
VLIINLTVADLLVTFVVMPVDAAWNITVEWLAGDFACRLLMFLKLLAMYSCAFVTVVISL
DRQAAILNPLAINKARMRNKIMLAVAWGMSVVFSIPQIFLFHNVTIVHPEKFTQCTTWGS
FATRWHETAYNMFTFSCLFLLPLVIMITCYARIFHEISKRLKKDNLPTSGVHLRCSKNNI
PKARMRTLKMSVVIVSSFIVCWTPYYLLGLWYWFFPDHLEDKVSHSLTHILFIFGLVNAC
LDPLIYGLFTIHFRKGLRRYYPNSASAGDAENTTVLTGSFTSPALSLSLRRELRRLSQEK
LVLCSDSHRKAEPPSLSSSFLAEDRDLQQSSPESIL

#gi|617389923|ref|XP_007549149.1|_PREDICTED:_putative_gonadotropin-releasing_hormone_II_receptor_Poecilia_formosa

MLRGRKMNASSCCTAPIAMYQQSSGSDLNAGCDLSAPRCNWTAVDGTPQLPTFSTAAKVR
VIVTFILCGVSTLCNVAVLWAASGHRRRSHVRVLIVNLTAADLLVTLIVMPVDATWNITV
QWLAGDLACRFLMFLKLQAMYSCAFVTVVISLDRQSAILNPLLISMAPKRNRVMLMVAWI
MSALLAVPQVFIFHNVTITYPANFTQCTTRGNFVTHWQETAYNMFTFSWLFLLPLAIMIT
CYTRIFIHISKQMTKKNVSSDEPHLRCSKNNIPKARMRTLKMSVVIVVSFIVCWTPYYLL
GLWYWFFPDDLEVKVSHSLTHILFIFGLFNTCLDPIIYGLFTVRFNRRLRSCCSTATVAS
GLDAKPATADSVKCTLAGESAEGGRDGSCESNEQKSTDSRL

#gi|617444841|ref|XP_007566469.1|_PREDICTED:_gonadotropin-releasing_hormone_II_receptor-like_Poecilia_formosa
MFHQLTDQTVNGSCQGTTLDCNKSADGNALQLPTFSTAAKVRVIITFTLCAVSAVCNLIV
LWAAGKGGKRKSHVRILIMNLTVADLLVTFIVMPVDAVWNITVQWQAGDAACRLLMFMKL
VAMYSCAFVTVVISLDRQSAILNPLGISEAKRKSKIMLTVAWTMSFILSLPQMFIFHNVT
ITVPQNFTQCTTHGSFIHRWQETLYNMFTFTCLFLLPLVIMIFCYTRILVEISSRMAQNN
TLSRDIHLRRSHSNIPKARMRTLKMSVVIVTSFIICWTPYYLLGLWYWLFPEKMEETVSH
SLTHMLFIFGLFNACLDPITYGLFTIHFRQGLRRRRQSSTAHTELENNTCLVQVSGLSSR
RQITSGGSGKQTGEESDSSNLKNASCPVISVSKV

#gi|617456508|ref|XP_007570475.1|_PREDICTED:_oxytocin_receptor_Poecilia_formosa
METISNESDIWQLNESWRNSSLLNGTGGLNQTNPLKRNEEVAKVEVTVLALVLFLALAGN
LCVLLAIHTTKHSQSRMYYFMKHLSIADLVVAIFQVLPQLIWDITFRFYGPDILCRLVKY
LQVVGMFASTYMLVLMSVDRCLAICQPLRSLHRRKDRFYVIFSWLLSLLFSTPQMFIFSL
REVGSGVYDCWGDFVKPWGAKAYITWISLTIYIIPVAILSICYGLISFKIWQNFKLKTRR
EQCISMTPKTCKSNTLARVSSVRLISKAKITTVKMTFVIVVAYIVCWTPFFSVQMWSAWD
PAAPREAMPFIISMLLASLNSCCNPWIYMCFAGHLFHDLRQNFLCCSARYLKSSQCRCEH
DFDSSHKSNSSTFAIKSTSSQRSITQTSTT

#gi|617599806|ref|XP_007522348.1|_PREDICTED:_oxytocin_receptor_Erinaceus_europaeus
MEGALAANWSIEASGNASGEHPGVPGNGTAGPPQRNEALARVEVAVLCLILVLALGGNAC
VLLALRTTRRQPSRLFFFMKHLSIADLVVALFQVLPQLLWDITFRFYGPDLLCRLVKYLQ
VVGMFASTYLLLLMSLDRCLAICQPLRALRRRADRLAVLGTWLGCLVVSVPQVHIFSLRE
VADGVFDCWAVFIQPWGPRAYVTWITLAVYIVPVIVLAACYGLISFKIWQNLRLKAAAAA
GAAGGQAGEPAALARVSSVKLISKAKIRTVKMTFIIVLAFVVCWTPFFFVQMWSVWDKDA
PKEASAFIIAMLLASLNSCCNPWIYMLFSGHLFHELAQRFLCCWASRLQAARPGDTSVSK
KSNSSTFVLSRHSSSQRNGPQPTSAAQ

#gi|617665003|ref|XP_007538756.1|_PREDICTED:_gonadotropin-releasing_hormone_II_receptor-like_Erinaceus_europaeus
MSVGNGTPWGSAVGEEVWAGPGVQVEGSELPTFSAAAKVRVGVTIVLFVSSAGGNLAVLW
SVTRQQSSQFRRSPVRRLFAHLAAADLLVTFVVMPLDATWNVTVQWLAGDIACRILMFLK
LVAMYAAAFLPVVIGLDRQAAILHPLGPRSTGSKLLGAAWGLSFLLALPQLFLFRTVRRA
GPVPFTQCVTKGSFKARWQETTYNFFTFCCLFLLPLTAMTICYSRIVLSVSSPRTRKGNH
AHADEFALRRSPDNRPRVRLRALRLALLVLLTFVLCWTPYYLLGLWYWLSPTMLAEVPSS
LSHILFLFGLLNAPLDPLLYGVFTLGCRRRPHELSIDSSREGGSGKMTQQGTQVPRQLEL
QANVATWAGQTRDILLTSIHSQQNMQEA

#gi|620949091|ref|XP_007660695.1|_PREDICTED:_oxytocin_receptor-like_Ornithorhynchus_anatinus
MGEPLDVWATNGSAFPGNGSLDPGPVGGNSSADPLKRNEAMAKVEVTVLCLILFLALTGN
LCVLLALHTTRHKHSRMFFFMKHLSIADLVVAVFQVLPQLLWDITFRFYGPDFLCRLVKY
LQVVGMFASTYLLLLMSLDRCLAICQPLRSLRRRADRASVLGTWLLCLVVSVPQIHIFSL
RDVGNGVYDCWAVFIQPWGPKAYITWITLAVYIIPVLVLSGCYGLISFKIWQNLRLKTAQ
EPPGGLASPDGADAGSRAVLARVSSVKLISKAKIRTVKMTFIIVLAFIVCWTPFFFVQMW
SVWDKDAPKEGQAVSQGGYSLNAMFDPQTAYTESGAMTIGY

#gi|63054892|gb|AAY28982.1|_gonadotropin-releasing_hormone_receptor_Paralichthys_olivaceus
MHHLPRDRQLNSSWNGSSPHSNWTAGGDTLQLPTFTTAAKVRVTITFILCATSAFCNLAV
LWAAHSDGKRKSHVRVLIVNLTVADLLVTFIVMPVDAVWNITVQWLAGDLACRLLMFLKL
QAMYSCAFVTVVISLDRQSAILNPLAINEARKRNRVMLSVAWTMSVVLSVPQIFLFHNVT
IVHPEDFTQCTTRENFVSHWHETAYNMFTFSCLFLLPLAIMIICYTRIFCEISKRLKSHN
LSSNEVHLRCSKNNIPRARMRTLKMSIVIVLSFIICWTPYYLLGLWYWFFPDDLEGKVSH
SLTHILFIFGLVNACLDPVIYGLFTIHFRKGLRRYFCRAPPAPDLDNNTVLTGSFTCTAN
ILPLKREVSPASQEKSTMYGNNHGKEESTSQGGSFLTADIYTARDRNQSSSDSTM

#gi|632949594|ref|XP_007890243.1|_PREDICTED:_gonadotropin-releasing_hormone_II_receptor-like_Callorhinchus_milii
MKHTLLFSRDFPLYNSSGCNGSSERDGAFSTKDSSVPGGENELLLPTFSAAAKVRVVITL
AIFLMSAYLNLTVLWSALRSKGGGSRSHLRMLILNLSCSDLLVTFIVMPLDAAWNTTVQW
YAGDLACKLLMFLKLLSMYSGAFITAVISLDRYCAILHPLAITRAKERNRTMLLVAWTLS
VILATPQLFLFHTVSISVPAPFTQCVTHGSFTEHWQQKVYFLFTFMWLFLLPLIIMVFCY
TSILICITRKMNAEIISKDMGLRCTRNYIPKVRMKTLKMTIVLVSTFIICWTPYYVLGFW
YNFFPAMINKKQLPESINHILFIFGTLNSFLDPVISRIGYLRLNSG

#gi|634825428|ref|XP_007946406.1|_PREDICTED:_gonadotropin-releasing_hormone_II_receptor_Orycteropus_afer_afer
MSAGNGTPWGSAAGEEVWAGSGVEVEGSELPTFSAAAKVRVGVTVVLFVSSAGGNLAVLW
SVTRPQPSQVRSSPVRRLFAHLAAADLLVTFVVMPLDATWNITVQWLAGDIACRTLMFLK
LMAMYAAAFLPVVIGLDRQAAVLHPFGPRSGGRKLLGAAWGLSFLLALPQLFLFHTVRRA
GPVPFIQCATKGSFKARWQETTYNLFTFCCLFLLPLTAMAICYSRIVLSVSSPRTKKGNH
ASADEFALRRSLDNRPRVRLRALRLALLVLLTFVLCWTPYYLLGLWYWFSPAMLTEVPPS
LSHILFLFGLLNAPLDPLLYGAFTLGCRRRHQEQGTDSSREGGSGRMPLRQLEAHTNVAA
RREGETEETFL

#gi|638262172|ref|NP_001279167.1|_arginine_vasopressin_receptor_1A_Callorhinchus_milii
MGVTETSGWDWGIWNFTRAGVSLSRLQNESFTKSRNKTDPFGRNENLAKTEISVLGIIFL
VAVIGNLSVLMALYKTKKKMSRMHLFIKHLSVADLVVAVFQVLPQFIWDITYRFNGPDFL
CRIVKHLQVLGMFASTYMMVMMSVDRYIAICHPLKTLQQATKRSYLMIITTWMGSFILSA
PQSFIFSLSEIETGSGVYDCWANFILPWGIKAYITWITVSVFIIPVLTLAACYICVCYNI
SKNVKYKTTNNSSESAVKNGLITSGVNVSNVKTISKAKIRTVKMTLVIVLAYIVCWAPFF
SVQMWSVWDQKAPKDDSTDTAFTLTMLLASLNSCCNPWIYMFFSGHLLSDVSKFFPCCHK
FTQSLKKEDSDSITKRHTLLTRLSHRSSTLSSCNWKDTETSPQLLRFIPIET

#gi|638272251|ref|NP_001279833.1|_gonadotropin-releasing_hormone_receptor_Callorhinchus_milii
MCLKPLPNNTMIGSVESGKNNFTLHNDSLDIAVSNSSLKFPTLSISGIIRVAITFTLFIL
SIAMNGIFLLKLSRQHKKKASRLKLLLDNLMVANLVETIIVMPMDGIWNIMVQWYGGQFL
CKVLNFLKLFSMYSPAFMVVVISIDRCLAVTKPLKSATQSTQIRKYMIYTAWLFSFVLAL
PQLWLFRMIHYSEPYAFSQCNTLRSFYNQWDQTIYNFFTFGFLFVIPLFIMLFCNFKIIF
KMMKILRHNVNEISLNRSKNIIPQARMKTLKMTIAFVTSFIICWTPYYLIGIWYWIDPDL
HNRLPEPMNHFFFVFGLLNPCFDPLIYGYFSL

#gi|640814950|ref|XP_008064224.1|_PREDICTED:_gonadotropin-releasing_hormone_II_receptor_Tarsius_syrichta
MSAGNSTPWKSAAGEDVWAGSGVEVEGSDLPTFSAAAKVRVGVTIVLFVSSAGGNLAVLW
SVTRPQPSQLRSSPVRRLFAHLAVADLLVTFVVMPLDATWNITVQWLAGDIACRTLMFLK
LMAMYAAAFLPVVIGLDRQAAVLHPLGPRSGGRKLLGAAWGLSFLLALPQLFLFHTVRRA
GPVPFTQCVTKGSFKAQWQETTYNLFTFCCLFLLPLTAMAICYSRIVLSVSSSRTRKGSH
APAGEYTLRRSLDNRPRVRLRALRLALLVLLTFVLCWTPYYLLGLWYWFSPAMLTEVPSS
LSHILFLFGLLNAPLDPLLYGAFTLGCRRGHQELSIDSSREGGSGRMPHQEIQAWRQPEA
QTNVTSRRATETKETFL

#gi|641733110|ref|XP_008156954.1|_PREDICTED:_gonadotropin-releasing_hormone_receptor_Eptesicus_fuscus
MANGASAEQSQNHCSSINSSSPLMQGSLPTLTLSGKIRVTVTFFLFLLSTTFNATFLLKL
KQWTQKKEKGKKLSRMNVLLKHLTLANLLETLIVMPLDGMWNITVQWYGGELLCKVLSYL
KLFSMYAPAFMMVVISLDRSLAITRPLPVSNSKLGQSLIGMAWLLSSLFAGPQLYIFRMI
HLADGSGQSDGFSQCVTHCSFPQWWHQAFYNFFTFSCLFIIPLLIMLICNAKIIFTLTRV
LHQDPHKLQLNQSKNNIPRARLRTLKMTVAFATSFTVCWTPYYVLGIWYWFDPEMLNRVS
DPVNHFFFLFAFLNPCFDPLIYGYFSL

#gi|641774155|ref|XP_008170675.1|_PREDICTED:_gonadotropin-releasing_hormone_II_receptor-like_isoform_X1_Chrysemys_picta_bellii
MQLNGTICVGMPEERDLMTPPQSADVVDENLSVSGCPEPWIEPTFTLAARVRVIVTICFF
LIAASSNSVVLYSIMRKRRKSHVRLLILSLTVADLLVTFTVMPLDAVWNVTVQWYAGDLP
CKLLNFLKLFAMYSAALVLVVISLDRHSAILHPFAFANSSRRNRLMLCVAWVMSLLLASP
QLFLFHLHTVPGVNFTQCVTHGSFQEHWEEIIYNMFTFTTLYVAPLSVMIVCYIRIIWEI
SKQLKINKGLARSKNDHISKARMKTLKMTVVIVASFIICWTPYYLLGLWYWFQPDMIQSM
PEYINHSLFLFGLLHTCSDPIIYGLYTPSFREDMKACLRGIETVITGQERNKLMSSSEMN
IKDYIINGGAASGASNGTIIHTVC

#gi|641774157|ref|XP_008170676.1|_PREDICTED:_gonadotropin-releasing_hormone_II_receptor-like_isoform_X2_Chrysemys_picta_bellii
MPEERDLMTPPQSADVVDENLSVSGCPEPWIEPTFTLAARVRVIVTICFFLIAASSNSVV
LYSIMRKRRKSHVRLLILSLTVADLLVTFTVMPLDAVWNVTVQWYAGDLPCKLLNFLKLF
AMYSAALVLVVISLDRHSAILHPFAFANSSRRNRLMLCVAWVMSLLLASPQLFLFHLHTV
PGVNFTQCVTHGSFQEHWEEIIYNMFTFTTLYVAPLSVMIVCYIRIIWEISKQLKINKGL
ARSKNDHISKARMKTLKMTVVIVASFIICWTPYYLLGLWYWFQPDMIQSMPEYINHSLFL
FGLLHTCSDPIIYGLYTPSFREDMKACLRGIETVITGQERNKLMSSSEMNIKDYIINGGA
ASGASNGTIIHTVC

#gi|646697814|gb|KDR09300.1|_Gonadotropin-releasing_hormone_II_receptor_Zootermopsis_nevadensis
MTMPTAESTAMTDGLTDDMKFNDGHRMSIFTYSILMAVSAVANVTVLVNILRRRRALRFR
NNYMFMHLAIADLLVTFLMMPLEIGWNATVSWKAGDAVCRIMSFFRTFGLYLSSFVIVSI
SLDRCFAILRPMSNVVNVAHRGNMMLTVAWSLATLCSIPQVVIFHVERHPNVTWYEQCVA
FNMFPTKLHELTYRVLGMVMMYGLPLAVIIISYACIIAEIFRRYQLSPDDSFRRSSLVFL
NRARNRTLKMAIIIFVVFFICWTPYYVMCLWYWIDEASAETVDQRVQKGLFLFACTNSCM
NPIVYGYFNFRSGRGSGYAAPGGRTGQQLQHQNIVALSASTTGANSRRGSNSSSICRNNS
NQNMWKNSHRPAHMPRINSKETDHPATQTTSSNMTDKPKTQQTTNNKEEEHEQPRPTSET
NNCSSRTSPTVERANDTTLTTVVCR

#gi|649572219|ref|NP_001280549.1|_ACP_receptor_Tribolium_castaneum
MQAVGKMGEEHYDEDSKSNFSVLNETLDGFANETVSPDVLFQQNLTVILVYSALFVVAAV
GNLTVFISLFRSRHRKSRISLMIRHLAIADLIVTFIMIPIEVGWRLTGKWIAGNVACKVF
LFLRAFGPYLSSNVLVCVSLDRYFAVLHPLRVNDARRRGKIMLAFAWGTSFVYCIPQSFV
FRVRAHPKYPNYEQCVSFGFFENTAQEIAYNLMCVMCMYFIPLFVIIVAYTAIMCEISKN
SKETKGESYRTSNGRMRLRRSDISNIERARSRTLRMTITIVAVYVWCCTPYVIITMWYMF
DRASATSLPEWLQDTFFMMVVSNSCMNPIVYGSYVINFQRVNCNCFCFRKTASESHLNVG
SGATRSTAMVHGAGGNGYTRSPTPKSNLNLTGLLSKSRLPDKPPSVGHISFLSEPRTARN
YRSSFHSEPCSRTRMCPDELCLDTSCHSADYYSSAVL

#gi|657525208|ref|XP_008279761.1|_PREDICTED:_gonadotropin-releasing_hormone_II_receptor-like_Stegastes_partitus
MAGNLSLWVSTPVQNVSVSPASPSPPLWEAPSFTVAAKCRVAATLVLFVFAAVSNLSVLI
SVCWGRGYRLAKHLRPLIASLASADLLMTFVVMPLDAIWNITVQWYAGDVMCKLMCFLKL
FAMHSAAFILVVVSLDRYRAILHPLDSLDAGLRNRRMLLVAWTLSLLLASPQLFIFRAVK
ADGVDFTQCVTHGSFQHHWQETAYNMFHFVTLYVFPLLVMTFCYTRILTKINGQMHRSKE

CEQHLRRSGTDVIPKARMKTLKMTIVIVSSFVICWTPYYLLGIWYWFQPAIIQHTPEYVH
HILFVFGNLNTCCDPVIYGFYTLSFRADLADVVARCCSRRSNDESPRSVDRLDGHSAGAA
GEMESELSSNPHCGDPHVPVVI

#gi|657588081|ref|XP_008298364.1|_PREDICTED:_putative_gonadotropin-releasing_hormone_II_receptor_Stegastes_partitus
MNSSLCDSAVTMYHLATDHQVNASCNCTSSNWTAEDEAPQLPTFTTAAKVRVIITFILCS
ISAFCNLAVLLAAHSDGKRKSHVRVLIINLTVADLLVTFIVMPVDAVWNITVQWLAGDFA
CRLLMFLKLQAMYSCAFVTVVISLDRQSAILNPLAINKARKRNRVMLTVAWGMSVVLSVP
QMFLFHNVTIIHPEDFTQCTTRGSFVTRWHETAYNMFTFSCLFLLPLVIMITCYTRIFCE
ISKRLKKDNLPSNELHLRCSKNNIPRARMRTLKMSIVIVLSFIICWTPYYLLGLWYWFFP
DDLEGKVSHSLTHILFIFGLINACLDPVIYGLFTIHFRKGLRRYYCKAATATDLDNDTVI
TGSFACAANSLSLKRGVSPATPERLTLCSDNNSKTESTSERSSFLTADTDAERVQSNPES
IL

#gi|657598554|ref|XP_008304061.1|_PREDICTED:_gonadotropin-releasing_hormone_II_receptor-like_Stegastes_partitus
MMFHQLTDQTVNDSCQQELTSVCNKSADGDALQLPTFSTAAKVRVIITFTLCAVSAVCNL
VVLWAACNGGKRKSHVRILIMNLTVADLLVTFIVMPVDAVWNITVQWQAGDVACRLLMFM
KLVAMYSCAFVTVVISLDRQSAILNPLGISEAKRKSKIMLTVAWTMSVILSLPQMFIFHN
VTITVPENFTQCTTHGSFVQRWQETLYNMFTFVCLFLLPLAIMIFCYTRILIEISSRMAR
SNQFSKDVHLRRSHNNIPKARMRTLKMSIVIVTSFIICWTPYYLLGLWYWLFPEKMEETV
SHSLTHMLFIFGLFNACLDPITYGLFTIHLHQGLKRCRQPSNTRTELENNTCLIRMSSHT
QIGHNAHAEGLNDNNGTKNATQSVVPVSKI

#gi|657747846|ref|XP_008309235.1|_PREDICTED:_gonadotropin-releasing_hormone_II_receptor-like_Cynoglossus_semilaevis
MMFHQLTEPSVNGTCTGATVCNGSADGDTLQLPTFSTAAKVRVIITFSLCAVSAVCNLLV
LWAASNGGKRKSHVRILIMNLTVADLLVTFIVMPVDAVWNITVQWQAGDAACRLLMFMKL
VAMYSCAFMTVVISLDRQSAILNPLGISEAKRKSKIMLTVAWTMSVILALPQMFLFHNVT
ITVPANFTQCTTYGSFMQHWQETVYNMFTFVCLFLLPLIIMIFCYTRILVEISSRMARGN
LVSRDVHLRRSHNNIPKARMRTLKMSIVIVTSFIICWTPYYLLGLWYWLYPEDMEETVSH
SLTHMLFIFGLFNACLDPIIYGLFTIHLNHRVRRCCRRSNTPTELENNTCLLHMTHLSTH
RHNASESHSAEAGEGGNKLSNIRPSSRTIIPISKIA

#gi|657805279|ref|XP_008329777.1|_PREDICTED:_gonadotropin-releasing_hormone_II_receptor-like_Cynoglossus_semilaevis
MDRTLSPWNSTHSAPLQNNSTFYLLNASPSPSPPPWKAPSFTVAAGCRVAATLVLFIFAA
VSNLSVLVSICWGRGYRLAAHLRPLIASLASADLMMTFVVMPLDAIWNITVQWYAGDVMC
KLMCFLKLFAMHSAAFILMVVSLDRYWAILHPLDSLDAGLRNRRMLAVAWTLSVLVASPQ
LFIFRTIKADGVDFTQCVTHGSFQHRWQETAYNMFHFLTLYVFPLLVVTFCYTRIFTKIN
AQMLKRKDGEHCLRRSGTDMIPKARMKTLKMTIVIVSSFVICWTPYYLLGIWYWFQPSIV
QYTPEYVHHILFFFGNLNTCCNPVIYGFYTSSFRADLADVVAFCRGRRGQNASPHSVDGL
SARRAGAPGEMESDLSSNQHSGNPC

#gi|658841242|ref|XP_008403020.1|_PREDICTED:_gonadotropin-releasing_hormone_II_receptor-like_Poecilia_reticulata
MNSIPCESTVTMRDSVVDLHVNVTSNCSLAASNLTAAEDVPQLPTFTTAAKIRVIITFIL
CGTSAFCNLAVLWAAHRDGKRKSHVRVLIINLTVADLLVTFVVMPVDAAWNITVQWLAGD
FACRLLMFLKLLAMYSCAFVTVVISLDRQAAILNPLAINKARMRNKIMLAVAWGMSVVLS
IPQIFLFHNVTIVHPEKFTQCTTWGSFATRWHETAYNMFTFFCLFLLPLVIMITCYARIF
HEISKRLKKDNLPTSGVHLRCSKNNIPKARMRTLKMSVVIVSSFIVCWTPYYLLGLWYWF
FPDHLEDKVSHSLTHILFIFGLVNACLDPLIYGLFTIHFRKGLRRYYPNAASAGDAENTT
VLTGSFMSPALSLSLRRELRRLSQEKMVLCGDSHRKAEPPSLSSSCLAEDRDLQQSSPES
IL

#gi|658841340|ref|XP_008403070.1|_PREDICTED:_putative_gonadotropin-releasing_hormone_II_receptor_isoform_X1_Poecilia_reticulata
MNMTVSCLLSFPSSSVCATDQNADSMLRGRKMNASSCCAAPVAMYQQSSGSDLNASCDLS
APRCNWTAVDGTPQLPTFSTAAKVRVIVTFILCGVSTLCNVAVLWAASGHRRRSHVRVLI
VNLTAADLLVTLIVMPVDAAWNITVQWLAGDLACRFLMFLKLQAMYSCAFVTVVISLDRQ
SAILDPLLISMAPKRNRVMLTVAWIMSALLAVPQVFIFHNVTITYPANFTQCTTRGSFVT
HWQETAYNMFTFSWLFLLPLAIMVVCYTRIFIHISKQITKKNVSSDEPHLRCSKNNIPRA
RMRTLKMSVVIVVSFIVCWTPYYLLGLWYWFFPDDLEVKVSHSLTHILFIFGLFNTCLDP
IIYGLFTVRFNRRLSSRAAAASGVDAKPATADSVKCTLAGGSAEGRRDGSCESNEQNSPT
AGSE

#gi|658841342|ref|XP_008403071.1|_PREDICTED:_putative_gonadotropin-releasing_hormone_II_receptor_isoform_X2_Poecilia_reticulata
MLRGRKMNASSCCAAPVAMYQQSSGSDLNASCDLSAPRCNWTAVDGTPQLPTFSTAAKVR
VIVTFILCGVSTLCNVAVLWAASGHRRRSHVRVLIVNLTAADLLVTLIVMPVDAAWNITV
QWLAGDLACRFLMFLKLQAMYSCAFVTVVISLDRQSAILDPLLISMAPKRNRVMLTVAWI
MSALLAVPQVFIFHNVTITYPANFTQCTTRGSFVTHWQETAYNMFTFSWLFLLPLAIMVV
CYTRIFIHISKQITKKNVSSDEPHLRCSKNNIPRARMRTLKMSVVIVVSFIVCWTPYYLL
GLWYWFFPDDLEVKVSHSLTHILFIFGLFNTCLDPIIYGLFTVRFNRRLSSRAAAASGVD
AKPATADSVKCTLAGGSAEGRRDGSCESNEQNSPTAGSE

#gi|658855851|ref|XP_008410575.1|_PREDICTED:_gonadotropin-releasing_hormone_II_receptor-like_Poecilia_reticulata
MFHQLKDQAVNGSCQGTTLDCNKSADGNALQLPTFSTAAKVRVIITFTLCAVSAVCNLIV
LWAAGKGGKRKSHVRILIMNLTVADLLVTFIVMPVDAAWNITVQWQAGDAACRLLMFMKL
VAMYSCAFVTVVISLDRQSAILNPLGISEAKRKSKIMLTVAWTMSFILSLPQVFIFHNVT
ITVPENFTQCTTHGSFIHRWQETLYNMFTFTCLFLLPLVIMIFCYTRILVEISSRMAQNN
TLSRDIHLRRSHSNIPKARMRTLKMSIVIVTSFIICWTPYYLLGLWYWLFPEKMEETVSH
SLTHMLFIFGLFNACLDPITYGLFTIHFRQGLRRRRQSSATHTELENNTCLVQVSGLSSR
RQITSGGSGKQTGEESDSSNLKNASCPVISVSEV

#gi|659134986|gb|AID62089.1|_gonadotropin-releasing_hormone_receptor_Gallus_gallus
MARLGGGTGQDAAAAGGGWLDPGPTVGNVSTEPSSSTSHPKRGCAWSPRLESAEEPLLLP
TFSPAAQARVAATFVLFVLSAGCNVAVLRAAGGRRGGGCSHIRVLLRHLAAADLLVTVVV
MPLDAIWNITLQWRAGDLACRLLMYLRLLAMYASAFVTVVISLDRQAAILRPLAIARARC
RNRAMLRAAWMLSAALAVPQLFLFHTVTLHAPHNFTQCTTHGSFPQPWHETLYNMLSFSC
LFLLPLLIMVCCYTRILLEISRRMGSSLFSSRDVPLRCSGSNIPRARLRTLKMSLVIVSS
FILCWTPYYLLGLWYWFCPRAMQQKVPPSLSHILFIFGLFNACLDPITYGLFTIPFRRRC
GCPCGHSPEPEPPSPATGSFHCSASSLRGRQGMGGTEGPHPPIELGLPTGAGSCQSSAL

#gi|668454279|gb|KFB42760.1|_G-protein_coupled_receptor_Anopheles_sinensis
MLELLPGNGTSALENYTASLADLFATEQPVALLGNASLAIGAVYAASSQHLQQHPNTSSE
VALATASGLHPAIHQLSSGLATLTTDLLGQSGSLVLAREECDQLNISYAFENGSALAVPG
LSCYEHAPTLSRSGVIRVIVLSAMAIVSLLGNVATMWNIQKNRKSRRVTRHNWSAIYSLI
FHLSIADVLVTGFCIIGEAAWYYTVDWVAGNLFCKLFKLCQMFSLYLSTYVLVLVGVDRW
VAVKYPMKSLNTARRCHRFLFVAYLLSFVLSLPQVCKEGC

#gi|6689212|emb|CAB65407.1|_gonadotropin-releasing_hormone_receptor_Seriola_dumerili
MDTTLCDAAVTMYHLTTDHQLNASCNCSSPACNWTAGGDAPQLPTFTTAAKVRVIITFIL
CAISAFCNLAVLWAAHSDGKRKSHVRVLIINLTVADLLVTFIVMPVDAVWNITVQWLAGD
FACRLLMFLKLQAMYSCAFVTVVISLDRQSAILNPLAINKARKRNRVMLSVAWGMSTVLS
VPQIFLFRNVTINHPEDFTQCTTRGSFVTHWHETAYNMFTFSCLFLLPLVIMITCYTRIF
CEISKRLKKDNLPSNEVHLRCSKNNIPRARMRTLKMSIVIVLSFIICWTPYYLLGLWYWF
FPDDLEGKVSHSLTHILFIFGLVNACLDPVIYGLFTIHFRKGLRRYYCNAATASDLDTNT
VITGSFTCATNSLPLKRQVSPSSQERFMLCSDNHSKAESASPGSSFLTADNDAERDLNQS

SPESVI

#gi|669263119|ref|XP_008636455.1|_PREDICTED:_putative_gonadotropin-releasing_hormone_II_receptor_Corvus_brachyrhynchos
MARLGKAGQDTLDAAAILHPLAIARARARNRAMLHVAWILSAGLAVPQLFLFHTITLRPP
HNFTQCTTRGSFPRPWHETLYNMVGFACLFLLPLLIMVCCYTRILLEISRRMGSSLFSSQ
DASLRCSRNNIPRARLRMLRMSLVIVSSFILCWTPYYLLGLWHWFCPRAMEKRVSPALTH
ILFIFGLFNACLDPITYGLFTIPLRGGWGCPCRHGPPA????SGSFHCSASSLPPKRGAQ
GVQRSRGAARDASCHSSSL

#gi|669263183|ref|XP_008636805.1|_PREDICTED:_gonadotropin-releasing_hormone_II_receptor-like_Corvus_brachyrhynchos
MSEEQLPASPHCPSVWEDTNVSASGYPQYWVEPQFTQAAKVRVIITAVFFLLAVGSNMAV
LGSLLRKRRKSHVQPLILSLALADLLVTVMVMPLDAAWNVTVQWYGGDISCKILNFLKLF
AMYAAALVLVVISLDRHAAILHPFSRAHHRNGMLLRAAWAGSVLLALPQLFLFHLNTAPG
GNFTQCVTHGSFRAHWEETVYNMFTFTTLYITPLSVMIVCYVRILWEISKQLKVNKGLTR
NQNDHISKARMKTLKMTIVIVATFIICWTPYYLLGLWYWFQPAMIQKMPEYVNHSFFLFG
LLHTCTDPIIYGLYTPSFREDVQLCLRGIETAITRQKRHKPISASEKNIKDSAANGGVAS
GGSNGTTVSMV

#gi|671030373|ref|XP_008705977.1|_PREDICTED:_gonadotropin-releasing_hormone_II_receptor_Ursus_maritimus
MSAGNGTPRGLAAGEEVWAGSGVEVEGSELPTFSAAAKVRVGVTIALFISSAGGNLAVLW
SVTRPQPSQLRPSPVRRLFAHLAAADLLVTFVVMPLDAIWNITVQWLAGDIACRTLMFLK
LVAMYAAAFLPVVIGLDRQAAILHPLGPRSAGRKLLGTAWGLSFLLALPQLFLFHTVRRA
GPVPFTQCVTKGSFKARWQEITYNLFTFCCLFLLPLTAMTICYSRIVLSVSSPRTRNGNH
APAGEFALRRSLDNRSRVRLRALRLALLVLLTFVLCWTPYYLLGLWYWFSPTMLTKVSPS
LSHILFLFGLLNAPLDPLLYGAFTLGCRRRHQELSIDSSREKGSERLPRQEIQALRQLQV
QTNVAARRTGEMKETFL

#gi|675416294|ref|XP_008921392.1|_PREDICTED:_gonadotropin-releasing_hormone_receptor_Manacus_vitellinus
MSEEQLPASAHCPTTQEDTNVSASAYPQYWVEPRFTRAAKVRVIITAIFFLLAAGSNAAV
LGSLLRKRRKSHVQPLILSLALADLLVTVTVMPLDAAWNVTVQWYGGDISCKVLNFLKLF
AMYAAALVLVVISLDRHAAVLHPFSRAHRRNGMLLRAAWAGSVLLALPQLFLFHLHTIPG
GNFTQCVTHGSFRAHWEETVYNMFTFTTLYITPLSVMVVCYIRILWEISRQLKINKGLTR
SQNDHISKARMKTLKMTVVIVATFIICWTPYYLLGLWYWFQPAMIQKMPEYVNHSFFLFG
LLHTCTDPVIYGLYTPSFREDVQLCLRGIETAITRHKRHKPISASEKNIKDGAVNGGVAS
GGSNGTTASAV

#gi|676423937|ref|XP_009044166.1|_hypothetical_protein_LOTGIDRAFT_103088_Lottia_gigantea
MNYFEVIGNDSSINRSGNWGIIIPEPQPPTFDDIAMTKCIVFGTMFVIAFIGNVATLIQM
YRMRHRKSTINTLIVNLAIGDLFIVCFCFPVEGIWAATVQWYGGTAMCKIIKFIQVFSLY
LSTYVTVAIGLDRCVAILDPMRRKSAPTRVRTMIMLAWFFSALFSIPQAIIFNVRRGPFK
EDFYQCVTMGSYPNAWQEEVYSIASFLLMFVIPLVIIGTAYGLIFSTIARKSKEFIKKNP
TVRTTSYNDIQRGPVRSNLLRKAKRKSLRMSIVIVVVFVMCWTPYYVIFICLTFLDWEEI
NPRIYRLVLWLFFLGMSNAVMNPLIYGAFQICKVHTPRSVHLRAFRSHKILLLFYSKFYD
GFMFG

#gi|676439762|ref|XP_009048957.1|_hypothetical_protein_LOTGIDRAFT_140886_Lottia_gigantea
MAENQTDIRCNDSLLNVTPCNTFPSLPRELTFNDDSLVSVITCSCLFIIAACGNLTVFIT
LFRNRNIKSRVNLFIMHLSIADLIVTFVMLPLEAAWHLTVAWKGGDMACRAMMFFRAFGF
YLSSFILVTISLDRYFAIMHPLSLNDADRRGRIMLSLAWICSIVASIPQSVIFHVERHPV
YEWFTQCVTFNFFPSPSHELAYNLFNVIAVYGLPLVIITGTYSLILCEISKKSRQSQMQL
ESSSIRQKGELRRSAMGIIERARIRTLKMTLVIGKIHIVFFLLFLSGSLVD

#gi|678000198|ref|XP_009077232.1|_PREDICTED:_gonadotropin-releasing_hormone_II_receptor-like_Acanthisitta_chloris
MSEEQLPASPHCPTVREDTNVSVSTYPQYWVEPQFTQAAKVRVIITAIFFLLAAGSNAAV
LGSLLRKRRKSHVQPLILSLAVADLLVTVTVMPLDAAWNVTVQWYGGDISCKILNFLKLF
AMYAVALVLVVISLDRQAAILHPFSHTHRRNGMLLRAAWAASVLLALPQLFLFHLHTIPG
GNFTQCVTHGSFRAHWEETVYNMFTFTTLYITPLSVMIVCYIRILWEISRQLKINKGLTR
NQNDHISKARMKTLKMTIVIVATFIICWTPYYLLGLWYWFQPAMIQKMPEYVNHSFFLFG
LLHTCTDPVIYGLYTPSFREDVQLCLRGIETAITRHKRQNPISASEKNIKDGAVNGGVAS
GGSNGTTASTV

#gi|6808613|gb|AAF28464.1|_pituitary_gonadotropin_releasing_hormone_receptor_Morone_saxatilis
MNTTLCDSAVAMYHLTTDHQLNASCNYSSPTSNWTSGGGSLQLPTFTTAAKVRVIITCIL
CGISAFCNLAVLWAAHSDGKRKSHVRVLIINLTVADLLVTFIVMPVDAVWNITVQWLAGD
LACRLLMFLKLQAMYSCAFVTVVISLDRQSAILNPLAINKARKRNRVMLTVAWGMSVVLS
VPQLFLFHNVTIIYPEDFTQCTTRGSFVTHWHETAYNMFTFSCLFLLPLIIMITCYTRIF
CEISKRLKKDNLPSNEVHLRRSKNNIPRARMRTLKMSIVIVSSFIVCWTPYYLLGLWYWF
FPDDLEGKVSHSLTHILFIFGLVNACLDPVIYGLFTIHFRKGLRRYYCNATKASDLDNNT
VITGSFICAANSLPLKREVSPASQERFVLYSDNHSRAELTSPRSSFLRDPNQSSSESNL

#gi|683912473|ref|XP_009088429.1|_PREDICTED:_gonadotropin-releasing_hormone_II_receptor-like_Serinus_canaria
MSEEQLPASPHCPTTREDTNVSASAYPQYWVEPRFTQAAKVRVIITAIFFLLAAGSNAAV
LGSLLRKRRKSHVQPLILSLALADLLVTVMVMPLDAAWNVTVQWYGGDISCKILNFLKLF
AMYAAALVLVVISLDRHAAILHPFSRAHRRNGMLLRAAWAGSMLLALPQLFIFHLNTIPG
RNFTQCVTHGSFRAHWEETVYNMFTFTTLYITPLSVMIVCYIRILWEISKQLKVNKGLTR
NQNDHISKARMKTLKMTIVIVTTFIICWTPYYLLGLWYWFQPAMIQKMPEYVNHSFFLFG
LLHTCTDPIIYGLYTPSFREDVQLCLRGIETAITRQKRHKPISASEKNTKDGAANGGVAS
GGSNGTTVSTV

#gi|686604635|ref|XP_009278739.1|_PREDICTED:_gonadotropin-releasing_hormone_II_receptor-like_Aptenodytes_forsteri
MDAGALSAETRQRVSSSPDMARLGSAGWDTLAGGRGHLDPDPAVGNTSVEPPGSLPPLER
GCAWSPQAEGGEEPLRLPTFSPAAQARVAVTFALFALSAGCNLAVLRVAGGRRSGRRSHI
RLLLLHLAAADLLVTVAVMPLDAIWNITLQWRAGDLACRLLMYLRLLAMYASAFVTVVIS
LDRQAAILHPLAIARARKRNRIMLYIAWLLSAGLSVPQLFLFRTVTLHPPHNFTQCTTRG
SFPQPWHETLYNMLGFACLFLLPLLIMVCCYTRILLEISRRMGSSLFSSRDVSLRCSRNN
IPRARLRMLKMSLVIVSSFILCWTPYYLLGLWYWFCPRAMDKRVSPALTHILFIFGLFNA
CLDPITYGLFTIPFRRGWGCPCGHSPEPQPPSPATGSFRCSASSLPPKRGIPGVRGWRVP
AEPGLPAGAGSCQSSSL

#gi|690437213|ref|XP_009319807.1|_PREDICTED:_gonadotropin-releasing_hormone_receptor_Pygoscelis_adeliae
MSDEQLPASPRCPTAEGDTNISAYGCPEHWVEPRFTQAARVRVIVTAIFFLLAAGSNAVV
LGSLLRKRRKSHVRPLILSLALADLLVTVAVMPLDAAWNVTVQWYGGDVSCKVLNFLKLF
AMYAAALVLVVISLDRHAAVLHPFSRARRRNGLLLRVAWASSVLLALPQLFLFHLHTVPG
GNFTQCVTHGSFRAHWEETVYNMFTFTTLYITPLSVMIVCYIRIIWEISKQLKINKGLIR
NQNDHISKARMKTLKMTIVIVATFIICWTPYYLLGLWYWFQPAMIQKMPEYVNHSFFLFG
LLHTCTDPVIYGLYTPSFRDDVQLCLRGIETAITRHERHKPVSVSEKNIKDGAINGGVAS
GGSNGTTVNTVC

#gi|694671877|ref|XP_009489623.1|_PREDICTED:_gonadotropin-releasing_hormone_receptor_Pelecanus_crispus
MPRPVGRRGQGENRLRMLILAICTSHPFRYGIGRVTVAESRLHCAGRVAGMGMSDEQVPA
SPCHPTAEGDTNASASGCPEHWVEPRFTQAARVRVIITAIFFLLAVGSNAAVLGSLLRKR
KKSHVRPLILSLVLADLLVTVVVMPLDAAWNVTVQWYGGDISCRLLNFLKLFAMYAAALV

LVVISLDRHAAVLHPFSRARRRNGLLLCTAWASSVLLALPQLFLFHLHTVPGRNFTQCVT
HGSFRAHWEETVYNMFTFTTLYITPLSIMIVCYIRIIWEISKQLKINKGLIRNQNDHISK
ARMKTLKMTIVIVATFIICWTPYYLLGLWYWFQPAMIQKMPEYVNHSFFLFGLLHTCTDP
VIYGLYTPSFREDVQLCLRGIETAITRHERHKPVSVSEKNIKDGTANGGVASGVSNGTTV
NTVC

#gi|694853743|ref|XP_009468305.1|_PREDICTED:_gonadotropin-releasing_hormone_II_receptor-like_Nipponia_nippon
MDGDANVSASGCPEHWVEPRFTQAARVRVIITVIIFLLAAGSNATVLFSLLRKRRKSHVR
PLILSLALADLLVTVAVMPLDAAWNVTVQWYGGDISCKVLNFLKLFAMYAAALVLVVISL
DRHAAVLHPFSRARRRNGLLLRAAWAGSVLLALPQLFLFHLHTVPGGNFTQCVTHGSFRV
HWEETVYNMFTFTTLYITPLSVMIVCYIRIIWEISKQLKINKGLIRNQNDHISKARMKTL
KMTIVIVATFIICWTPYYLLGLWYWFQPAMIQKMPEYVNHSFFLFGLLHTCTDPVIYGLY
TPSFREDVQLCLRGIETAITRHERHKPVSVSEKNIKDGAVNGGVASGGSNGTTVNTVC

-
#gi|694854041|ref|XP_009468470.1|_PREDICTED:_gonadotropin-releasing_hormone_receptor-like_Nipponia_nippon
MARLGGAGQDTLAGGRGHPHPEPAVGNASAEPPSSLLPPERGCAWSPQAEGGEEPLRLPT
FSPAAQARVAVTFALFALSAGCNLAVLRVAGGRRGGQRSHIRLLLLHLAAADLLVTVAVM
PLDAVWNITLQWRAGDLACRLLMYLRLLAMYASAFVTVVISLDRQAAILRPLAIARARRR
NRIMLYVAWLLSAGLSVPQLFLFRTVTLRPPHNFTQCTTRGSFPQPWHETLYNMLGFACL
FLLPLLIMVCCYARILLEISRRMGSSLFSSQDVSLRCSRNNIPRARLRMLKMSLVIVSSF
ILCWTPYYLLGLWYWFCPRAMEKRVSPALTHILFIFGLFNACLDPITYGLFTIPFQRGWG
CPCGDKPALLGTGWVGVGPGCSCPCRGVVPGPPGRPSSEGIPRIGQGWVLASAAAAGAVG
PRRAPGPGGARGWSLLVELSWQQSQGTVQPCTRWQLR

#gi|697825712|ref|XP_009633309.1|_PREDICTED:_gonadotropin-releasing_hormone_receptor_Egretta_garzetta
MSDEQLPAAPCRPAAEGDANISASGCPEHWVEPQFTQAARVRVIVTAVFFLLAAGSNAAV
LSSLLRKRRKSHVRPLILSLALADLLVTVAVMPLDAAWNVTVQWYGGDIACKLLNFLKLF
AMYAAALVLVVISLDRHAAVLHPFSRARRRNGLLLRAAWASSVILALPQLFLFHLHTVPG
GNFTQCVTHGSFRAHWEETVYNMFTFTTLYITPLSVMIICYIRIVWEISKQLKINKGLIR
NQNDHISKARMKTLKMTIVIVATFIICWTPYYLLGLWYWFQPAMIQKMPEYVNHSFFLFG
LLHTFTDPVIYGLYTPSFREDVQLCLRGIETAITRHERHKPVSVSEKNIKDGAVNGGVAS
GGSNGTTVNTVC

#gi|698438062|ref|XP_009816572.1|_PREDICTED:_gonadotropin-releasing_hormone_receptor_Gavia_stellata
MLRPGALLQAEPPHHPAAEGDANVSASDCPEHWVEPRFTQAARVRVIVTAIFFLLAAGSN
AAVLGSLLRKRRKSHVRPLILSLALADLLVTVAVMPLDAAWNVTVQWYGGDISCKLLNFL
KLFAMYAAALVLVVISLDRHAAVLHPFSRARRRNGLLLRAAWAGSVLLALPQLFLFHLHT
VPGGNFTQCVTHGSFRAHWEETFYNMFTFTTLYITPLSVMIVCYIRIIWEISKQLKINKG
LIRNQNDHISKARMKTLKMTIVIVATFIICWTPYYLLGLWYWFQPAMIQKMPEYVNHSFF
LFGLLHTCTDPVIYGLYTPSFREDVQLCLRGIETAITRHERHKPVSLSEKNIKDGAVNGG
VASGGSNATTVNTVC

#gi|699627265|ref|XP_009885730.1|_PREDICTED:_gonadotropin-releasing_hormone_II_receptor-like_Charadrius_vociferus
MTETRSQDVNERDGNMGMSDERLPTSSHHPAAEGDTNVSASGCPEHWVEPRFTQAARVRV
IVTAIFFFLAAGSNAAVLGSLLRKRRKSHVRPLILSLALADLLVTVAVMPLDAAWNVTVQ
WYGGDISCKLLNFLKLFAMYAAALVLVVISLDRHAAILHPFSRARRRNGLLLRAAWASSV
LLASPQLFLFHLHTVPGGNFTQCVTHGSFRAHWEETVYNMFTFTTLYITPLSVMIICYIR
IIWEISKQLKINKGLIRNQNDHISKARMKTLKMTIVIVATFIVCWTPYYLLGLWYWFQPA
MIQKMPEYVNHSFFLFGLLHTCTDPVIYGLYTPSFREDVQLCLRGIETAITRHERHKPIS
VSEKNIKDGAVTGGVASGGSNGTTVNTVC

#gi|700420040|ref|XP_009948194.1|_PREDICTED:_gonadotropin-releasing_hormone_II_receptor-like_Leptosomus_discolor
MSEEQLPASPHCPAAEGDTNVSASGCPEHWIEPRFTQAARVRVIITAIFFLLAAGSNAAV
LGSLLRKRRKSHVRPLILSLALADLLVTVTVMPLDAAWNVTVQWYGGDLSCKLLNFLKLF
AMYAAALVLVVISLDRHAAVLRPFSRARRRNGLLLRAAWASSLLLASPQLFLFHLHTVPG
GNFTQCVTHGSFRAHWEETVYNMFTFTTLYITPLSVMIICYIRIIWEISKQLKINKGLIR
NQNDHISKARMKTLKMTIVIVATFIICWTPYYLLGLWYWFQPAMIQKMPEYVNHSFFLFG
LLHTCTDPIIYGLYTPSFREDVQLCLRGIETAMTRHERHKPVSVSENIKDGAVNGGVASG
GSNGTTVNTVC

#gi|701299949|ref|XP_010010396.1|_PREDICTED:_gonadotropin-releasing_hormone_II_receptor-like_Nestor_notabilis
MALEPVLEMKGMSDEQLPASPPFPTIEEDANVSASICPERWVEPRFTQAARVRVIITVIF
FLLAAGSNAAVLGSLLRKRRKSHVRPLILSLALADLLVTVAVMPLDAAWNVTVQWYGGDI
SCKLLNFLKLFAMYAAALVLVVISLDRHAAILHPFSRARRRNGLLLRAAWVGSVLLASPQ
LFLFHLDTAPGGNFTQCVTHGSFRTHWEETVYNMFTFTTLYITPLSVMIVCYTRIIWEIS
KQLKINKGLMRNQSDHISKARMKTLKMTIVIVATFIICWTPYYLLGLWYWFQPAMIQKMP
EYVNHSFFLFGLLHTCTDPIIYGLYTPSFREDVQLCLKGIETAITRHERHKPVSVSEKNT
KDGAVNGGVASGCSNGTTVNTVC

#gi|701379768|ref|XP_009987972.1|_PREDICTED:_gonadotropin-releasing_hormone_receptor_Tauraco_erythrolophus
MSDQQLPASPHRPTTEEDANVSASGCPERWVEPRFTQAARVRVIVTAVFFLLAAGSNAAV
LGSLLKKRRKSHVRPLILSLALADLLVTVVVMPLDAAWNVTVQWYGGDVSCKLLNFLKLF
AMYAAALVLVVISLDRHAAVLHPFSRARRRNGLLLRAAWAGSVLLASPPLFLFHLHTVPG
GNFTQCVTHGSFRAHWEETVYNMFTFTTLYITPLSVMIVCYIRIIWEISKQLKINKGLLR
NHNNHISKARMKTLKMTIVIVATFIICWTPYYLLGLWYWFQPAMIQKMPEYINHSFFLFG
LLHTCTDPVIYGLYTPSFREDMQLCLRGIKTAITRHERHKPVPVSELGREENIKDGAVNG
GVTSGGSNGTTVNTVC

#gi|701422600|ref|XP_010001135.1|_PREDICTED:_putative_gonadotropin-releasing_hormone_II_receptor_Chaetura_pelagica
MDGLFLFRTVTFHPPHNFTQCTTRGSFPQPWHETLYNMLGFACLFLLPLLIMVCCYARIL
LEISRRMGSSLFSSRDVSLRRSQNNIPRARLRTLKMSLVIVSSFILCWTPYYLLGLWYWF
CPRAMEKRVSPALTHILFIFGLFNACLDPITYGLFTIPLPRGWGCPCGPPPEPQPPSPAT
GSFHCSASSSSLPPKRGVPGARGQWEPTRTGLPTGTSSCQSSSL

#gi|704303860|ref|XP_010163755.1|_PREDICTED:_gonadotropin-releasing_hormone_receptor_Caprimulgus_carolinensis
MSDEELPASLRHLTVDGDTNISTSSCPERWVEPQFTPAAKVRVIVTAIFFLLAAGSNAAV
LGSLLKKRRKSHVRPLILSLALADLLVTVVVMPLDAVWNMTVQWYGGDISCKLLNFLKLF
AMYAAALVLVVISLDRHAAVLHPFSRARRRNGLLLRAAWAGSGLLALPQLFLFHLHTVPG
GNFTQCVTHGSFRAHWEETVYNMFTFTTLYITPLSVMIVCYIRIIWEISKQLKINKGLIR
NQNDHISKARMKTLKMTIVIVATFIICWTPYYLLGLWYWFQPAMIQKMPEYVNHSFFLFG
LLHTCTDPIIYGLYTPSFREDMQLCLRGIETAITRHERHKPVSVSEKNIKDGAVNGGVAS
AGSNGTTVNTVC

#gi|705659720|ref|XP_010121386.1|_PREDICTED:_gonadotropin-releasing_hormone_receptor_Chlamydotis_macqueenii
MSEEEHLPTSPHHPTTEGDANVSAAGCPEHWVEPQFTQAARVRVIVTAVFFLLAAGSNAA
VLGSLLRKRRKSHVRPLILSLALADLLVTVAVMPLDAVWNVTVQWYGGDISCKLLNFLKL
FAMYAAALVLVVISLDRHAAVLHPFSRARRRNGLLLRAAWAGSVLLASPQLFLFHLQTVP
GGNFTQCVTHGSFRAHWEETLYNMFTFTTLYITPLSVMIVCYIRIVWEISKQLKINKGLI
RNQNDHISKARMKTLKMTIVIVATFIICWTPYYLLGLWYWFQPAMIHKMPEYVNHSFFLF
GLLHTCTDPVIYGLYTPSFREDVQLCLRGIETALVRHERHKPVSVSEKNIKDGAVNGGVA
SGGSNGTTVNTVC

#gi|70910044|emb|CAI64587.1|_G_protein-coupled_receptor_Crassostrea_gigas
MDEHLSTNESTSLPSYLKFSDNYAHSIISLGVVFLIGALGNIASFVTLLCSKNRKSPTNF
FLLHLSSADLLVMFVIPVGEMIHNATIAWLGGNFLCKLYHFLWNFGQYVATFLLCCISID
RYLAFVFPLRSLTQSPRTRSFMAATAWVLSAILSAPESVIFHVETHPKYRTFRQCVTFNF
FPSHNHELAYNLFNLITLYALPLLIITTSYSLILWEISKKTKQCKEETKCLSTRSRLRRS
SVGNMERARIRTLKMTLVIVSVFVICWTPYFVLSAWWWFDSDSASQLDPKVQRGLFLFAV
SNSCMDPIVYGMFTTTFRRETQKWGRWL

#gi|729735621|ref|XP_010563842.1|_PREDICTED:_gonadotropin-
releasing_hormone_II_receptor-like_Haliaeetus_leucocephalus
MNVTGTAGNWSCASKEPILLPTFSTAAKVRVAVTCLLFLSSACCNGAVLWAAARAPRRRP
PRVRVLMANLAVADLLVTVVVMPLDAAWNITVQWYGGDAACRVLMFLKLAAMYASAFVTV
VIALDRHAAIVNPLAVGCAERRNKAMLCVAWVLSAVLALPQAFIFRTVSRLQPRHFVQCA
TVGSFRAHWQETLYNMFTFTCLFLLPLLVMVLCYGRILAAISGRMKDTRVSSQEIQLRRS
YDNIPRARMRTLKMSIVIVLTFIVCWTPYYLLGLWYWFSPEMLTREKVPPSLSHILFLFG
LFNTCLDPLIYGLFTVHFRREMWRTCRCSHRQHKQEVASALSGSFRVSTTAVPARRAGNS
LGGSGKHELEVMADAALPGRRCELCRRRTVESFM

#gi|733905280|ref|XP_010715876.1|_PREDICTED:_gonadotropin-
releasing_hormone_II_receptor-like_Meleagris_gallopavo
MCVPAALIKAELPHHPTTEGVTNASAAHCPENWVEPRFTQAAKVRVAITAIFFLLAACSN
TAVLGSLLRKRRKCHVRPLILSLVLADLLVTAVMPLDAAWNVTVQWYGGDLSCKLLNFL
KLFAMYAAALVLVVISLDRHAAVLQPFARARRRNGLLLRAAWLGSVLLASPQLFLFHLHT
VPGGNFTQCVTHGSFRAHWEETVYNMFTFTTLYITPLSIMIVCYVRIIWEISKQLKINKS
LIRSQNDHISKARMKTLKMTIVIVATFIICWTPYYLLGLWYWFQPAMIQRMPEYINHSFF
LFGLLHTCTDPIIYGLYTPSFREDVQLCLRGIEAAITRHERHKPILVSEKTTKDGAVNGQ
VASGGSNGTTINTVC

#gi|733905578|ref|XP_010715998.1|_PREDICTED:_putative_gonadotropin-
releasing_hormone_II_receptor_Meleagris_gallopavo
MRPGLPALPRAAPPRSELPRVLLQRAVWGTSWESGYCATISSKSESCLICSSCMECLRRS
FFLVLRSPRLGELGATGTRALKPAASGEGALPLGRGLHLPPPLCFQLFLFHTVTLRAPHN
FTQCTTRGSFPQPWHETLYNMLSFSCLFLLPLLIMVCCYTRILLEISRRMGSSLFSSSDV
PLRCSGSNIPRARLRTLKMSLVIVSSFIFCWTPYYLLGLWYWFCPRAMQEKVPPSLSHIL
FIFGLFNACLDPITYGLFTIPFQRRCSCPCGHSPEPEPPSSATASFQCSASSLRGRQSTG
GTEGPQPPIELGLPTGASSCQSSAP

#gi|734595245|ref|XP_010730519.1|_PREDICTED:_putative_gonadotropin-
releasing_hormone_II_receptor_Larimichthys_crocea
MNASSCCDSPVIMYQESSESDLNASCDWLAPHCNWTSVDRAPQLPTFSTAAKIRVIITFT
LCGISTFCNLAVLWAINSHKRKSHLRVLIINLTAADLLVTFIVMPVDAVWNITVQWLAGD
LACRFLMFLKLQAMYSCAFVTVVISLDRQSAILNPLAISMARRRNRVMLIVAWTMSTLFS
IPQMFIFHNVTITYPANFTQCTTRGSFVTHWQETAYNMFTFSCLFLLPLVIMIICYTRIF
IQISKRMTKKNLSSSESDLRCSKNNIPKARMKTLKMSIVIVMCFIVCWTPYYLLGLWYWF
FPDDIEGKVSHSLTHILFIFGLFNACLDPIIYGLFTIRFRKGLRSCYRKAAAKSDLGTNI
TEPLKSTTASLPSKRGTSSGEKDRNCGQVKPKPSDGRHLTVFIRGEGGQKSNSSADSTA

#gi|734610510|ref|XP_010732618.1|_PREDICTED:_gonadotropin-
releasing_hormone_II_receptor-like_Larimichthys_crocea
MFHQLTDPTVNGSCQGPTSACNKSADGDALQLPTFSTAAKVRVIITFTLCAVSAMCNLAV
LWAASNGGKRKSHVRILIMNLTVADLLVTFIVMPVDAVWNITVQWQAGDVACRLLMFLKL
VAMYSCAFVTVVISLDRQSAILNPLGISEAKRKSKIMLTVAWTMSVILSLPQMFIFHNVT
ITVPENFTQCTTHGSFVQHWQETLYNMFTFVCLFLLPLVIMIFCYTRILIEISSRMARNN
LLSRDVHLRRSHNNIPKARMRTLKMSIVIVTSFIICWTPYYLLGLWYWLFPEKMEETVSH
SLTHMLFIFGLFNACLDPITYGLFTIHLHQGLKRCCRNANTRTELEKNTSLVHMTRLSSK
RQTASGGHNTDIEEGNDNNSTKSVSRPIIPVSKM

#gi|734636182|ref|XP_010746719.1|_PREDICTED:_gonadotropin-releasing_hormone_II_receptor-like_Larimichthys_crocea
MAGNLSADPFQNDSIFSLLDSSTPTSPPPWKAPTFTVAARCRVAATMVLFVFAAGSNLSV
LISVCWGRGYRLAAHLRPLIASLASADLVMTFVVMPLDAIWNITVQWYAGDIMCKLLCFL
KLFAMHSAAFILVVVSLDRYRAILHPLDSLDAGLRNRRMLLVAWTLSLLLASPQLFIFRA
IKADGVDFTQCVTHGSFRYLWQKTAYNMFHFVTLYVFPLLVMTFCYTRILTKINGQMRKS
KDGEHCLRRSGTDMIPKARMKTLKMTIVIVSSFVICWTPYYLLGIWYWFQPAMIQHTPEY
VHHILFVFGNLNTCCDPVIYGFFTPSFRADLADVMACCRGRRASNASPRSVDRLSARAGA
AVEMESDLSSNQHSGNPG

#gi|74136245|ref|NP_001028014.1|_gonadotropin-releasing_hormone_II_receptor_Macaca_mulatta
MSAGNGTPWGSAAGEESWAASGVAVEGSELPTFSAAAKVRVGVTIVLFVSSAGGNLAVLW
SVTRPQPSQLRPSPVRTLFAHLAAADLLVTFVVMPLDATWNITVQWLAEDIACRTLMFLK
LMAMYSAAFLPVVIGLDRQAAVLNPLGSRSGVRKLLGAAWGLSFLLALPQLFLFHTVHRA
GPVPFTQCVTKGSFKARWQETTYNLFTFRCLFLLPLTAMAICYSHIVLSVSSPQTRKGSH
APAGEFALCRSFDNCPRVRLWALRLALLILLTFILCWTPYYLLGLWYWFSPTMLTEVPPS
LSHILFLFGLLNAPLDPLLYGAFTLGCQRGHQELSIDSSNEGSGRMLQQEIHALRQQEVQ
KTVTSRSAGETKDISITSI

#gi|76253723|ref|NP_001028997.1|_gonadotropin-releasing_hormone_receptor_1_Ciona_intestinalis
MMTSDISQGATNIDNNWTSTAAVLGLNSTVNSTALPCEAHDVLIQTWFQFDTLHLVRVLV
TWLLFCLSMAGNMFVLWSLRGSKSRHFIMFHLALSNLIYTIFVMPSDAVWNTTMEWLAGD
VMCRLCQMMKQFGMYASSFMVVVIGADRVTGILSPLPCHSQRKRGYYMVATAWISSLICC
LPAGFIFSVASIPTCEGIPIYQCIDFNVLQDVSLLRPYYFFTMCMSFLLPLICTLVSYSL
IVCEISTMKERDRVLMGRRHSVNTASIQRAKNRTILMRTLITLTFLVCWGPYYGKGIYDW
FIRYEDHTPPDAWDTVMYVVMYLNPVLHPIVFGVFLKEIRGKFKQRLNCARKRFFKQGDF
KTVPNAQSSMNYSIASVLNRPRRMSSTSRGSFSSYATGATHLNGSSHVTINGQCSNNGSN
GSIKTQPQFFGANRMVAPQQQLLLSSESAL

#gi|76253773|ref|NP_001028996.1|_gonadotropin-releasing_hormone_receptor_2_Ciona_intestinalis
MTTAAFTQDYVDGIYPSTATDTFMPMCQSHDTIIQTWFQFDTLYLVRVLVTWLLFCLSMA
GNMFVLWSLRGSRSRHFIMFHLALSDLIYTIFVMPSDAVWNTTMEWLAGDVMCRLCQMMK
QFGMYASSFMVVVIGMDRVTAILSPLTHEGQRKRGYCMVLAAWTTSLLCCIPAGALFSLL
TVETCEGINVYQCVDFNIVKDRSLLRPYYFFTMCMSFLLSLICTLVSYSLIVCEISNMKE
RDRVLMGRRKSVNTASIQRAKNRTILMRTLITLTFLVCWGPYYGKGIYDWFQKPAIGPPA
PLDTAMYIVMYLNPVLHPIVFGVFMKEIRSKFKKTSCVAGLKRKRVQVRSGSVSRLSLTS
YATGLTQVQYLSNGAAETTVLTGATQQVPSLDESVHTKAPE

#gi|76253787|ref|NP_001028995.1|_gonadotropin-releasing_hormone_receptor_3_Ciona_intestinalis
MATVSSLVTTAATAMDGTTMSTPITSTFDINASYHSLNATLDSNSTYFEKWCPYYHKMLT
FNTIQLTRVIITWILFLVSTCGNSFVLYCLCKQKQRLHVHVITMHLTLADLAFTFFSMPM
DATWNTTMAWLGSEFLCRLCQFLKQFGMYISSLMVVVIALDRVFSILSPMSANQQRKRTK
ILLISAWTLSLLCAIPALFLFSLIRKQFCPDQPIFHQCVDFSRNINKQDLKPYYFFTMCV
SFLIPLFFTVISYSLILCEINAMQRRDERITGRRDNNIERARMKTLVLTSLVTLSFIVLW
GPYYAMGIYHWFNPIERATFPKEISVGLFVLMYFHPAVHPILYGFFMKDIRKHFLATLMR
CFKLSRIPASRRASDKFGSCQRHLPEGNPHPRAGRAASSPLLSPVTERTVLRATSVPFID
AVKRNQNNNRQEIFLQVPPTNGELSTQRCCDERV

#gi|76253816|ref|NP_001028994.1|_gonadotropin-releasing_hormone_receptor_4_Ciona_intestinalis
MMTSPTMTSNITGCNVTSTYSPEFIIENQYDCDAMFESFLSSQRLVFDSYHLTRIWVTWV
IFFISLAGNLTVLISVTVLRKTQSYSHCQLIMTHLSLANLAFTLFVLPMDAIWNYTLEWL
AGDVMCRIMNSLKQFAMYISSAMIMVMGVDRVTGLLRPVSANQQRRRIVKFLTVAWVFSF

INSIPPSVMFSAGPYWPMRCECPNHYVVQCVDFHLIKKGREIFYIYSMFISFFIPLYCII
ICYLIIAFSIAKMAKRAKATELQSSGFRPPSSRKSLARRSLQRAKKISQLVTGLITITFV
ICWGPYYVVGLMHWFNEQHIERLPEGIMLMSLYLNPCLHPFITVCLMKEIRESICRKPNC
SLATPR

#gi|76880395|dbj|BAE45694.1|_type_1/III-1_gonadotropin-
releasing_hormone_receptor_Tetraodon_nigroviridis
MNQSSQRPALLHPAWELLDPGCQPTSAPLPRLPTFTAAAKARVFITFVLCGVSALCNLAV
LWAASGQRRRSRVHLLIVHLSVADLLVTFVVMPVDAAWNLTVQWLAGDLACRILMFLKLQ
AMYSCAFVTVVISLDRQWAVLHPLAVVTARRRNKALLVAAWTTSVLLSLPQIFLFHKVSI
TRPANFTQCTTRGSFSSRWQETAYNLLTFACLFLLPLALMVTCYTRIFLHISRRLARRRP
SSGEPDLRRSRDNIPRARRRTLKMSIVIVLCFVLCWGPYYLLGLWYWFFPEHLEGKVSHS
LTHILFIFGLFNTCADPLVYGLFTVRFRRGS

#gi|76880399|dbj|BAE45696.1|_type_1/III-2_gonadotropin-
releasing_hormone_receptor_Tetraodon_nigroviridis
MSRTSSFQPDFTPAIPCQLTLSAAYNHSRSWSNLTVAGGTPLLPTFGAAARVRVAVTWTL
CLLSAFCNLAVLRAARSGGAGRRSRVRMLMVHLTSADLLVTFIVMPVDAVWNVTVQWLAG
DLACRALMFLKLQAMYSCAFVTVVISLDRQSAILHPLAITEAGRRNKVMLAVAWVMSFAL
SLPQIFIFHSVTIIRPEAFTQCTTRGSFPTRWHETAYNMFTFSCLFLLPLVVMVTCYTRI
LVEISKRVENNSSSNQVCLRRSRNNIPRARMRTLKMSIVIVASFIICWTPYYLLGLWYWF
FPEDLEGKVSHAFTHILFIFGLLNACLDPLIYGLFTIRFRKGLRRLFAGGAEAAEPENNT
VLSGSFTRATSSLPLRSQVIPASPESHDAAPLTHTHTHSSSVNLQGCSTQKLHLCST

#gi|76880403|dbj|BAE45698.1|_type_1/III-3_gonadotropin-
releasing_hormone_receptor_Tetraodon_nigroviridis
MFHQLMDAALNDSCAGATSGCNRSSAGDALQLPTFSTAAKVRVIITFSLCAVSAVCNLVV
LWAAGNGGKRKSHVRILIMNLTVADLLVTFIVMPVDAVWNITVQWQAGDAACRLLMFLKL
VAMYSCAFVTVVISLDRQSAILNPLGISEAKRKSKITLAVAWTMSVVLSLPQMFIFHNVT
ITVPENFTQCTTHGSFVQHWQETLYNMFTFACLFLLPLIIMIFCYTRILVEISSRMARNN
LLSRDVHLRRSHNNIPKARMRTLKMSIVIVTSFIVCWTPYYLLGLWYWLFPEKMEETVSH
SLTHMLFIFGLFNACLDPITYGLFTVRLHQGLKRCCRTANTQTDLENNTCLVHMTRFSSQ
RHYASGAHETHKEMIDDTCTNNCSRPIIPVSKM

#gi|76880407|dbj|BAE45700.1|_type_2/nmI-1_gonadotropin-
releasing_hormone_receptor_Tetraodon_nigroviridis
MSTNVSLFLPPSSSPPWELPSFSVAARCRVATTLVLFVFAAASNLSVLISVFWGRGYRLG
AHLRPVIASLAAADLMMSFIVMPLDAVWNITVQWYAGDVMCKLMCFLKLFAMHSAAFILV
VVSLDRYRAILHPLDSLDAVRRNRRMLAVAWSLSVLLASPQLFIFRTIKAEGVDFTQCVT
HGSFLHLWQETTYNMFHFVTLYIFPLLVMIFCYTRIFTKINGQIHENKDNESCLRRSGGD
VIPKARMKTLKMSVVIVSSFVICWTPYYLLGIWYWFHPAMIQHTPEYVHHMLFVFGNLNT
CCDPIIYSFYTPSFRADLADVVACCCARRAADASPRSVDRLSAPAGGAGVDMESDLSSNQ
QSSNAA

#gi|78098578|gb|ABB20590.1|_adipokinetic_hormone_receptor_Periplaneta_americana
MALSSCSDQTLNCTAVITMATPTPSPTVLMEEYMTDDMKFNDGHRMSIITYSILMVVSVA
GNSTVLITILKRRRTLRYGNNYMFMHLAIADLLVTFLMMPLEIAWNITVSWKGGDLMCRI
MLFFRTFGLFLSSFVIVCISLDRCVAILRPMSKKLLNVARRGKLMLTVAWILATLCSLPQ
AVIFHVEPHPNVTWYEQCVSFNFFSTKMHEFTYRVLGMVMMYGLPLIVIVISYACILGEI
IRRYQLSPDDSFRRSSLVFLNRARNRTLKMAIIIFVVFFICWTPYYVMCLWYWIDERSAE
TVDHRVQKALFLFASTNSCMNPIVYGYFNFRLGRGSGYGATGGRVGQQLHHQNVVALSGN
STGLNSRRGSNSSSIYRNNSNQSMSWKNSLRSGRRNSRETEHLHPLPHRNSAQAITNVNG
RDDQHQLHTNSNKTPNVLEDASNSNSKNALTSVICR

#gi|82754176|gb|ABB89900.1|_type_1/III_gonadotropin-
releasing_hormone_receptor_Eublepharis_macularius
MVPEMNTSMVDGPVPWESLFPLVGVAPANCSQEEALALPTFSAAAQARVVITLLLCVFST

ACNVAVLWAGVGRSHAKRSHARVLLLHLAGADLLVALVVMPLDAAWNITVQWRAGDMACR
LLMFLKLLAMYASAFVTALISLDRQGAILHPLAFAEAGQRSQVLLQAAWLLSVALSVPQL
FLFHTITISAPQNFTQCTTRGSFAQRWHETAYNMLTFSCLFLLPLLIMLSCYSRILLEIF
YRTGSTSTLSAMELPLRRSRNPIPQARLRMLRLSVAIVCSFVVCWTPYYLLGLWYWFWPA
AMESQVSHSLAHLLFIFGLLNACLDPVIYGLFTMPWPWRLRGCCAEDGPGLQPQPSSSVT
GSFHCSASTVCDRRGTSHLGLVEAARPPSVWTKGPIHGSCL

#gi|82754178|gb|ABB89901.1|_type_3/II_gonadotropin-
releasing_hormone_receptor_Eublepharis_macularius
MNTTFPMKVHKMVESGLSIVDNQSHGKPNSIEEIWADGTTNGSCSGEELFQLPTFSTAAK
VRVAITIVLFLSSAFFNIAVLWTITQKYHKRPHLRILLMNLAAADLLVTFVVMPLDAVWN
ITVQWYAGDVACRLLMFLKLVAMYASAFVTVVISLDRRAAILNPLSVGEAKRKNKVMLCV
AWALSLLLALPQMFVFHTVSRSQPVYFIQCATVGSFHAHWQEILYNMFTFCFLFLLPLLI
MVVCYSRIFIEISRKMKKACAPPKSREFHLRRSYNNIPRARMRTLKMSVVIVLTFVVCWT
PYYMLGLWYWFSPEMLTREQVPPSLSHILFLFGLFNACLDPIIYGLFAVHFRVESKQGCC
CGRRKKEPEASSMFTSSFRASTTMPVRQPEDIREKYKAEFTVGESPPGAKLPLYRRKMVE
SFI

#gi|83033264|gb|ABB97085.1|_gonadotrophin-
releasing_hormone_receptor_2A_Micropogonias_undulatus
MNTTLCDSEATMYHLMTDHQLNASCNYSSPTSNWTAGGGALQLPTFTTAAKVRVIITCIL
CGISAFCNLAVLWAAHIDGKRKSHVRVLIINLTVADLLVTFIVMPVDAVWNITVQWLAGD
FACRLLMFLKLQAMYSCAFVTVVISLDRQYAILNPLAINKARKRNRVMLTVAWGMSIALS
VPQIFLFHNVTIIHPEDFTQCTTRGSFVTHWHETAYNMFTFSCLFLLPLIIMITCYTRIF
CEIPKRLNKDNLPSNEIHLRRSKNNIPRARMRTLKMSIVIVSSFIICWTPYYLLGLWYWF
FPDDLEGKVSHSLTHILFIFGLVNACLDPVIYGLFTIHFRKGLRRYYCNATTVSDLDNNT
VITGSFSCTANSLPLKRHVSPASQEMFILCSDNCKSASPRSSFLTADIGAETDPNQSSSE
SII

#gi|871209077|ref|XP_012941361.1|_PREDICTED:_gonadotropin-
releasing_hormone_II_receptor-like_Aplysia_californica
MPETHSCITSQKVLTKFTTTVSLIAMLFPAAMALATKHLHPPMTARLQEDPLSLQSPSAL
RSTVSGTENASSLDHTYEVQVVVEREFSAKSGREDEINALNTAAAVAATAASTTTPINNN
SYSHCINLTLSEIQTNYTECLHPLPKELYFTDDNAVSVAAYSCLFVVAAVGNLTVFITLF
RNRGVKSRINMFIMHLAIADLIVTFMMLPLEIAWHSTVAWKAGDAVCRLLMFFRAMGFYL
SSCILVSISLDRYFAIMKPLSISDAGNRSKMMLTISWFLSIVASIPQSIIFHVERHPKYK
WFEQCVTFNFFPTPEHELAYNLFSIIALYGLPLVIITSSYCVILVRISKKSRQSKDEMRI
SGYDGTPAEQGGLRRSGIGNIERARSRTLKMTLVIVGAYVLCWTPYFVISAWYYFDRTSA
VKIDGKVQRGLFLFAVSNSCINPIVYGMFTAAFRRESKRWGDWLKTTFSYRGSGPRTYCG
AGASHHCAL

#gi|871247004|ref|XP_012940604.1|_PREDICTED:_gonadotropin-
releasing_hormone_receptor-like_isoform_X1_Aplysia_californica
MDGTDSDGSILQRANQAFLASSSGPLQLPIPYNFSAQNSSVHRKRHDVYSPRSAHHDFIL
ASDNISSLLLNNTTAPNMISNNSSFEFQFLNTTHPSNVTFENAPTFTTTSLIKTIVFGIMF
GISFVGNMATIVQMRRLRRRKSTINTLIVNLALADLLVTFFCIAGEAAWTVTVQWLAGNV
MCKFVKYMQVFALYLSTYITVAISLDRCVAILDPMRRNGAAQRVRTMIVFAWIFSALFSI
PQPIVFNVLRGPFKEVFYQCVTFGSYDSAWQLQMYAIASLMLMFVLPLAVMGTAYGLIFT
TISRKSKEHSVMDSIQMPARKTWVTRWVNYKRYLCMAFCSKPISRRSSIMSNYSEDHAAR
GPVRSYLLRKAKRKSLIMSFVIVLAFMVCWTPYYIIFICITFLDKVIDPVIFNYFSFIGL
SNSMLNPMIYGAFQLCKVQFYNPSSWRRNVWRGTSPKLQSSNPNRRSDFPPGTPASSSIS
RDSRNRLLGQGQGCHCQTCHYEAKEQKVCIPALDRKAQASKTSTKSSERCGKFTHKHFTN
GEVKHCLCFHSY

#gi|871247011|ref|XP_012940606.1|_PREDICTED:_gonadotropin-
releasing_hormone_receptor-like_isoform_X2_Aplysia_californica
MDGTDSDGSILQRANQAFLASSSGPLQLPIPYNFSAQNSSVHRKRHDVYSPRSAHHDFIL

ASDNISSLLLNTTAPNMISNNSSFEFQFLNTTHPSNVTFENAPTFTTTSLIKTIVFGIMF
GISFVGNMATIVQMRRLRRRKSTINTLIVNLALADLLVTFFCIAGEAAWTVTVQWLAGNV
MCKFVKYMQVFALYLSTYITVAISLDRCVAILDPMRRNGAAQRVRTMIVFAWIFSALFSI
PQPIVFNVLRGPFKEVFYQCVTFGSYDSAWQLQMYAIASLMLMFVLPLAVMGTAYGLIFT
TISRKSKEHSVMDSIQMPARKTWVTRWVNYKRYLCMAFCSKPISRRSSIMSNYSEDHAAR
GPVRSYLLRKAKRKSLIMSFVIVLAFMVCWTPYYIIFICITFLDKVIDPVIFNYFSFIGL
SNSMLNPMIYGAFQLCKASLSQCSWRRNVWRGTSPKLQSSNPNRRSDFPPGTPASSSISR
DSRNRLLGQGQGCHCQTCHYEAKEQKVCIPALDRKAQASKTSTKSSERCGKFTHKHFTNG
EVKHCLCFHSY

#gi|871247014|ref|XP_012940607.1|_PREDICTED:_gonadotropin-
releasing_hormone_receptor-like_isoform_X3_Aplysia_californica
MDGTDSDGSILQRANQAFLASSSGPLQLPIPYNFSAQNSSVHRKRHDVYSPRSAHHDFIL
ASDNISSLLLNTTAPNMISNNSSFEFQFLNTTHPSNVTFENAPTFTTTSLIKTIVFGIMF
GISFVGNMATIVQMRRLRRRKSTINTLIVNLALADLLVTFFCIAGEAAWTVTVQWLAGNV
MCKFVKYMQVFALYLSTYITVAISLDRCVAILDPMRRNGAAQRVRTMIVFAWIFSALFSI
PQPIVFNVLRGPFKEVFYQCVTFGSYDSAWQLQMYAIASLMLMFVLPLAVMGTAYGLIFT
TISRKSKEHSVMDSIQMPARKTWVTRWVNYKRYLCMAFCSKPISRRSSIMSNYSEDHAAR
GPVRSYLLRKAKRKSLIMSFVIVLAFMVCWTPYYIIFICITFLDKVIDPVIFNYFSFIGL
SNSMLNPMIYGAFQLCKVQFYNPRFVLRLTGRLPCTFLLRCMMAVRSAYPPFI

#gi|871247017|ref|XP_012940608.1|_PREDICTED:_gonadotropin-
releasing_hormone_receptor-like_isoform_X4_Aplysia_californica
MDGTDSDGSILQRANQAFLASSSGPLQLPIPYNFSAQNSSVHRKRHDVYSPRSAHHDFIL
ASDNISSLLLNTTAPNMISNNSSFEFQFLNTTHPSNVTFENAPTFTTTSLIKTIVFGIMF
GISFVGNMATIVQMRRLRRRKSTINTLIVNLALADLLVTFFCIAGEAAWTVTVQWLAGNV
MCKFVKYMQVFALYLSTYITVAISLDRCVAILDPMRRNGAAQRVRTMIVFAWIFSALFSI
PQPIVFNVLRGPFKEVFYQCVTFGSYDSAWQLQMYAIASLMLMFVLPLAVMGTAYGLIFT
TISRKSKEHSVMDSIQMPARKTWVTRWVNYKRYLCMAFCSKPISRRSSIMSNYSEDHAAR
GPVRSYLLRKAKRKSLIMSFVIVLAFMVCWTPYYIIFICITFLDKVIDPVIFNYFSFIGL
SNSMLNPMIYGAFQLCKFLEKKRMARNVAKAAELQPKPPLRFSARHTC

#gi|89039345|gb|ABD60146.1|_adipokinetic_hormone_receptor_Anopheles_gambiae
MPNTMAAHINQRIEDHRNLADWSYYANETAGEEYYEMPIDMRFNSGHILSIMVYTTLMVF
SATGNLTVLSILAQRKVRASSRINIMLAHLAIADLLVTFLMMPLEIGWAYTVRWTAGDLM
CRVMAFFRTFGLYLSSFILICISVDRYFAVLKPLKVHEHRAVLMIAAAWIMSGLCSLPQA
FIFHLEGHPNITGYQQCVTYHYFEEEIYQIIYNVLVMCLMYTFPLIVILYCYGSIYYEIF
SRTNPRNLESFRRSSIDVLGRAKRKTLRMTIMIVIVFVVCWTPYYVMSLWYWLDKESTKN
VDQRIQKGLFLFASTNSCMNPVVYGVFNVRKKHTKKLLKTTHEKSCGSHLTMRA

#gi|90074697|dbj|BAE87048.1|_gonadotropin-releasing_hormone_receptor_type_1-
1_Colisa_lalia
MKASSSSSCCERPVIVHQQSPASGLNLTGCDALDPHCDWTLEGSALELPTFSTAAKVRVI
ATFMLCGISTLCNLAVLWAASGHKRKSHVRVLIVNLTAADLLVTLIVMPVDAVWNITVQW
LAGELACRVLMFLKLQAMYSCAFVTVVISLDRQSAILHPLTIGMARRRNKVMLMVAWSMS
ALLSIPQMFLFHNVTITYPANFTQCTTRGSFATHWQETAYNMFTFGCLFLLPLVIMVVCY
TRIFIQISRRMMKSSFSSGEPHLRCSKNNIPKARMRALKMSVVIVICFIVCWTPYYLLGL
WYWFFPDHIEGKVSHSLTHILFVFGLFNACLDPVIYGLFTIRFSRGLKSCCRKAAVMSDN
EADSAMTESLKYTALPSKRGRGSGQKVCGQAEVGPSDASCSTVFRLEDRGQHS

#gi|90074699|dbj|BAE87049.1|_gonadotropin-releasing_hormone_receptor_type_1-
2_Colisa_lalia
MYRLTTDHWLNTSCNCSSPLTNWSSERDSLQLPTFTTAAKVRVIITFILCGVSAFCNLAV
LWAAHSDGKRKSHVRVLIVNLTVADLLVTFIVMPVDAVWNITVQWLAGDFACRLLMFLKL
QAMYSCAFVTVVISLDRQSAILNPLDINKARKRNRVMLAVAWGMSVMLSVPQMFLFHSVT
ITHPEAFTQCTTRGSFTVHWHETAYNMFTFTCLFLLPLIIMITCYNRIFCEISKRLKRDN
LSSSEVHLRCSKNNIPRARMRTLKMSIVIVLSFIVCWTPYYLLGLWYWFFPDDLEGKVSH

SLSHILFIFGLVNACVDPVIYGLFTIHFRKGIRRQYCNATTASDVDNNTVISGSFTCGSL
RTKRDVSSASPERFTLCGDSKAEKDLIQPSPESFT

#gi|90074701|dbj|BAE87050.1|_gonadotropin-releasing_hormone_receptor_type_2-1_Colisa_lalia
MSGNLSQWVSTPAEHAPNSSAPTAPPPWEAPSFTVAARCRVAATLVLFAFAAASNLSVLI
SVCWGRGYRLAAHLRPLIASLASADLMMTFVVMPLDAVWNITVQWYAGDFLCKLLCFLKL
FAMHAAAFILVVVSLDRYRAILHPLDSLDAGLRNKRMLPVAWVLSLLLASPQLFIFRAIK
AEGADFTQCVTHGSFPQRWQETAYNMFNFLTLYVFPLLVMIFCYMRILSKINGQMRRNKD
GEHCLRRSGTDVIPKARMKTLKMTIVIVSSFVICWTPYYLLGLWYWFQPAIIKFTPDYVH
HVLFVFGNLNTCCDPVIYGFYTASFRADLADTLSCCCGRRANGALQHSVDRLSGHSPGVS
GEMESDLSSTSPVETPDKLW

#gi|94400905|ref|NP_001035354.1|_adipokinetic_hormone_receptor_Apis_mellifera
MESSIKIITTTGLENWRVNNSNYTELLPIDMRFNEGHIVSIVFYSVLMIISAIGNTTVLI
LITCRKRVSKSRIHIMLMHLAIADLLVTFLMMPLEIGWAITVSWKAGDVMCRIMAFFRMF
GLYLSSFVLVCISMDRYYAVIKPLQLWDVDKRGKIMLSFAWIGSVVCSLPQTIVFHLETH
PNVTWYSQCVTFNAFPTYTHEITYSLFGMIMMYWFPLVVIIYTYTSILLEIRRRSKKSED
DKIRRSSIGFLTRAKIRTLKMTVIIIAVFFICWTPYYVMSLWYWIDRNSAYKIDQRIQKG
LFLFACTNSCMNPIVYGAFNIRDRNKTSARPTTIETRVTPLSLSLKLLD

#gi|9857657|dbj|BAB11961.1|_gonadotropin-releasing_hormone_receptor_Anguilla_japonica
MRMSENSSLLMLGSQGTSMNSSSNSSVSPPSADWVAPTFTRAAQFRVVATLVLFLFAAFS
NLAVLISVARGRGRRLASHLRPLIMSLAVADLMMTFIVMPLDMVWNVTVQWYAGDAMCKL
LCFLKLFAMHSSAFILVVISLDRHHAILRPLDSFDAKRRNKRMLLLAWSLSLLLASPQLF
IFRVIKAEGVDFTQCVTHGSFQQRWQETVYNMFQFVTLYVVPLLVMSLCYTRILIEINHQ
IHKSKGGESCLRRSGTDMIPKARMKTLKMTIIIVVSFVVCWTPYYLLGIWYWFHPQMIQV
TPEYVNHILFVFGNLNTCCDPVIYGLYTPSFRADLAVCWFCRPQDSSPKSLDRLSARQGN
PSAEQESDAPSVDQMRANEE
