# Supplementary figures and images for: Functional Authentication of a Novel Gastropod Gonadotropin-Releasing Hormone Receptor Reveals Unusual Features and Evolutionary Insight
Source: PLoS One. 2016 Jul 28;11(7):e0160292. doi: 10.1371/journal.pone.0160292 (PMC4964986; doi:10.1371/journal.pone.0160292)

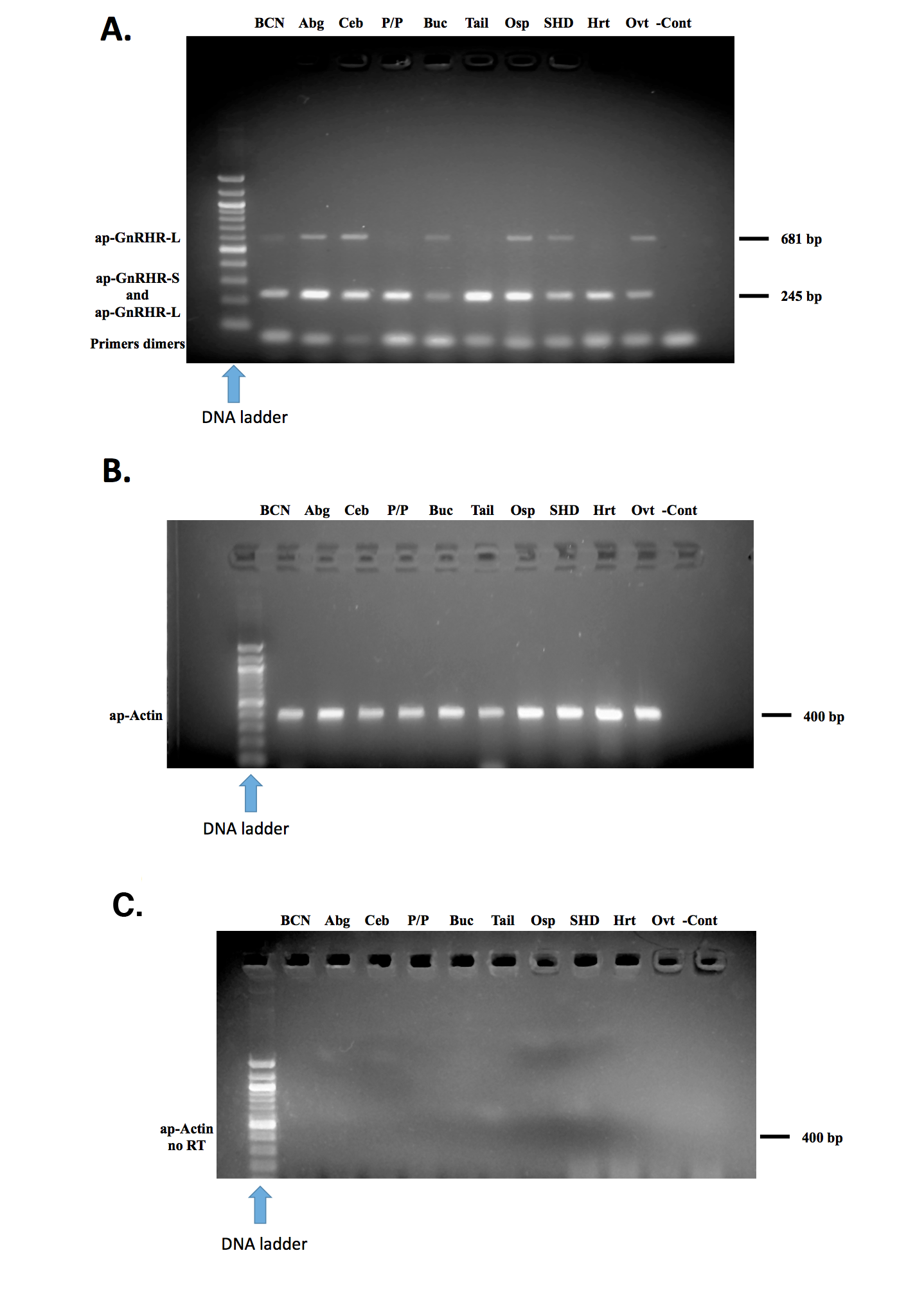

Supplement: S1 Fig — Amplification of ap-GnRHR-L and ap-GnRHR-S (A), ap-Actin (B), and ap-Actin using RNA samples that have not been reversed transcribed (C) are shown. Tissue abbreviations are indicated in Fig 3 legend. The 12 visible DNA ladder bands are: 1517, 1200, 1000, 900, 800, 700, 600, 500, 400, 300, 200, and 100 bp (New England Biolabs, Ipswich, MA). (TIF) [file pone.0160292.s002.tif]
